# Synthesis and Cytotoxic Activity of a New Family of α-Hydroxyphosphonates with the Benzothiophene Scaffold

**DOI:** 10.3390/ph18070949

**Published:** 2025-06-24

**Authors:** Mátyás Milen, Tamás Miklós John, Anna Sára Kis, Zsófia Garádi, Zsuzsanna Szalai, Angéla Takács, László Kőhidai, Konstantin Karaghiosoff, György Keglevich

**Affiliations:** 1 Directorate of Drug Substance Development, Egis Pharmaceuticals Plc., P.O. Box 100, 1475 Budapest, Hungary; johntamas@edu.bme.hu (T.M.J.); garadi.zsofia@egis.hu (Z.G.); 2 Department of Organic Chemistry and Technology, Faculty of Chemical Technology and Biotechnology, Budapest University of Technology and Economics, Műegyetem rkp. 3, 1111 Budapest, Hungary; kisannasari@gmail.com (A.S.K.); szalai.zsuzsanna@edu.bme.hu (Z.S.); 3 Institute of Genetics, Cell and Immunobiology, Semmelweis University, Nagyvárad tér 4, 1089 Budapest, Hungary; takacs.angela@semmelweis.hu (A.T.); kohidai.laszlo@semmelweis.hu (L.K.); 4 Department Chemie, Ludwig-Maximilians-Universität München, Butenandtstr. 5-13, D-81377 München, Germany; klk@cup.uni-muenchen.de

**Keywords:** α-hydroxyphosphonate, Pudovik reaction, X-ray structure, biological activity, cytostatic effect

## Abstract

**Background**: α-Hydroxyphosphonates, one of the most prominent classes of phosphonates, remain of utmost importance because of their potential and real biological activity as pharmaceutical or pesticide agents. The effect is the consequence of their enzyme inhibitory properties. **Objectives**: It was planned to make available new heterocyclic hydroxyphosphonate derivatives with cytotoxic activity. Methods: After optimizing the synthesis, 23 members of a new family, α-hydroxy-α-(benzothiophen-2-yl)-methylphosphonates, were prepared by the Pudovik reaction of benzo[*b*]thiophene-2-carboxaldehydes and diethyl phosphite. The addition was performed at 26 °C in the presence of triethylamine as the catalyst. One of the products was also characterized by single-crystal X-ray analysis. **Results**: The cytotoxic effect of the α-hydroxy-α-benzothiophenyl-methylphosphonates was tested on U266 myeloma, A2058 melanoma, HT-29 colon, and EBC-1 lung cancer cell lines. Most of the molecules showed significant activity; the greatest effects were seen after treatment with hydroxyphosphonates with a trifluoromethyl group in the benzene ring. **Conclusions**: The cytotoxic activity of the newly synthesized α-hydroxyphosphonates is encouraging to find even better derivatives.

## 1. Introduction

The large family of α-hydroxyphosphonates and their derivatives is still in the focus these days due to their biological activity [1,2]. A broad scale of the activity has been described, including pesticidal [3], insecticidal [4,5], and herbicidal [3,6] effects. As regards the pharmaceutical relevancies, antibacterial [7,8], antimicrobial [7,8,9,10,11], antifungal [7,8,11], and antioxidant [12,13] activities were described. An actual challenge is the applicability of hydroxyphosphonic derivatives in the treatment of cancer. There are encouraging data on their cytotoxic activity [14,15,16,17,18]. The above biological effects are all connected with the enzyme inhibitory properties of the α-hydroxyphosphonic derivatives [19]. The species under discussion may act as farnesyl protein transferase inhibitors [20], HIV protease [21] and viral cysteine protease inhibitors [22], inhibitors of CD45 tyrosine phosphatase [23] and undecaprenyl diphosphate phosphatase [24], as well as an influencer of the P5C reductase [25].

The practical method for the synthesis of α-hydroxyphosphonates is the Pudovik reaction involving the addition of dialkyl phosphites to the carbonyl carbon atom of an oxo compound that may be an aldehyde or ketone [26,27]. A wide variety of methods were described [28]. Special efforts were made to elaborate green synthetic methods, including catalyst-free and/or microwave-assisted accomplishments [29]. Solvent-free additions on the surface of solid catalysts (e.g., on aluminum oxide/KF) were also developed; however, these methods required solvents during the work-up comprising extraction, chromatography, and recrystallization [30]. The group of senior author of this paper described an indeed green procedure for the synthesis of α-hydroxy-benzylphosphonates, applying triethylamine as the catalyst and only a small quantity of acetone as the medium. The arylaldehyde-dialkyl phosphite adducts precipitated in a crystalline form from the reaction mixtures on cooling [31].

**Figure 1:**
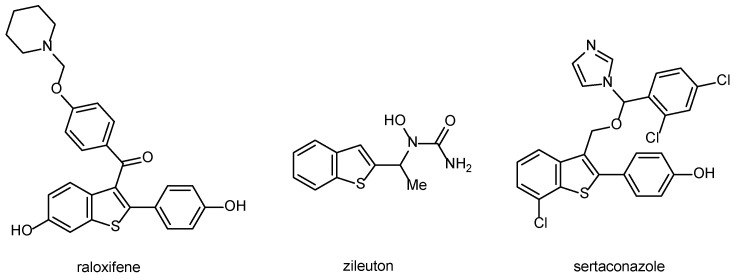
Structure of commercially available benzothiophene-containing drugs.

Compounds containing the benzo[*b*]thiophene ring play a significant role in medicinal chemistry, as certain derivatives exhibit various biological activities [32]. Several commercially available drugs contain this privileged scaffold, such as raloxifene, zileuton, and sertaconazole (Figure 1). Raloxifene is a selective estrogen receptor modulator to prevent and treat postmenopausal osteoporosis [33]. Zileuton is an orally active 5-lipoxygenase inhibitor used to treat asthma [34]. Sertaconazole displays activity against pathogenic fungi and is used for the treatment of superficial skin mycoses [35].

Recently, the anticancer activity of benzothiophene derivatives has been investigated by a number of research groups. For example, Eldehna and co-workers identified urea-tethered benzothiophenes as dual VEGFR-2/EGFR anticancer agents [36]. Benzothiophene acrylonitrile derivatives were tested against human cancer cell lines, and a few derivatives showed significant anticancer effects [37]. A 5-Hydroxybenzothiophene-2-carboxamides form a novel class of group-selective kinase inhibitors targeting Dyrk1A, Dyrk1B, and Clk1 [38].

In this article, we describe the synthesis of α-benzothiophenyl-α-hydroxymethylphosphonates, a brand new family of compounds alloying two scaffolds bearing potential activity: the hydroxyphosphonate skeleton and the benzothiophene heterocyclic ring.

## 2. Results and Discussion

### 2.1. Synthesis of the New α-Benzothiophenyl-α-hydroxy-ethylphosphonates

Our plan was to prepare a family of new α-hydroxyphosphonates (**2**) by the Pudovik reaction of a series of benzo[*b*]thiophene-2-carboxaldehydes (**1**) with diethyl phosphite. For the optimization, the addition of the phosphite to the 5-fluorobenzothiophene-2-carboxaldehyde (**1a**) served as the model reaction. It was found that carrying out the addition in dichloromethane at room temperature (26 °C), there was a need for 4 equivalents of triethylamine and 4 equivalents of diethyl phosphite. No complete conversions were attained by applying smaller amounts of the base and the P-reagent. Then, the reaction was extended to benzothiophene-carboxaldehydes (**1b**–**w**) containing methyl, ethyl, halogen, and trifluoromethyl substituents in different positions of the benzothiophene ring. The aldehyde—diethyl phosphite adducts (**2a**–**w**) were purified by flash column chromatography, and their structures were characterized by spectroscopic methods, such as ^31^P, ^13^C, and ^1^H NMR, as well as HRMS. In this way, 23 new α-hydroxyphosphonate derivatives (**2a**–**w**) were prepared in good to excellent yields. The preparative results are summarized in Table 1.

### 2.2. Spectroscopic Characterization

The structural elucidation of the synthesized α-hydroxyphosphonates was primarily supported by multinuclear NMR and IR spectroscopic data, which provided consistent and highly informative datasets across the compound library.

#### 2.2.1. ^13^C NMR Spectroscopy

The ^13^C spectra confirmed structural features through diagnostic ^13^C-^31^P and, where applicable, ^13^C-^19^F couplings. Universally, a strong 1-bond ^13^C-^31^P coupling (^1^*J*_C-P_ ≈ 165–168 Hz) was observed for the carbon atoms α to the phosphorus atom, typically appearing around *δ* 66–67 ppm. For the *O*-ethyl moieties, the ethyl CH_2_ and CH_3_ carbons exhibited 2- and 3-bond ^13^C-^31^P couplings (^2^*J*_C-P_ ≈ 7 Hz and ^3^*J*_C-P_ 5–6 Hz, respectively). In 3-methyl-substituted compounds, a weak 4-bond ^13^C-^31^P coupling (^4^*J*_C-P_ ≈ 1–1.5 Hz) was observable in the methyl carbon resonance. In contrast, non-methylated analogues displayed a 3-bond coupling on the aromatic C-3 carbon (^3^*J*_C-P_ = 8–10 Hz). The ^31^P coupling influence extended across the aromatic system, with 4- and even 5-bond couplings (^4,5^*J*_C-P_ = 1–3 Hz) detectable in certain ring carbons of the benzothiophene moiety.

#### 2.2.2. ^1^H NMR Spectroscopy

In the ^1^H NMR spectra, the aromatic protons of the benzothiophene ring appeared downfield, typically in the range of *δ* 8.0–6.8 ppm, with splitting patterns reflecting the substitution pattern of the aromatic ring. Fluorine substituents introduced additional splitting due to ^1^H-^19^F couplings, increasing spectral complexity. In most cases, a broad or multiplet resonance attributable to the hydroxyl proton was also observed, which lacked the corresponding ^1^H-^13^C HSQC cross-peak, consistent with assignment as a hydroxyl proton.

The methine proton adjacent to the hydroxyl group typically appeared as a doublet or doublet of doublets, depending on whether the coupling with the OH proton was resolved. Notably, a 2-bond ^1^H-^31^P coupling (^2^*J*_H-P_ ≈ 10–11 Hz) was consistently observed. For *O*-ethyl substituents, the methylene protons of the ethyl groups showed complex multiplets, while the terminal methyl protons appeared as triplets with the most upfield chemical shifts. In compounds with a 3-methyl substituent, a methyl doublet was seen around *δ* 2.5 ppm, showing a 5-bond ^1^H-^31^P coupling (^5^*J*_H-P_ ≈ 3 Hz). In non-methylated analogues, the aromatic proton in position 3 exhibited a 4-bond ^1^H-^31^P coupling (^4^*J*_H-P_ ≈ 3.5 Hz).

#### 2.2.3. ^31^P NMR Spectroscopy

A sharp singlet resonance was consistently detected around *δ* 19–20 ppm in the ^31^P NMR spectra, confirming the presence of the α-hydroxyphosphonate moiety.

**Figure 2:**
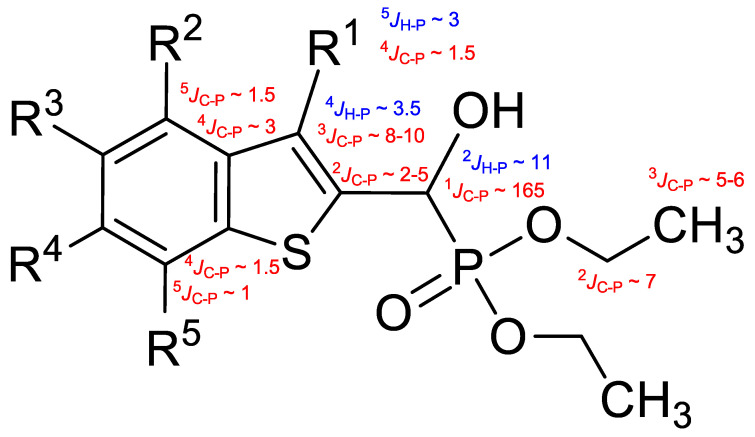
Typical ^1^H-^31^P (blue) and ^13^C-^31^P (red) coupling constants (in Hz) observed in the synthesized α-hydroxyphosphonates, shown on the general structural formula.

Typical values of the coupling constants of ^1^H or ^13^C nuclei with ^31^P nuclei are summarized in Figure 2, indicated on the general structural formula.

#### 2.2.4. IR Spectroscopy

The IR spectra exhibited characteristic absorption bands associated with phosphonate functional groups. Strong P=O stretching bands were seen in the range of 1220–1260 cm^−1^, while P-O-C stretching vibrations occurred around 1020–1060 cm^−1^. Additional bands observed between 700 and 800 cm^−1^ were assigned to P-C stretching modes. These vibrational features provide further verification of the core phosphonate structure.

### 2.3. Single Crystal X-Ray Analysis of Diethyl α-(7-Chlorobenzothiophenyl-)α-hydroxy-methylphosphonate (***2f***)

**Figure 3:**
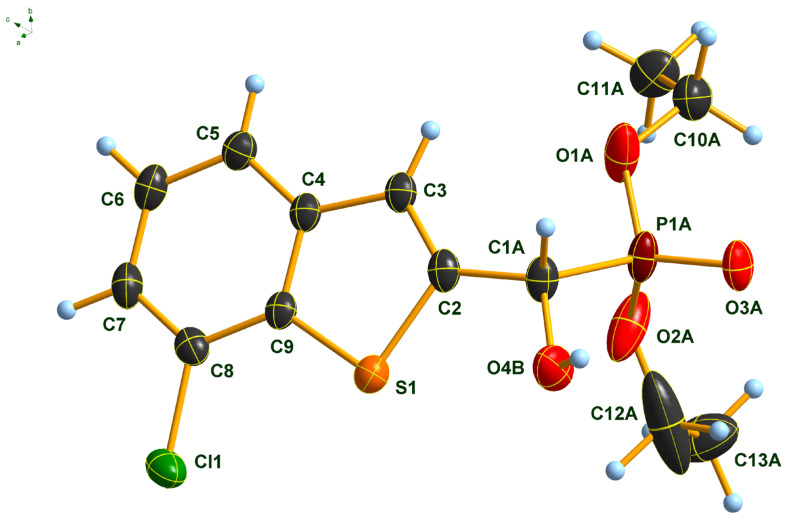
Molecular structure of 7-chlorobenzothiophenyl-α-hydroxy-methylphosphonate (**2f**) in the crystal. DIAMOND [39] representation; thermal ellipsoids are drawn at 50% probability level.

The molecular structure of α-(7-chlorobenzo[*b*]thiophen-2-yl)-α-hydroxy-methylphosphonate **2f** was determined by single-crystal X-ray diffraction that is shown in Figure 3. The P(O)(OEt)_2_ group of the molecule is disordered at least over two, most probably over more than two, positions. Only a split over two positions was considered. Further splitting did not result in an improvement of the structure model. Due to the strong disorder, the C-O and C-C distances of the OEt groups were fixed. Only one of the two positions of the P(O)(OEt)_2_ group (position A) is shown for clarity. In addition, the OH group is also disordered over two positions, most probably resulting from the two enantiomers occupying the same crystallographic position. Also, in this case, only one of the positions (position B) is shown for clarity. A figure showing the full disorder of the P(O)(OEt)_2_ and the OH groups can be found in the Supporting Information (Figure S1). Atom distances and bond angles are within the expected ranges (Tables S1–S3). Nevertheless, in addition to proving the structure of the compound, some interesting structural features can be observed. The heterocyclic part of the molecule is, as expected, planar, and the carbon atom C1A of the -CH(OH)- group is also lying in the plane of the heterocycle. This arrangement results in a flat molecule except for the -P(O)(OEt)_2_ unit, which is rotated out of the molecular plane with a dihedral angle P1A-C1A-C2-S1 of 117.8(2)°. This particular structural feature is anticipated to have an impact on the biological activity of benzothiophenyl α-hydroxyphosphonates.

### 2.4. Bioactivity Study of the α-Benzothiophenyl-α-hydroxy-methylphosphonates (***2a**–**w***)

A series of 23 different α-hydroxy-α-benzothiophenyl-methylphosphonates (**2a**–**w**) were screened for their effects on cell viability in four human tumor cell lines: U266 (multiple myeloma), EBC-1 (lung cancer), A2058 (melanoma), and HT-29 (colon cancer). Based on the cell viability results shown in Table 2, most compounds, regardless of the substituents in positions R^1^–R^5^, exhibited at least a mild viability-reducing effect across the tested cell lines with normalized cell viability values of 0.8 or lower.

Among the compounds with R^1^ = H, the appearance of a fluoro-substituent as R^3^-R^5^ in the benzothiophene ring (as in **2a**, **2b**, and **2c**) led to modest effects against the U266 myeloma cell line. In the case of R^3^ = Br (as in **2g**), only a moderate decrease in the cell viability of the A2058 melanoma and the HT-29 colon cell lines was observed, while the EBC-1 lung and U266 myeloma cell lines were clearly impacted. Furthermore, the appearance of a chloro- or iodo substituent as R^2^ or R^5^ had mostly favorable effects (as in **2d**, **2e**, **2f**, and **2h**), similarly to the presence of a trifluoromethyl group as R^2^ (**2j**). Similar results were observed following the treatment of the cell lines with compound **2w** (R^2^ = F, R^5^ = I).

Interestingly, when R^1^ = H was replaced with a methyl group (as in **2i**), long-term treatments had no significant effects on the cell viability of any cell line. Furthermore, the substitution of the benzene ring of species **2i** with one or two fluorine atoms (as in **2k**, **2l**, **2m**, **2n**, **2o**, and **2p**) did not markedly alter the antiproliferative effects as compared to homologue **2i**. Nevertheless, hydroxyphosphonates **2o** and **2p** exhibited some degree of effectiveness on the U266 cell line. This result aligns with earlier observations from treatments using derivatives **2a**, **2b**, or **2c**. The two chlorinated species, **2q** and **2r**, displayed profiles similar to those obtained for compounds **2d**, **2e**, and **2f**. Hydroxyphosphonates **2s**, **2t**, and **2u**, containing a trifluoromethyl group as the R^3^, R^4^, and R^5^ groups, respectively, showed varying effectiveness in the cell viability assays. The para-substituted hydroxyphosphonate (**2u**) exhibited the weakest activity, while the meta-substituted species (**2t**) showed the strongest activity on three cell lines (U266, EBC-1, and HT-29) out of the four. It should be pointed out that, on the U266 cell line, compound **2t** reduced cell viability to 0.09 ± 0.02. Changing R^1^ = Me to Et (as in **2v**) resulted in reduced activity, according to.

Structure–activity relationship studies have already revealed that the trifluoromethyl group can exert a strong electron-withdrawing effect and increase lipophilicity. This property can enhance the pharmacokinetic profile of drug candidates by improving bioavailability and metabolic stability. Additionally, the CF_3_ group may contribute to an increased binding affinity to target molecules, leading to higher efficacy—as also observed in our experimental data [40,41,42]. Additional input is required to further characterize the pharmacokinetic and pharmacodynamic profiles of the compounds.

Overall, U266 cells appeared to be the most sensitive to the hydroxyphosphonates applied in the tests. However, the effect of the vehicle control dimethyl sulfoxide (DMSO) cannot be overlooked, as it had a profound impact on the viability of U266 cells, but not on A2058, EBC-1, or HT-29 cells.

## 3. Materials and Materials

### 3.1. General

All chemicals and solvents were purchased from Sigma Aldrich (Saint Louis, MO, USA). All NMR spectra were recorded in CDCl_3_ or DMSO-*d*_6_ solution in standard 5 mm NMR tubes at 295 K on a Bruker Avance III HD 600 spectrometer (Bruker, Karlsruhe, Germany) (^1^H: 600.00 MHz, ^13^C: 150.89 MHz, ^31^P: 242.87 MHz). The ^31^P, ^1^H, and ^13^C chemical shifts (*δ*) are reported in parts per million (ppm) relative to the internal standard H_3_PO_4_, TMS, and the NMR solvent (CDCl_3_), respectively. Coupling constants (*J*) are given in hertz (Hz). The following abbreviations were used to designate multiplicities: s = singlet, d = doublet, dd = doublet of doublets, t = triplet, q = quartet, m = multiplet, br = broad. IR spectra were obtained on a Bruker Alpha FT-IR spectrometer (Bruker, Karlsruhe, Germany) in transmission mode in KBr pellets. High-resolution mass spectra (HRMS) were recorded on an Agilent 7250 Q-TOF mass spectrometer coupled to an Agilent 8890 gas chromatographic system (Waters Corporation Company, Milford, MA, USA). All melting points were determined on a Büchi B-540 capillary melting point apparatus (Büchi, Flawil, Swietzerland) and are uncorrected. Reactions were monitored by thin-layer chromatography (TLC) carried out on silica gel plates (60 F_254_) using UV light as a visualizing agent. Purifications by column chromatography were carried out using Merck 107736 silica gel 60 H (Merck, Darmstadt, Germany) with a CH_2_Cl_2_–MeOH solvent system. All reagents were purchased from commercial sources and were used without further purification. Benzo[*b*]thiophene-2-carboxaldehydes (**2**) were prepared according to literature methods [43,44,45].

### 3.2. General Procedure for the Synthesis of Diethyl [(1-Benzothiopen-2-yl)(hydroxy)methyl]phosphonates ***2***

The appropriate aldehyde (**1**, 1.0 mmol) was dissolved in dichloromethane (5 mL), then triethylamine (0.61 mL, 0.445 g, 4.4 mmol) and diethyl phosphite (0.57 mL, 0.608 g, 4.4 mmol) were added to this solution. The reaction mixture was stirred at room temperature for 24 h, diluted with dichloromethane (10 mL), and washed with water (2 × 10 mL). The organic layer was dried (Na_2_SO_4_), filtered, and the solvent removed under reduced pressure. The residue was purified by flash column chromatography on silica gel (CH_2_Cl_2_ → CH_2_Cl_2_/MeOH 9:1) to afford products **2**. Analytical samples were obtained by recrystallization from the solvents or solvent mixtures given below in parentheses after the melting point.

The following products were thus prepared:

#### 3.2.1. Diethyl [(5-Fluoro-1-benzothiophen-2-yl)(hydroxy)methyl]phosphonate (**2a**)


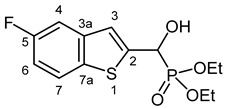


Yield: 240 mg (75%). White crystals. Mp 109–111 °C (hexane–EtOAc). IR (KBr): 3424, 3205, 1445, 1226, 1204, 1044, 870 cm^−1^. ^1^H NMR (CDCl_3_, 600 MHz): *δ* 7.72 (dd, *J*_1_ = 8.8 Hz, *J*_2_ = 4.8 Hz, 1H, H-7), 7.37 (dd, *J*_1_ = 9.4 Hz, *J*_2_ = 2.4 Hz, 1H, H-4), 7.34 (d, *J* = 3.4 Hz, 1H, H-3), 7.07 (td, *J*_t_ = 8.8 Hz, *J*_d_ = 2.5 Hz, 1H, H-6), 5.31 (d, *J* = 11.9 Hz, 1H, CH), 4.93 (br s, 1H, OH), 4.20–4.11 (m, 4H, 2 × CH_2_), 1.32 (t, *J* = 7.1 Hz, 3H, CH_3_), 1.28 (t, *J* = 7.1 Hz, 3H, CH_3_) ppm. ^13^C NMR (CDCl_3_, 150 MHz): *δ* 160.7 (d, *J* = 241.5 Hz, C-5), 143.3 (d, *J* = 1.6 Hz, C-2), 140.3 (dd, *J*_1_ = 9.5 Hz, *J*_2_ = 2.7 Hz, C-3a), 135.1 (t, *J* = 1.5 Hz, C-7a), 123.3 (d, *J* = 9.4 Hz, C-7), 121.9 (dd, *J*_1_ = 8.1 Hz, *J*_2_ = 4.3 Hz, C-3), 113.1 (dd, *J*_1_ = 25.3, *J*_2_ = 1.0 Hz, C-6), 109.0 (dd, *J*_1_ = 22.9 Hz, *J*_2_ = 0.7 Hz, C-4), 67.5 (d, *J* = 165.4 Hz, CH), 64.0 (d, *J* = 7.1 Hz, CH2), 63.5 (d, *J* = 7.4 Hz, CH_2_), 16.4 (d, *J* = 5.6 Hz, 2 × CH_3_) ppm. 31P NMR (CDCl_3_, 242 MHz): *δ* 19.0 ppm. HRMS: [M]^+^ Calcd. for C_13_H_16_FO_4_PS 318.0491; Found 318.0474.

#### 3.2.2. Diethyl [(6-Fluoro-1-benzothiophen-2-yl)(hydroxy)methyl]phosphonate (**2b**)


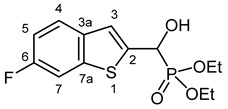


Yield: 277 mg (87%). White crystals. Mp 114–116 °C (EtOAc). IR (KBr): 3196, 2985, 1601, 1469, 1223, 1058, 1023, 568 cm^−1^. ^1^H NMR (CDCl_3_, 600 MHz): *δ* 7.64 (dd, *J*_1_ = 8.7 Hz, *J*_2_ = 5.1 Hz, 1H, H-4), 7.48 (dd, *J*_1_ = 8.7 Hz, *J*_2_ = 2.0 Hz, 1H, H-7), 7.33 (d, *J* = 3.4 Hz, 1H, H-3), 7.08 (td, *J*_t_ = 8.9 Hz, *J*_d_ = 2.3 Hz, 1H, H-5), 5.29 (d, *J* = 11.6 Hz, 1H, CH), 5.22 (br s, 1H, OH), 4.20–4.10 (m, 4H, 2 × CH_2_), 1.31 (t, *J* = 7.1 Hz, 3H, CH_3_), 1.28 (t, *J* = 7.1 Hz, 3H, CH_3_) ppm. ^13^C NMR (CDCl_3_, 150 MHz): *δ* 160.4 (dd, *J*_1_ = 244.3 Hz, *J*_2_ = 1.2 Hz, C-6), 140.7 (dd, *J*_1_ = 10.3 Hz, *J*_2_ = 1.5 Hz, C-7a), 140.3 (dd, *J*_1_ = 3.7 Hz, *J*_2_ = 1.4 Hz, C-2), 135.8 (m, C-3a), 124.6 (dd, *J*_1_ = 9.1 Hz, *J*_2_ = 0.8 Hz, C-4), 121.8 (d, *J* = 8.3 Hz, C-3), 113.3 (d, *J* = 24.2 Hz, C-5), 108.3 (d, *J* = 25.4 Hz, C-7), 67.3 (d, *J* = 166.2 Hz, CH), 63.9 (d, *J* = 7.1 Hz, CH_2_), 63.5 (d, *J* = 7.3 Hz, CH_2_), 16.39 (d, *J* = 5.5 Hz, CH_3_), 16.38 (d, *J* = 5.8 Hz, CH_3_) ppm. ^31^P NMR (CDCl_3_, 242 MHz): *δ* 19.2 ppm. HRMS: [M]^+^ Calcd. for C_13_H_16_FO_4_PS 318.0491; Found 318.0486.

#### 3.2.3. Diethyl [(7-Fluoro-1-benzothiophen-2-yl)(hydroxy)methyl]phosphonate (**2c**)


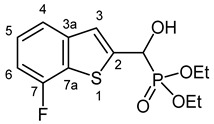


Yield: 299 mg (94%). Off-white crystals. Mp 125–127 °C (EtOAc). IR (KBr): 3405, 3230, 1470, 1228, 1058, 1016, 978, 784 cm^−1^. ^1^H NMR (CDCl_3_, 600 MHz): *δ* 7.52–7.50 (m, 1H, H-4), 7.42–7.40 (m, 1H, H-3), 7.31–7.27 (m, 1H, H-5), 7.03–6.99 (m, 1H, H-6), 5.33 (d, *J* = 11.9 Hz, 1H, CH), 5.03 (br s, 1H, OH), 4.22–4.13 (m, 4H, 2 × CH_2_), 1.32 (t, *J* = 7.1 Hz, 3H, CH_3_), 1.29 (t, *J* = 7.1 Hz, 3H, CH_3_) ppm. ^13^C NMR (CDCl_3_, 150 MHz): *δ* 157.5 (dd, *J*_1_ = 246.8 Hz, *J*_2_ = 1.0 Hz, C-7), 142.7 (dd, *J*_1_ = 4.6 Hz, *J*_2_ = 2.6 Hz, C-3a), 142.1 (t, *J* = 1.5 Hz, C-2), 126.6 (dd, *J*_1_ = 18.1 Hz, *J*_2_ = 1.5 Hz, C-7a), 125.6 (d, *J* = 6.8 Hz, C-5), 122.4 (dd, *J*_1_ = 8.0 Hz, *J*_2_ = 2.2 Hz, C-3), 119.4 (dd, *J*_1_ = 3.3 Hz, *J*_2_ = 0.8 Hz, C-4), 109.3 (dd, *J*_1_ = 18.5 Hz, *J*_2_ = 0.8 Hz, C-6), 67.4 (d, *J* = 165.2 Hz, CH), 64.0 (d, *J* = 7.1 Hz, CH_2_), 63.4 (d, *J* = 7.4 Hz, CH_2_), 16.42 (d, *J* = 5.4 Hz, CH_3_), 16.40 (d, *J* = 5.7 Hz, CH_3_) ppm. ^31^P NMR (CDCl_3_, 242 MHz): *δ* 18.9 ppm. HRMS: [M]^+^ Calcd. for C_13_H_16_FO_4_PS 318.0491; Found 318.0483.

#### 3.2.4. Diethyl [(4-Chloro-1-benzothiophen-2-yl)(hydroxy)methyl]phosphonate (**2d**)


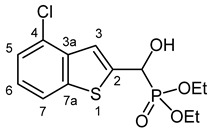


Yield: 241 mg (72%). White crystals. Mp 90–92 °C (hexane–EtOAc). IR (KBr): 3239, 1414, 1253, 1203, 1036, 761, 542 cm^−1^. ^1^H NMR (DMSO-*d*_6_, 600 MHz): *δ* 7.96 (d, *J* = 8.0 Hz, 1H, H-7), 7.47 (d, *J* = 3.6 Hz, 1H, H-3), 7.46–7.45 (m, 1H, H-5), 7.34 (t, *J* = 7.9 Hz, 1H, H-6), 6.91 (dd, *J*_1_ = 14.5 Hz, *J*_2_ = 6.0 Hz, 1H, OH), 5.45 (dd, *J*_1_ = 14.5 Hz, *J*_2_ = 5.4 Hz, 1H, CH), 4.10–4.06 (m, 2H, CH_2_), 4.06–4.01 (m, 2H, CH_2_), 1.22 (t, *J* = 7.1 Hz, 3H, CH_3_), 1.21 (t, *J* = 7.1 Hz, 3H, CH_3_) ppm. ^13^C NMR (DMSO-*d*_6_, 150 MHz): *δ* 145.9 (C-7a), 140.5 (d, *J* = 1.4 Hz, C-2), 137.1 (d, *J* = 2.5 Hz, C-3a), 127.0 (d, *J* = 1.1 Hz, C-4), 125.4 (C-6), 124.4 (C-5), 121.8 (C-7), 119.2 (d, *J* = 8.4 Hz, C-3), 65.7 (d, *J* = 168.1 Hz, CH), 63.0 (d, *J* = 7.0 Hz, CH_2_), 62.6 (d, *J* = 6.8 Hz, CH_2_), 16.54 (d, *J* = 5.6 Hz, CH_3_), 16.51 (d, *J* = 5.5 Hz, CH_3_) ppm. ^31^P NMR (DMSO-*d*_6_, 242 MHz): *δ* 19.4 ppm. HRMS: [M]^+^ Calcd. for C_13_H_16_ClO_4_PS 334.0195; Found 334.0186.

#### 3.2.5. Diethyl [(5-Chloro-1-benzothiophen-2-yl)(hydroxy)methyl]phosphonate (**2e**)


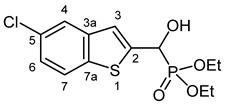


Yield: 207 mg (62%). Off-white crystals. Mp 104–105 °C (hexane–EtOAc). IR (KBr): 3425, 3169, 1440, 1224, 1067, 1052, 1027, 545 cm^−1^. ^1^H NMR (CDCl_3_, 600 MHz): *δ* 7.70 (d, *J* = 8.6 Hz, 1H, H-7), 7.67 (br s, 1H, H-4), 7.30 (d, *J* = 3.2 Hz, 1H, H-3), 7.28–7.25 (m, 1 H, H-6), 5.32 (br s, 1H, OH), 5.31 (d, *J* = 11.0 Hz, 1H, CH), 4.20–4.15 (m, 2H, CH_2_), 4.15–4.10 (m, 2H, CH_2_), 1.31 (t, *J* = 7.0 Hz, 3H, CH_3_), 1.27 (t, *J* = 7.1 Hz, 3H, CH_3_) ppm. ^13^C NMR (CDCl_3_, 150 MHz): *δ* 143.0 (d, *J* = 1.5 Hz, C-2), 140.5 (d, *J* = 2.6 Hz, C-3a), 137.8 (d, *J* = 1.6 Hz, C-7a), 130.4 (C-5), 124.7 (C-6), 123.2 (C-7), 123.0 (C-4), 121.4 (d, *J* = 7.9 Hz, C-3), 67.4 (d, *J* = 165.8 Hz, CH), 64.0 (d, *J* = 7.0 Hz, CH_2_), 63.5 (d, *J* = 7.4 Hz, CH_2_), 16.39 (d, *J* = 5.4 Hz, CH_3_), 16.38 (d, *J* = 5.7 Hz, CH_3_) ppm. ^31^P NMR (CDCl_3_, 242 MHz): *δ* 19.0 ppm. HRMS: [M]^+^ Calcd. for C_13_H_16_ClO_4_PS 334.0202; Found 334.0194.

#### 3.2.6. Diethyl [(7-Chloro-1-benzothiophen-2-yl)(hydroxy)methyl]phosphonate (**2f**)


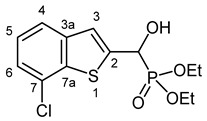


Yield: 287 mg (86%). White crystals. Mp 119–120 °C (EtOAc). IR (KBr): 3324, 2980, 1453, 1198, 1045, 974, 766 cm^−1^. ^1^H NMR (CDCl_3_, 600 MHz): *δ* 7.62 (d, *J* = 7.3 Hz, 1H, H-4), 7.41 (d, *J* = 3.4 Hz, 1H, H-3), 7.32–7.29 (m, 1H, H-6), 7.29–7.26 (m, 1H, H-5), 5.34 (d, *J* = 11.9 Hz, 1H, CH), 5.23 (br s, 1H, OH), 4.21–4.13 (m, 4H, 2 × CH_2_), 1.32 (t, *J* = 7.1 Hz, 3H, CH_3_), 1.29 (t, *J* = 7.1 Hz, 3H, CH_3_) ppm. ^13^C NMR (CDCl_3_, 150 MHz): *δ* 142.1 (d, *J* = 1.4 Hz, C-2), 140.7 (d, *J* = 2.6 Hz, C-3a), 138.9 (d, *J* = 1.6 Hz, C-7a), 127.6 (C-7), 125.4 (C-5), 123.8 (C-6), 122.7 (d, *J* = 8.0 Hz, C-3), 122.0 (d, *J* = 0.8 Hz, C-4), 67.4 (d, *J* = 165.5 Hz, CH), 64.0 (d, *J* = 7.0 Hz, CH_2_), 63.6 (d, *J* = 7.3 Hz, CH_2_), 16.43 (d, *J* = 5.4 Hz, CH_3_), 16.40 (d, *J* = 5.6 Hz, CH_3_) ppm. ^31^P NMR (CDCl_3_, 242 MHz): *δ* 18.9 ppm. HRMS: [M]^+^ Calcd. for C_13_H_16_ClO_4_PS 334.0195; Found 334.0190.

#### 3.2.7. Diethyl [(5-Bromo-1-benzothiophen-2-yl)(hydroxy)methyl]phosphonate (**2g**)


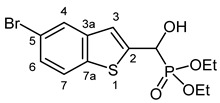


Yield: 277 mg (73%). White crystals. Mp 96–100 °C (EtOAc). IR (KBr): 3166, 2982, 1580, 1437, 1224, 1122, 1061, 1026, 980 cm^−1^. ^1^H NMR (CDCl_3_, 600 MHz): *δ* 7.85 (d, *J* = 1.7 Hz, 1H, H-4), 7.66 (d, *J* = 8.5 Hz, 1H, H-7), 7.41 (dd, *J*_1_ = 8.6 Hz, *J*_2_ = 1.4 Hz, 1H, H-6), 7.31 (d, *J* = 3.4 Hz, 1H, H-3), 5.31 (dd, *J*_1_ = 11.8 Hz, *J*_2_ = 4.7 Hz, 1H, CH), 4.85 (br s, 1H, OH), 4.20–4.10 (m, 4H, 2 × CH_2_), 1.32 (t, *J* = 7.1 Hz, 3H, CH_3_), 1.28 (t, *J* = 7.1 Hz, 3H, CH_3_) ppm. ^13^C NMR (CDCl_3_, 150 MHz): *δ* 142.7 (d, *J* = 1.7 Hz, C-2), 140.9 (d, *J* = 2.6 Hz, C-3a), 138.3 (d, *J* = 1.5 Hz, C-7a), 127.3 (d, *J* = 0.9 Hz, C-6), 126.2 (d, *J* = 0.9 Hz, C-4), 123.6 (C-7), 121.4 (d, *J* = 8.1 Hz, C-3), 118.2 (C-5), 67.5 (d, *J* = 165.1 Hz, CH), 64.0 (d, *J* = 7.0 Hz, CH_2_), 63.6 (d, *J* = 7.3 Hz, CH_2_), 16.42 (d, *J* = 5.8 Hz, CH_3_), 16.41 (d, *J* = 5.8 Hz, CH_3_) ppm. ^31^P NMR (CDCl_3_, 242 MHz): *δ* 18.9 ppm. HRMS: [M]^+^ Calcd. for C_13_H_16_BrO_4_PS 377.9690; Found 377.9687.

#### 3.2.8. Diethyl [Hydroxy(4-iodo-1-benzothiophen-2-yl]methyl]phosphonate (**2h**)


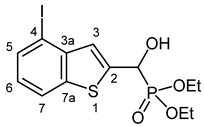


Yield: 283 mg (66%). White crystals. Mp 94–95 °C (hexane–EtOAc). IR (KBr): 3188, 2979, 1522, 1441, 1404, 1255, 1238, 1196, 1022, 976 cm^−1^. ^1^H NMR (CDCl_3_, 600 MHz): *δ* 7.77 (d, *J* = 8.0 Hz, 1H, H-7), 7.75 (dd, *J*_1_ = 7.6 Hz, *J*_2_ = 0.7 Hz, 1H, H-5), 7.46 (d, *J* = 3.5 Hz, 1H, H-3), 7.02 (t, *J* = 8.0 Hz, 1H, H-6), 5.35 (d, *J* = 11.7 Hz, 1H, CH), 4.24–4.14 (m, 5H, 2 × CH_2_, OH), 1.35 (t, *J* = 7.1 Hz, 3H, CH_3_), 1.32 (t, *J* = 7.1 Hz, 3H, CH_3_) ppm. ^13^C NMR (CDCl_3_, 150 MHz): *δ* 142.3 (d, *J* = 2.6 Hz, C-3a), 141.2 (d, *J* = 2.5 Hz, C-2), 138.9 (d, *J* = 1.5 Hz, C-7a), 134.3 (C-5), 126.3 (d, *J* = 8.3 Hz, C-3), 125.5 (d, *J* = 0.8 Hz, C-6), 122.3 (C-7), 90.1 (d, *J* = 1.5 Hz, C-4), 67.7 (d, *J* = 164.8 Hz, CH), 64.0 (d, *J* = 7.0 Hz, CH_2_), 63.6 (d, *J* = 7.3 Hz, CH_2_), 16.5 (d, *J* = 5.4 Hz, CH_3_), 16.4 (d, *J* = 5.8 Hz, CH_3_) ppm. ^31^P NMR (CDCl_3_, 242 MHz): *δ* 18.7 ppm. HRMS: [M]^+^ Calcd. for C_13_H_16_IO_4_PS 425.9552; Found 425.9553.

#### 3.2.9. Diethyl [(Hydroxy(3-methyl-1-benzothiophen-2-yl)methyl]phosphonate (**2i**)


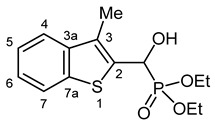


Yield: 193 mg (61%). White crystals. Mp 128–130 °C (hexane–EtOAc). IR (KBr): 3216, 2974, 1438, 1230, 1196, 1051, 1019, 964, 751 cm^−1^. ^1^H NMR (CDCl_3_, 600 MHz): *δ* 7.81 (d, *J* = 7.9 Hz, 1H, H-7), 7.68 (d, *J* = 7.9 Hz, 1H, H-4), 7.38 (t, *J* = 7.3 Hz, 1H, H-5 or H-6), 7.33 (t, *J* = 7.6 Hz, 1H, H-5 or H-6), 5.52 (dd, *J*_1_ = 10.7 Hz, *J*_2_ = 3.1 Hz, 1H, CH), 2.40 (d, *J* = 3.0 Hz, 3H, CH_3_), 1.30 (t, *J* = 6.6 Hz, 3H, CH_2_CH_3_), 1.28 (t, *J* = 7.2 Hz, 3H, CH_2_CH_3_) ppm. ^13^C NMR (CDCl_3_, 150 MHz): *δ* 140.1 (d, *J* = 2.9 Hz, C-3a), 139.2 (d, *J* = 1.6 Hz, C-7a), 134.2 (d, *J* = 5.2 Hz, C-2), 129.7 (d, *J* = 10.1 Hz, C-3), 124.4 (d, *J* = 1.1 Hz, C-5 or C-6), 123.9 (C-5 or C-6), 122.4 (d, *J* = 1.0 Hz, C-4), 121.8 (d, *J* = 1.3 Hz. C-7), 66.4 (d, *J* = 167.6 Hz, CH), 63.9 (d, *J* = 6.9 Hz, CH_2_), 63.2 (d, *J* = 7.3 Hz, CH_2_), 16.4 (t, *J* = 5.9 Hz, 2 × OCH_2_CH_3_), 12.3 (d, *J* = 1.3 Hz, CH_3_) ppm. ^31^P NMR (CDCl_3_, 242 MHz): *δ* 20.0 ppm. HRMS: [M]^+^ Calcd. for C_14_H_19_O_4_PS 314.0742; Found 314.0740.

#### 3.2.10. Diethyl {Hydroxy [4-(trifluoromethyl)-1-benzothiophen-2-yl]methyl}phosphonate (**2J**)


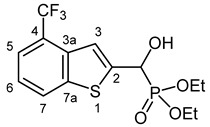


Yield: 289 mg (79%). White crystals. Mp 86–89 °C (hexane–EtOAc). IR (KBr): 3444, 3240, 1426, 1328, 1221, 1120, 1057, 1031 cm^−1^. ^1^H NMR (CDCl_3_, 600 MHz): *δ* 7.99–7.96 (m, 1H, H-7), 7.65–7.63 (m, 1H, H-5), 7.58 (br s, 1H, H-3), 7.39–7.35 (m, 1H, H-6), 5.43 (d, *J* = 11.5 Hz, 1H, CH), 4.91 (br s, 1H, OH), 4.22–4.12 (m, 4H, 2 × CH_2_), 1.32 (t, *J* = 7.1 Hz, 3H, CH_3_), 1.29 (t, *J* = 7.1 Hz, 3H, CH_3_) ppm. ^13^C NMR (CDCl_3_, 150 MHz): *δ* 143.9 (C-2), 141.2 (d, *J* = 1.6 Hz, C-7a), 135.3 (C-3a), 126.0 (C-7), 124.42 (q, *J* = 32.2 Hz, C-4), 124.35 (q, *J* = 273.1 Hz, CF_3_), 123.2 (C-6), 121.9 (q, *J* = 5.0 Hz, C-5), 119.9 (dq, *J*_d_ = 8.1 Hz, *J*_q_ = 1.6 Hz, C-3), 67.5 (d, *J* = 165.6 Hz, CH), 64.4 (d, *J* = 7.0 Hz, CH_2_), 63.6 (d, *J* = 7.6 Hz, CH_2_), 16.3 (d, *J* = 5.6 Hz, CH_3_), 16.2 (d, *J* = 6.0 Hz, CH_3_) ppm. ^31^P NMR (CDCl_3_, 242 MHz): *δ* 18.6 ppm. HRMS: [M]^+^ Calcd. for C_14_H_16_F_3_O_4_PS 368.0459; Found 368.0456.

#### 3.2.11. Diethyl [(4-Fluoro-3-methyl-1-benzothiophen-2-yl)(hydroxy)methyl]phosphonate (**2k**)


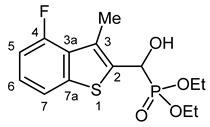


Yield: 236 mg (71%). White crystals. Mp 105–107 °C (EtOAc). IR (KBr): 3216, 2988, 1467, 1230, 1058, 1024, 973 cm^−1^. ^1^H NMR (CDCl_3_, 600 MHz): *δ* 7.54 (d, *J* = 7.9 Hz, 1H, H-7), 7.24–7.20 (m, 1H, H-6), 6.97 (dd, *J*_1_ = 11.8 Hz, *J*_2_ = 7.9 Hz, 1H, H-5), 5.50 (d, *J* = 10.4 Hz, 1H, CH), 4.22–4.15 (m, 2H, CH_2_), 4.15–4.11 (m, 1H, CH_2_), 4.11–4.07 (m, 1H, CH_2_), 2.53 (dd, *J*_1_ = 3.0 Hz, *J*_2_ = 2.5 Hz, 1H, CH_3_), 1.30 (t, *J* = 7.1 Hz, 6H, 2 × CH_2_CH_3_) ppm. ^13^C NMR (CDCl_3_, 150 MHz): *δ* 158.8 (dd, *J*_1_ = 251.7 Hz, *J*_2_ = 1.5 Hz, C-4), 141.7 (dd, *J*_1_ = 6.6 Hz, *J*_2_ = 1.6 Hz, C-7a), 134.8 (dd, *J*_1_ = 4.9 Hz, *J*_2_ = 1.1 Hz, C-2), 128.7 (dd, *J*_1_ = 15.1 Hz, *J*_2_ = 3.0 Hz, C-3a), 128.1 (dd, *J*_1_ = 10.3 Hz, *J*_2_ = 4.5 Hz, C-3), 125.0 (dd, *J*_1_ = 7.7 Hz, *J*_2_ = 1.0 Hz, C-6), 118.3 (dd, *J*_1_ = 3.9 Hz, *J*_2_ = 1.1 Hz, C-7), 109.8 (d, *J* = 20.6 Hz, C-5), 65.9 (d, *J* = 168.2 Hz, CH), 64.1 (d, *J* = 7.0 Hz, CH_2_), 63.2 (d, *J* = 7.5 Hz, CH_2_), 16.5 (d, *J* = 5.5 Hz, CH_2_CH_3_), 16.4 (d, *J* = 5.9 Hz, CH_2_CH_3_), 14.5 (dd, *J*_1_ = 6.6 Hz, *J*_2_ = 1.1 Hz, CH_3_) ppm. ^31^P NMR (CDCl_3_, 242 MHz): *δ* 19.9 ppm. HRMS: [M]^+^ Calcd. for C_14_H_18_FO_4_PS 332.0648; Found 332.0649.

#### 3.2.12. Diethyl [(5-Fluoro-3-methyl-1-benzothiophen-2-yl)(hydroxy)methyl]phosphonate (**2l**)


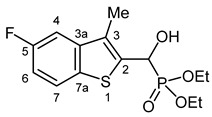


Yield: 264 mg (79%). White crystals. Mp 125–126 °C (EtOAc). IR (KBr): 3211, 1605, 1445, 1230, 1194, 1051, 1019, 801 cm^−1^. ^1^H NMR (CDCl_3_, 600 MHz): *δ* 7.71 (dd, *J*_1_ = 8.7 Hz, *J*_2_ = 4.9 Hz, 1H, H-7), 7.31 (dd, *J*_1_ = 9.7 Hz, *J*_2_ = 2.4 Hz, 1H, H-4), 7.08 (td, *J*_t_ = 8.8 Hz, *J*_d_ = 2.4 Hz, 1H, H-6), 5.51 (dd, *J*_1_ = 10.6 Hz, *J*_2_ = 4.3 Hz, 1H, CH), 4.90 (br s, 1H, OH), 4.19–4.14 (m, 2H, CH_2_), 4.14–4.06 (m, 2H, CH_2_), 2.33 (d, *J* = 2.9 Hz, 3H, CH_3_), 1.29 (t, *J* = 7.2 Hz, 3H, CH_2_CH_3_), 1.28 (t, *J* = 7.1 Hz, 3H, CH_2_CH_3_) ppm. ^13^C NMR (CDCl_3_, 150 MHz): *δ* 160.7 (d, *J* = 241.3 Hz, C-5), 141.3 (dd, *J*_1_ = 8.7 Hz, *J*_2_ = 2.9 Hz, C-3a), 137.3 (d, *J* = 5.0 Hz, C-2), 134.4 (C-7a), 129.1 (dd, *J*_1_ = 10.1 Hz, *J*_2_ = 4.3 Hz, C-3), 123.5 (d, *J* = 9.2 Hz, C-7), 113.1 (d, *J* = 25.1 Hz, C-6), 107.4 (d, *J* = 22.9 Hz, C-4), 66.3 (d, *J* = 167.9 Hz, CH), 64.0 (d, *J* = 7.0 Hz, CH_2_), 63.2 (d, *J* = 7.5 Hz, CH_2_), 16.4 (d, *J* = 5.5 Hz, CH_2_CH_3_), 16.3 (d, *J* = 5.8 Hz, CH_2_CH_3_), 12.3 (d, *J* = 1.1 Hz, CH_3_) ppm. ^31^P NMR (CDCl_3_, 242 MHz): *δ* 19.9 ppm. HRMS: [M]^+^ Calcd. for C_14_H_18_FO_4_PS 332.0648; Found 332.0637.

#### 3.2.13. Diethyl [(6-Fluoro-3-methyl-1-benzothiophen-2-yl)(hydroxy)methyl]phosphonate (**2m**)


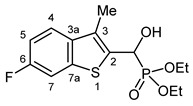


Yield: 169 mg (51%). White crystals. Mp 139–140 °C (EtOAc). IR (KBr): 3212, 2925, 1602, 1469, 1230, 1194, 1053, 1021, 965, 854 cm^−1^. ^1^H NMR (CDCl_3_, 600 MHz): *δ* 7.60 (dd, *J*_1_ = 8.7 Hz, *J*_2_ = 5.1 Hz, 1H, H-4), 7.49 (dd, *J*_1_ = 8.7, *J*_2_ = 1.9 Hz, 1H, H-7), 7.12 (td, *J*_t_ = 8.7 Hz, *J*_d_ = 1.9 Hz, 1H, H-5), 5.47 (dd, *J*_1_ = 10.5 Hz, *J*_2_ = 3.5 Hz, 1H, CH), 4.23–4.21 (m, 1H, OH), 4.21–4.14 (m, 2H, CH_2_), 4.14–4.07 (m, 2H, CH_2_), 2.38 (d, *J* = 3.0 Hz, 3H, CH_3_), 1.30 (t, *J* = 7.1 Hz, 6H, 2 × CH_2_CH_3_) ppm. ^13^C NMR (CDCl_3_, 150 MHz): *δ* 160.6 (dd, *J*_1_ = 244.3 Hz, *J*_2_ = 1.3 Hz, C-6), 140.1 (dd, *J*_1_ = 10.2 Hz, *J*_2_ = 1.6 Hz, C-7a), 136.6 (dd, *J*_1_ = 3.1 Hz, *J*_2_ = 1.3 Hz, C-3a), 133.8 (dd, *J*_1_ = 5.2 Hz, *J*_2_ = 3.7 Hz, C-2), 129.1 (d, *J* = 10.1 Hz, C-3), 122.8 (dd, *J*_1_ = 9.2 Hz, *J*_2_ = 1.3 Hz, C-4), 112.8 (d, *J* = 24.1 Hz, C-5), 108.5 (dd, *J*_1_ = 25.2 Hz, *J*_2_ = 1.0 Hz, C-7), 66.3 (d, *J* = 167.9 Hz, CH), 63.9 (d, *J* = 7.0 Hz, CH_2_), 63.2 (d, *J* = 7.4 Hz, CH_2_), 16.5 (d, *J* = 5.6 Hz, CH_2_CH_3_), 16.4 (d, *J* = 5.9 Hz, CH_2_CH_3_), 12.4 (d, *J* = 1.3 Hz, CH_3_) ppm. ^31^P NMR (CDCl_3_, 242 MHz): *δ* 19.9 ppm. HRMS: [M]^+^ Calcd. for C_14_H_18_FO_4_PS 332.0648; Found 332.0647.

#### 3.2.14. Diethyl [(7-Fluoro-3-methyl-1-benzothiophen-2-yl)(hydroxy)methyl]phosphonate (**2n**)


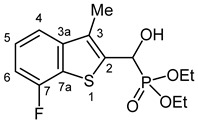


Yield: 264 mg (79%). White crystals. Mp 134–136 °C (EtOAc). IR (KBr): 3216, 1551, 1473, 1229, 1196, 1058, 1025, 779, 713 cm^−1^. ^1^H NMR (CDCl_3_, 600 MHz): *δ* 7.45 (d, *J* = 8.0 Hz, H-4), 7.34–7.30 (m, 1H, H-5), 7.03 (t, *J* = 8.8 Hz, H-6), 5.54 (dd, *J*_1_ = 10.6 Hz, *J*_2_ = 4.4 Hz, CH), 5.08 (br s, 1H, OH), 4.23–4.20 (m, 2H, CH_2_), 4.16–4.12 (m, 1H, CH_2_), 4.10–4.02 (m, 1H, CH_2_), 2.38 (d, *J* = 2.38 Hz, CH_3_), 1.31 (t, *J* = 7.1 Hz, CH_2_CH_3_), 1.28 (t, *J* = 7.1 Hz, CH_2_CH_3_) ppm. ^13^C NMR (CDCl_3_, 150 MHz): *δ* 157.6 (dd, *J*_1_ = 246.7 Hz, *J*_2_ = 1.3 Hz, C-7), 143.6 (dd, *J*_1_ = 4.5 Hz, *J*_2_ = 3.1 Hz, C-3a), 131.6 (dd, *J*_1_ = 5.0 Hz, *J*_2_ = 1.3 Hz, C-2), 129.7 (dd, *J*_1_ = 10.0 Hz, *J*_2_ = 2.1 Hz, C-3), 125.8 (dd, *J*_1_ = 18.3 Hz, *J*_2_ = 1.5 Hz, C-7a), 125.2 (d, *J* = 6.8 Hz, C-5), 117.5 (dd, *J*_1_ = 3.0 Hz, *J*_2_ = 1.7 Hz, C-4), 109.5 (d, *J* = 18.6 Hz, C-6), 66.2 (d, *J* = 167.9 Hz, CH), 64.1 (d, *J* = 7.0 Hz, CH_2_), 63.2 (d, *J* = 7.5 Hz, CH_2_), 16.5 (d, *J* = 5.5 Hz, CH_2_CH_3_), 16.4 (d, *J* = 5.8 Hz, CH_2_CH_3_), 12.6 (CH_3_) ppm. ^31^P NMR (CDCl_3_, 242 MHz): *δ* 19.8 ppm. HRMS: [M]^+^ Calcd. for C_14_H_18_FO_4_PS 332.0648; Found 332.0638.

#### 3.2.15. Diethyl [(5,7-Difluoro-3-methyl-1-benzothiophen-2-yl)(hydroxy)methyl]phosphonate (**2o**)


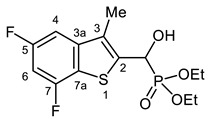


Yield: 262 mg (75%). White crystals. Mp 150–153 °C (EtOAc). IR (KBr): 3205, 2977, 1620, 1578, 1423, 1228, 1193, 1136, 1059, 1019 cm^−1^. ^1^H NMR (CDCl_3_, 600 MHz): *δ* 7.15 (dd, *J*_1_ = 9.1 Hz, *J*_2_ = 2.0 Hz, 1H, H-4), 6.85 (td, *J*_t_ = 9.3 Hz, *J*_d_ = 1.5 Hz, 1H, H-6), 5.51 (dd, *J*_1_ = 10.7 Hz, *J*_2_ = 4.0 Hz, 1H, CH), 5.27–5.21 (m, 1H, OH), 4.25–4.20 (m, 2H, CH_2_), 4.19–4.11 (m, 1H, CH_2_), 4.11–4.05 (m, 1H, CH_2_), 2.34 (d, *J* = 3.2 Hz, 3H, CH_3_), 1.32 (t, *J* = 7.1 Hz, 3H, CH_2_CH_3_), 1.29 (t, *J* = 7.1 Hz, 3H, CH_2_CH_3_) ppm. ^13^C NMR (CDCl_3_, 150 MHz): *δ* 160.7 (dd, *J*_1_ = 243.5 Hz, *J*_2_ = 10.5 Hz, C-5), 157.1 (ddd, *J*_1_ = 248.7, *J*_2_ = 13.6 Hz, *J*_3_ = 1.3 Hz, C-7), 143.0 (m, C-3a), 138.4 (dd, *J*_1_ = 4.9 Hz, *J*_2_ = 1.2 Hz, C-2), 129.3 (ddd, *J*_1_ = 9.8 Hz, *J*_2_ = 4.7 Hz, *J*_3_ = 2.3 Hz, C-3), 121.6 (d, *J* = 18.5 Hz, C-7a), 103.4 (dd, *J*_1_ = 23.0, *J*_2_ = 2.5 Hz, C-4), 99.8 (dd, *J*_1_ = 28.9 Hz, *J*_2_ = 22.6 Hz, C-6), 66.2 (d, *J* = 167.8 Hz, CH), 64.3 (d, *J* = 7.0 Hz, CH_2_), 63.3 (d, *J* = 7.5 Hz, CH_2_), 16.5 (d, *J* = 5.4 Hz, CH_2_CH_3_), 16.4 (d, *J* = 5.9 Hz, CH_2_CH_3_), 12.5 (CH_3_) ppm. ^31^P NMR (CDCl_3_, 242 MHz): *δ* 19.5 ppm. HRMS: [M]^+^ Calcd. for C_14_H_17_F_2_O_4_PS 350.0553; Found 350.0553.

#### 3.2.16. Diethyl [(6,7-Difluoro-3-methyl-1-benzothiophen-2-yl)(hydroxy)methyl]phosphonate (**2p**)


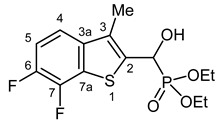


Yield: 197 mg (56%). White crystals. Mp 176–179 °C (EtOAc). IR (KBr): 3226, 2984, 1623, 1497, 1440, 1268, 1205, 1018 cm^−1^. ^1^H NMR (CDCl_3_, 600 MHz): *δ* 7.36 (dd, *J*_1_ = 8.7 Hz, *J*_2_ = 3.9 Hz, H-4), 7.22–7.18 (m, 1H, H-5), 5.49 (dd, *J*_1_ = 10.4 Hz, *J*_2_ = 3.7 Hz, 1H, CH), 4.91–4.85 (m, 1H, OH), 4.25–4.18 (m, 2H, CH_2_), 4.18–4.12 (m, 1H, CH_2_), 4.12–4.07 (m, 1H, CH_2_), 2.36 (d, *J* = 3.3 Hz, 3H, CH_3_), 1.32 (t, *J* = 7.1 Hz, 3H, CH_2_CH_3_), 1.30 (t, *J* = 7.1 Hz, 3H, CH_2_CH_3_) ppm. ^13^C NMR (CDCl_3_, 150 MHz): *δ* 147.1 (ddd, *J*_1_ = 245.2 Hz, *J*_2_ = 11.1 Hz, *J*_3_ = 1.3 Hz, C-6), 145.1 (ddd, *J*_1_ = 250.0 Hz, *J*_2_ = 15.5 Hz, *J*_3_ = 1.3 Hz, C-7), 138.8 (m, C-3a), 135.7 (t, *J* = 3.7 Hz, C-2), 129.3 (d, *J* = 10.0 Hz, C-3), 127.7 (d, *J* = 14.5 Hz, C-7a), 117.2 (m, C-4), 114.6 (d, *J* = 19.9 Hz, C-5), 66.2 (d, *J* = 168.0 Hz, CH), 64.2 (d, *J* = 7.0 Hz, CH_2_), 63.3 (d, *J* = 7.5 Hz, CH_2_), 16.5 (d, *J* = 5.4 Hz, CH_2_CH_3_), 16.4 (d, *J* = 5.8 Hz, CH_2_CH_3_), 12.5(CH_3_) ppm. ^31^P NMR (CDCl_3_, 242 MHz): *δ* 19.6 ppm. HRMS: [M]^+^ Calcd. for C_14_H_17_F_2_O_4_PS 350.0553; Found 350.0556.

#### 3.2.17. Diethyl [(5-Chloro-3-methyl-1-benzothiophen-2-yl)(hydroxy)methyl]phosphonate (**2q**)


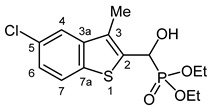


Yield: 276 mg (79%). White crystals. Mp 130–131 °C (EtOAc). IR (KBr): 3279, 2982, 1444, 1231, 1050, 977, 857, 617 cm^−1^. ^1^H NMR (CDCl_3_, 600 MHz): *δ* 7.70 (d, *J* = 8.5 Hz, 1H, H-7), 7.63 (s, 1H, H-4), 7.30–7.27 (m, 1H, H-6), 5.50 (d, *J* = 10.7 Hz, 1H, CH), 4.85 (br s, 1H, OH), 4.20–4.14 (m, 2H, CH_2_), 4.14–4.10 (m, 1H, CH_2_), 4.10–4.03 (m, 1H, CH_2_), 2.34 (d, *J* = 3.1 Hz, 3H, CH_3_), 1.28 (t, *J* = 7.0 Hz, 6H, 2 × CH_2_CH_3_) ppm. ^13^C NMR (CDCl_3_, 150 MHz): *δ* 141.3 (d, *J* = 3.0 Hz, C-4a), 137.4 (d, *J* = 1.6 Hz, C-7a), 136.9 (d, *J* = 5.0 Hz, C-2), 130.2 (C-5), 128.7 (d, *J* = 10.0 Hz, C-3), 124.7 (C-6), 123.4 (d, *J* = 0.7 Hz, C-7), 121.4 (d, *J* = 1.2 Hz, C-4), 66.2 (d, *J* = 167.8 Hz, CH), 64.1 (d, *J* = 6.9 Hz, CH_2_), 63.2 (d, *J* = 7.5 Hz, CH_2_), 16.5 (d, *J* = 5.6 Hz, CH_2_CH_3_), 16.4 (d, *J* = 5.7 Hz, CH_2_CH_3_), 12.3 (d, *J* = 1.2 Hz, CH_3_) ppm. ^31^P NMR (CDCl_3_, 242 MHz): *δ* 19.8 ppm. HRMS: [M]^+^ Calcd. for C_14_H_18_ClO_4_PS 348.0352; Found 348.0339.

#### 3.2.18. Diethyl [(6-Chloro-3-methyl-1-benzothiophen-2-yl)(hydroxy)methyl]phosphonate (**2r**)


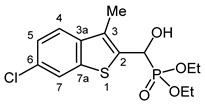


Yield: 229 mg (66%). White crystals. Mp 133–135 °C (MeCN). IR (KBr): 3213, 1453, 1228, 1061, 1020, 809, 758 cm^−1^. ^1^H NMR (DMSO-*d*_6_, 600 MHz): *δ* 8.08 (d, *J* = 1.7 Hz, 1H, H-7), 7.74 (d, *J* = 8.6 Hz, 1H, H-4), 7.41 (dd, *J*_1_ = 8.6 Hz, *J*_2_ = 1.8 Hz, 1H, H-5), 6.76 (dd, *J*_1_ = 17.6 Hz, *J*_2_ = 4.5 Hz, 1H, OH), 5.41 (dd, *J*_1_ = 17.6 Hz, *J*_2_ = 4.5 Hz, 1H, CH), 4.07–4.02 (m, 2H, CH_2_), 4.02–3.96 (m, 2H, CH_2_), 2.34 (d, *J* = 3.0 Hz, 3H, CH_3_), 1.21 (t, *J* = 7.1 Hz, 3H, CH_2_CH_3_), 1.18 (t, *J* = 7.0 Hz, 3H, CH_2_CH_3_) ppm. ^13^C NMR (DMSO-*d*_6_, 150 MHz): *δ* 139.9 (C-7a), 138.9 (d, *J* = 2.7 Hz, C-3a), 138.8 (d, *J* = 3.3 Hz, C-2), 129.3 (C-6), 128.0 (d, *J* = 9.9 Hz, C-3), 124.6 (C-5), 123.3 (C-4), 122.1 (C-7), 65.0 (d, *J* = 172.1 Hz, CH), 62.8 (d, *J* = 7.2 Hz, CH_2_), 62.4 (d, *J* = 6.7 Hz, CH_2_), 16.6 (d, *J* = 5.5 Hz, CH_2_CH_3_), 16.5 (d, *J* = 5.5 Hz, CH_2_CH_3_), 12.2 (CH_3_) ppm. ^31^P NMR (DMSO-*d*_6_, 242 MHz): *δ* 19.9 ppm. HRMS: [M]^+^ Calcd. for C_14_H_18_ClO_4_PS 348.0352; Found 348.0341.

#### 3.2.19. Diethyl {Hydroxy [3-methyl-5-(trifluoromethyl)-1-benzothiophen-2-yl]methyl}phosphonate (**2s**)


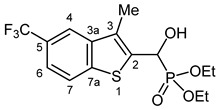


Yield: 253 mg (66%). White crystals. Mp 138–139 °C (EtOAc). IR (KBr): 3241, 2987, 1446, 1350, 1330, 1295, 1203, 1104, 1023, 809 cm^−1^. ^1^H NMR (CDCl_3_, 600 MHz): *δ* 7.92 (s, 1H, H-4), 7.90 (d, *J* = 8.4 Hz, 1H, H-7), 7.55 (d, *J* = 8.3 Hz, 1H, H-6), 5.54 (d, *J* = 10.4 Hz, 1H, CH), 4.23–4.18 (m, 2H, CH_2_), 4.16–4.12 (m, 1H, CH_2_), 4.12–4.05 (m, 1H, CH_2_), 2.42 (d, *J* = 3.0 Hz, 3H, CH_3_), 1.29 (t, *J* = 7.4 Hz, 3H, OCH_2_CH_3_), 1.30 (t, *J* = 7.3 Hz, 3H, OCH_2_CH_3_) ppm. ^13^C NMR (CDCl_3_, 150 MHz): *δ* 142.3 (C-7a), 139.7 (d, *J* = 3.1 Hz, C-3a), 137.0 (d, *J* = 5.3 Hz, C-2), 129.5 (d, *J* = 9.9 Hz, C-3), 126.5 (q, *J* = 32.3 Hz, C-5), 124.7 (q, *J* = 272.0 Hz, CF_3_), 122.9 (C-7), 120.6 (d, *J* = 2.5 Hz, C-6), 118.9 (d, *J* = 2.9 Hz, C-4), 66.2 (d, *J* = 167.5 Hz, CH), 64.2 (d, *J* = 7.0 Hz, CH_2_), 63.3 (d, *J* = 7.5 Hz, CH_2_), 16.5 (d, *J* = 5.4 Hz, OCH_2_CH_3_), 16.4 (d, *J* = 5.8 Hz, OCH_2_CH_3_), 12.3 (d, *J* = 1.2 Hz, CH_3_) ppm. ^31^P NMR (CDCl_3_, 242 MHz): *δ* 19.7 ppm. HRMS: [M]^+^ Calcd. for C_15_H_18_F_3_O_4_PS 382.0616; Found 382.0609.

#### 3.2.20. Diethyl {Hydroxy [3-methyl-6-(trifluoromethyl)-1-benzothiophen-2-yl]methyl}phosphonate (**2t**)


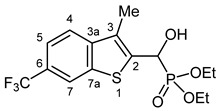


Yield: 265 mg (69%). White crystals. Mp 141–143 °C (EtOAc). IR (KBr): 3229, 2991, 1327, 1230, 1159, 1128, 1056, 1021, 822 cm^−1^. ^1^H NMR (CDCl_3_, 600 MHz): *δ* 8.10 (s, 1H, H-7), 7.76 (d, *J* = 8.4 Hz, 1H, H-4), 7.60 (dd, *J*_1_ = 8.4 Hz, *J*_2_ = 0.9 Hz, H-5), 5.53 (d, *J* = 10.6 Hz, 1H, CH), 4.37 (br s, 1H, OH), 4.23–4.17 (m, 2H, CH_2_), 4.16–4.05 (m, 2H, CH_2_), 2.43 (d, *J* = 3.2 Hz, 3H, CH_3_), 1.31 (t, *J* = 7.3 Hz, 3H, CH_2_CH_3_), 1.30 (t, *J* = 7.3 Hz, 3H, CH_2_CH_3_) ppm. ^13^C NMR (CDCl_3_, 150 MHz): *δ* 142.4 (C-7a), 138.8 (d, *J* = 1.6 Hz, C-3a), 138.2 (d, *J* = 5.3 Hz, C-2), 129.2 (d, *J* = 9.7 Hz, C-3), 126.5 (q, *J* = 32.3 Hz, C-6), 124.5 (q, *J* = 272.1 Hz, CF_3_), 122.1 (d, *J* = 1.4 Hz, C-4), 120.7 (d, *J* = 3.0 Hz, C-5), 119.8 (d, *J* = 3.4 Hz, C-7), 65.9 (d, *J* = 166.7 Hz, CH), 64.1 (d, *J* = 7.0 Hz, CH_2_), 63.3 (d, *J* = 7.5 Hz, CH_2_), 16.5 (d, *J* = 5.5 Hz, CH_2_CH_3_), 16.4 (d, *J* = 5.8 Hz, CH_2_CH_3_), 12.4 (d, *J* = 1.3 Hz, CH_3_) ppm. ^31^P NMR (CDCl_3_, 242 MHz): *δ* 19.5 ppm. HRMS: [M]^+^ Calcd. for C_15_H_18_F_3_O_4_PS 382.0616; Found 382.0618.

#### 3.2.21. Diethyl {Hydroxy [3-methyl-7-(trifluoromethyl)-1-benzothiophen-2-yl]methyl}phosphonate (**2u**)


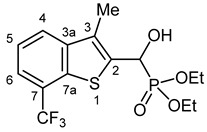


Yield: 299 mg (78%). White crystals. Mp 153–157 °C (EtOAc). IR (KBr): 3441, 3237, 2985, 1629, 1446, 1312, 1246, 1213, 1155, 1113, 1026 cm^−1^. ^1^H NMR (CDCl_3_, 600 MHz): *δ* 7.82 (d, *J* = 8.0 Hz, 1H, H-4), 7.63 (d, *J* = 7.4 Hz, 1H, H-6), 7.46 (t, *J* = 7.7 Hz, 1H, H-5), 5.55 (d, *J* = 10.3 Hz, 1H, CH), 4.27–4.20 (m, 2H, CH_2_), 4.18–4.11 (m, 1H, CH_2_), 4.11–4.04 (m, 2H, CH_2_, OH), 2.40 (d, *J* = 3.2 Hz, 3H, CH_3_), 1.23 (t, *J* = 7.1 Hz, 3H, CH_2_CH_3_), 1.29 (t, *J* = 7.1 Hz, 3H, CH_2_CH_3_) ppm. ^13^C NMR (CDCl_3_, 150 MHz): *δ* 141.8 (d, *J* = 3.1 Hz, C-3a), 136.5 (d, *J* = 5.3 Hz, C-2), 135.6 (m, C-7a), 128.8 (d, *J* = 9.8 Hz, C-3), 125.2 (C-4), 124.6 (qd, *J*_q_ = 32.7 Hz, *J*_d_ = 1.2 Hz, C-7), 124.2 (q, *J* = 273.4 Hz, CF_3_), 123.7 (C-5), 122.1 (q, *J* = 4.5 Hz, C-6), 66.3 (d, *J* = 167.4 Hz, CH), 64.3 (d, *J* = 7.0 Hz, CH_2_), 63.3 (d, *J* = 7.5 Hz, CH_2_), 16.4 (d, *J* = 5.5 Hz, CH_2_CH_3_), 16.3 (d, *J* = 5.9 Hz, CH_2_CH_3_), 12.4 (d, *J* = 1.3 Hz, CH_3_) ppm. ^31^P NMR (CDCl_3_, 242 MHz): *δ* 19.6 ppm. HRMS: [M]^+^ Calcd. for C_15_H_18_F_3_O_4_PS 382.0616; Found 382.0607.

#### 3.2.22. Diethyl [(3-Ethyl-5-trifluoromethyl-1-benzothiophen-2-yl)](hydroxy)methyl]phosphonate (**2v**)


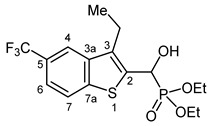


Yield: 306 mg (77%). White crystals. Mp 156–157 °C (EtOAc). IR (KBr): 3258, 2975, 1756, 1610, 1567, 1439, 1394, 1354, 1328, 1235, 1221, 1116, 978 cm^−1^. ^1^H NMR (CDCl_3_, 600 MHz): *δ* 7.95 (s, 1H, H-4), 7.91 (d, *J* = 8.4 Hz, 1H, H-7), 7.55 (d, *J* = 8.4 Hz, 1H, H-6), 5.53 (d, *J* = 10.9 Hz, 1H, CH), 4.20–4.17 (m, 2H, OCH_2_CH_3_), 4.16–4.11 (m, 2H, OCH_2_CH_3_), 2.97–2.95 (m, 1H, CH_2_CH_3_), 2.91–2.89 (m, 1H, CH_2_CH_3_), 1.29 (m, 6H, OCH_2_CH_3_), 1.28 (m, 3H, CH_2_CH_3_) ppm. ^13^C NMR (CDCl_3_, 150 MHz): *δ* 142.9 (C-7a), 138.7 (d, *J* = 2.6 Hz, C-3a), 136.6 (d, *J* = 4.6 Hz, C-2), 136.3 (d, *J* = 10.4 Hz, C-3), 126.5 (q, *J* = 32.2 Hz, C-5), 124.6 (q, *J* = 272.0 Hz, CF_3_), 123.1 (C-7), 120.6 (m, C-6), 118.9 (m, C-4), 65.8 (d, *J* = 168.0 Hz, CH), 64.1 (d, *J* = 7.0 Hz, OCH_2_CH_3_), 63.3 (d, *J* = 7.5 Hz, OCH_2_CH_3_), 20.1 (CH_2_CH_3_), 16.5 (d, *J* = 5.5 Hz, OCH_2_CH_3_), 16.4 (d, *J* = 5.7 Hz, OCH_2_CH_3_), 14.4 (d, *J* = 2.6 Hz, CH_2_CH_3_) ppm. ^31^P NMR (CDCl_3_, 242 MHz): *δ* 19.6 ppm. HRMS: [M + H]^+^ Calcd. for C_16_H_21_F_3_O_4_PS 397.0850; Found 397.0842.

#### 3.2.23. Diethyl [(4-Fluoro-7-iodo-1-benzothiopen-2-yl)(hydroxy)methyl]phosphonate (**2w**)


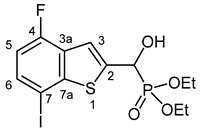


Yield: 300 mg (68%). White crystals. Mp 145–146 °C (EtOAc). IR (KBr): 3225, 2983, 1458, 1251, 1203, 1042, 977 cm^−1^. ^1^H NMR (CDCl_3_, 600 MHz): *δ* 7.72 (d, *J* = 3.5 Hz, 1H, H-3), 7.57 (dd, *J*_1_ = 8.3 Hz, *J*_2_ = 4.6 Hz, 1H, H-6), 6.81 (dd, *J*_1_ = 9.7 Hz, *J*_2_ = 8.3 Hz, 1H, H-5), 5.35 (d, *J* = 12.1 Hz, 2H, CH, OH), 4.25–4.15 (m, 4H, 2 × CH_2_), 1.35 (t, *J* = 7.1 Hz, 3H, CH_3_), 1.32 (t, *J* = 7.1 Hz, 3H, CH_3_) ppm. ^13^C NMR (CDCl_3_, 150 MHz): *δ* 157.8 (dd, *J*_1_ = 252.8 Hz, *J*_2_ = 1.1 Hz, C-4), 148.3 (dd, *J*_1_ = 6.4 Hz, *J*_2_ = 1.5 Hz, C-7a), 142.1 (d, *J* = 1.7 Hz, C-2), 134.1 (d, *J* = 6.4 Hz, C-6), 128.0 (dd, *J*_1_ = 20.0 Hz, *J*_2_ = 2.6 Hz, C-3a), 118.2 (d, *J* = 8.0 Hz, C-3), 111.4 (d, *J* = 20.1 Hz, C-5), 80.0 (d, *J* = 3.0 Hz, C-7), 67.5 (d, *J* = 165.5 Hz, CH), 64.2 (d, *J* = 7.1 Hz, CH_2_), 63.3 (d, *J* = 7.4 Hz, CH_2_), 16.5 (d, *J* = 5.3 Hz, CH_3_), 16.4 (d, *J* = 5.3 Hz, CH_3_) ppm. ^31^P NMR (CDCl_3_, 242 MHz): *δ* 18.6 ppm. HRMS: [M + 1]^+^ Calcd. for C_13_H_16_FIO_4_PS 444.9536; Found 444.9531.

### 3.3. X-Ray Experimental

#### Single Crystal X-Ray Diffraction Studies

Single crystals of compound **2f**, suitable for X-ray diffraction, were obtained by slow evaporation of ethyl acetate solution. The crystals were introduced into perfluorinated oil, and a suitable single crystal was carefully mounted on the top of a thin glass wire. Data collection was performed with an Oxford Xcalibur 3 diffractometer (Oxford Diffraction, Abingdon. UK) equipped with a Spellman generator (50 kV, 40 mA) and a Kappa CCD detector, operating with Mo-K_α_ radiation (λ = 0.71071 Ǻ).

Data collection and data reduction were performed with the CrysAlisPro version 1.171.40.82a software [46]. Absorption correction using the multiscan method [46] was applied. The structures were solved with SHELXS-97 [47], refined with SHELXL-97 [48], and finally checked using PLATON [49]. Details for data collection and structure refinement are summarized in Table 3.

CCDC-2440279 contains supplementary crystallographic data for this compound. These data can be obtained free of charge from The Cambridge Crystallographic Data Centre via www.ccdc.cam.ac.uk/data_request/cif (last accessed on 16 June 2025).

### 3.4. Bioactivity Experimental

#### 3.4.1. Cell Culturing

In our study, four different human cell lines were tested to screen the antitumor effect of the α-hydroxyphosphonates. The myeloma (U266), colon adenocarcinoma cell line (HT-29), and the metastatic melanoma (A2058) were obtained from the European Collection of Authenticated Cell Cultures (ECACC, Salisbury, UK). The lung squamous carcinoma cells (EBC-1) were purchased from the Japanese Collection of Research Bioresources Cell Bank (JCRB Cell Bank, Osaka, Japan). These cell lines represent different types of cancers, allowing us to test how broadly applicable or specific our investigated molecules are across malignancies. The EBC-1, A2058, and HT-29 cell lines have adherent growth properties, but the U266 cells (85051003 ECACC) grow in suspension.

EBC-1 cells were cultured in DMEM medium (Sigma Ltd., St. Louis, MO, USA), while HT-29, A2058, and U266 cells were cultured in RPMI 1640 (Sigma Ltd., St. Louis, MO, USA). Both media were supplemented with 10% fetal bovine serum (Invitrogen Corporation, New York, NY, USA), 1% L-glutamine (Invitrogen Corporation, New York, NY, USA), and 1% penicillin/streptomycin (Invitrogen Corporation, New York, NY, USA). The medium for EBC-1 was further completed with 1% non-essential amino acids (Sigma Ltd., St. Louis, MO, USA) and 1% sodium pyruvate (Sigma Ltd., St. Louis, MO, USA).

#### 3.4.2. Cell Viability Assays

Compounds were dissolved in dimethyl sulfoxide (DMSO; AppliChem GmbH, Darmstadt, Germany) to prepare stock solutions at a concentration of 10⁻^1^ M. During experiments, the final DMSO concentration was kept below 1% (*v*/*v*). Stock solutions were stored at −80 °C, and working solutions were freshly prepared prior to each experiment. Adherent EBC-1, A2058, and HT-29 cells were seeded in transparent 96-well plates (Sarstedt AG, Nümbrecht, Germany) at a density of 1 × 10^5^ cells/mL. After overnight incubation, cells were treated with test compounds at a final concentration of 100 μM, alongside appropriate controls (medium only and vehicle control with DMSO). Following a 72 h incubation period with the compounds, 11 μL of alamarBlue reagent (Thermo Scientific, Waltham, MA, USA) was added to each well, and plates were incubated for an additional 3 h. Fluorescence was measured using a Fluoroskan FL Microplate Fluorometer and Luminometer (Thermo Scientific, Waltham, MA, USA) at excitation/emission wavelengths of 530–560 nm and 590 nm, respectively.

Due to technical difficulties in assessing U266 cell viability using the alamarBlue assay, the CellTiter-Glo Luminescent Cell Viability Assay (Promega, Madison, WI, USA) was employed instead, following the protocol described in our previous publication [50]. All experiments were conducted in triplicate within a single experimental run, and results were normalized to the DMSO vehicle control using OriginPro 8 software (OriginLab Corporation, Northampton, MA, USA) and reported as mean ± standard deviation (SD).

## 4. Conclusions

Encouraged by our earlier experiences that the modification of α-hydroxyphosphonates may lead to derivatives displaying significant biological activity, including cytotoxic effects, 23 members of a new α-hydroxyphosphonate family with the benzothiophene scaffold were synthesized by the Pudovik reaction of benzo[*b*]thiophene-2-carboxaldehydes and diethyl phosphite performed at 26 °C in the presence of triethylamine as the catalyst. All compounds were fully characterized, including one single-crystal X-ray analysis. With a few exceptions, most of the compounds affected the viability of the tumor cell lines, such as the U266 (multiple myeloma), the EBC-1 (lung cancer), the A2058 (melanoma), and the HT-29 (colon cancer) cell cultures—with U266 cells displaying the highest sensitivity. Single “halogenation” generally had weaker effects as compared to double “halogenation” or substitution with a trifluoromethyl group. Notably, the meta-substituted hydroxyphosphonate bearing a trifluoromethyl group showed the strongest antiproliferative effect on three of the four cell lines, which may be attributed to its increased lipophilicity and stability, underscoring its potential as a promising lead compound.

## Figures and Tables

**Table 1:** Preparative data for the new α-benzothiophenyl-α-hydroxy-ethylphosphonates (**2a**–**w**).

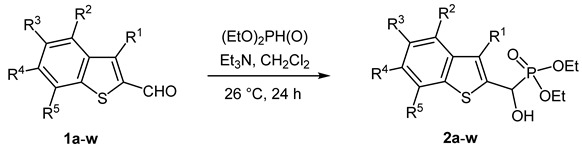							
Entry	Product	R^1^	R^2^	R^3^	R^4^	R^5^	Yield (%)
1	**2a**	H	H	F	H	H	75
2	**2b**	H	H	H	F	H	87
3	**2c**	H	H	H	H	F	94
4	**2d**	H	Cl	H	H	H	72
5	**2e**	H	H	Cl	H	H	62
6	**2f**	H	H	H	H	Cl	86
7	**2g**	H	H	Br	H	H	73
8	**2h**	H	I	H	H	H	66
9	**2i**	Me	H	H	H	H	61
10	**2j**	H	CF_3_	H	H	H	79
11	**2k**	Me	F	H	H	H	71
12	**2l**	Me	H	F	H	H	79
13	**2m**	Me	H	H	F	H	51
14	**2n**	Me	H	H	H	F	79
15	**2o**	Me	H	F	H	F	75
16	**2p**	Me	H	H	F	F	56
17	**2q**	Me	H	Cl	H	H	79
18	**2r**	Me	H	H	Cl	H	66
19	**2s**	Me	H	CF_3_	H	H	66
20	**2t**	Me	H	H	CF_3_	H	69
21	**2u**	Me	H	H	H	CF_3_	78
22	**2v**	Et	H	CF_3_	H	H	77
23	**2w**	H	F	H	H	I	68

Me: methyl; Et: ethyl.

**Table 2:** Cell viability of U266 myeloma, EBC-1 lung, A2058 melanoma, and HT-29 colon cell lines following long-term treatment (72 h) with α-benzothiophenyl-α-hydroxy-methylphosphonate derivatives (**2a**–**w**) at 100 μM. The data are expressed as mean ± standard deviation (SD), with a sample size of n = 3. Statistical analysis was performed using a one-way ANOVA followed by Fisher’s LSD post hoc test. Significance levels are denoted as follows: *: *p* < 0.05, **: *p* < 0.01, or ***: *p* < 0.001.

Compounds	U266	EBC-1	A2058	HT-29
	100 µM			
Medium	2.05 ± 0.21 ***	0.92 ± 0.04 ***	1.03 ± 0.04	0.87 ± 0.02 ***
DMSO	1.00 ± 0.05	1.00 ± 0.02	1.00 ± 0.07	1.00 ± 0.04
**2a**	0.85 ± 0.07 ***	0.93 ± 0.02 **	0.96 ± 0.06 **	0.90 ± 0.02 *
**2b**	0.76 ± 0.04 ***	0.97 ± 0.01	0.97 ± 0.06 ***	0.97 ± 0.02
**2c**	0.78 ± 0.06 ***	0.89 ± 0.02 ***	0.93 ± 0.05 **	0.90 ± 0.01 **
**2d**	0.55 ± 0.07 ***	0.77 ± 0.01 ***	0.82 ± 0.03 ***	0.83 ± 0.03 ***
**2e**	0.52 ± 0.05 ***	0.71 ± 0.02 ***	0.90 ± 0.02 ***	0.86 ± 0.02 ***
**2f**	0.51 ± 0.03 ***	0.66 ± 0.03 ***	0.78 ± 0.11 ***	0.79 ± 0.03 ***
**2g**	0.46 ± 0.01 ***	0.71 ± 0.01 ***	0.92 ± 0.01 ***	0.91 ± 0.06 **
**2h**	0.51 ± 0.01 ***	0.66 ± 0.04 ***	0.76 ± 0.01 ***	0.81 ± 0.04 ***
**2i**	0.94 ± 0.01	0.99 ± 0.03 *	1.06 ± 0.01	0.98 ± 0.01
**2j**	0.61 ± 0.02 ***	0.76 ± 0.02 ***	0.80 ± 0.02 ***	0.78 ± 0.03 ***
**2k**	1.08 ± 0.05	0.78 ± 0.03 ***	0.83 ± 0.03 ***	0.83 ± 0.02 ***
**2l**	0.75 ± 0.08 ***	0.93 ± 0.01 **	0.97 ± 0.05 *	0.92 ± 0.03 *
**2m**	0.87 ± 0.01 **	0.89 ± 0.02 ***	0.90 ± 0.04 ***	0.95 ± 0.04 *
**2n**	0.81 ± 0.08 ***	0.86 ± 0.03 ***	0.90 ± 0.01 ***	0.91 ± 0.02 *
**2o**	0.63 ± 0.02 ***	0.93 ± 0.05 *	0.98 ± 0.04*	0.96 ± 0.02
**2p**	0.69 ± 0.02 ***	0.85 ± 0.01 ***	0.82 ± 0.01 ***	0.91 ± 0.02 **
**2q**	0.52 ± 0.06 ***	0.91 ± 0.04 ***	0.92 ± 0.02 ***	0.92 ± 0.02 **
**2r**	0.51 ± 0.05 ***	0.68 ± 0.02 ***	0.89 ± 0.06 ***	0.84 ± 0.04 ***
**2s**	0.30 ± 0.01 ***	0.71 ± 0.01 ***	1.02 ± 0.01	0.86 ± 0.03 ***
**2t**	0.09 ± 0.02 ***	0.65 ± 0.01 ***	0.95 ± 0.05 **	0.58 ± 0.14 ***
**2u**	0.55 ± 0.05 ***	0.85 ± 0.01 ***	0.82 ± 0.04 ***	0.87 ± 0.02 ***
**2v**	0.77 ± 0.02 ***	1.03 ± 0.01	1.05 ± 0.01	0.99 ± 0.01
**2w**	0.33 ± 0.01 ***	0.69 ± 0.01 ***	0.71 ± 0.01 ***	0.75 ± 0.01 ***

DMSO: dimethyl sulfoxide. Blue indicates a reduction in cell viability to 80–65%, pale orange to 65–50%, and pale pink to 50% or less.

**Table 3:** Details for X-ray data collection and structure refinement for compound **2f**.

	2f
Empirical formula	C_13_H_16_ClO_4_PS
Formula mass	334.74
T [K]	123(2)
Crystal size [mm]	0.40 × 0.25 × 0.20
Crystal description	colorless block
Crystal system	triclinic
Space group	*P*-1
a [Ǻ]	7.6579(4)
b [Ǻ]	7.8950(4)
c [Ǻ]	13.9491(6)
α [°]	78.234(4)
β [°]	85.603(4)
γ [°]	65.219(5)
V [Ǻ^3^]	749.57(7)
Z	2
ρ_calcd_. [g cm^−3^]	1.483
μ [mm^−1^]	0.509
*F*(000)	348
Θ range [°]	2.89–25.24
Index ranges	−10 ≤ *h* ≤ 10
	−10 ≤ *k* ≤ 10
	−18 ≤ *l* ≤ 18
Reflns. collected	12,988
Reflns. obsd.	3270
Reflns. unique	3718 (R_int_ = 0.0204)
*R*_1_, *wR*_2_ (2σ data)	0.0405, 0.1050
*R*_1_, *wR*_2_ (all data)	0.0471, 0.1111
GOOF on *F*^2^	1.029
Peak/hole [e Ǻ^−3^]	0.754/−0.457

## Data Availability

Data is contained within the article and supplementary material.

## Supplementary Materials

The following supporting information can be downloaded at: https://www.mdpi.com/article/10.3390/ph18070949/s1, X-ray data for 7-chlorobenzothiophenyl-α-hydroxy-methylphosphonate (**2f**); ^31^P, ^13^C, and ^1^H NMR and IR spectra for compounds **2a**–**w** synthesized; HSQC, HMBC, and COSY NMR spectra for the compounds synthesized; Figure S1. View of the molecular structure of compound **2f** showing the full disorder of the molecule in the crystal; Table S1. Selected bond lengths (Å) of hydroxy-methylphosphonate **2f**; Table S2. Selected bond angles (°) of hydroxy-methylphosphonate **2f**; Table S3. Selected torsion angles (°) of hydroxy-methylphosphonate **2f**.
