# Supplementary material for: Synthesis and Cytotoxic Activity of a New Family of α-Hydroxyphosphonates with the Benzothiophene Scaffold
Source: Pharmaceuticals (Basel). 2025 Jun 24;18(7):949. doi: 10.3390/ph18070949 (PMC12300344; doi:10.3390/ph18070949)

Supplementary Materials

Synthesis and Cytotoxic Activity of a New Family of α-Hydroxyphosphonates with the Benzothiophene Scaffold

Mátyás Milen,[1,*] Tamás Miklós John,[1,2] Anna Sára Kis,[2] Zsófia Garádi,[1] Zsuzsanna Szalai,[2] Angéla Takács,[3] László Kőhidai,[3] Konstantin Karaghiosoff,[4] and György Keglevich[2,*]

[1]Directorate of Egis Pharmaceuticals Plc., Directorate of Drug Substance Development, 1475 Budapest, P.O. Box 100, Hungary; johntamas@edu.bme.hu (T.M.J.); garadi.zsofia@egis.hu (Z.G.)

[2]Department of Organic Chemistry and Technology, Faculty of Chemical Technology and Biotechnology, Budapest University of Technology and Economics, Műegyetem rkp. 3, 1111 Budapest, Hungary; kisannasari@gmail.com (S.A.K.); szalai.zsuzsanna@edu.bme.hu (Z.S.)

[3]Department of Genetics, Cell and Immunobiology, Semmelweis University, Nagyvárad tér 4, 1089 Budapest, Hungary; takacs.angela@semmelweis.hu (A.T.); kohidai.laszlo@semmelweis.hu (L.K.)

[4]Department Chemie, Ludwig-Maximilians-Universität München, Butenandtstr. 5-13, D-81377 München, Germany; klk@cup.uni-muenchen.de

Correspondence: milen.matyas@egis.hu, Tel.: +36-1-803-5874, (M.M.); keglevich.gyorgy@vbk.bme.hu, Tel.: +36-1-463-1111 (ext. 5883) (G.K.)

Table of Contents

1. Geometrical data for 7-chlorobenzothiophenyl-α-hydroxy-methylphosphonate (**2f**) obtained from the X-ray measurements S2
2. ^{31}P, ^{13}C, ^{1}H NMR and IR spectra for the hydroxy-methylphosphonates **2a-w** synthesized.... S5
3. HSQC, HMBC and COSY NMR spectra for the compounds synthesized S51

1. Geometrical data for 7-chlorobenzothiophenyl-α-hydroxy-methylphosphonate (2f) obtained from the X-ray measurements

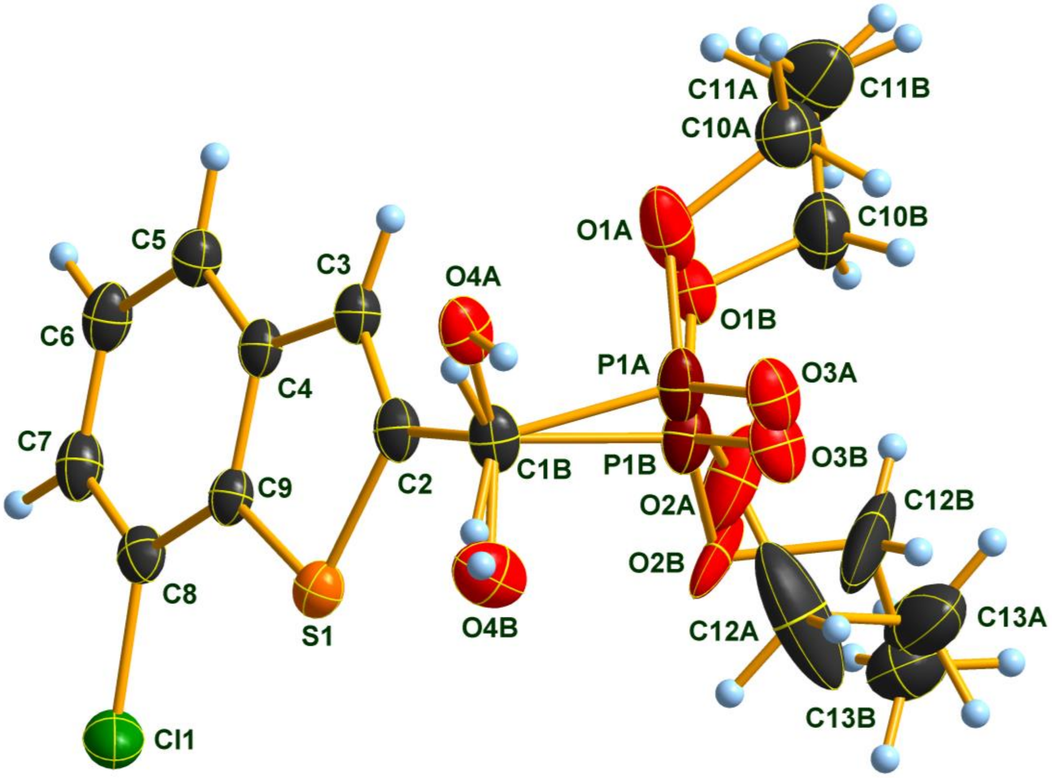

Figure S1. View of the molecular structure of compound **2f** showing the full disorder of the molecule in the crystal. The P(O)(OEt)$_2$ group is disordered of ver two positions (A and B) with a population ratio of 66 : 34, respectively. Also the OH group is disordered over two positions (A and B) with a population ratio of 89 : 11, respectively. The coordinates and thermal ellipsoids of C1A and C1B have been set equal in this structure model.

Table S1. Selected bond lengths (Å) of hydroxy-methylphosphonate **2f**.

S1 – C9	1.730(2)	C1A – O4B	1.392(3)
S1 – C2	1.742(2)	C5 – C6	1.386(3)
C3 – C2	1.352(3)	P1A – O3A	1.480(6)
C3 – C4	1.436(2)	P1A – O2A	1.534(4)
Cl1 – C8	1.732(2)	P1A – O1A	1.569(4)
C7 – C8	1.374(3)	P1A – C1A	1.824(3)
C7 – C6	1.403(3)	O2A – C12A	1.421(1)
C9 – C8	1.396(2)	C12A – C13A	1.540(1)
C9 – C4	1.405(2)	O1A – C10A	1.433(5)
C4 – C5	1.401(2)	C10A – C11A	1.496(6)
C2 – C1A	1.506(2)		

Table S2. Selected bond angles (°) of hydroxy-methylphosphonate **2f**.

C9 – S1 – C2	90.6(1)	C5 – C6 – C7	121.3(2)
C2 – C3 – C4	113.3(2)	O4B – C1A – C2	111.1(2)
C8 – C7 – C6	119.8(2)	O4B – C1A – P1A	110.3(2)
C8 – C9 – C4	120.4(2)	C2 – C1A – P1A	114.9(1)
C8 – C9 – S1	127.1(1)	O3A – P1A – O2A	113.8(2)
C4 – C9 – S1	112.5(1)	O3A – P1A – O1A	113.6(2)
C5 – C4 – C9	119.7(2)	O2A – P1A – O1A	105.5(2)
C5 – C4 – C3	129.6(2)	O3A – P1A – C1A	113.4(3)
C9 – C4 – C3	110.7(2)	O2A – P1A – C1A	105.9(2)
C3 – C2 – C1A	129.3(2)	O1A – P1A – C1A	103.7(2)
C3 – C2 – S1	112.9(1)	C12A – O2A – P1A	125.1(3)
C1A – C2 – S1	117.8(1)	O2A – C12A – C13A	107.7(5)
C6 – C5 – C4	118.9(2)	C10A – O1A – P1A	122.8(3)
C7 – C8 – C9	119.9(2)	O1A – C10A – C11A	109.5(4)
C7 – C8 – Cl1	120.9(1)	C9 – C8 – Cl1	119.2(1)

Table S3. Selected torsion angles (°) of hydroxy-methylphosphonate **2f**.

C2 – S1 – C9 – C8	178.4(2)	C4 – C5 – C6 – C7	-0.1(3)
C2 – S1 – C9 – C4	-0.7(1)	C8 – C7 – C6 – C5	0.3(3)
C8 – C9 – C4 – C5	1.2(2)	C3 – C2 – C1A – O4B	170.6(2)
S1 – C9 – C4 – C5	-179.6(1)	S1 – C2 – C1A – O4B	-8.2(2)
C8 – C9 – C4 – C3	-178.5(2)	C3 – C2 – C1A – P1A	-63.4(2)
S1 – C9 – C4 – C3	0.7(2)	S1 – C2 – C1A – P1A	117.8(2)
C2 – C3 – C4 – C5	-180.0(2)	O3A – P1A – C1A – O4B	-53.5(2)
C2 – C3 – C4 – C9	-0.4(2)	O2A – P1A – C1A – O4B	72.0(2)
C4 – C3 – C2 – C1A	-179.0(2)	O1A – P1A – C1A – O4B	-177.2(2)
C4 – C3 – C2 – S1	-0.2(2)	O3A – P1A – C1A – C2	-179.9(2)
C9 – S1 – C2 – C3	0.5(1)	O2A – P1A – C1A – C2	-54.4(2)
C9 – S1 – C2 – C1A	179.5(1)	O1A – P1A – C1A – C2	56.4(2)
C9 – C4 – C5 – C6	-0.6(2)	O1A – P1A – O2A – C12A	160.1(5)
C3 – C4 – C5 – C6	178.9(2)	C1A – P1A – O2A – C12A	-90.3(5)
C6 – C7 – C8 – C9	0.2(3)	P1A – O2A – C12A – C13A	-138.2(6)
C6 – C7 – C8 – Cl1	-178.1(1)	O3A – P1A – O1A – C10A	43.1(5)
C4 – C9 – C8 – C7	-1.0(3)	O2A – P1A – O1A – C10A	-82.2(4)
S1 – C9 – C8 – C7	180.0(1)	C1A – P1A – O1A – C10A	166.7(3)
C4 – C9 – C8 – Cl1	177.3(1)	P1A – O1A – C10A – C11A	121.7(5)
S1 – C9 – C8 – Cl1	-1.7(2)	O3A – P1A – O2A – C12A	34.9(6)

2. ^{31}P, ^{13}C, ^{1}H NMR and IR spectra for the hydroxymethylphosphonates 2a-w synthesized

^{31}P NMR (242 MHz, $CDCl_3$) spectra for compound 2a

2a

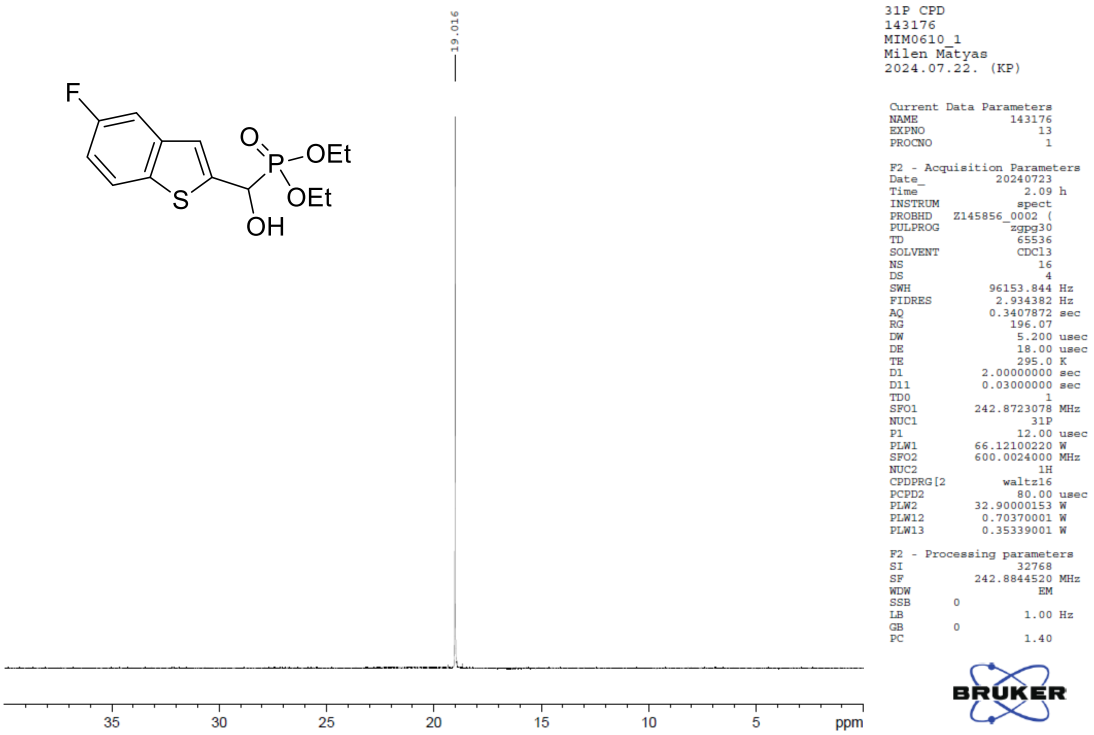

^{13}C NMR (150 MHz, $CDCl_3$) spectra for compound 2a

2a

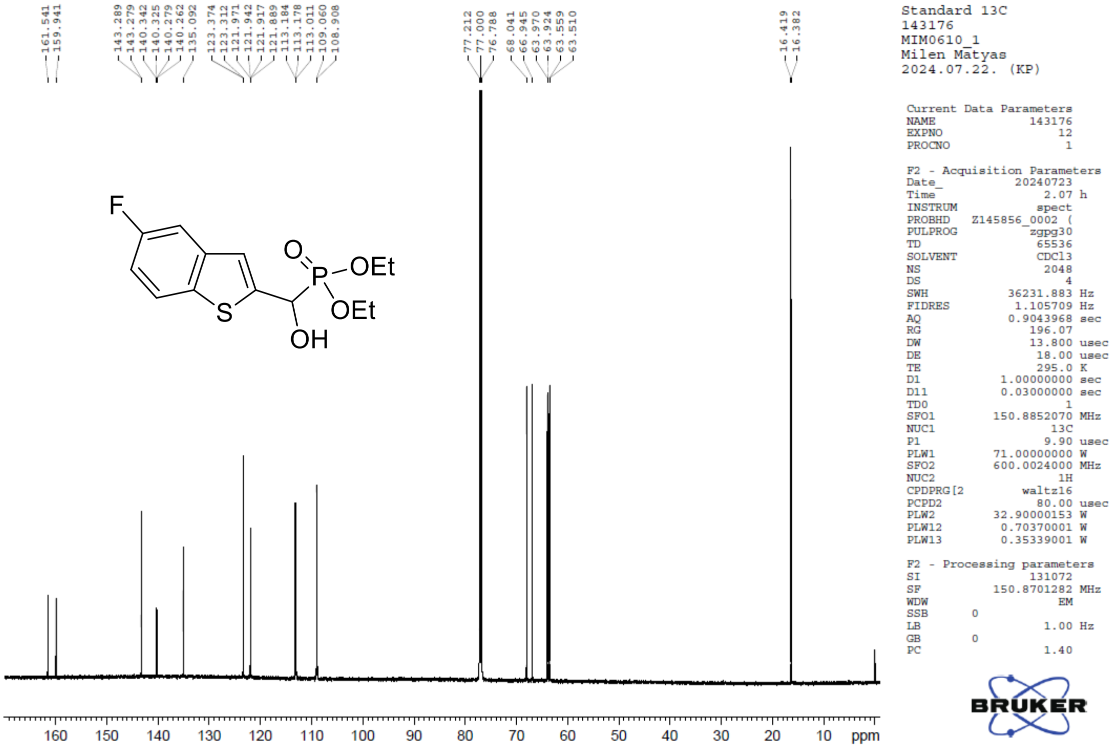

^{1}H NMR (600 MHz, $CDCl_3$) spectra for compound 2a

2a

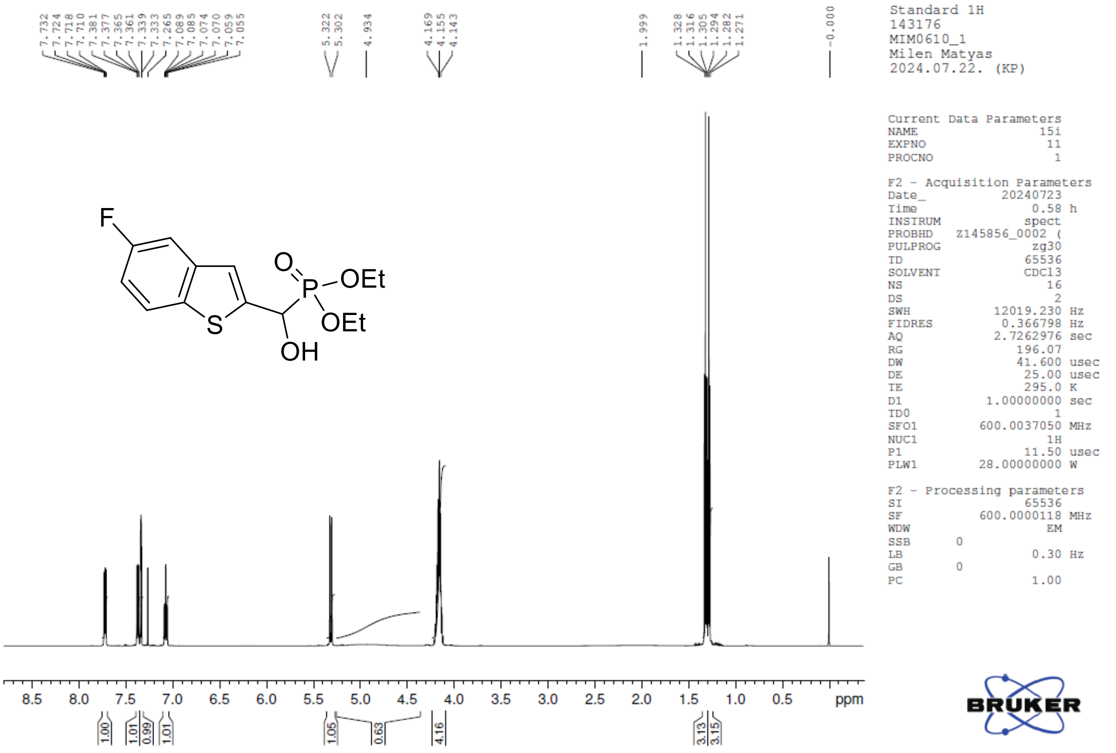

IR (KBr) spectra for compound 2a

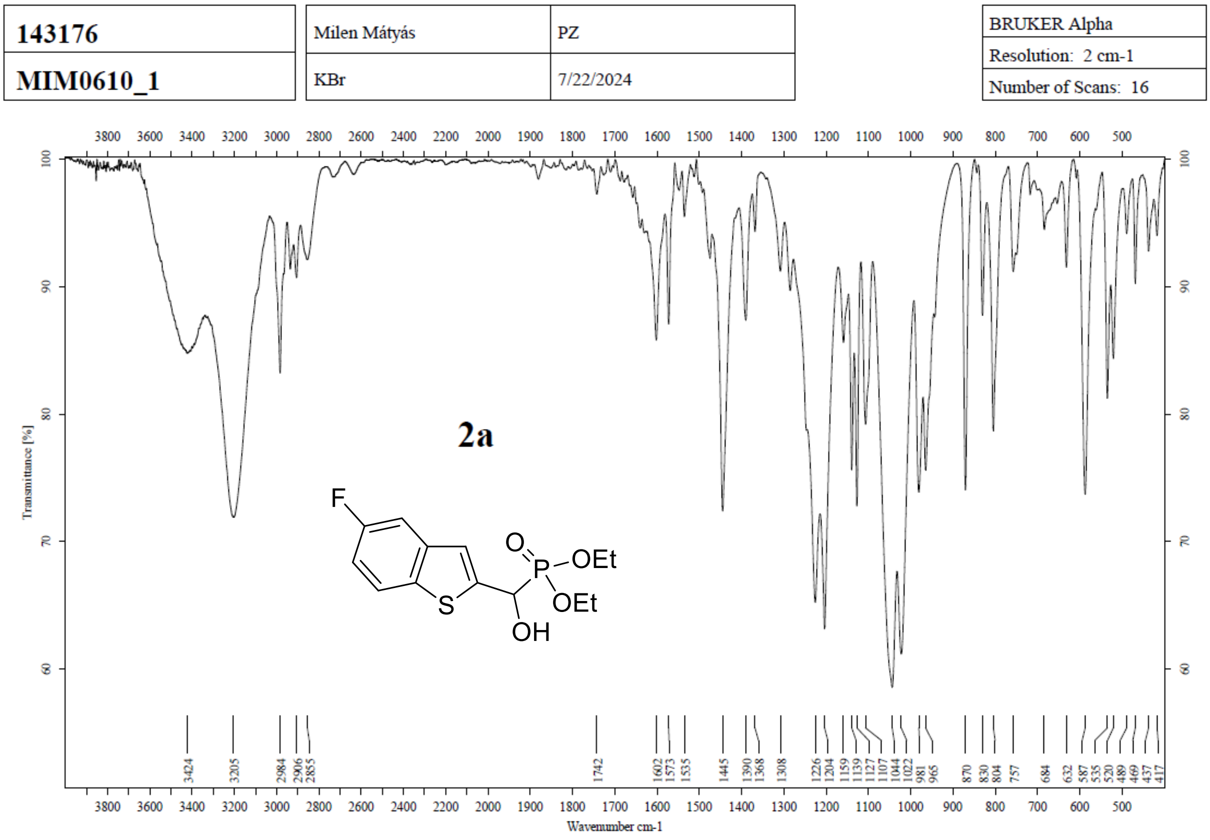

^{31}P NMR (242 MHz, CDCl$_3$) spectra for compound 2b

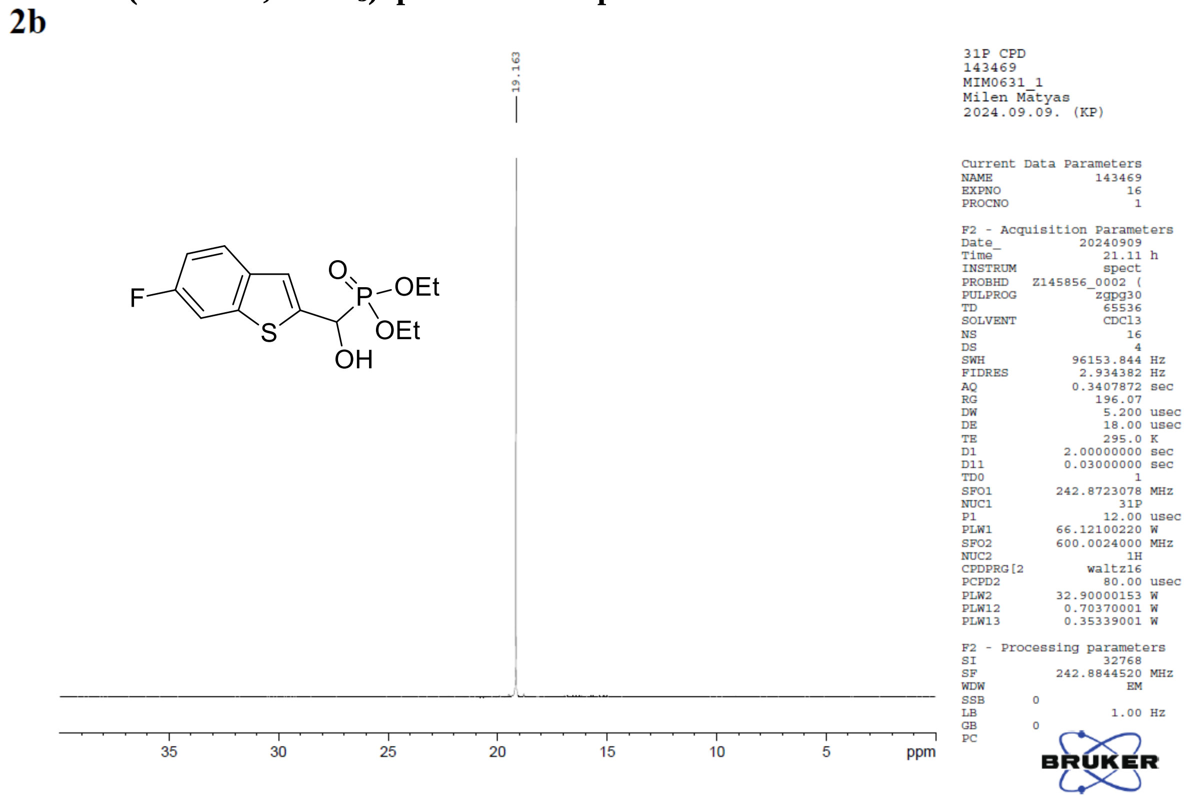

^{13}C NMR (150 MHz, CDCl$_3$) spectra for compound 2b

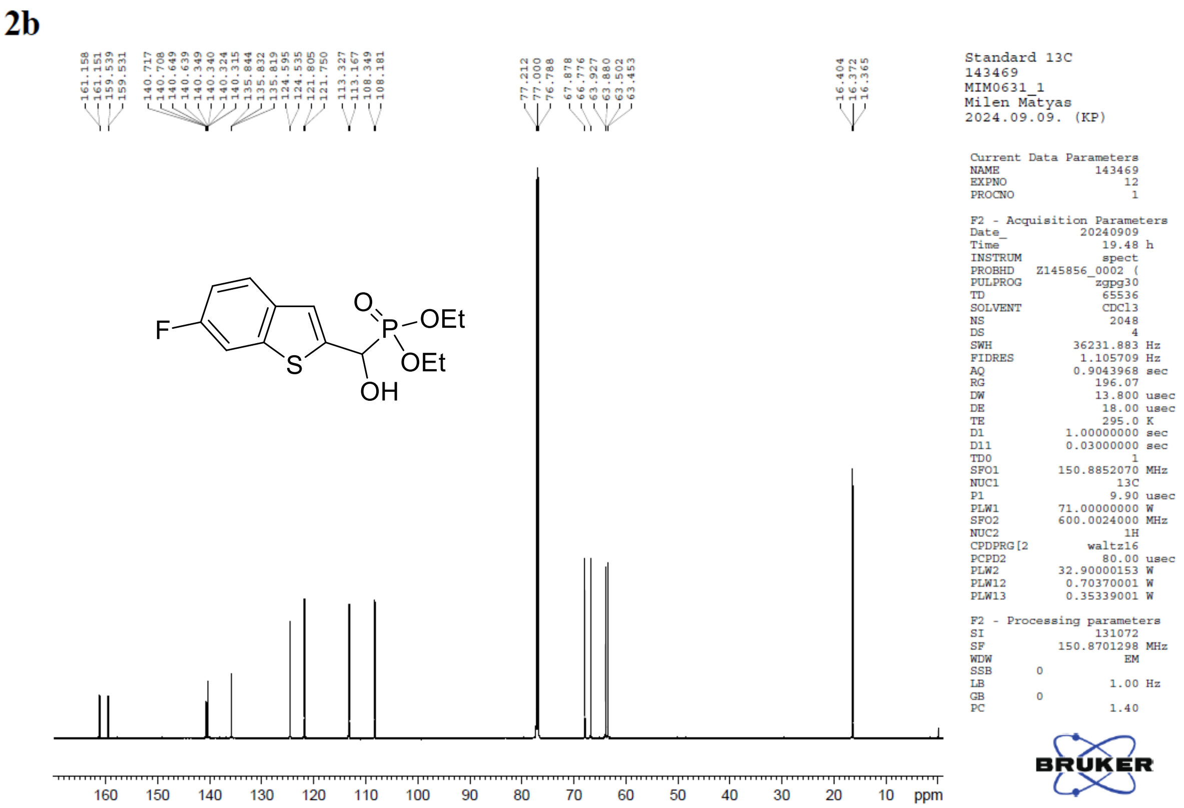

^{1}H NMR (600 MHz, $CDCl_3$) spectra for compound 2b

2b

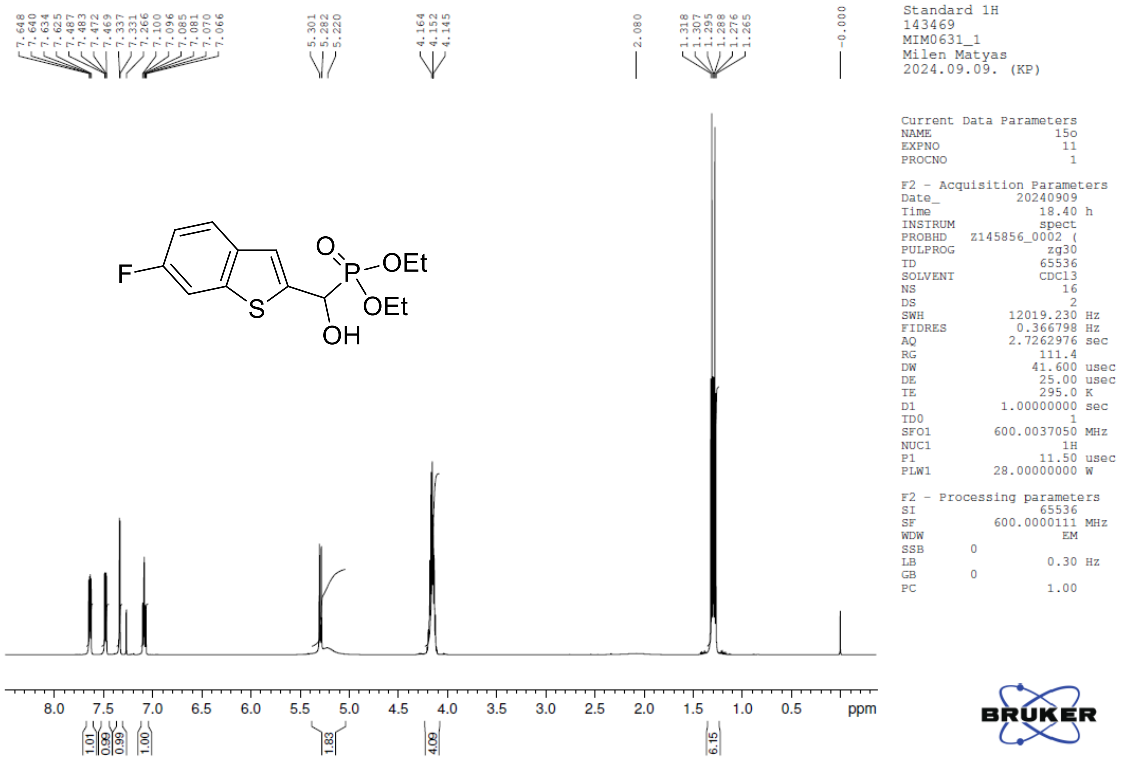

IR (KBr) spectra for compound 2b

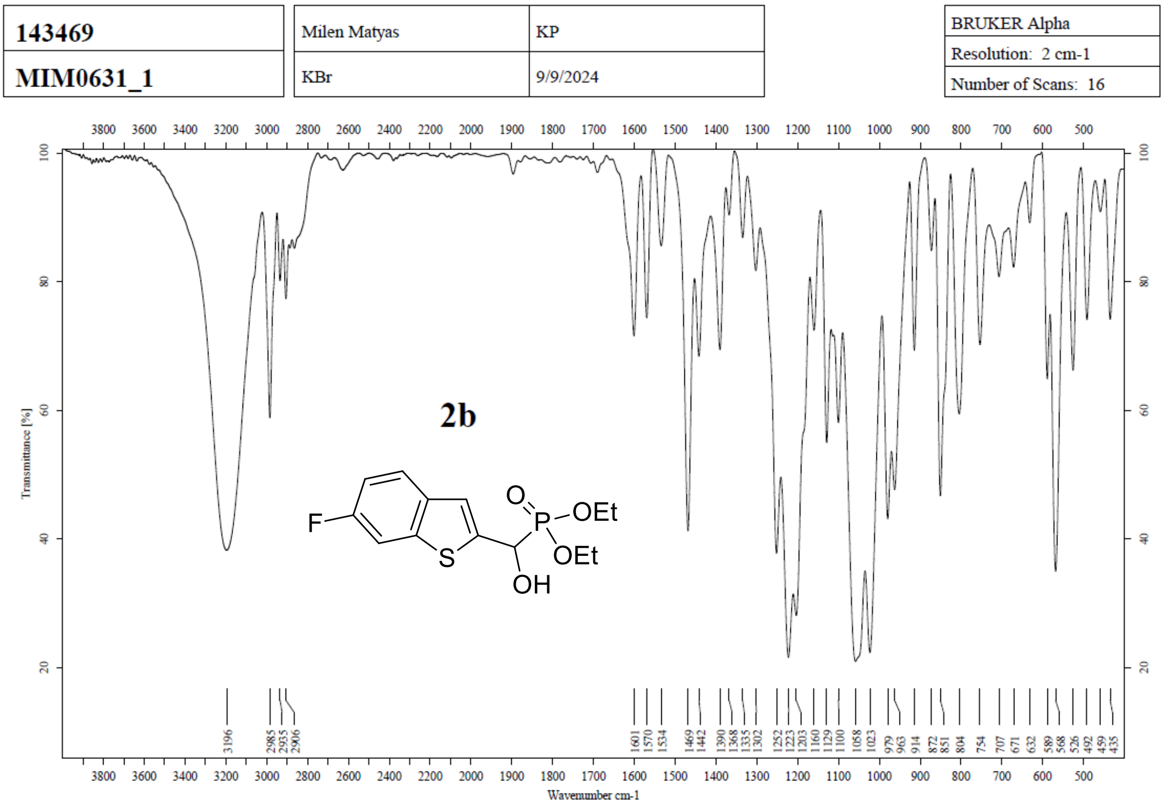

^{31}P NMR (242 MHz, $CDCl_3$) spectra for compound 2c

2c

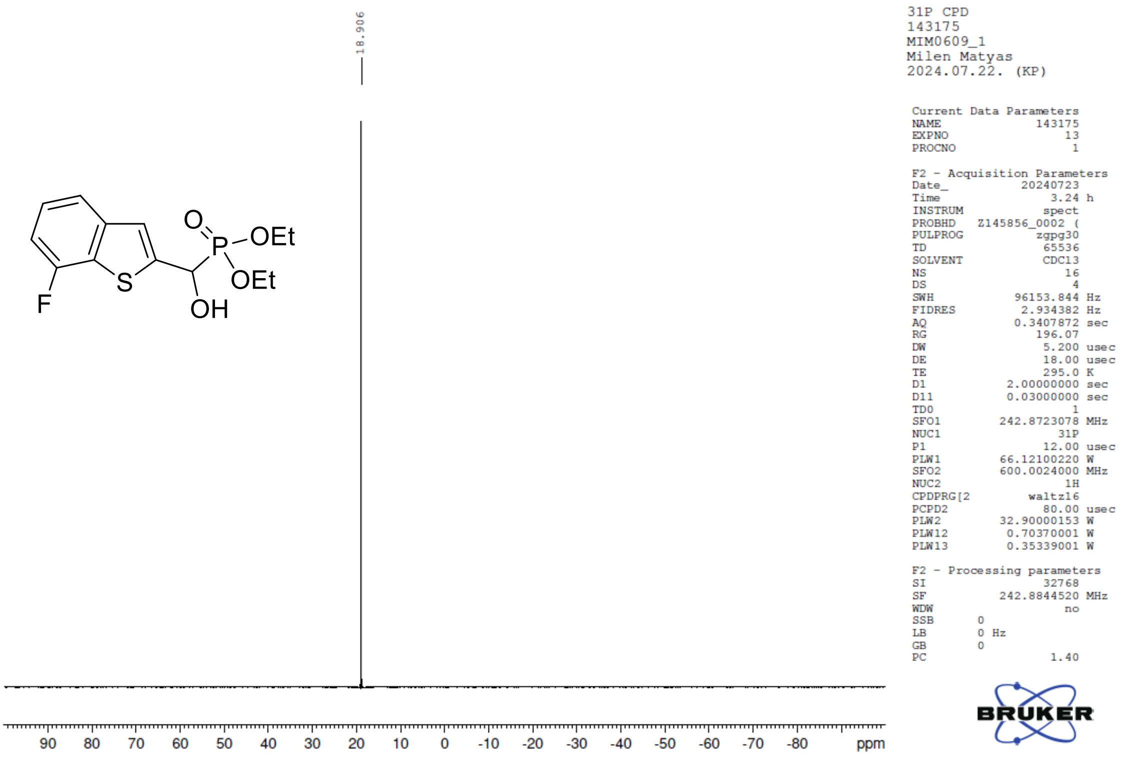

^{13}C NMR (150 MHz, $CDCl_3$) spectra for compound 2c

2c

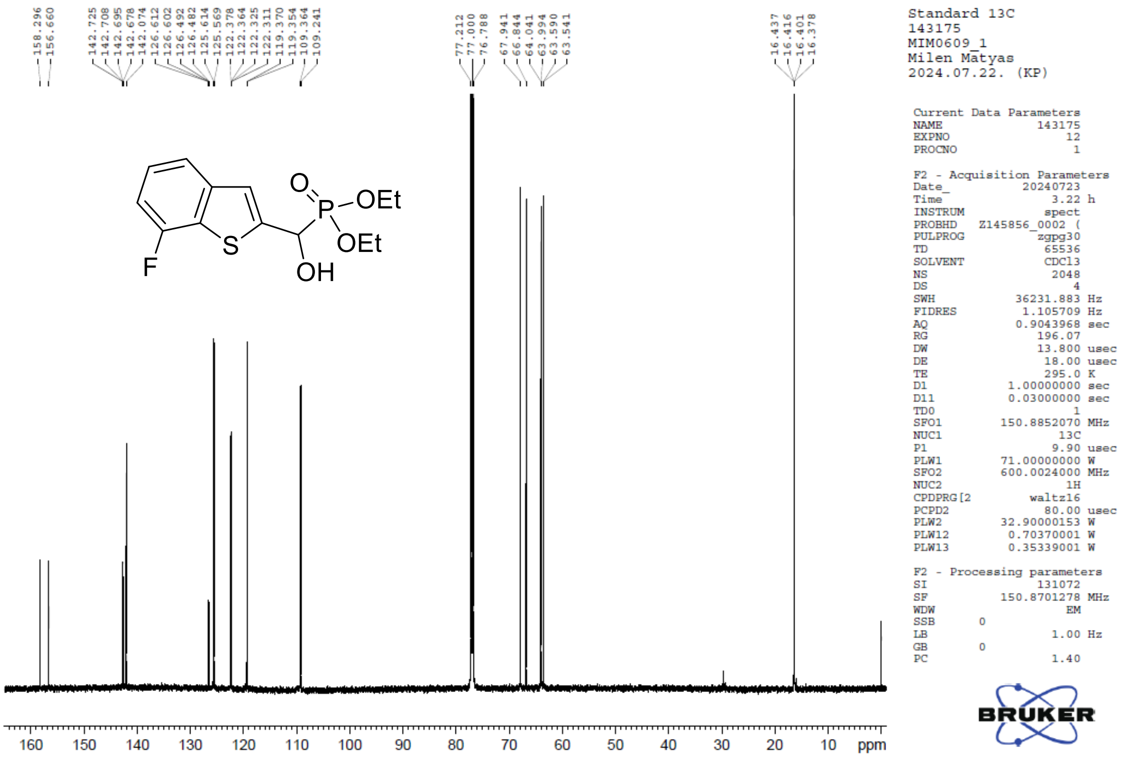

^{1}H NMR (600 MHz, $CDCl_3$) spectra for compound 2c

2c

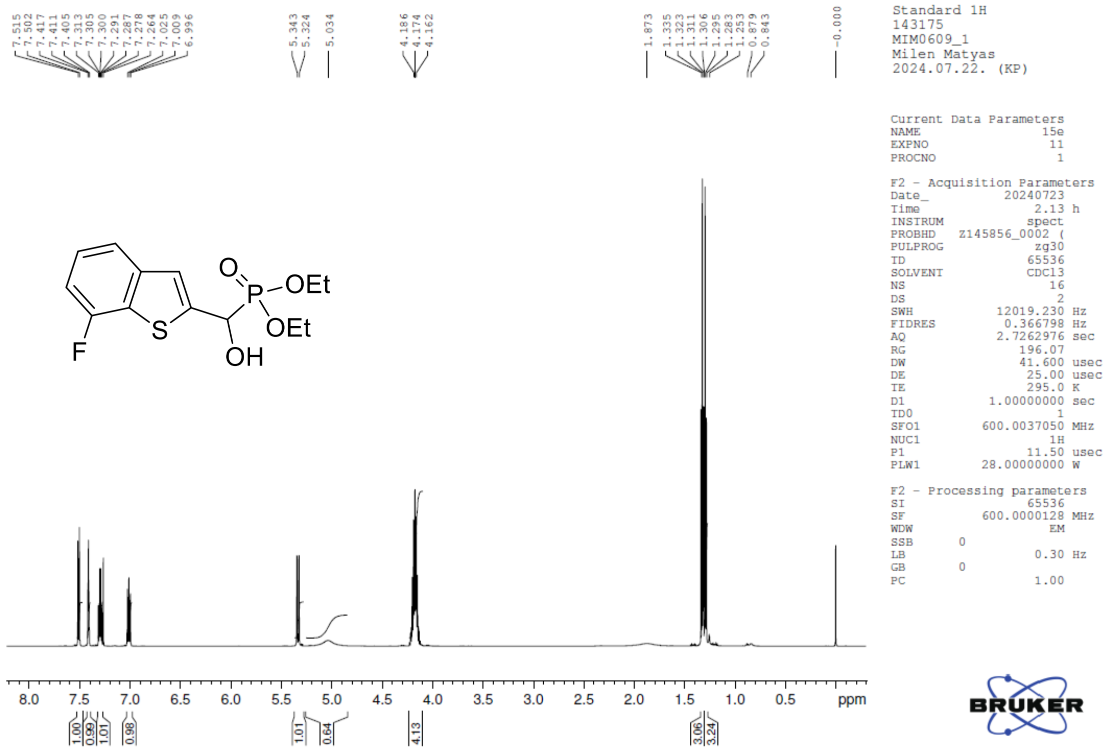

IR (KBr) spectra for compound 2c

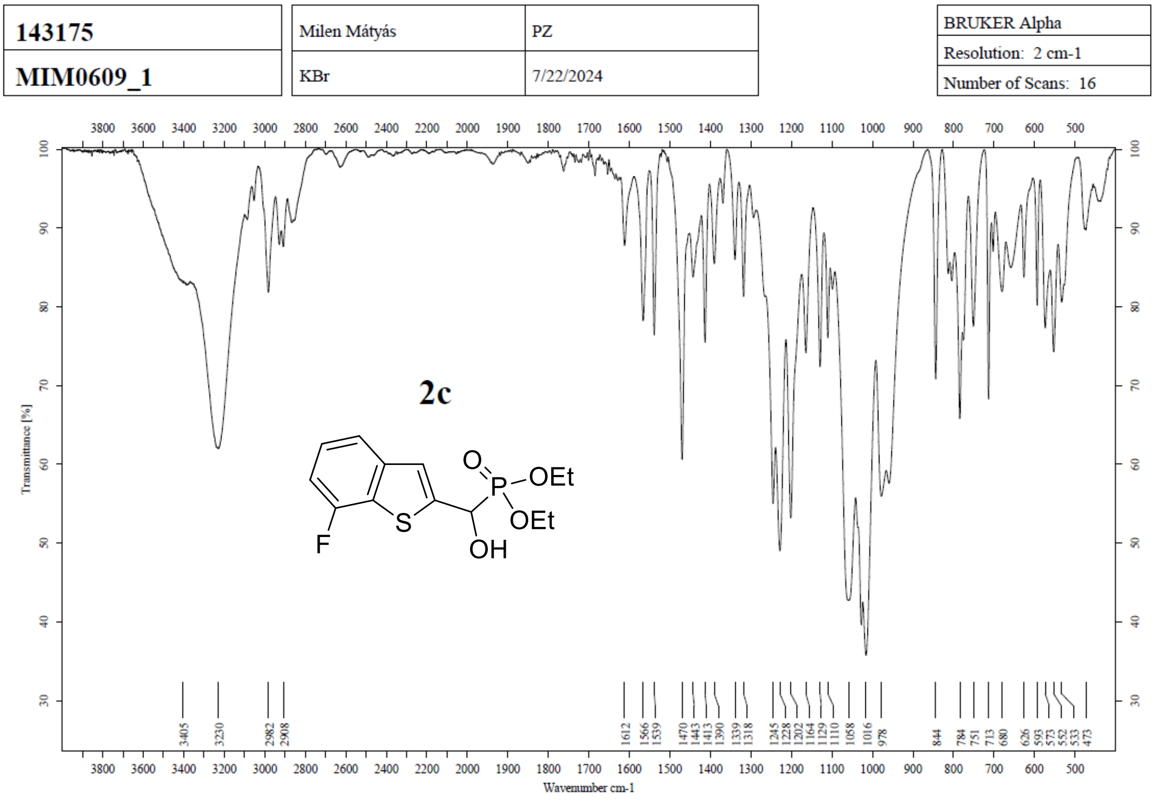

^{31}P NMR (242 MHz, DMSO-d_6) spectra for compound 2d

2d

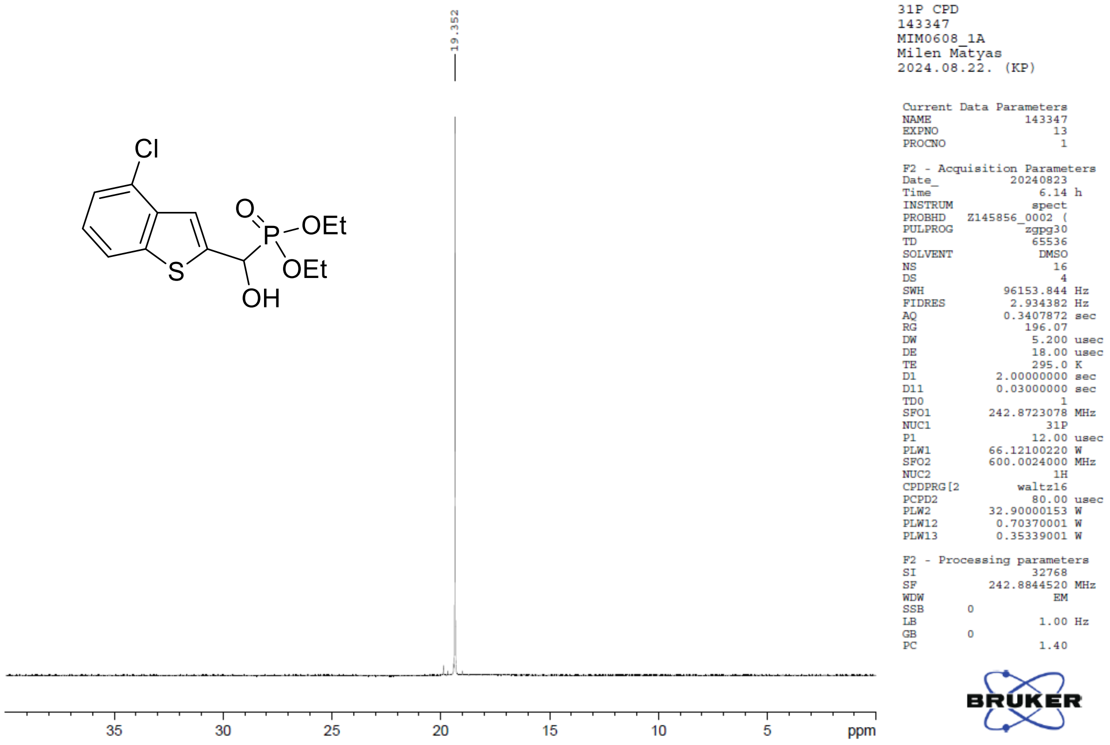

^{13}C NMR (150 MHz, DMSO-d_6) spectra for compound 2d

2d

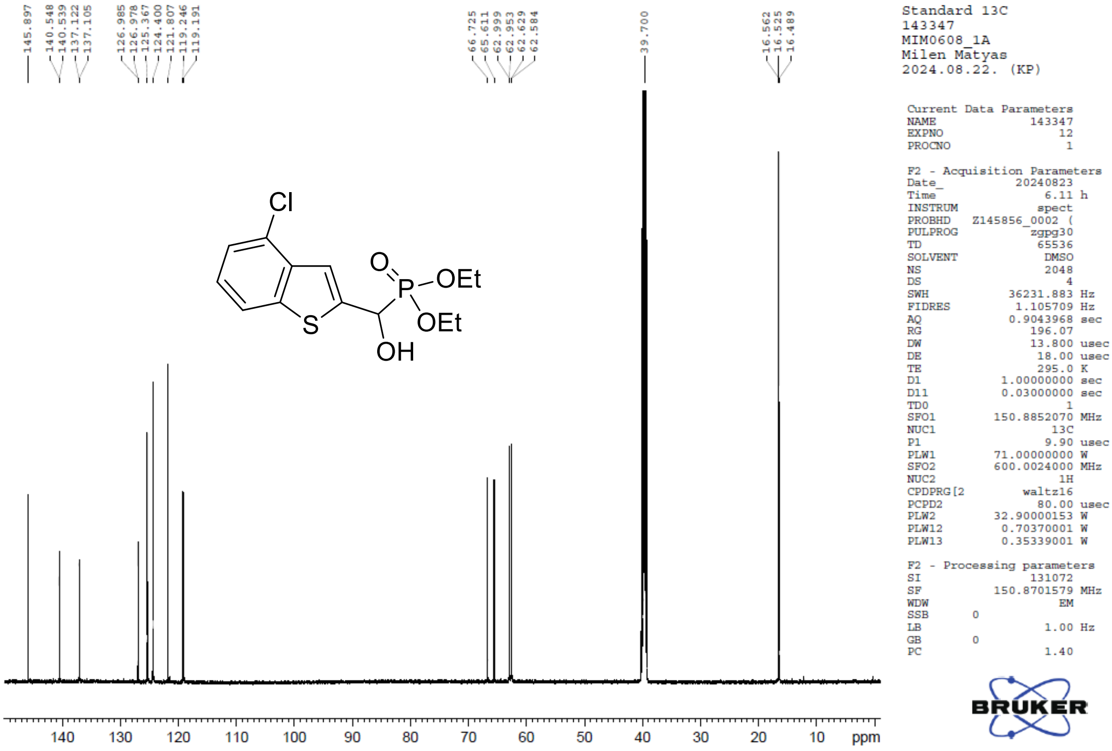

^{1}H NMR (600 MHz, DMSO-d_6) spectra for compound 2d

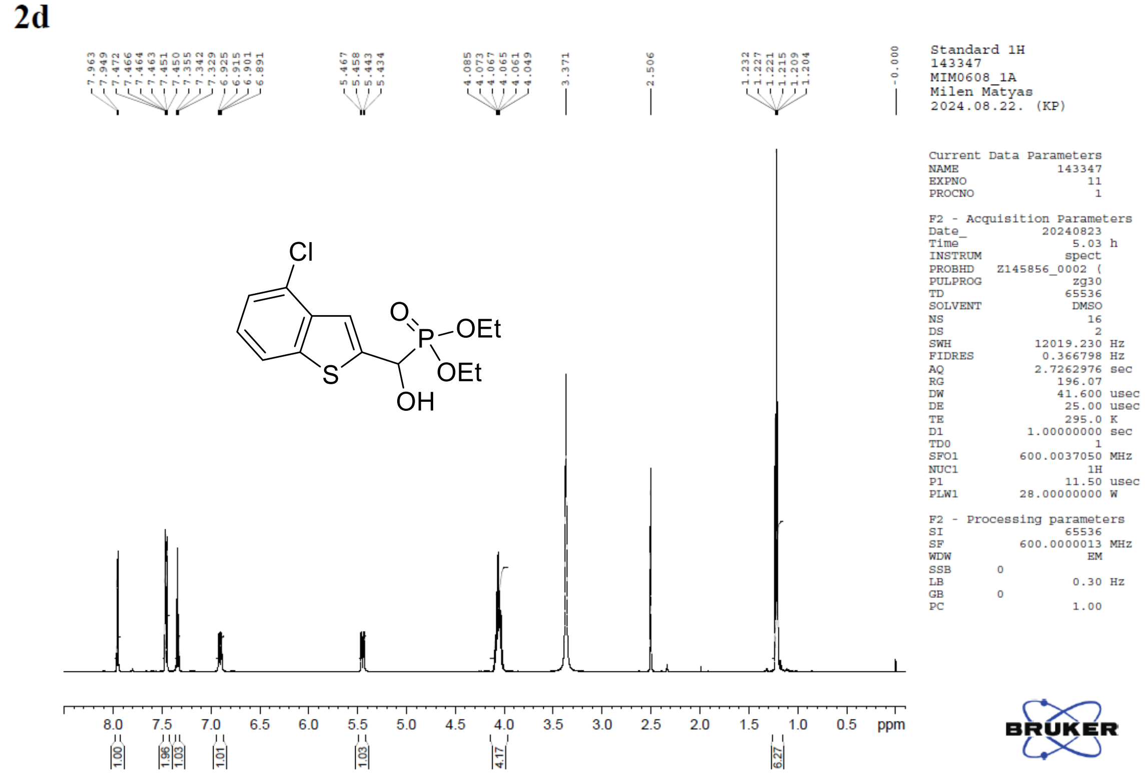

IR (KBr) spectra for compound 2d

143347	Milen Mátyás	HZs
MIM0608_1A	KBr	8/16/2024

BRUKER Alpha
Resolution: 2 cm-1
Number of Scans: 16

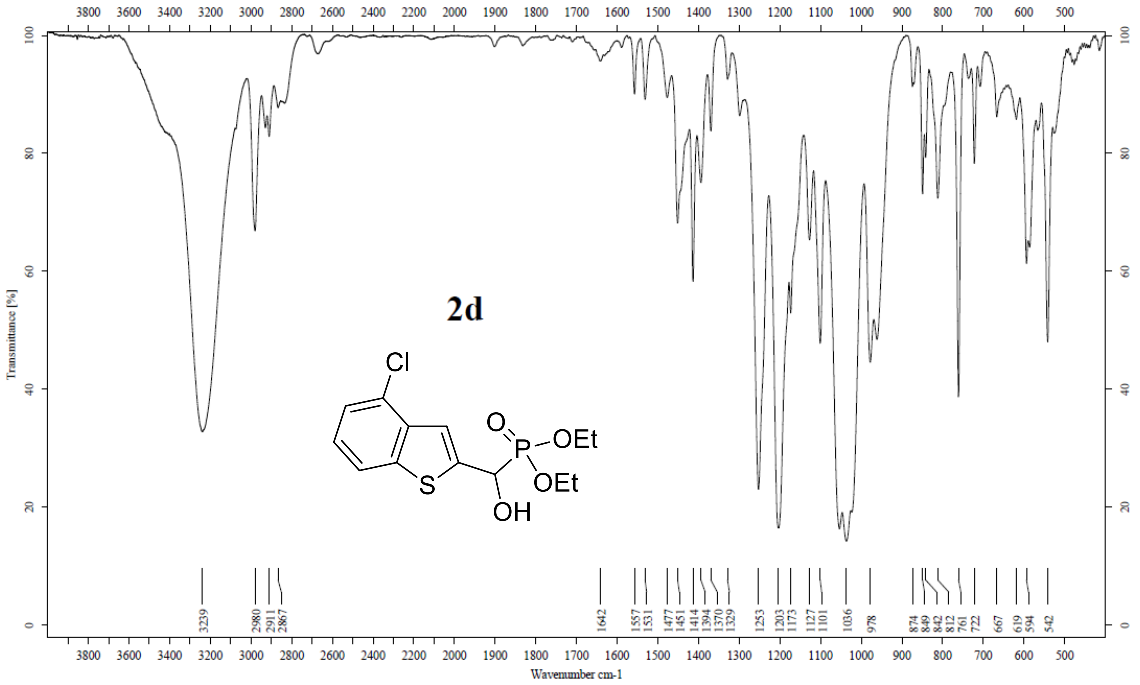

^{31}P NMR (242 MHz, $CDCl_3$) spectra for compound 2e

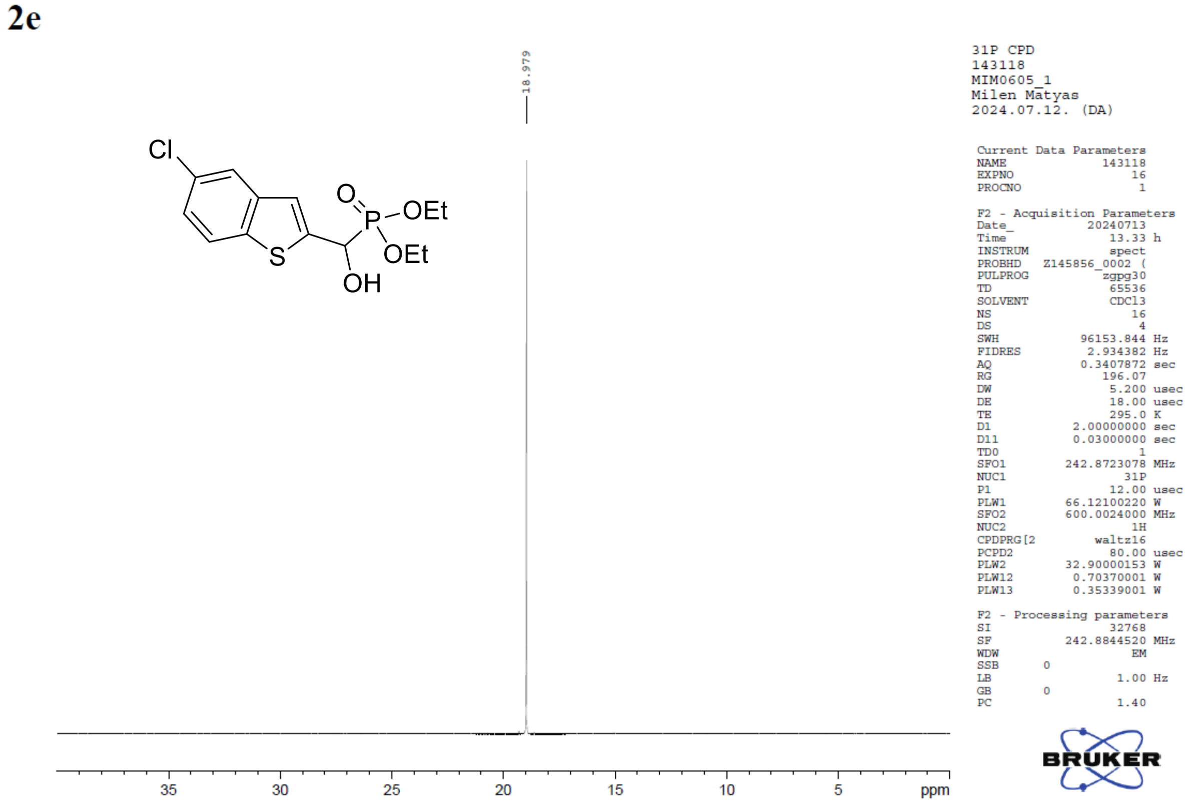

^{13}C NMR (150 MHz, $CDCl_3$) spectra for compound 2e

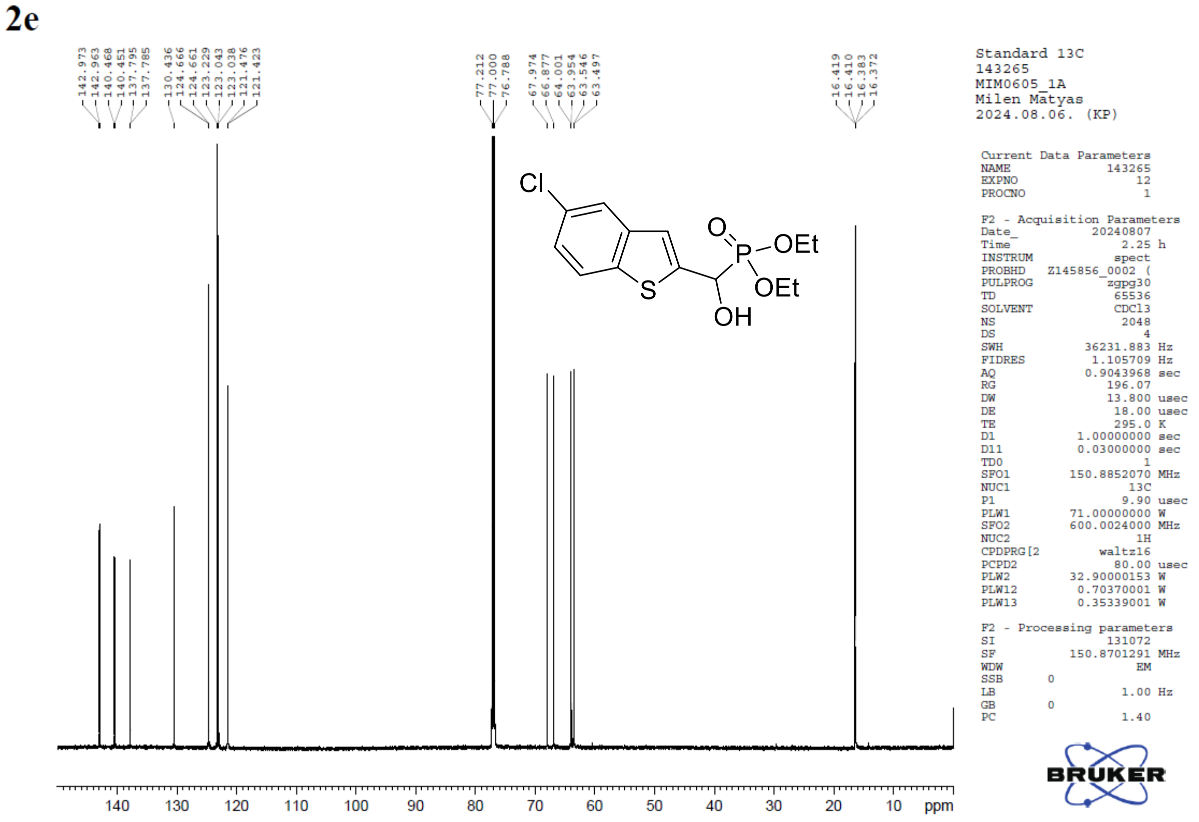

^{1}H NMR (600 MHz, $CDCl_3$) spectra for compound 2e

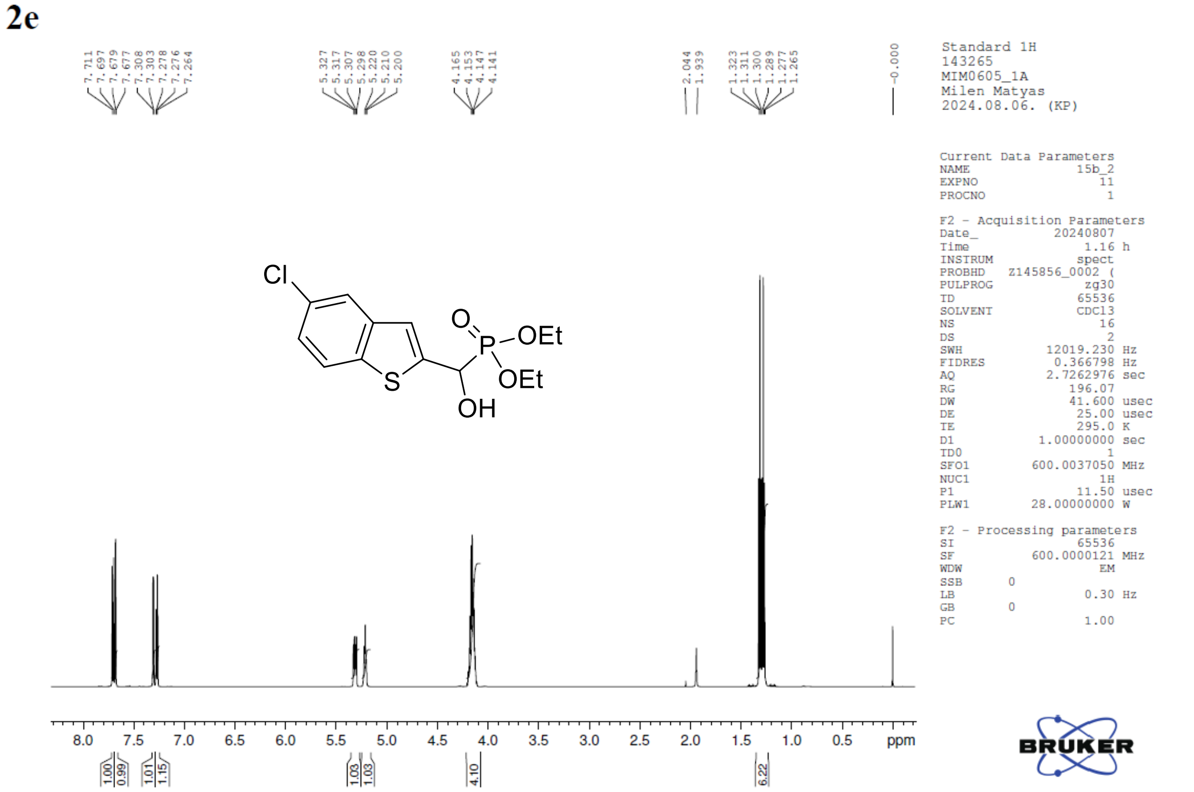

IR (KBr) spectra for compound 2e

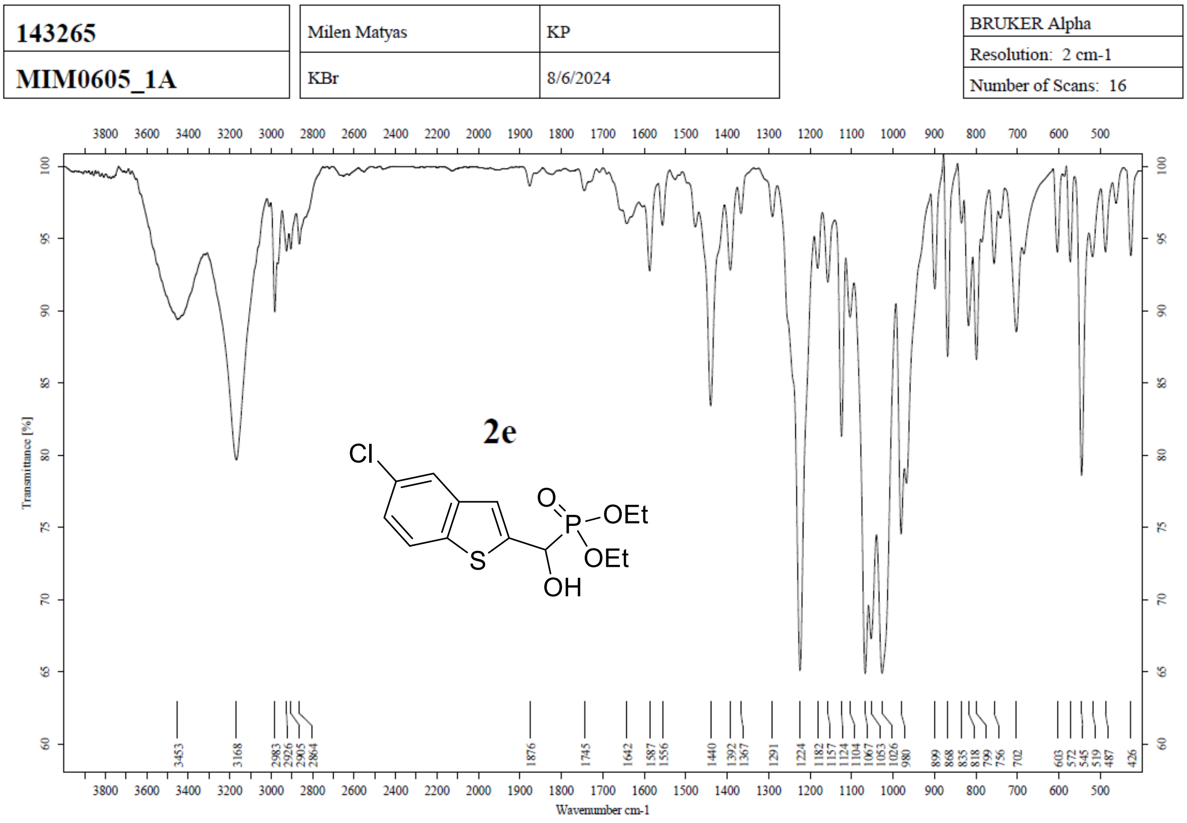

^{31}P NMR (242 MHz, $CDCl_3$) spectra for compound 2f

2f

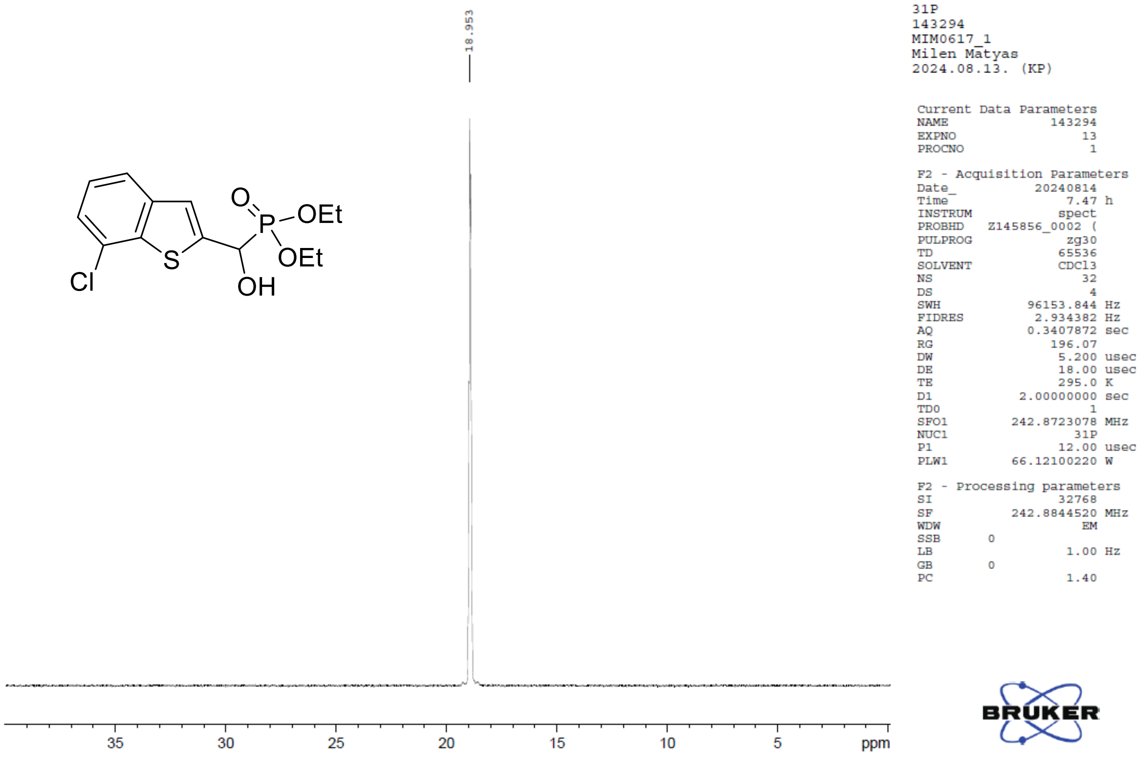

^{13}C NMR (150 MHz, $CDCl_3$) spectra for compound 2f

2f

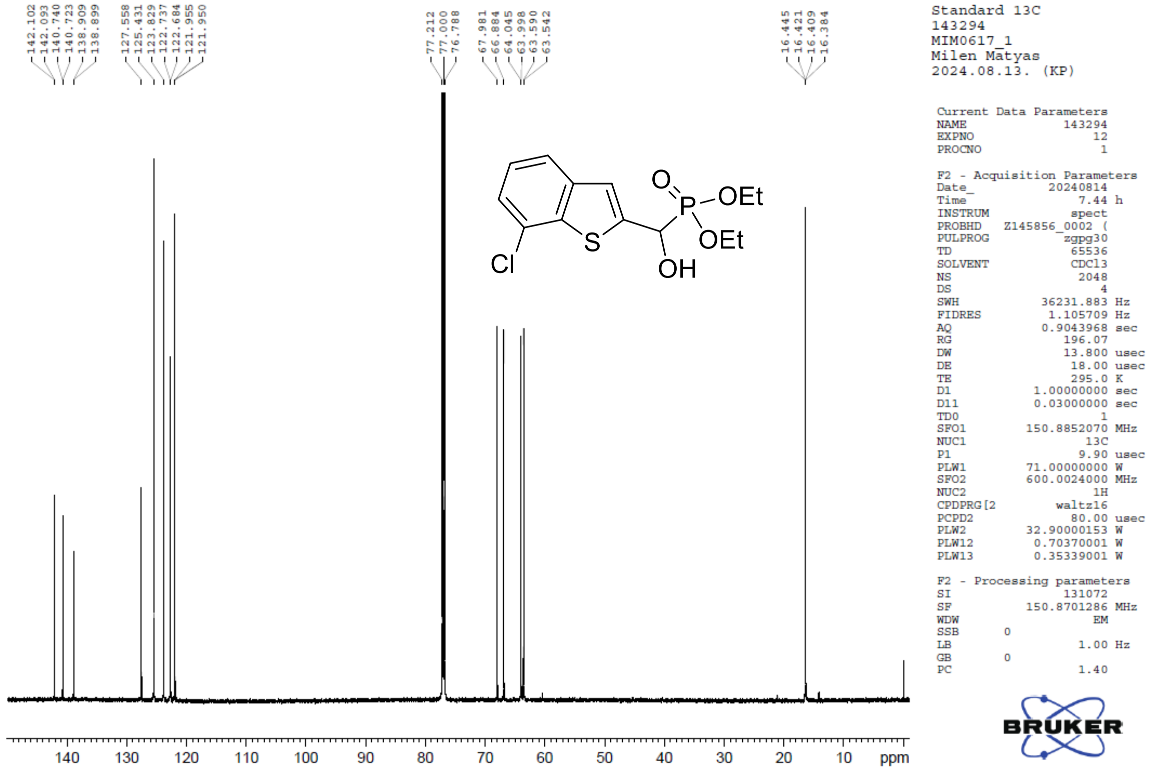

^{1}H NMR (600 MHz, $CDCl_3$) spectra for compound 2f

2f

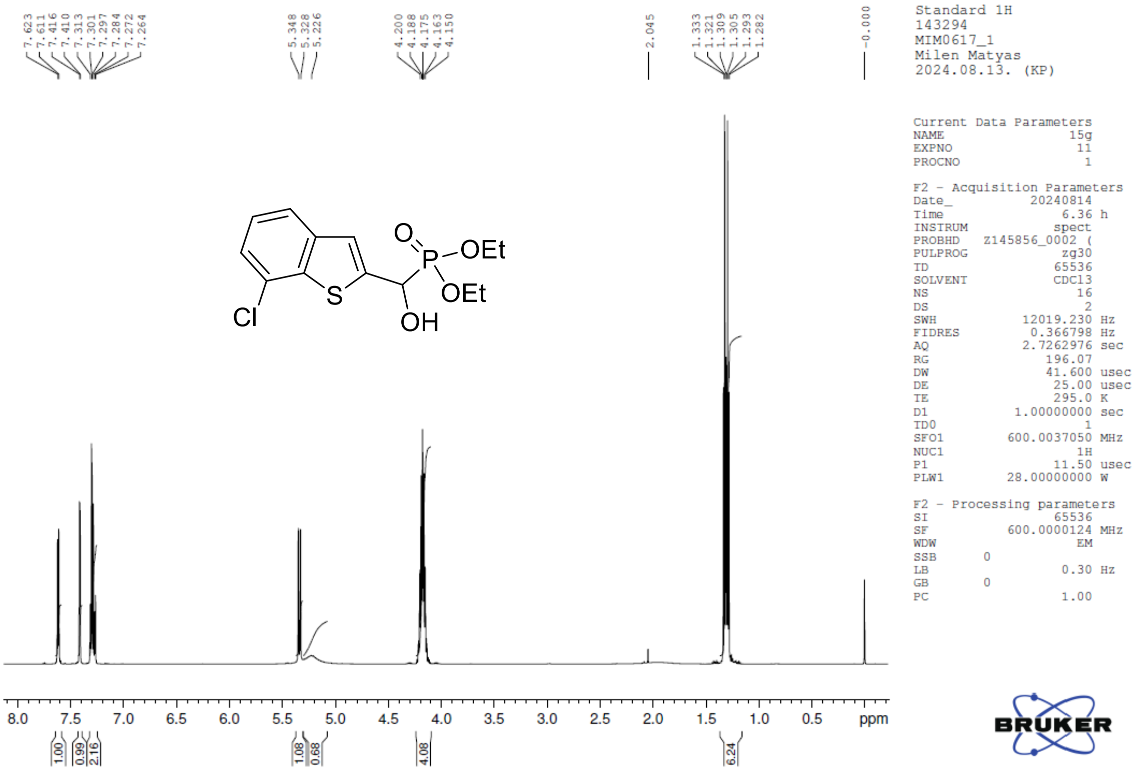

IR (KBr) spectra for compound 2f

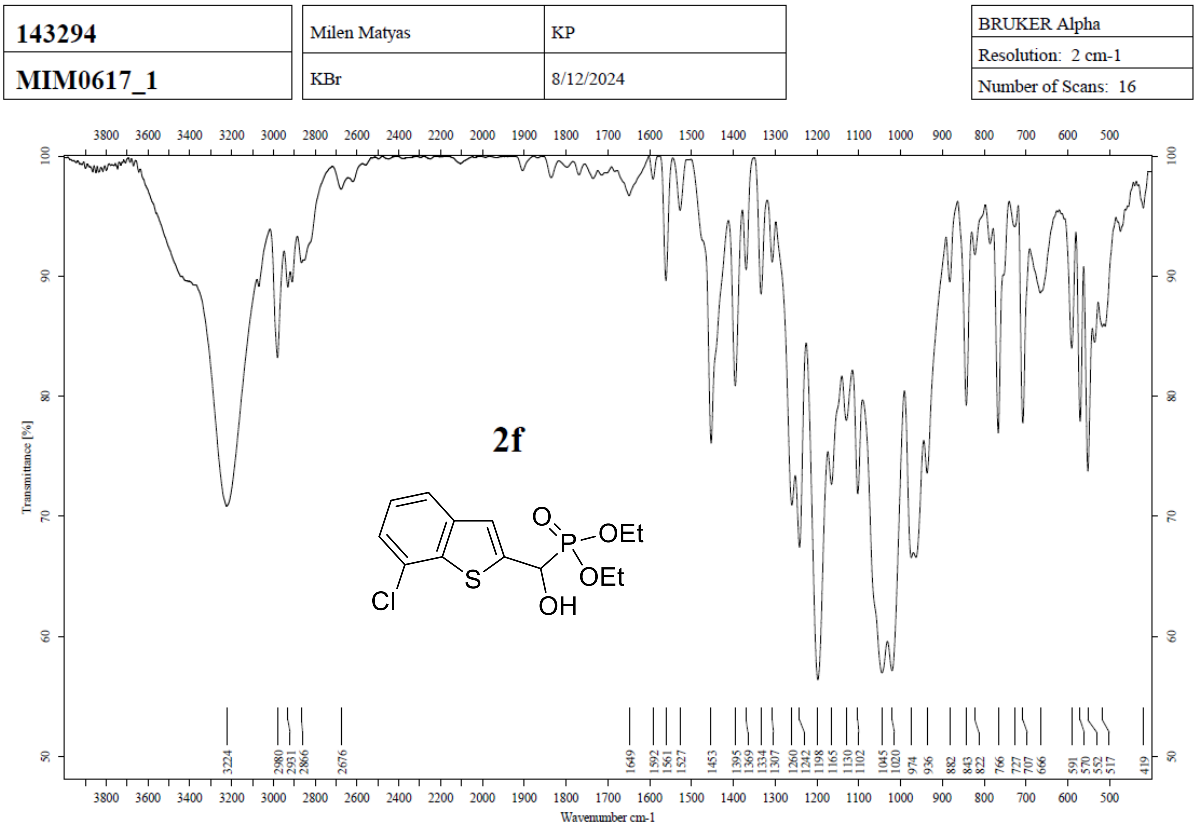

^{31}P NMR (242 MHz, $CDCl_3$) spectra for compound 2g

2g

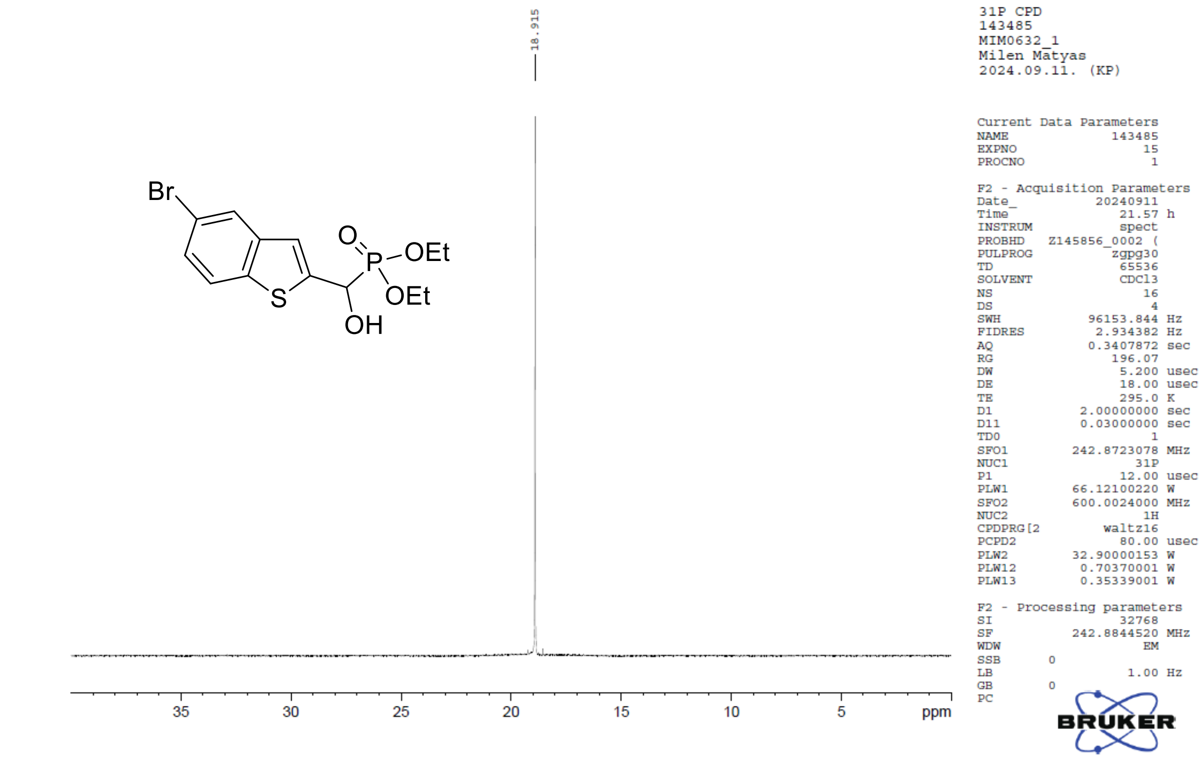

^{13}C NMR (150 MHz, $CDCl_3$) spectra for compound 2g

2g

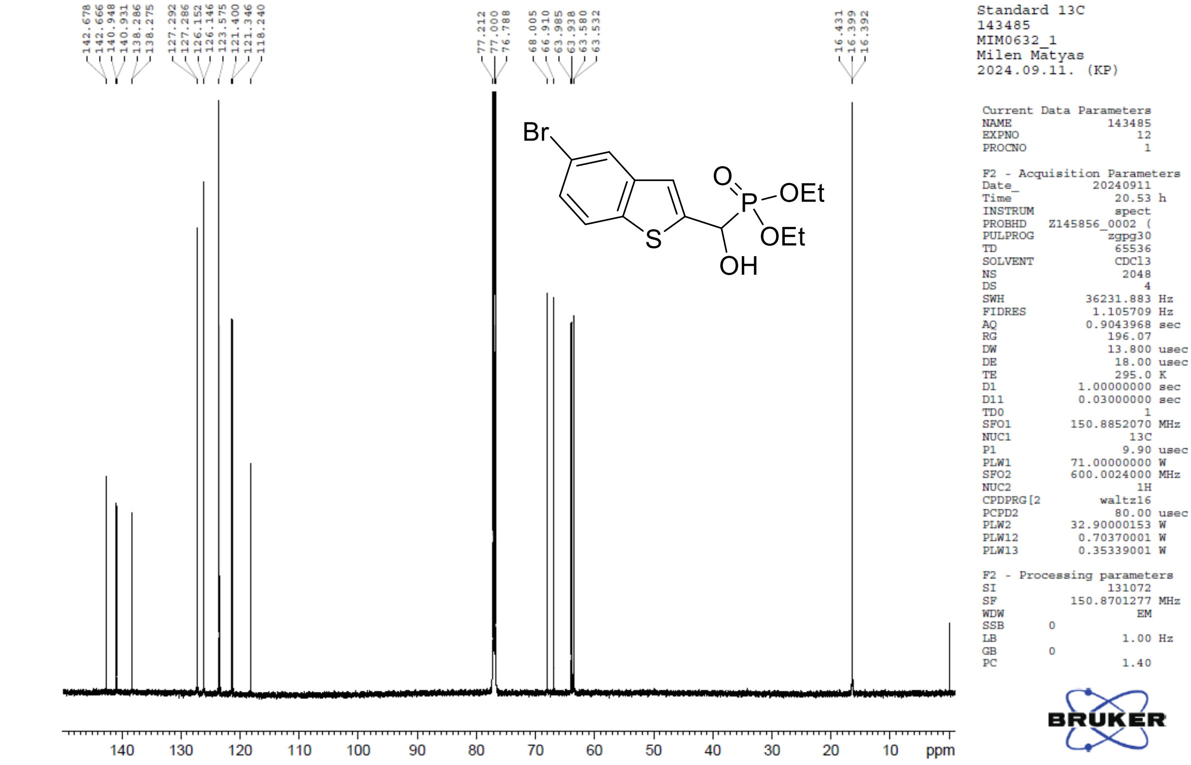

^{1}H NMR (600 MHz, $CDCl_3$) spectra for compound 2g

2g

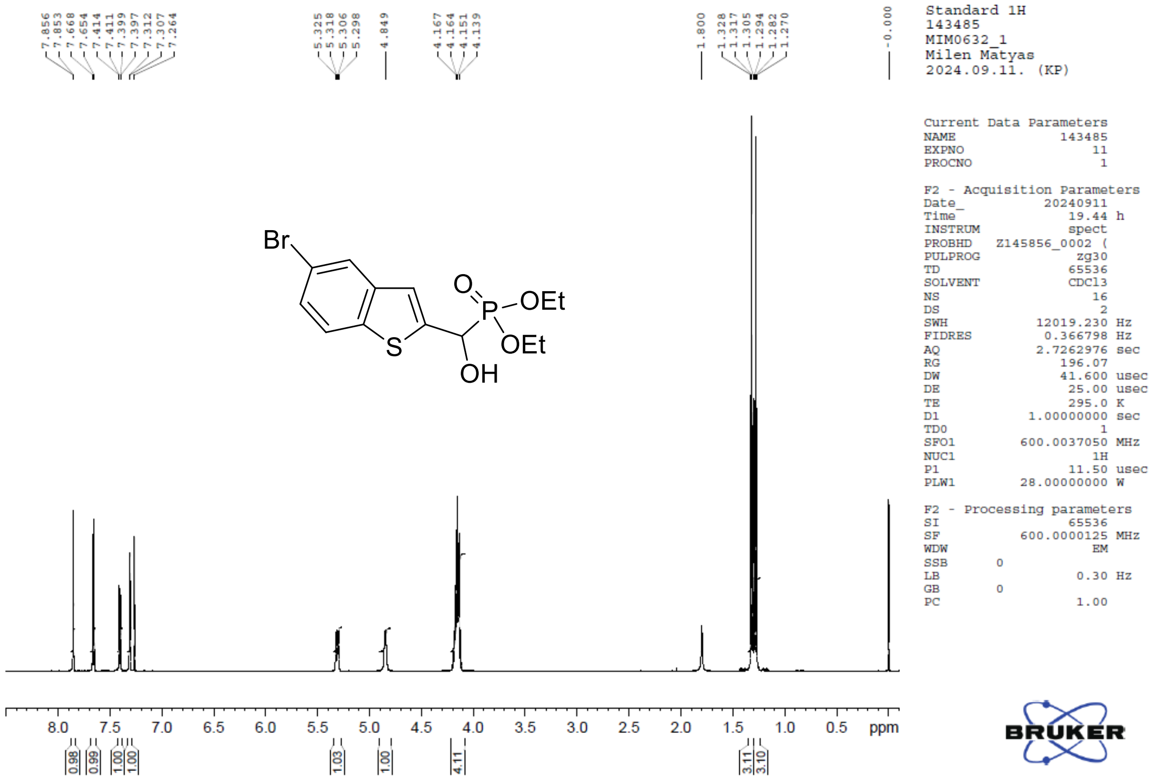

IR (KBr) spectra for compound 2g

143485	Milen Matyas	KP		BRUKER Alpha
MIM0632_1	KBr	9/11/2024		Resolution: 2 cm-1
				Number of Scans: 16

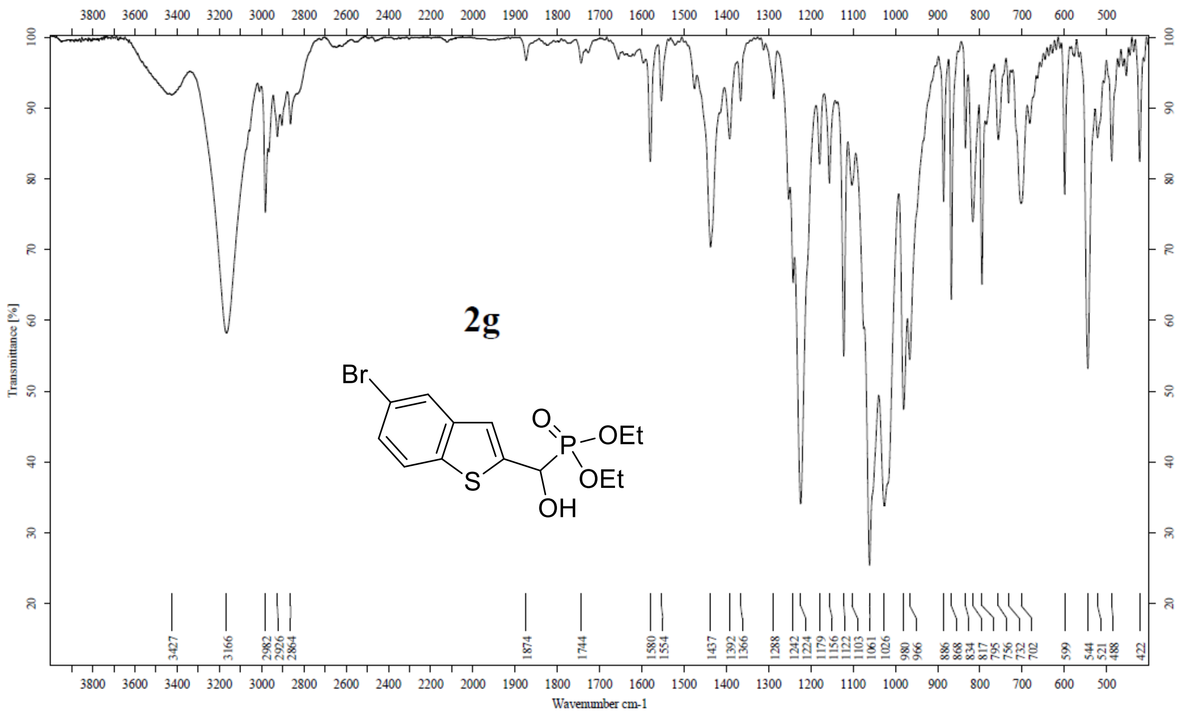

^{31}P NMR (242 MHz, $CDCl_3$) spectra for compound 2h

2h

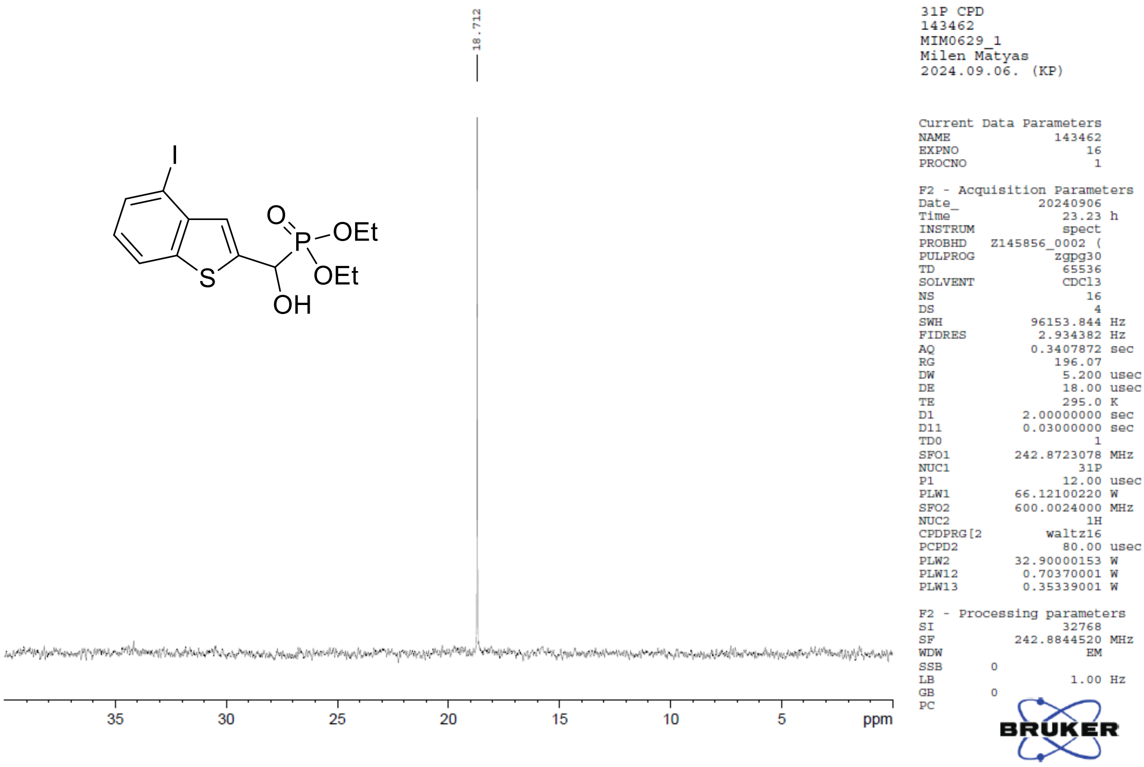

^{13}C NMR (150 MHz, $CDCl_3$) spectra for compound 2h

2h

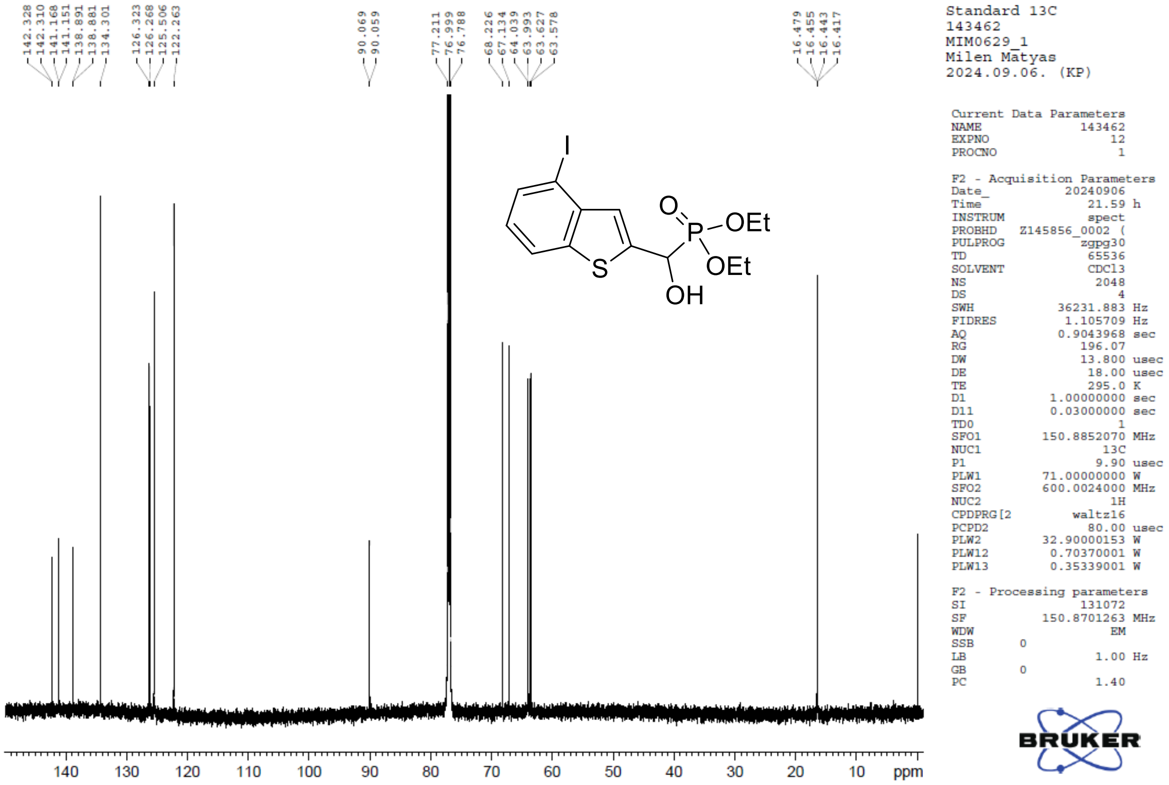

^{1}H NMR (600 MHz, $CDCl_3$) spectra for compound 2h

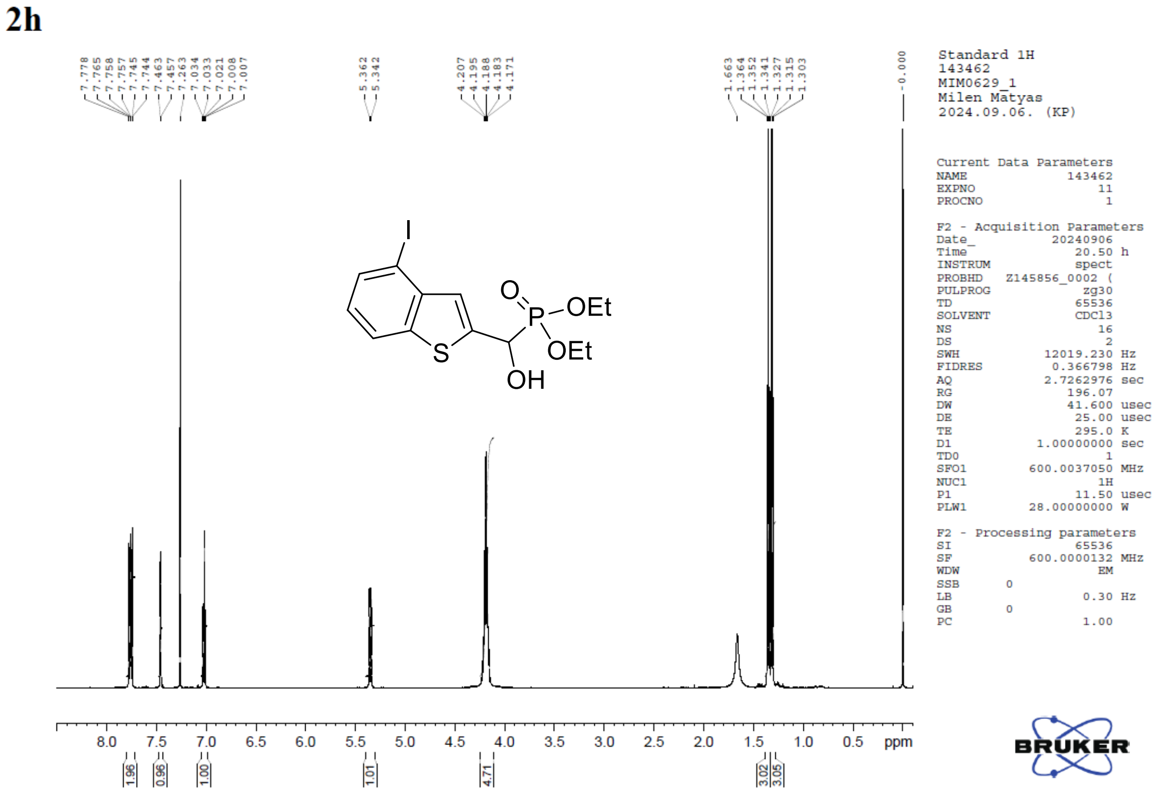

IR (KBr) spectra for compound 2h

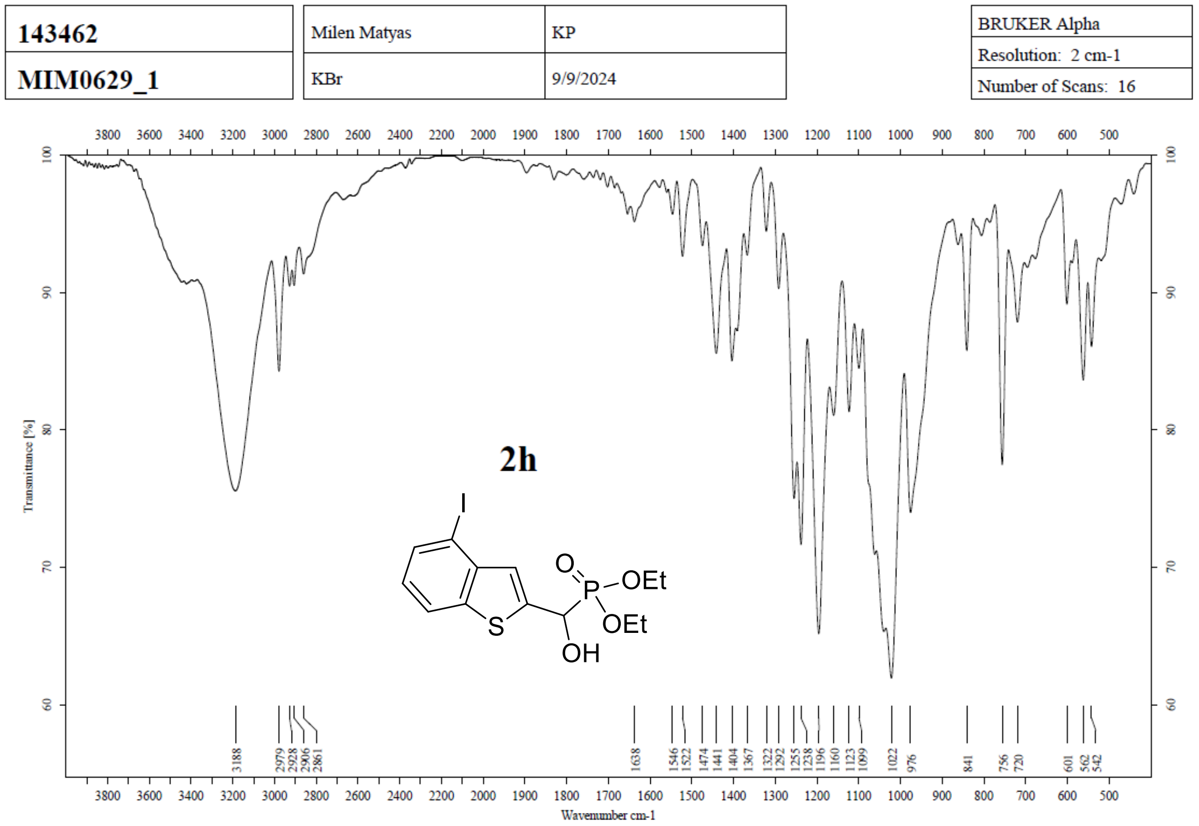

^{31}P NMR (242 MHz, $CDCl_3$) spectra for compound 2i

2i

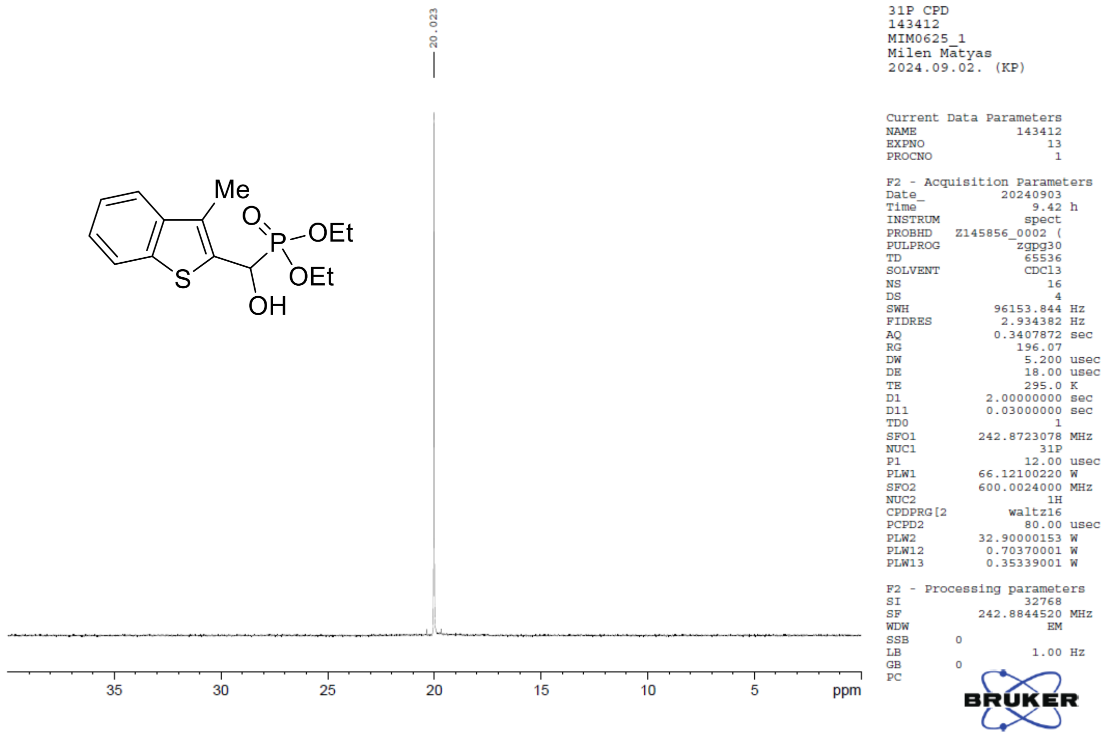

^{13}C NMR (150 MHz, $CDCl_3$) spectra for compound 2i

2i

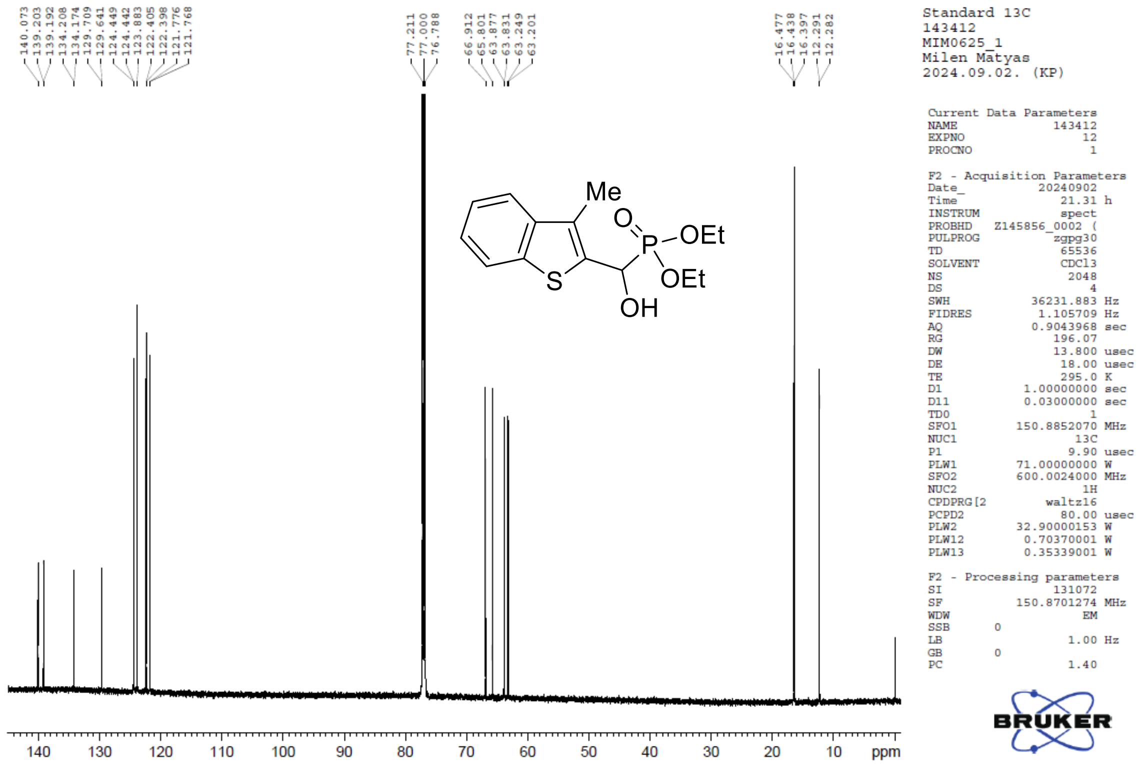

^{1}H NMR (600 MHz, $CDCl_3$) spectra for compound 2i

2i

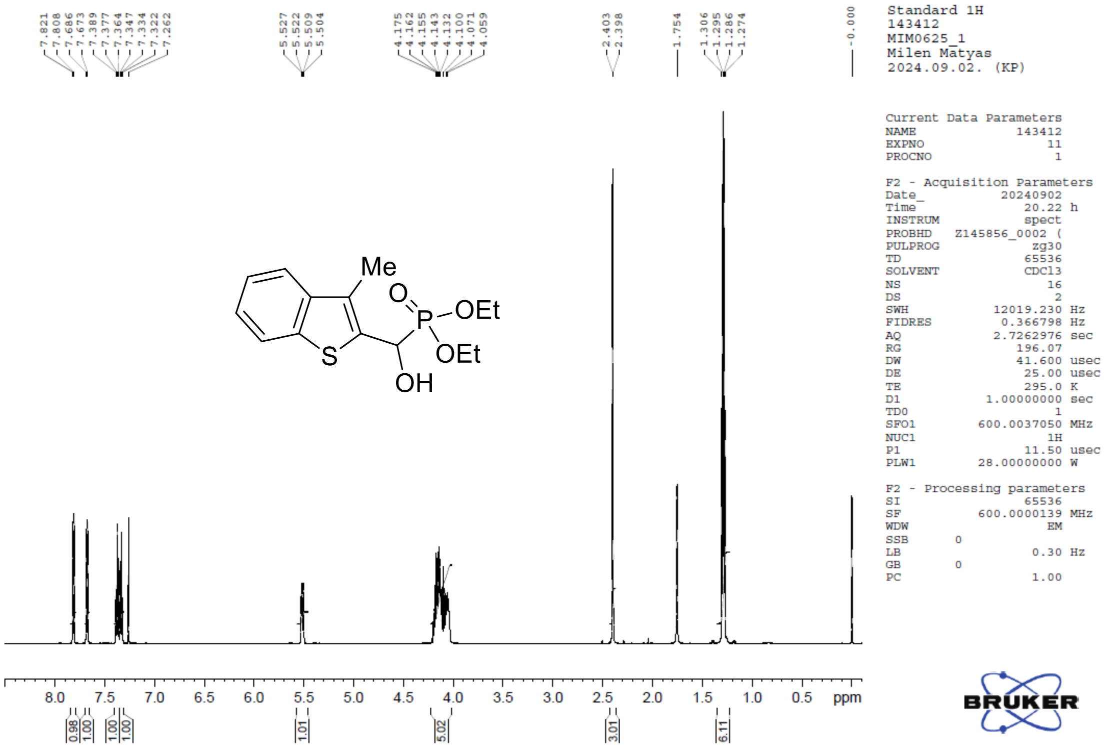

IR (KBr) spectra for compound 2i

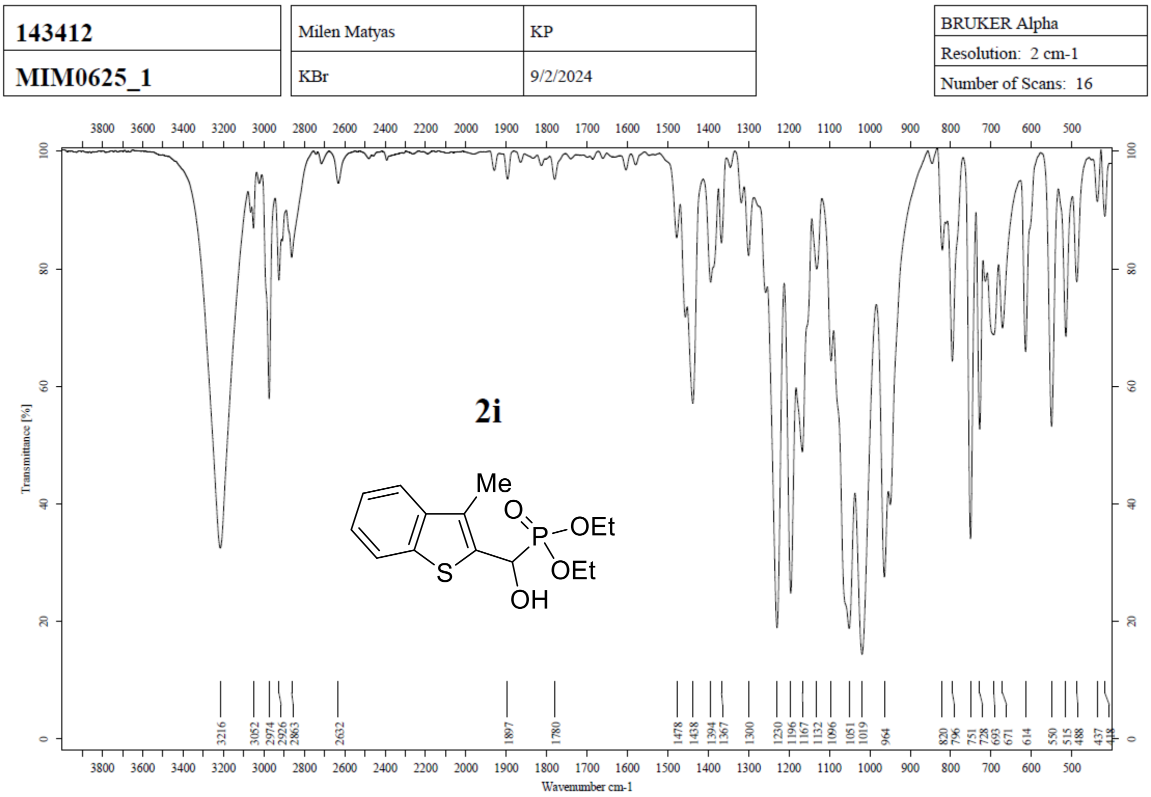

^{31}P NMR (242 MHz, CDCl$_3$) spectra for compound 2j

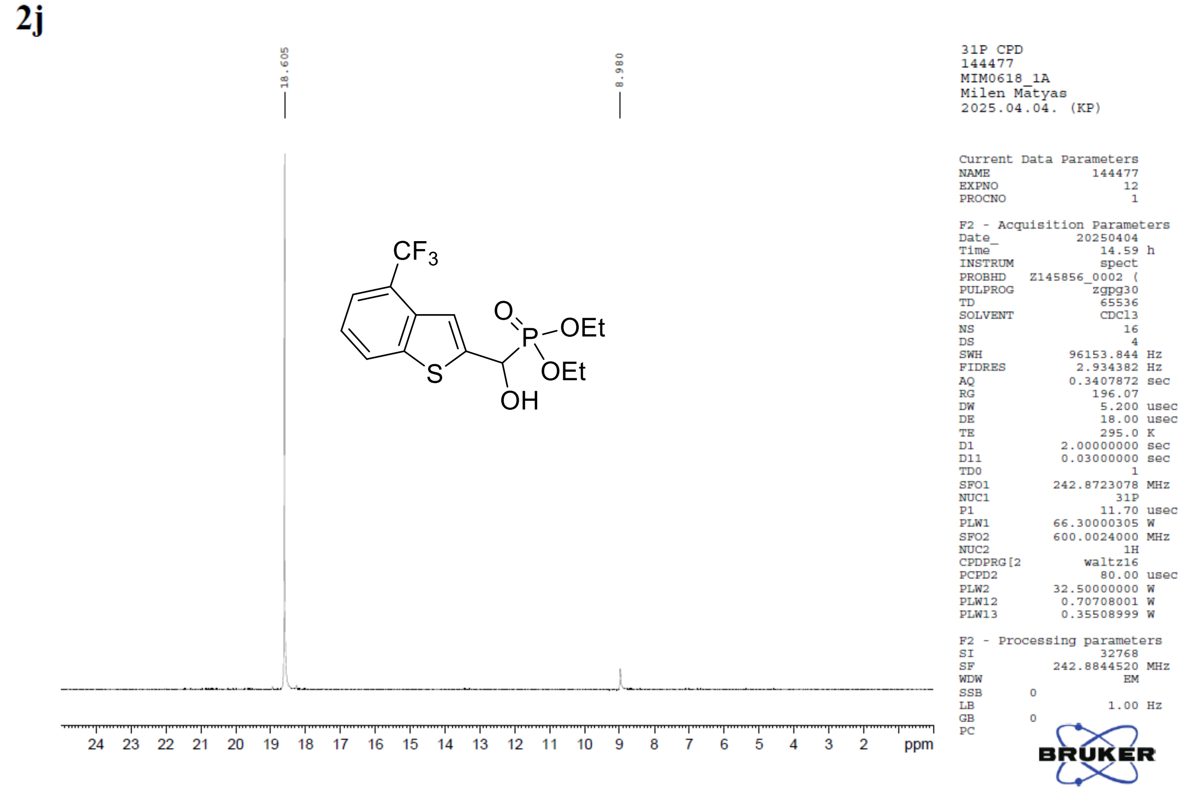

^{13}C NMR (150 MHz, CDCl$_3$) spectra for compound 2j

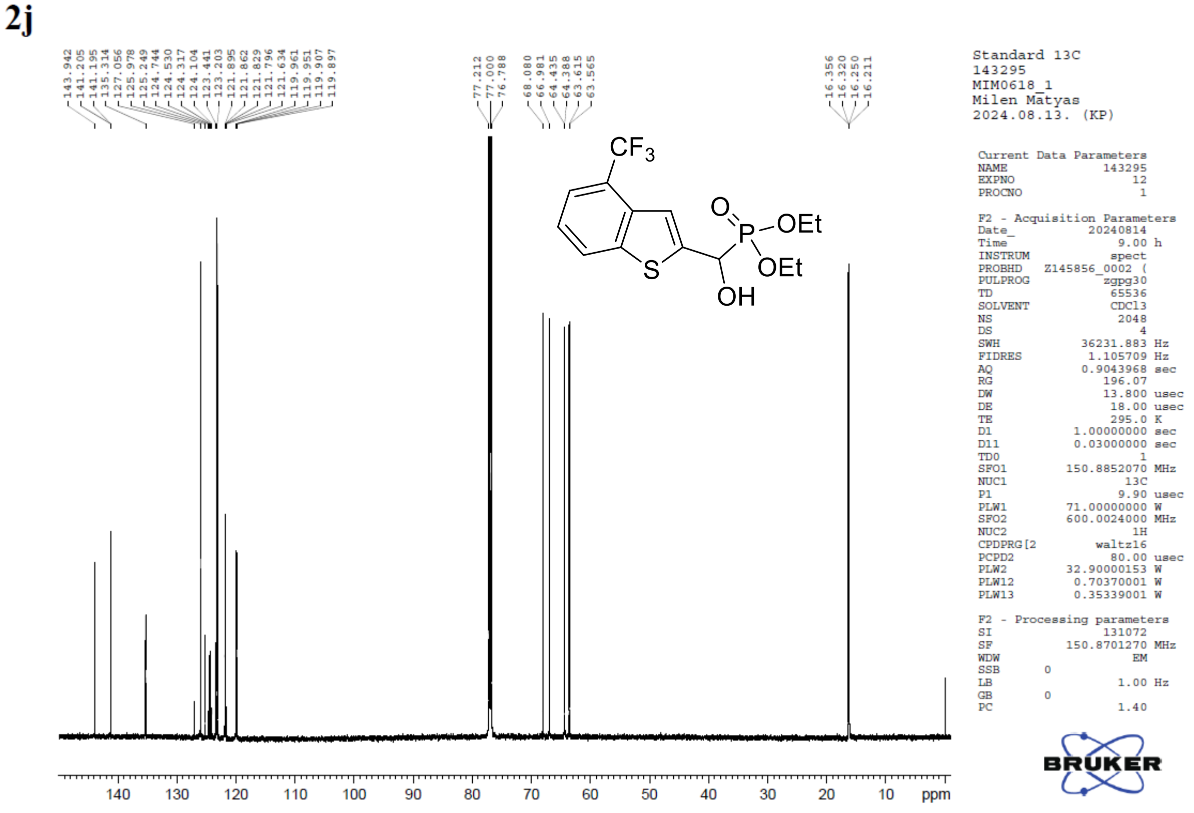

^{1}H NMR (600 MHz, $CDCl_3$) spectra for compound 2j

2j

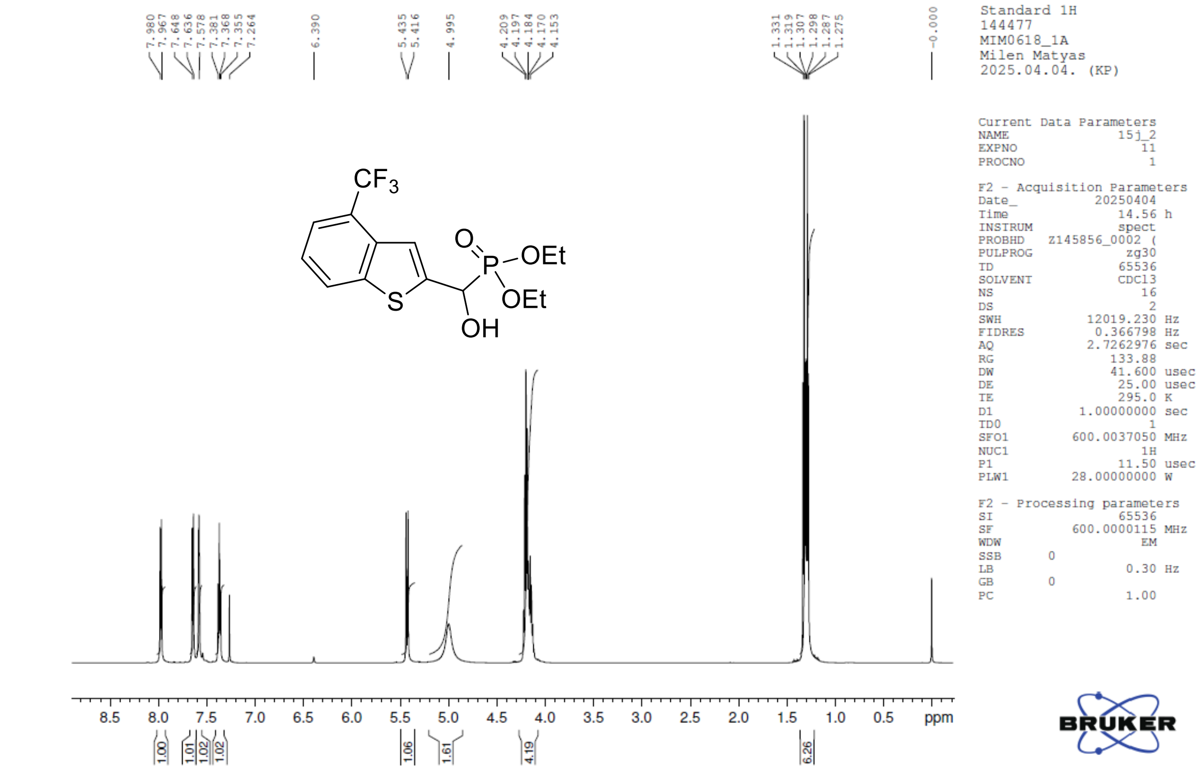

IR (IR) spectra for compound 2j

**143295**	Milen Matyas	KP		BRUKER Alpha
**MIM0618_1**	KBr	8/12/2024		Resolution: 2 cm-1
				Number of Scans: 16

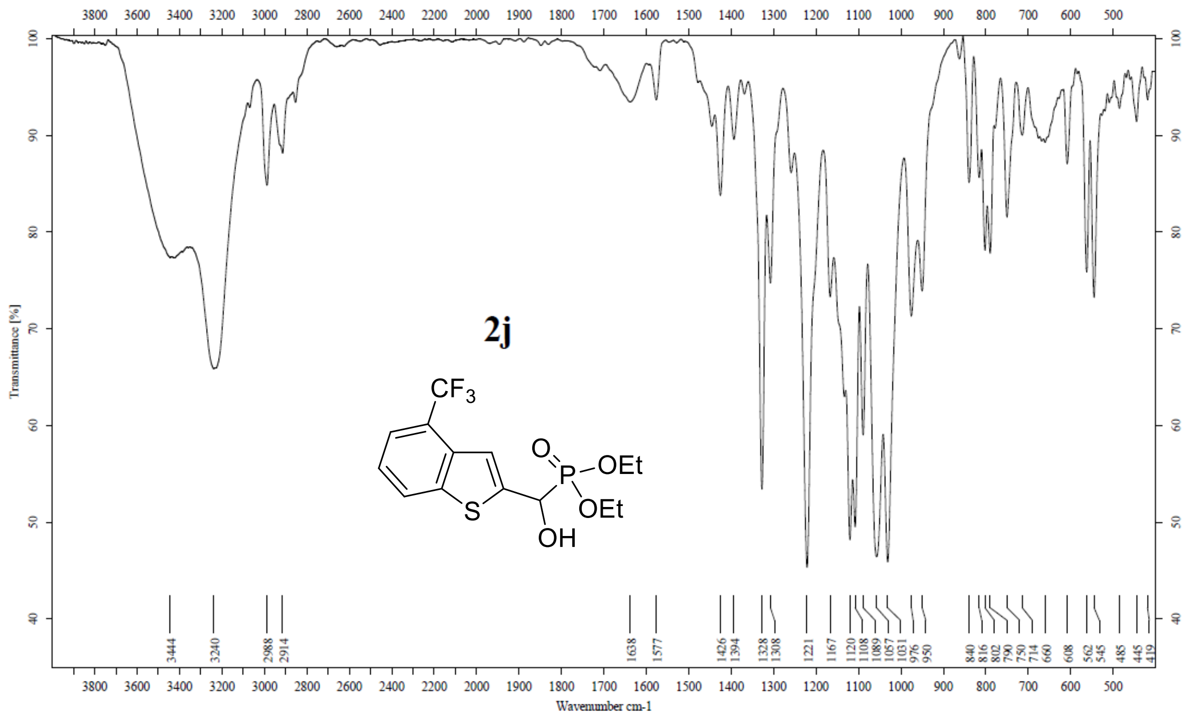

^{31}P NMR (242 MHz, $CDCl_3$) spectra for compound 2k

2k

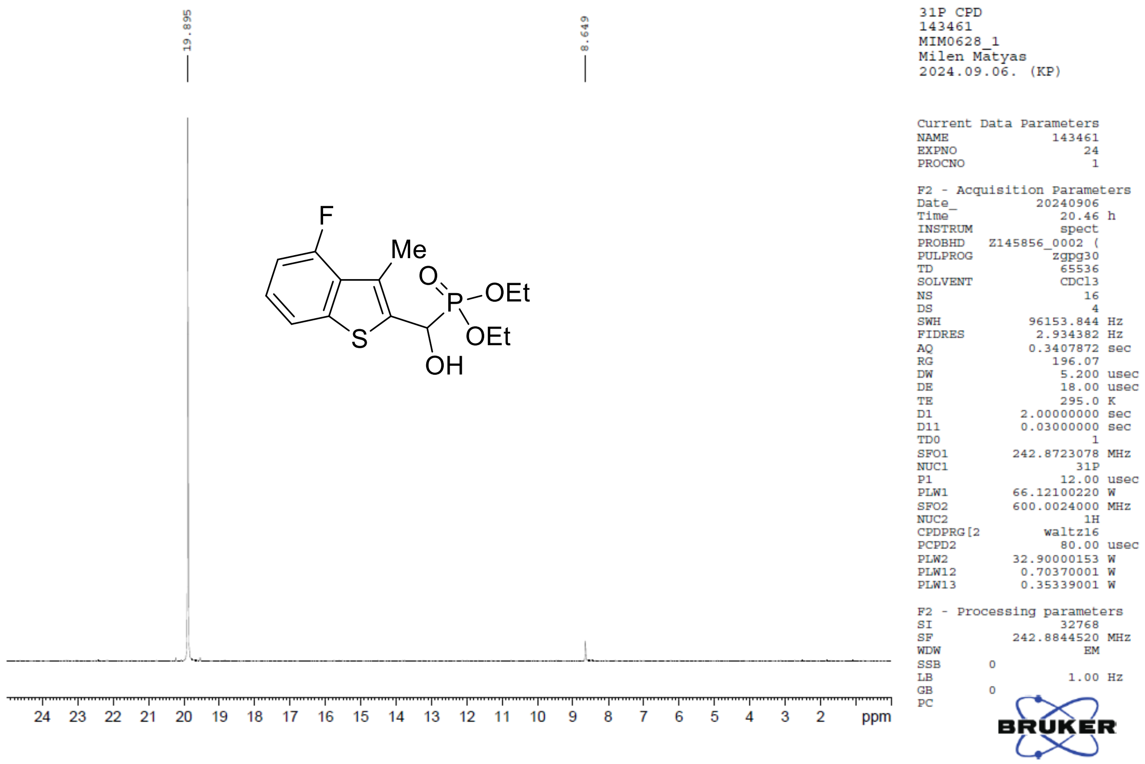

^{13}C NMR (150 MHz, $CDCl_3$) spectra for compound 2k

2k

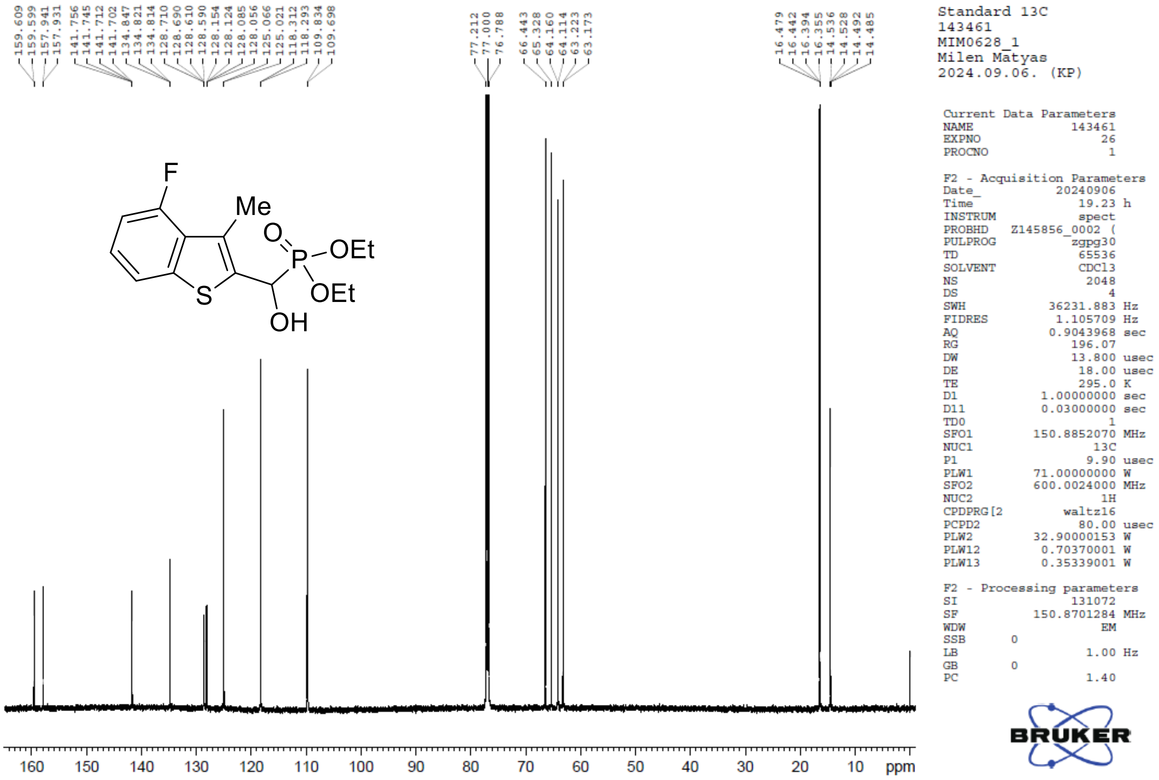

^{1}H NMR (600 MHz, $CDCl_3$) spectra for compound 2k

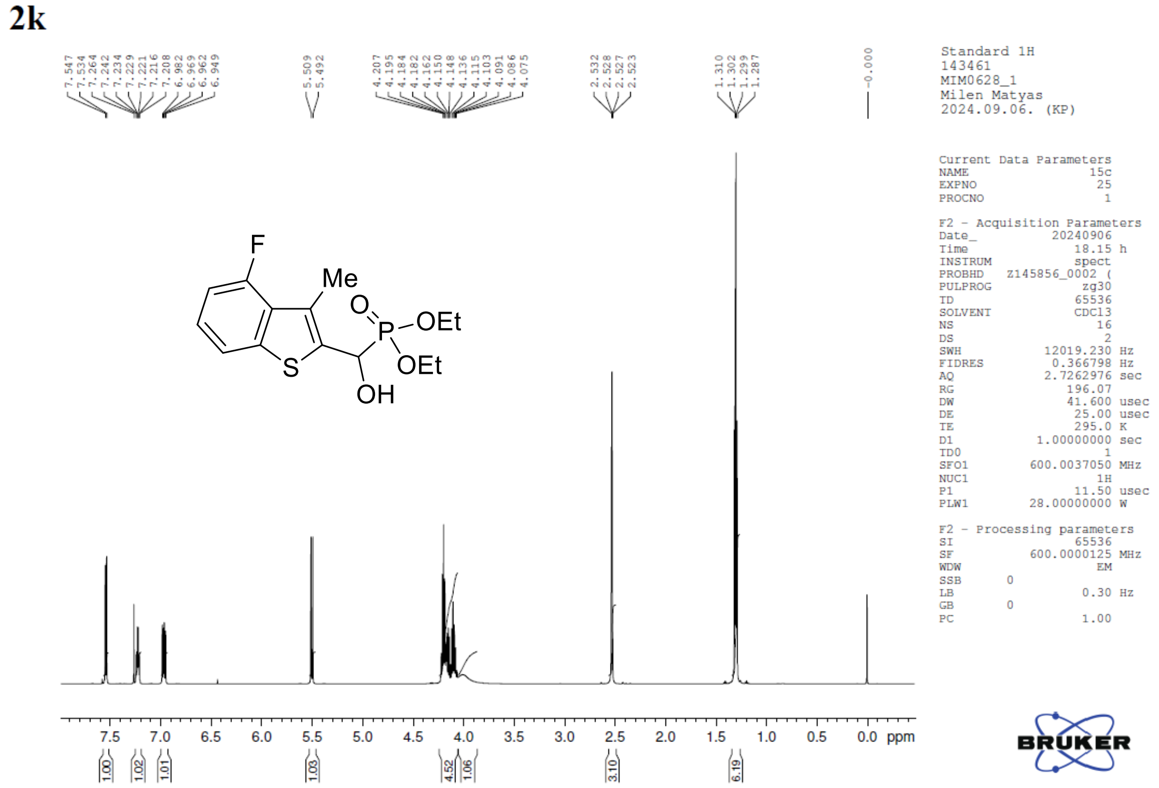

IR (KBr) spectra for compound 2k

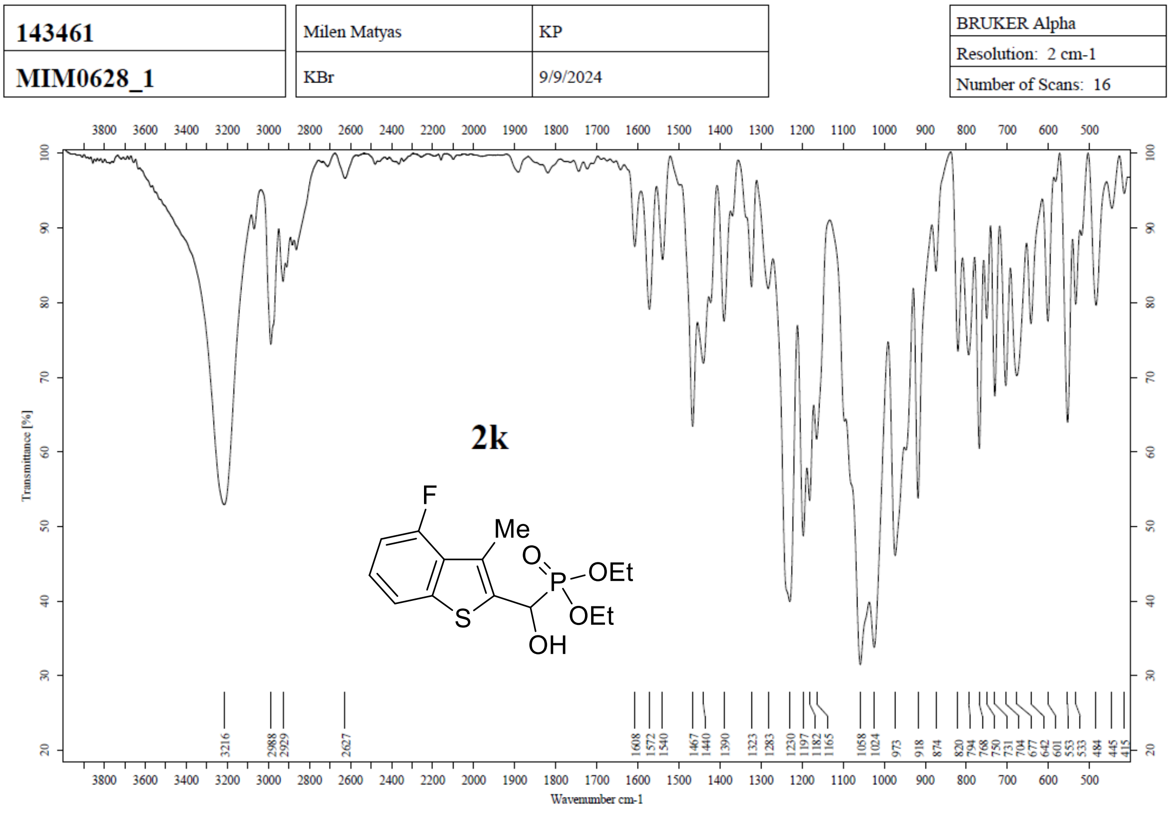

^{31}P NMR (242 MHz, $CDCl_3$) spectra for compound 2l

2l

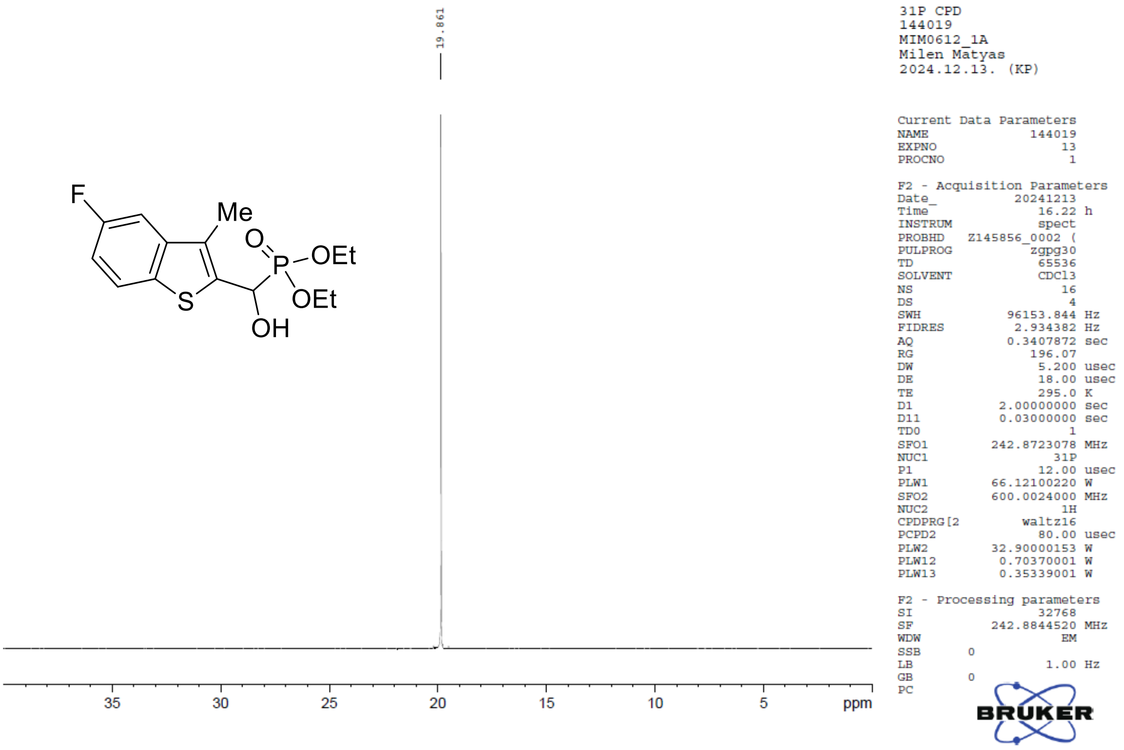

^{13}C NMR (150 MHz, $CDCl_3$) spectra for compound 2l

2l

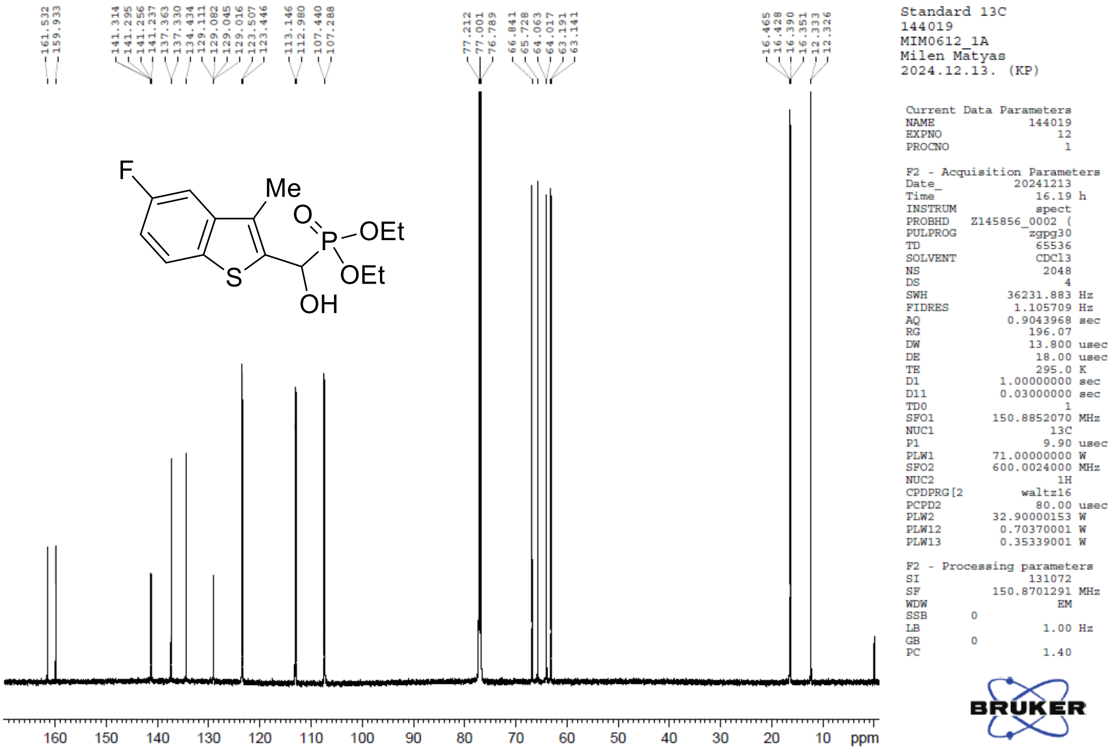

^{1}H NMR (600 MHz, $CDCl_3$) spectra for compound 2l

2l

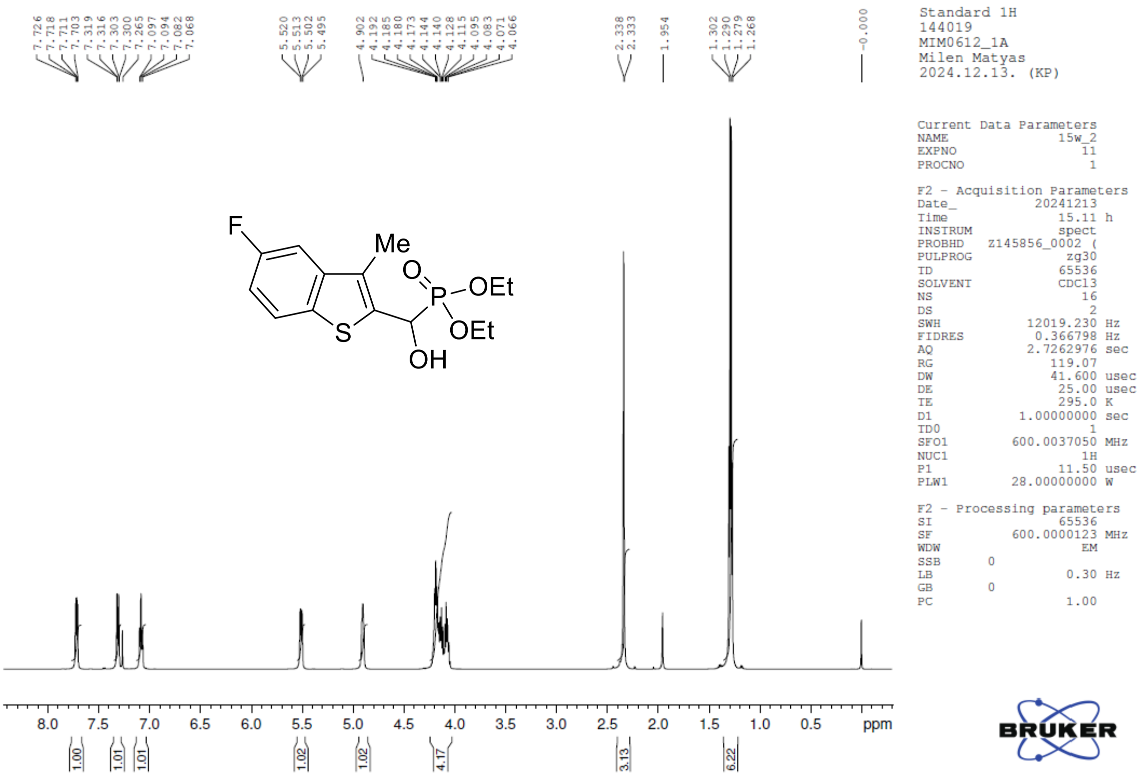

IR (KBr) spectra for compound 2l

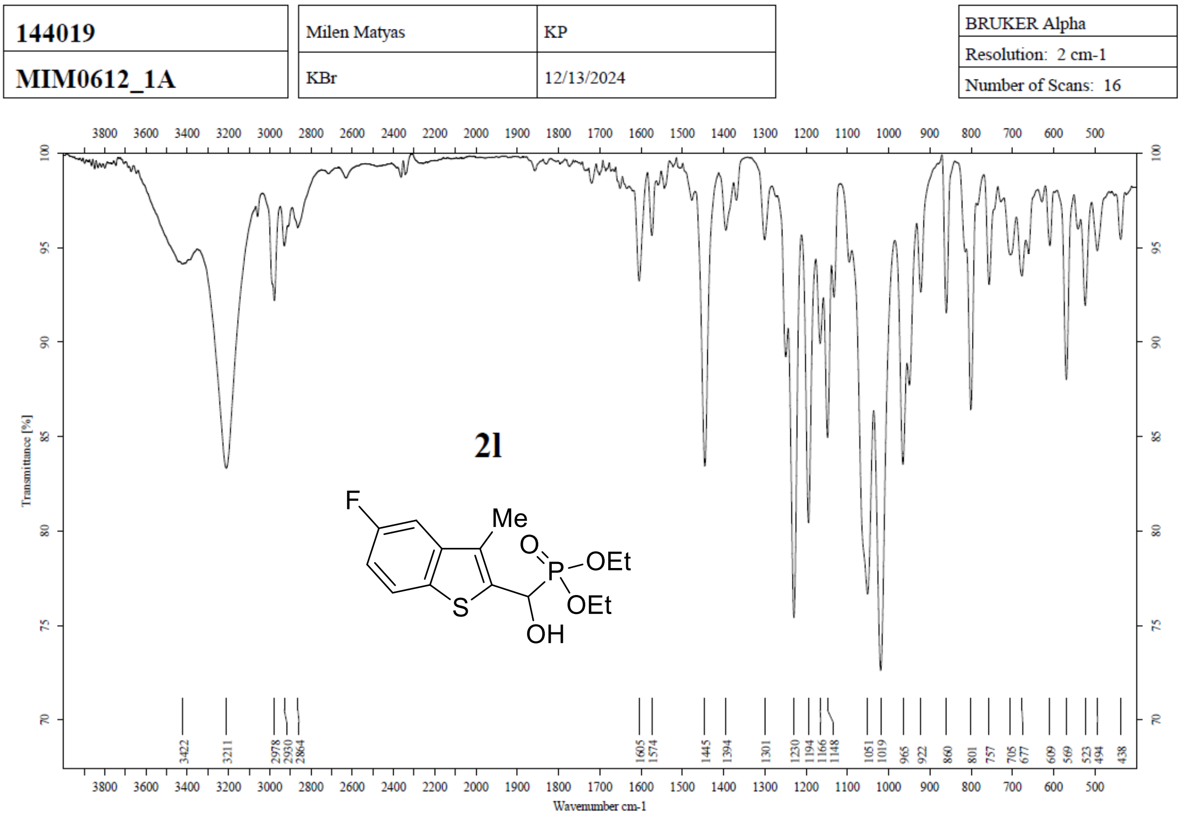

^{31}P NMR (242 MHz, $CDCl_3$) spectra for compound 2m

2m

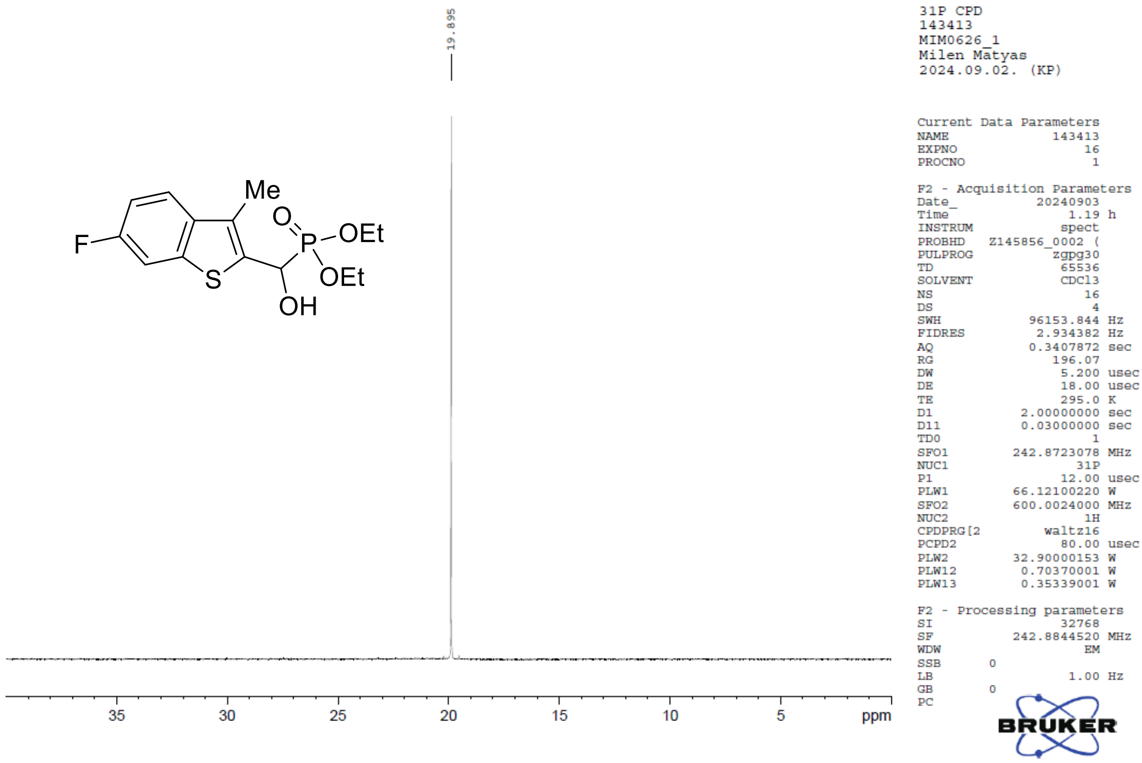

^{13}C NMR (150 MHz, $CDCl_3$) spectra for compound 2m

2m

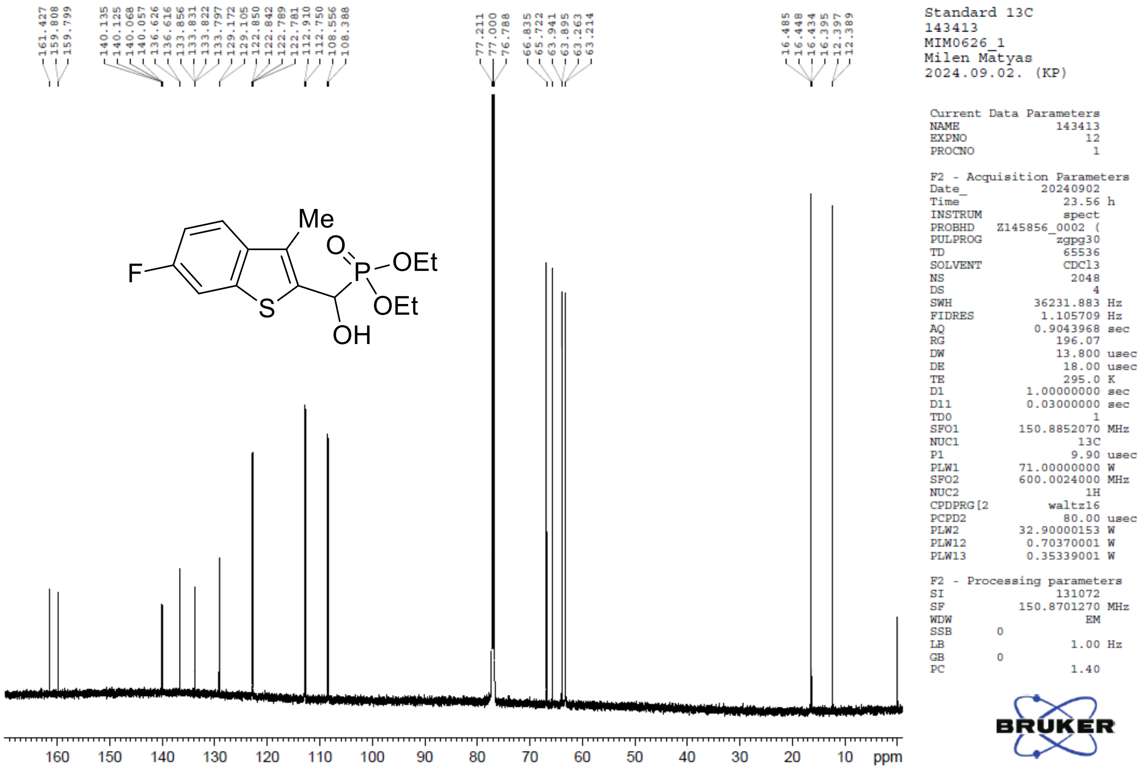

^{1}H NMR (600 MHz, $CDCl_3$) spectra for compound 2m

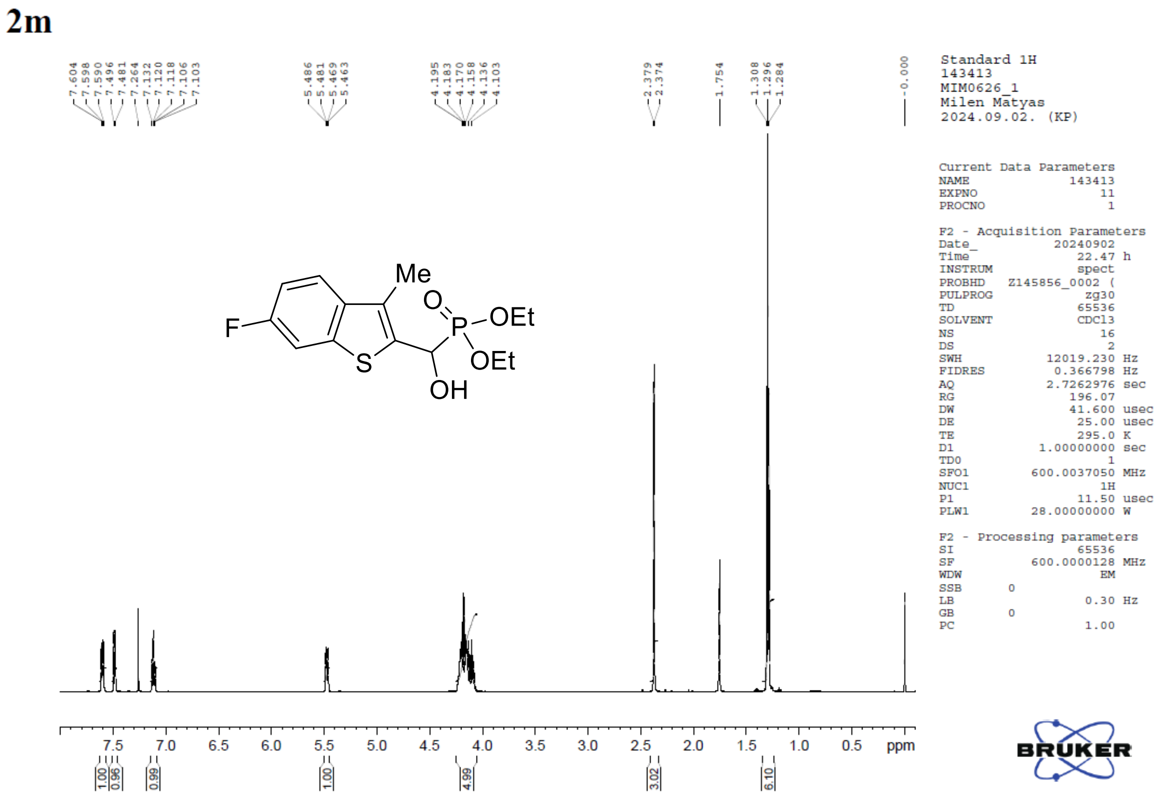

IR (KBr) spectra for compound 2m

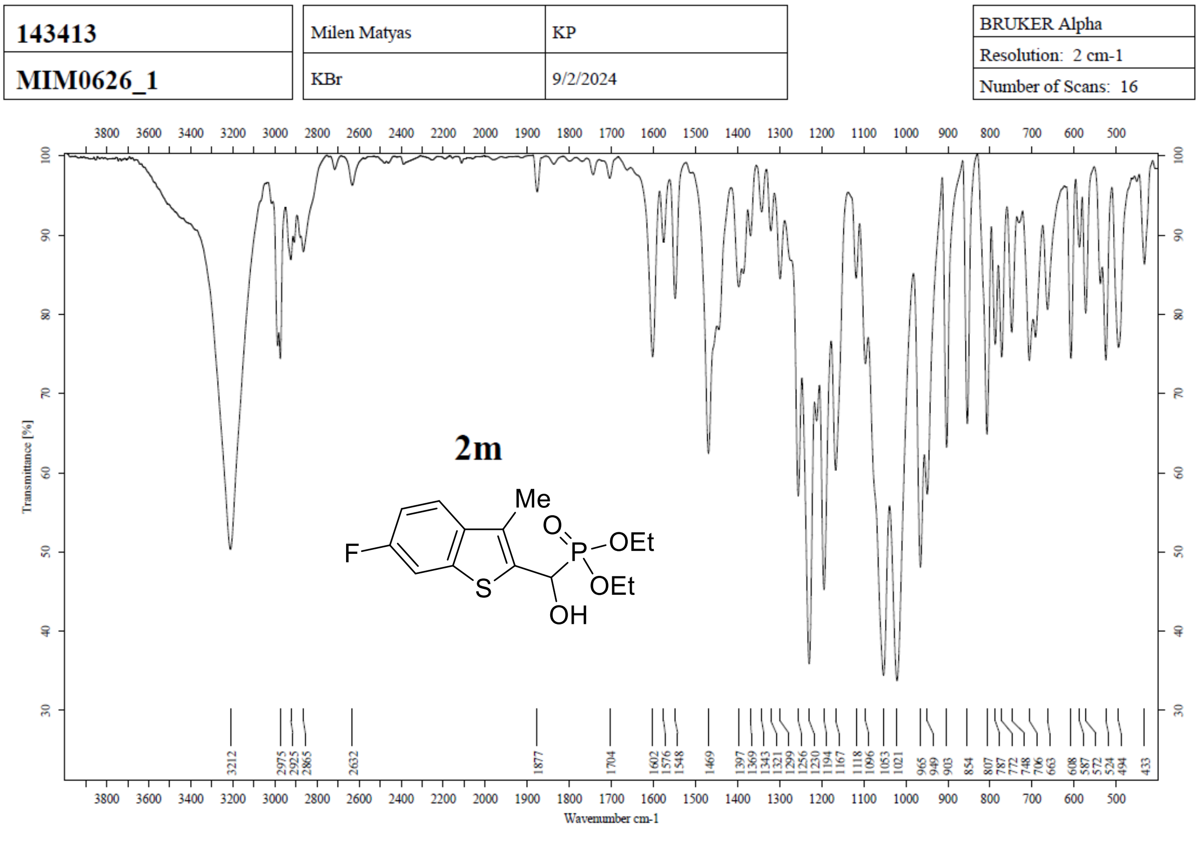

^{31}P NMR (242 MHz, $CDCl_3$) spectra for compound 2n

2n

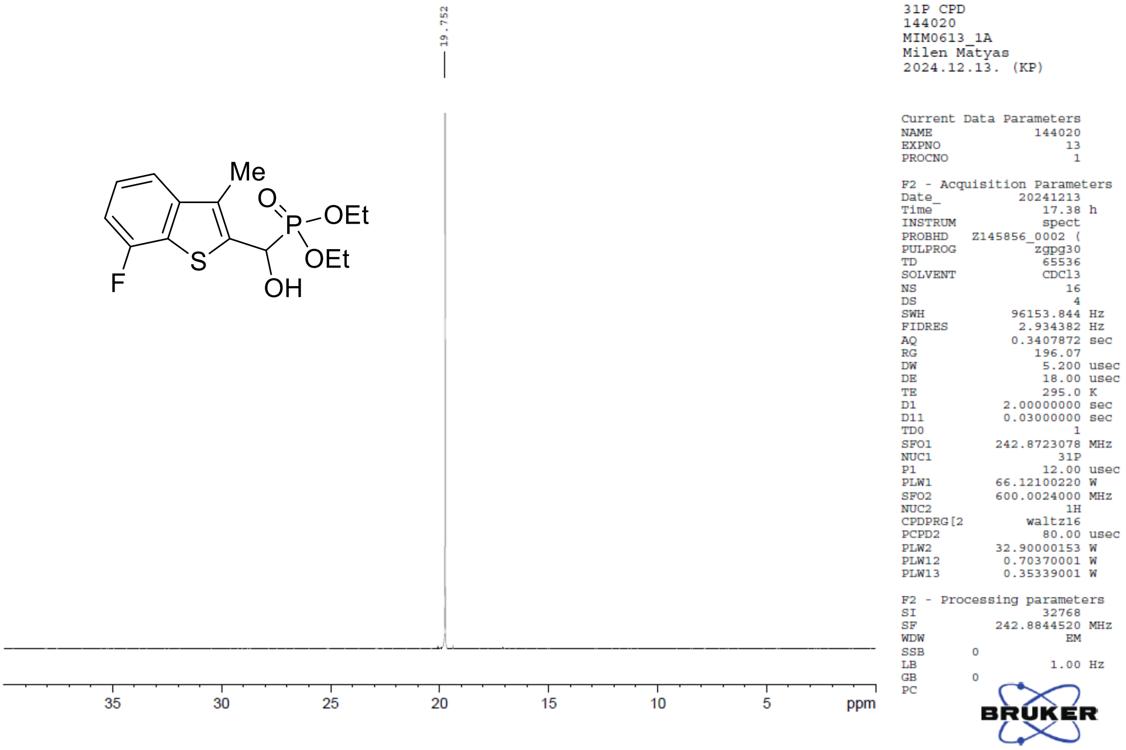

^{13}C NMR (150 MHz, $CDCl_3$) spectra for compound 2n

2n

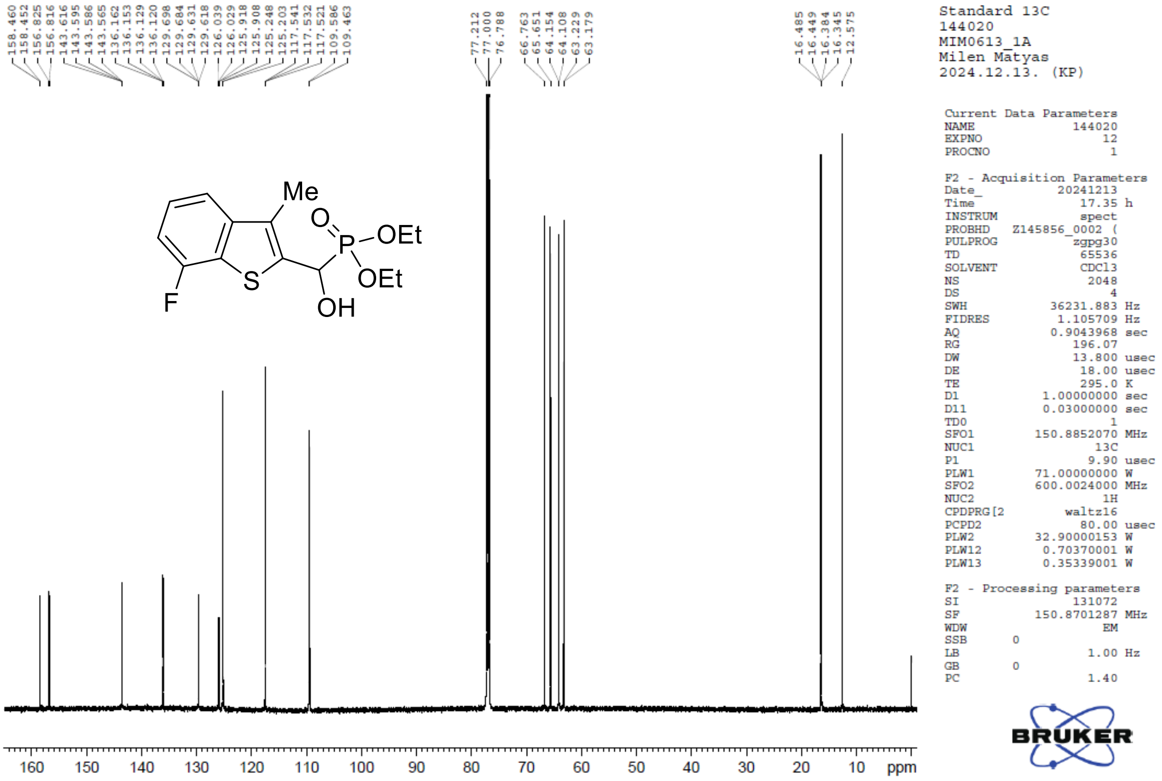

^{1}H NMR (600 MHz, $CDCl_3$) spectra for compound 2n

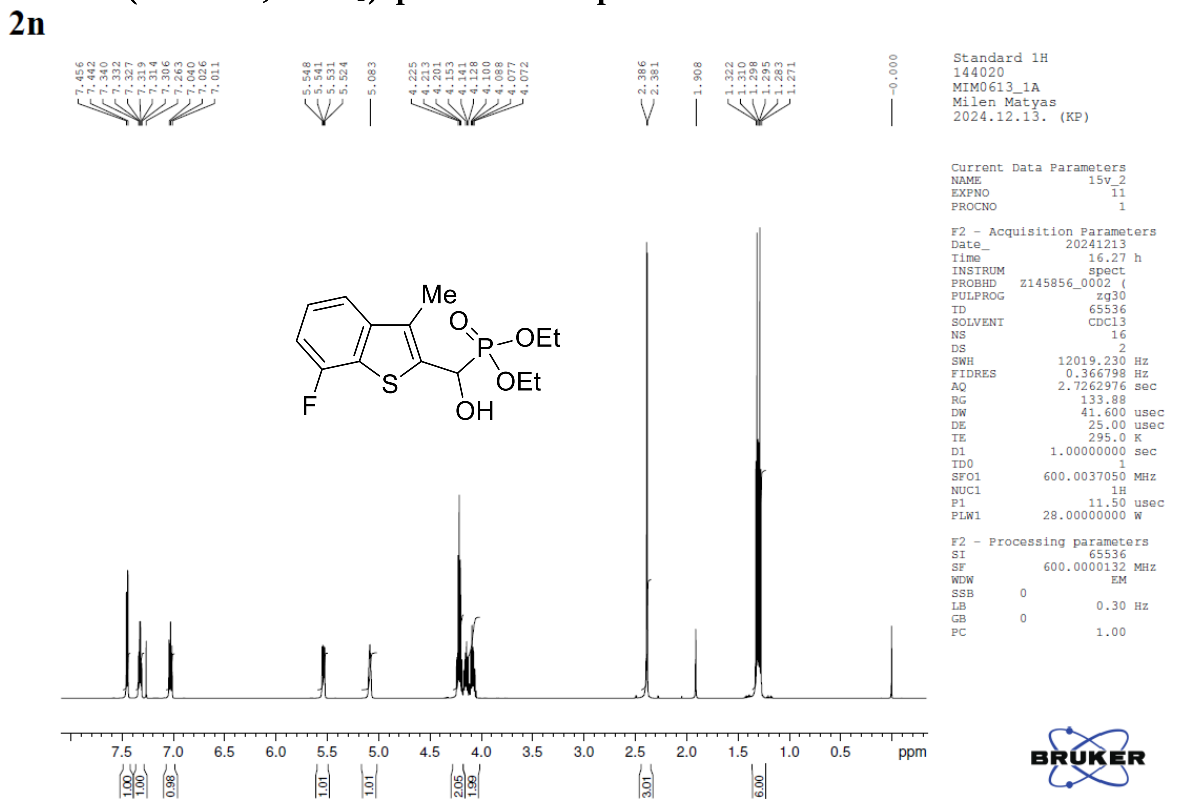

IR (KBr) spectra for compound 2n

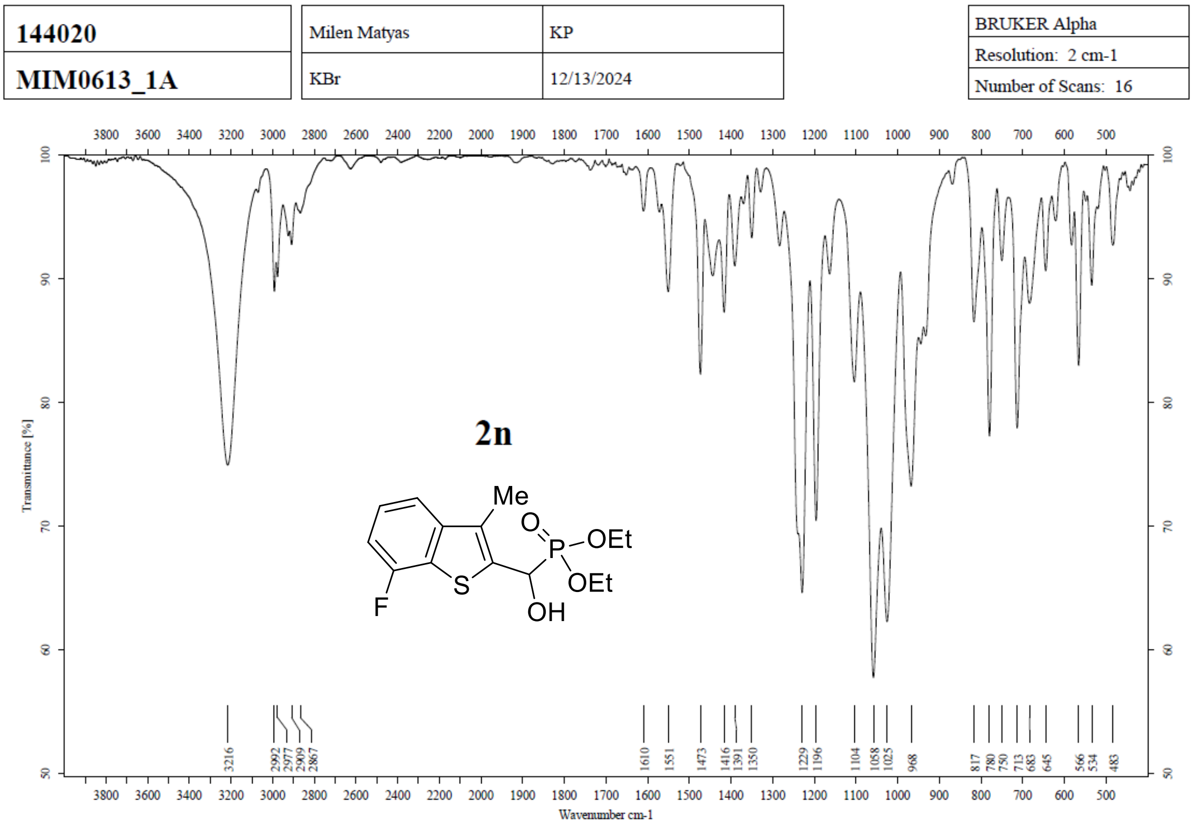

^{31}P NMR (242 MHz, $CDCl_3$) spectra for compound 2o

2o

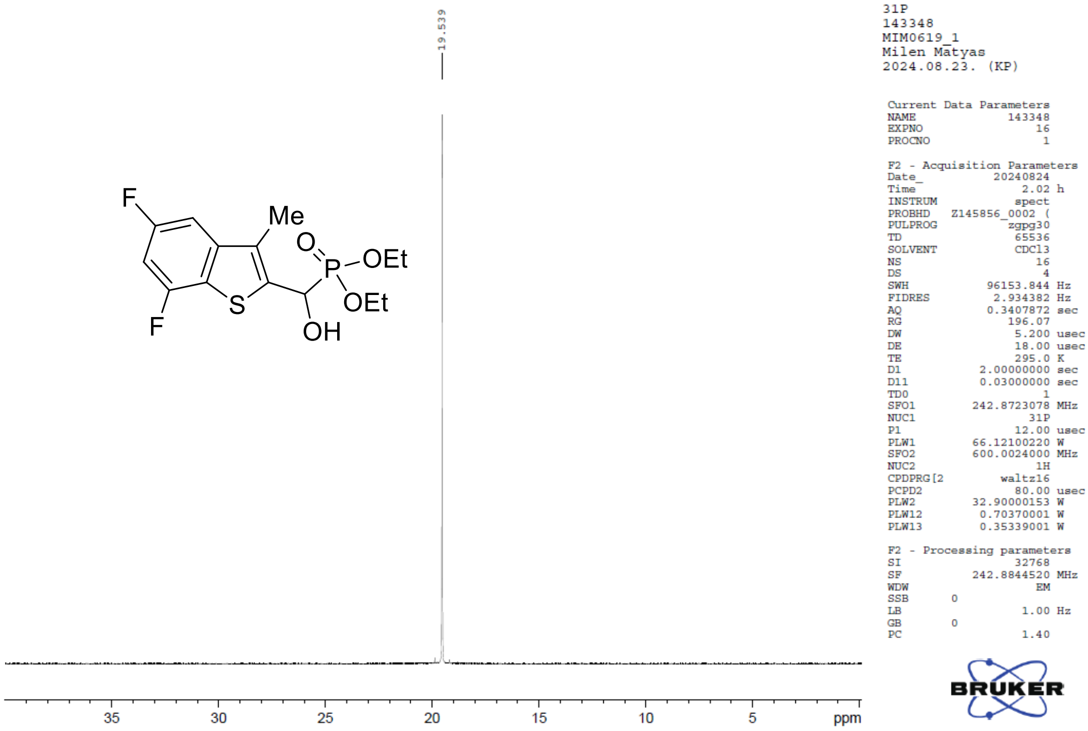

^{13}C NMR (150 MHz, $CDCl_3$) spectra for compound 2o

2o

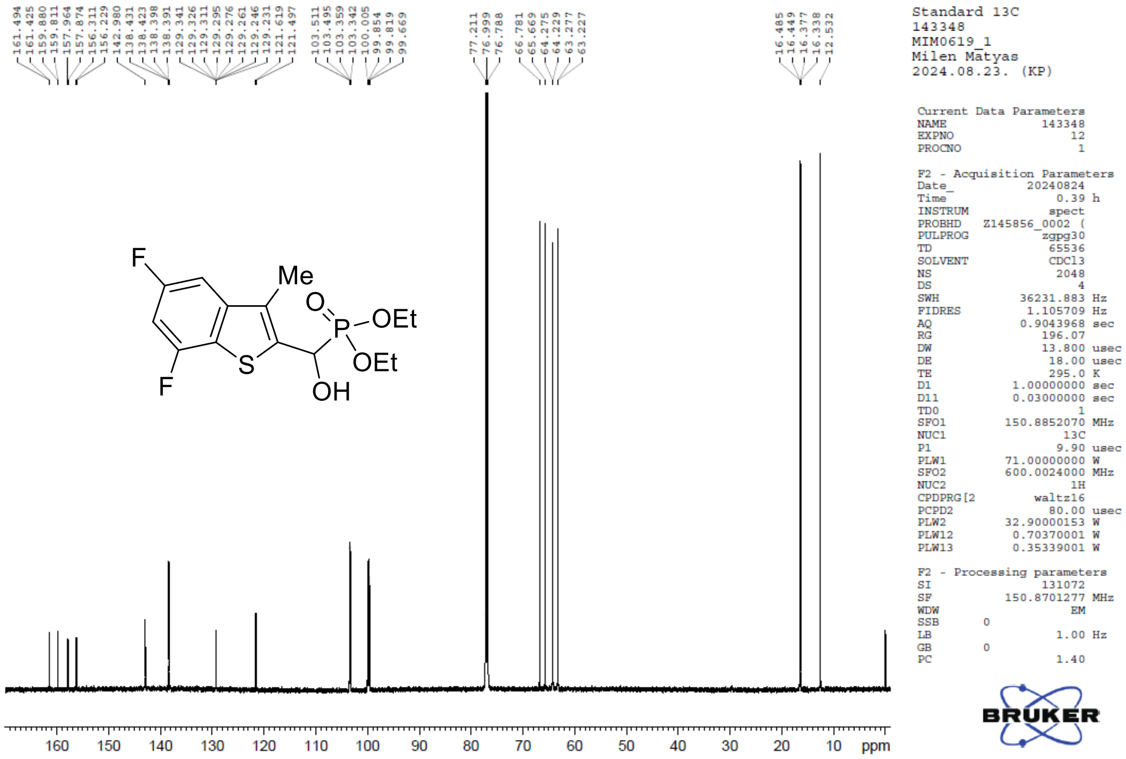

^{1}H NMR (600 MHz, $CDCl_3$) spectra for compound 2o

2o

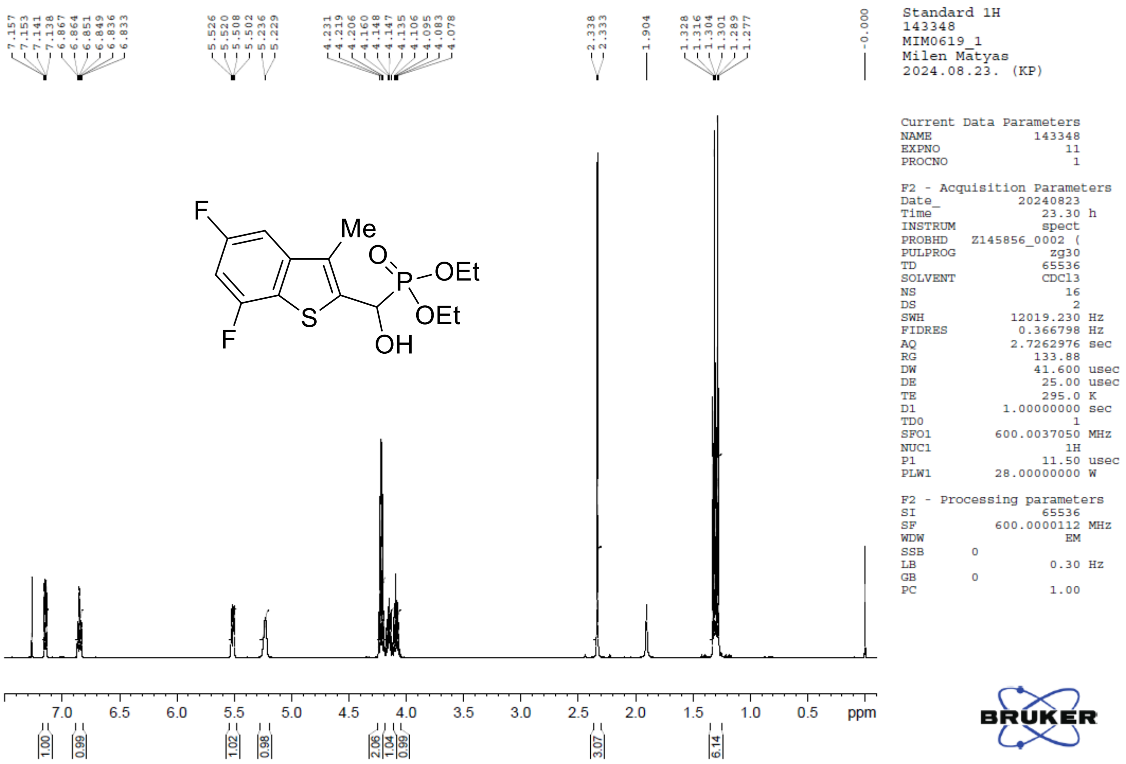

IR (KBr) spectra for compound 2o

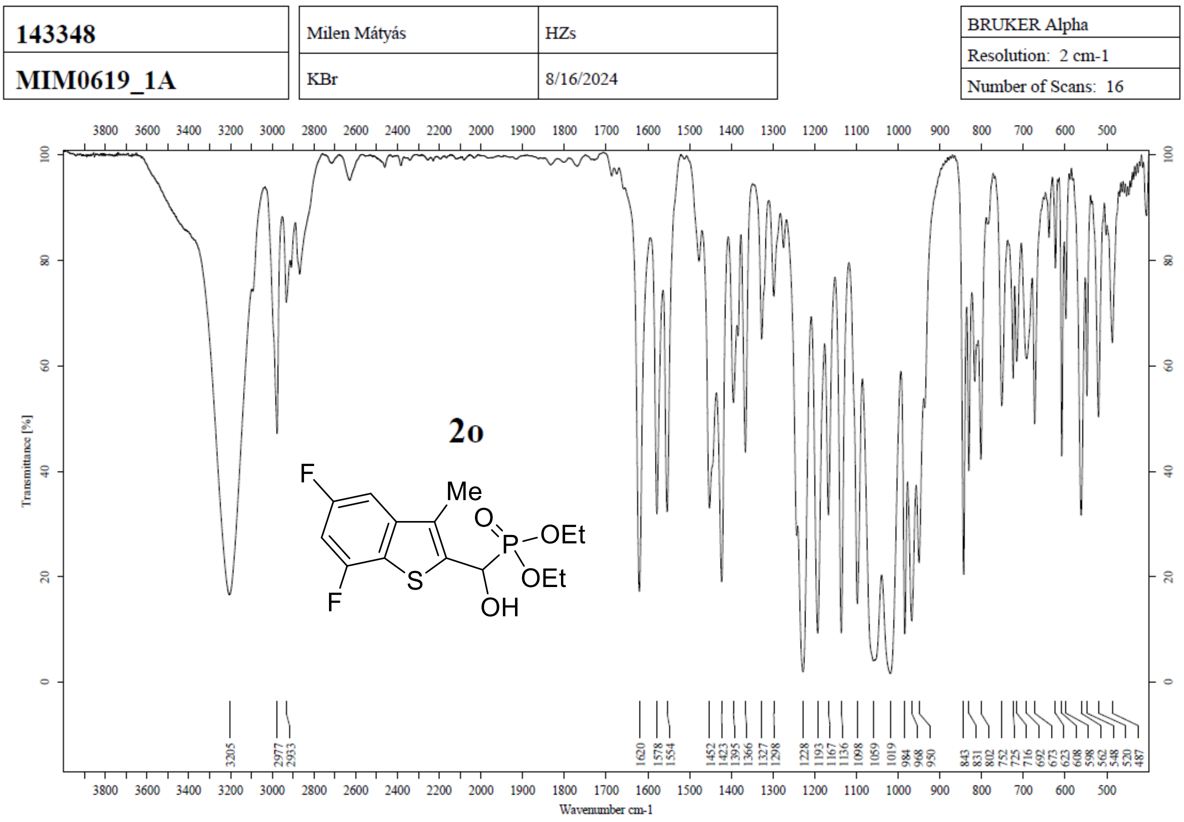

^{31}P NMR (242 MHz, $CDCl_3$) spectra for compound 2p

2p

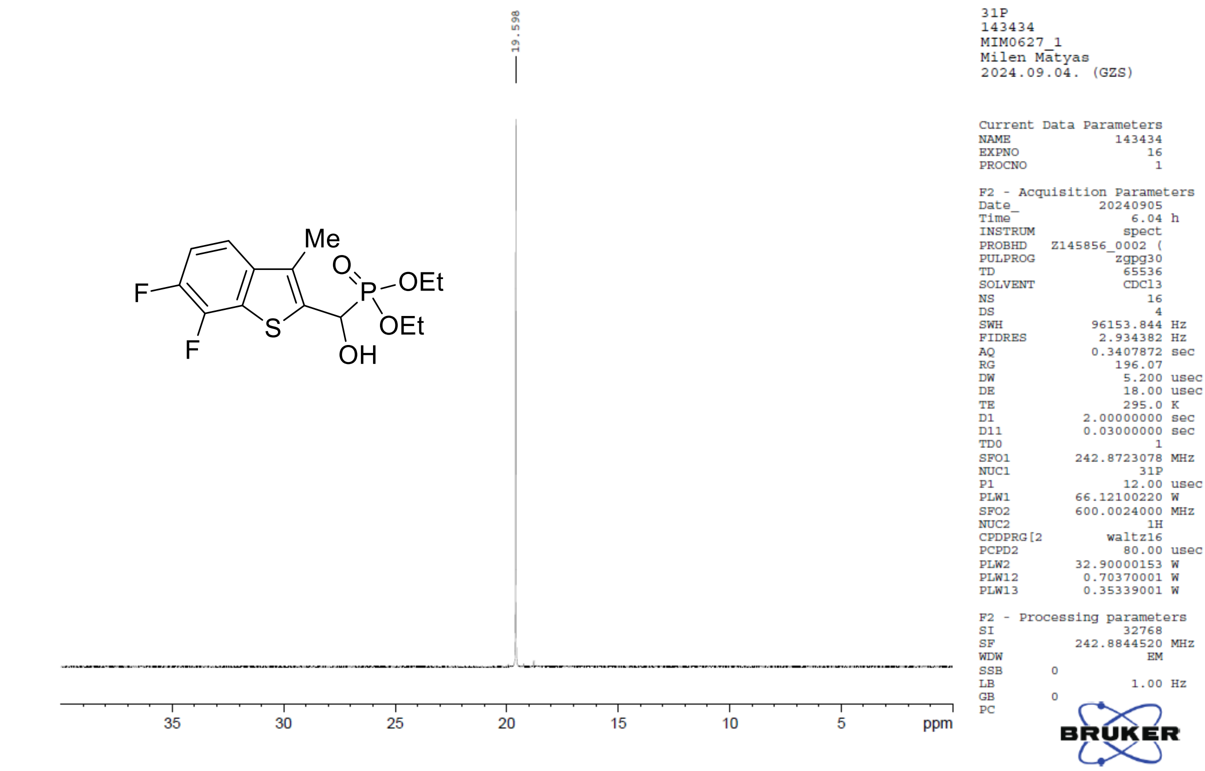

^{13}C NMR (150 MHz, $CDCl_3$) spectra for compound 2p

2p

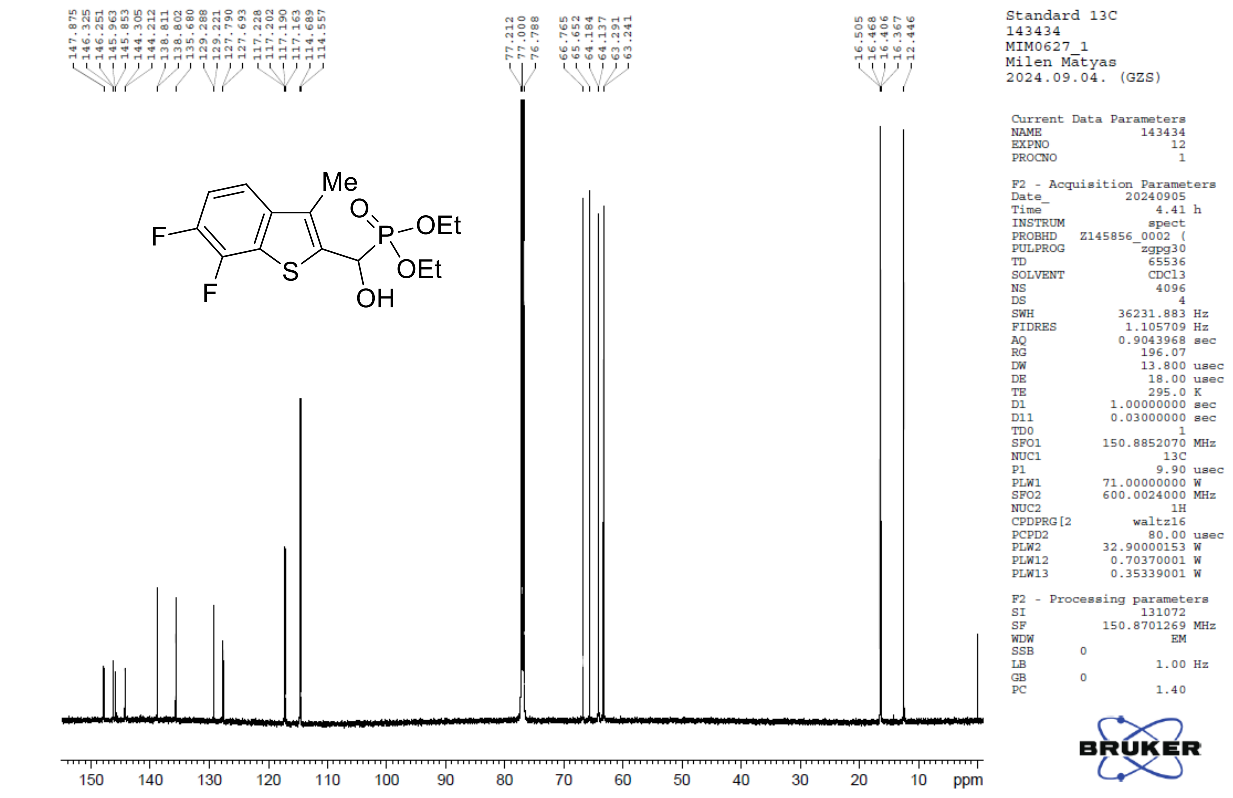

^{1}H NMR (600 MHz, $CDCl_3$) spectra for compound 2p

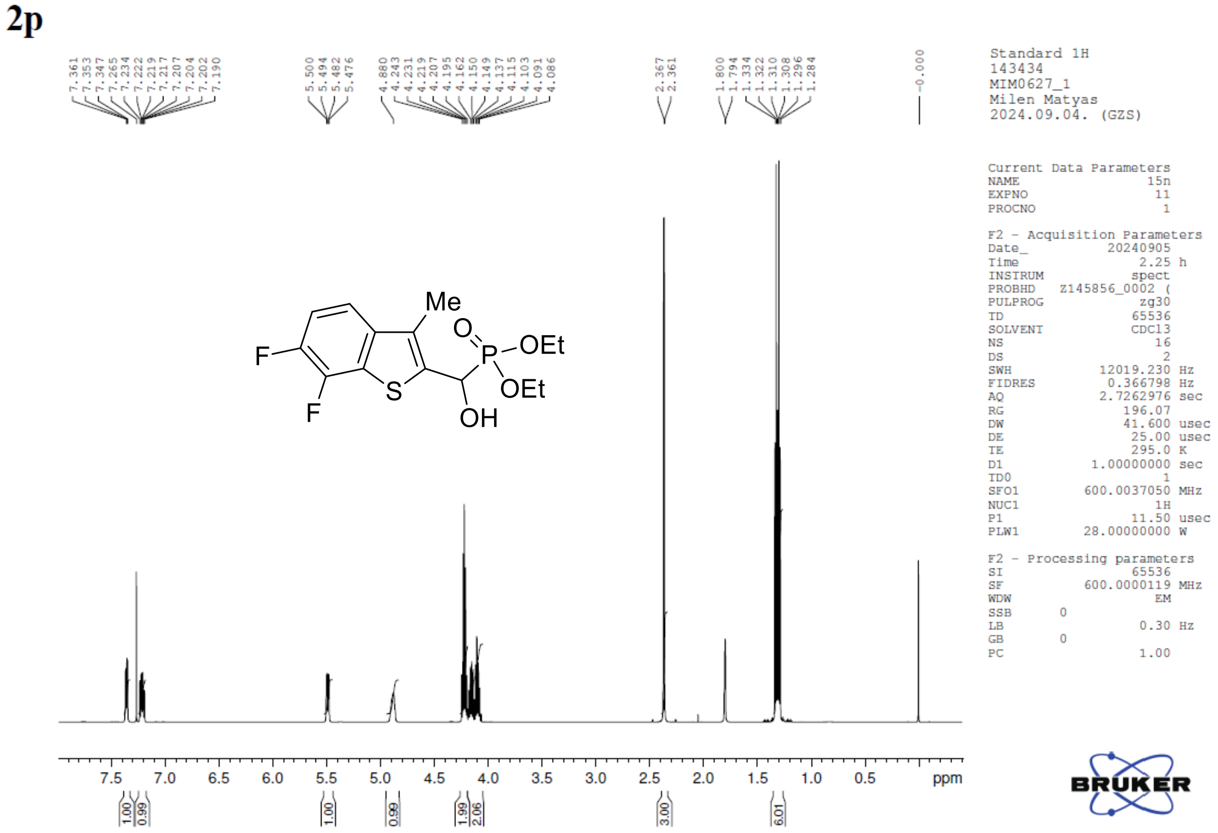

IR (KBr) spectra for compound 2p

143434	Milen Matyas	KP	BRUKER Alpha
MIM0627_1	KBr	9/5/2024	Resolution: 2 cm-1
			Number of Scans: 16

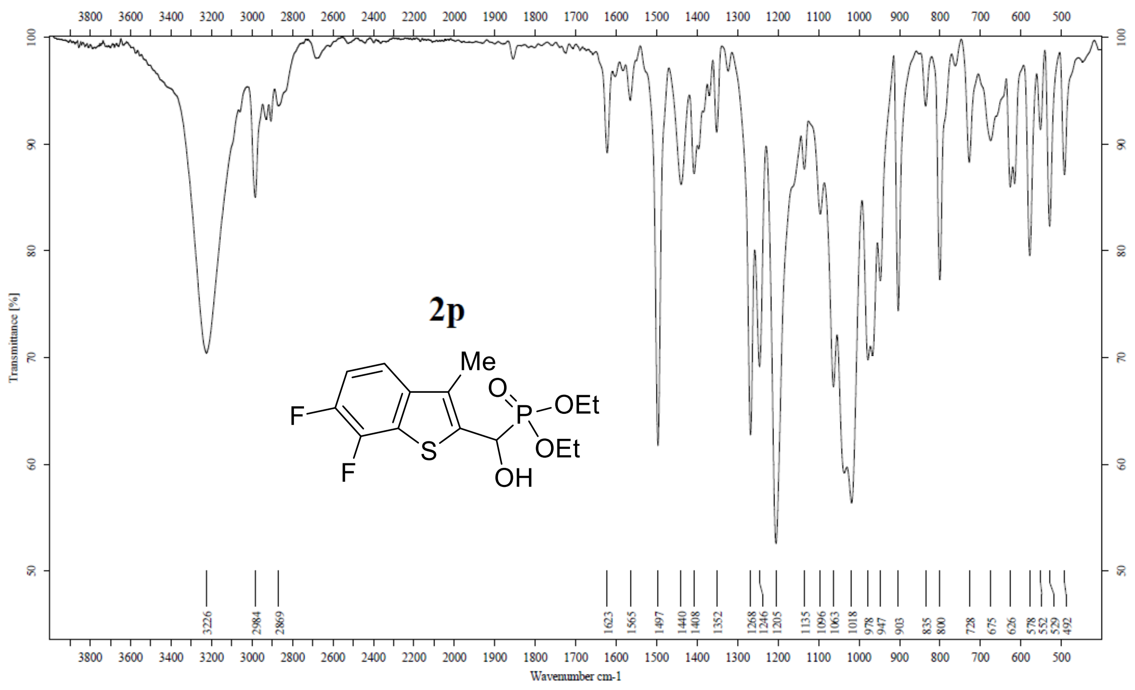

^{31}P NMR (242 MHz, CDCl$_3$) spectra for compound 2q

2q

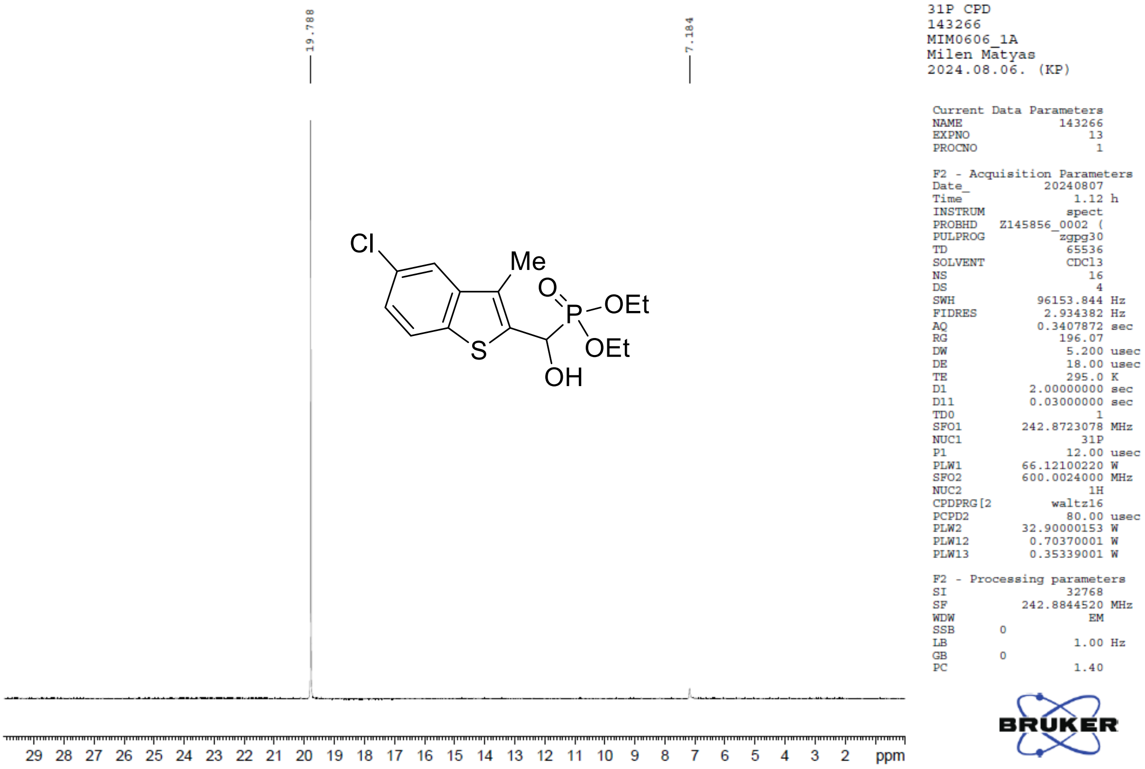

^{13}C NMR (150 MHz, CDCl$_3$) spectra for compound 2q

2q

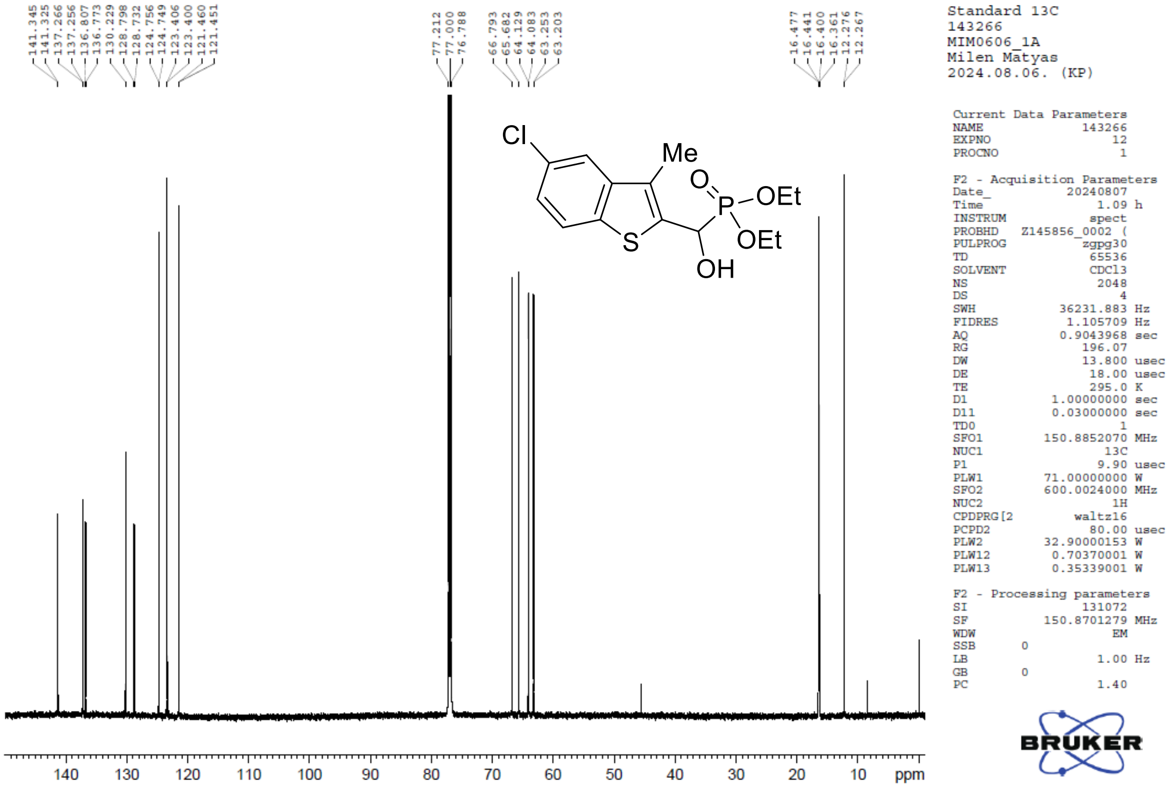

^{1}H NMR (600 MHz, $CDCl_3$) spectra for compound 2q

2q

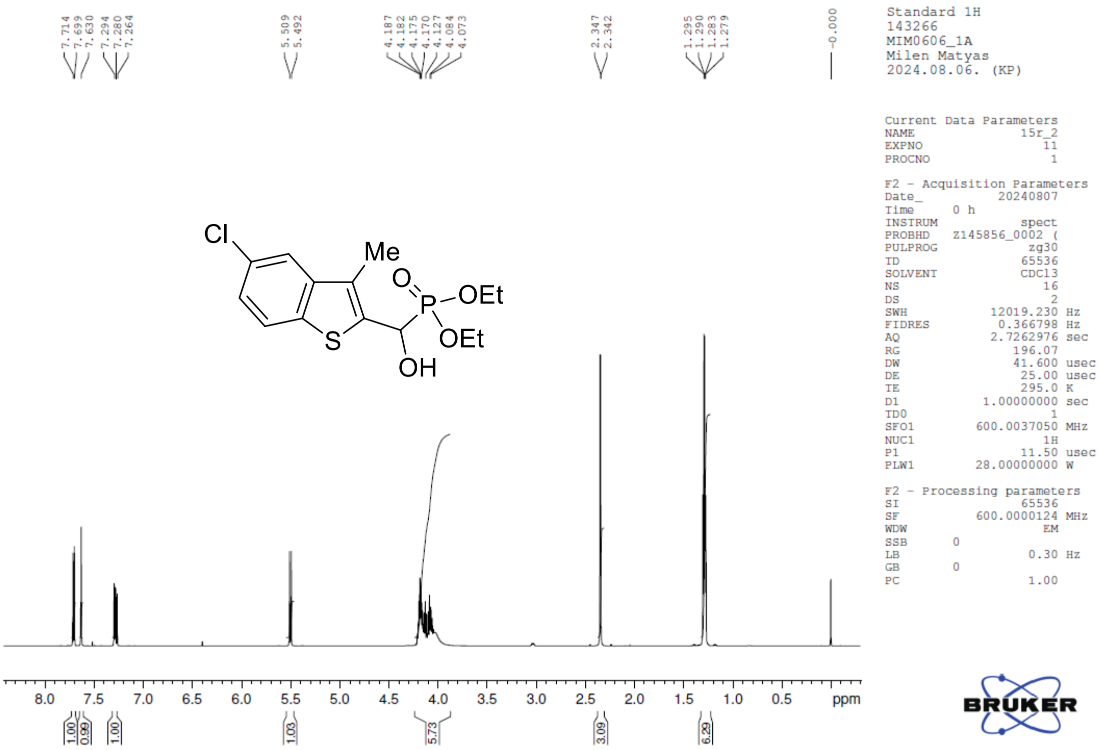

IR (KBr) spectra for compound 2q

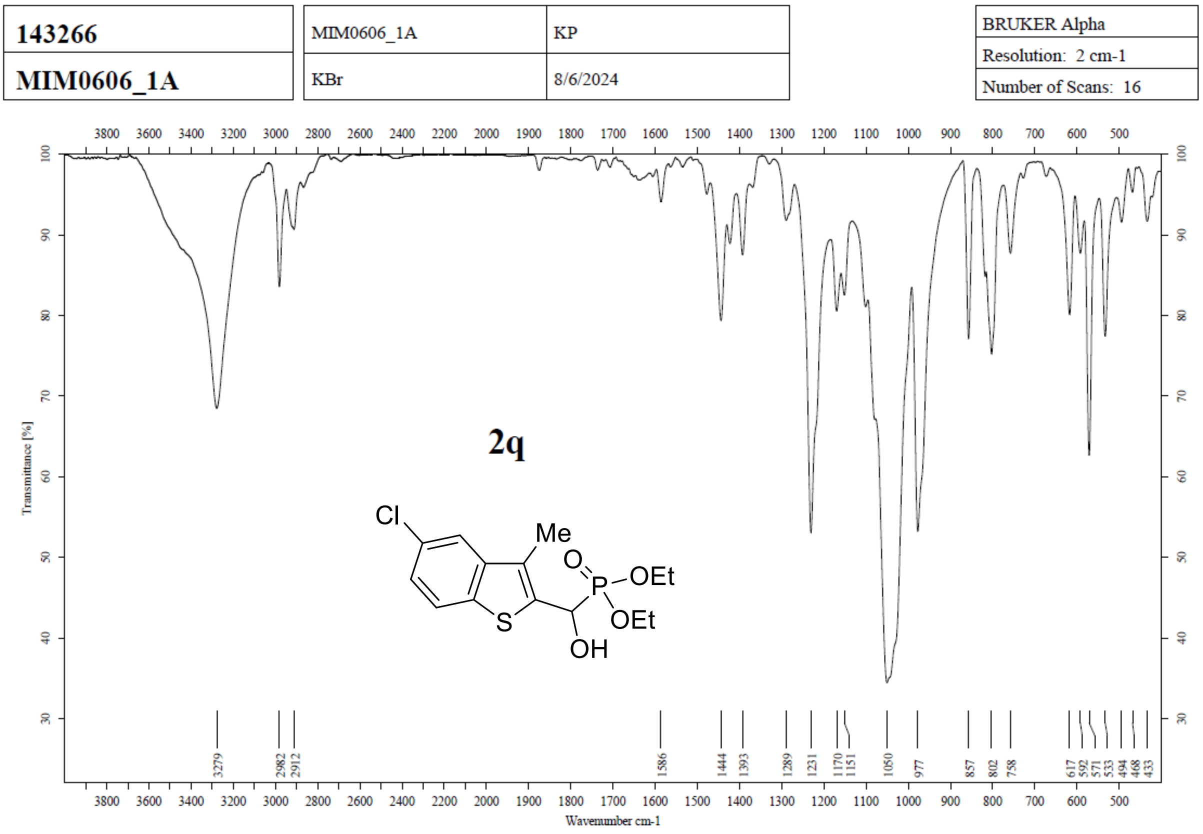

^{31}P NMR (242 MHz, DMSO-d_6) spectra for compound 2r

2r

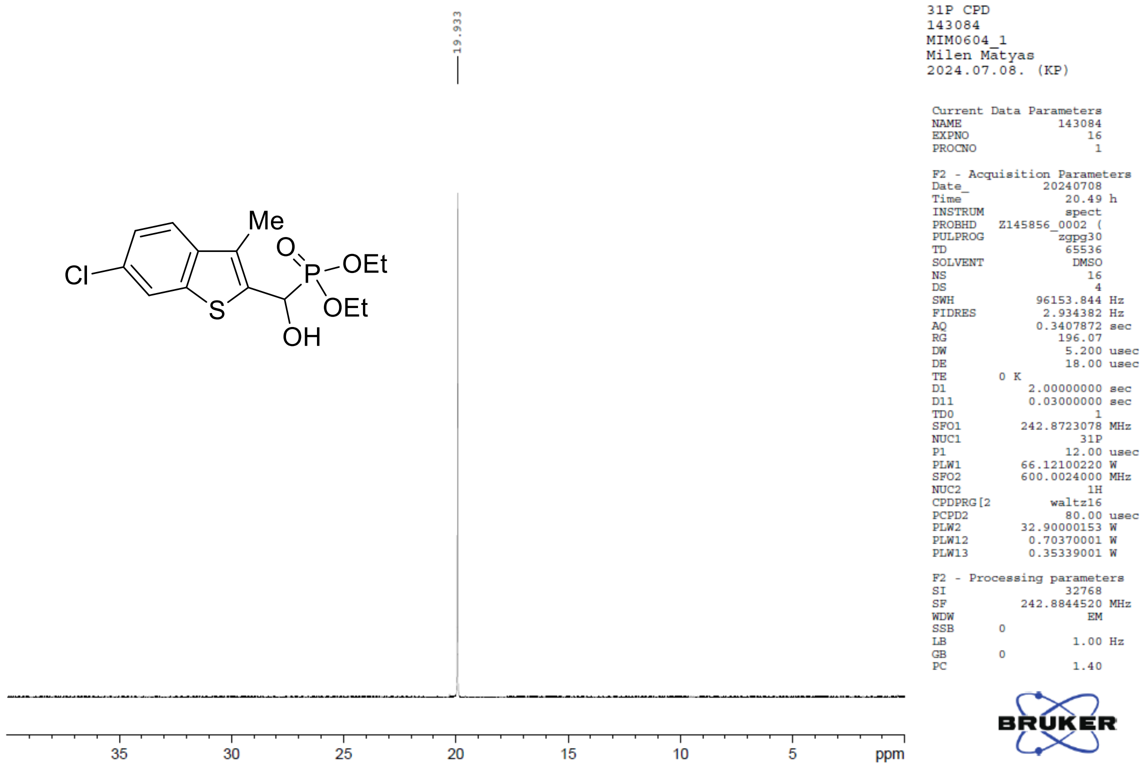

^{13}C NMR (150 MHz, DMSO-d_6) spectra for compound 2r

2r

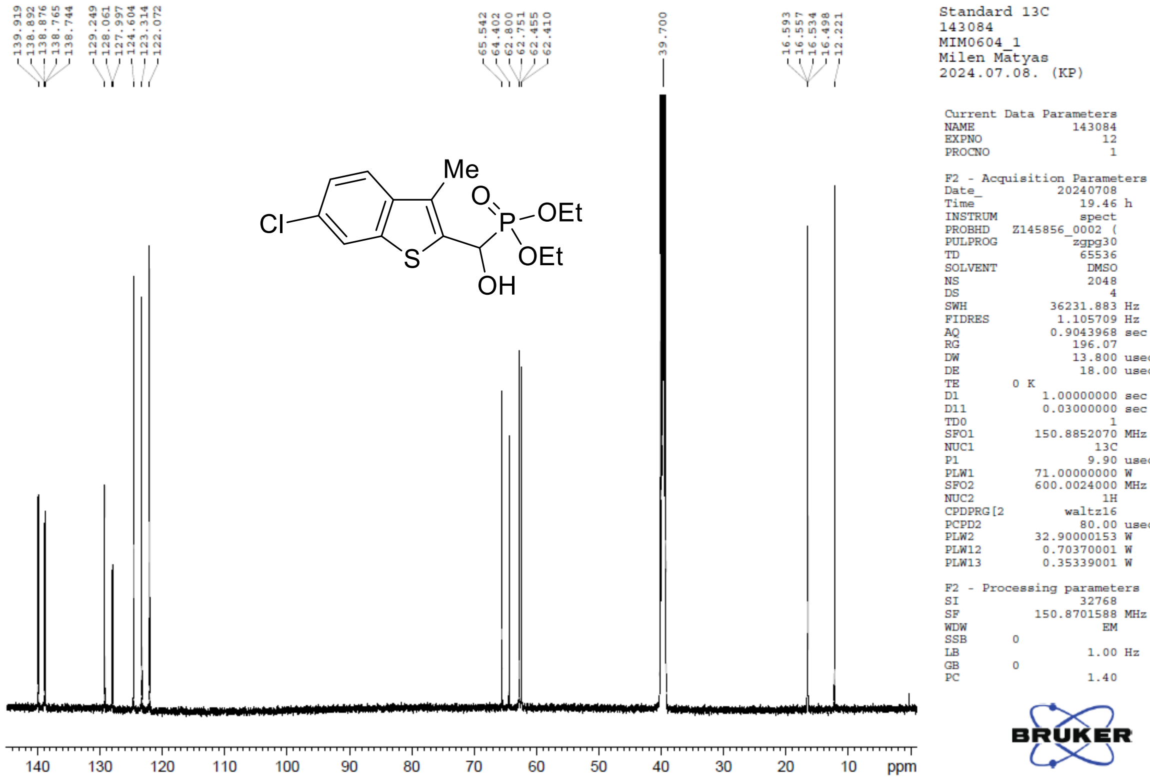

^{1}H NMR (600 MHz, DMSO-d_6) spectra for compound 2r

2r

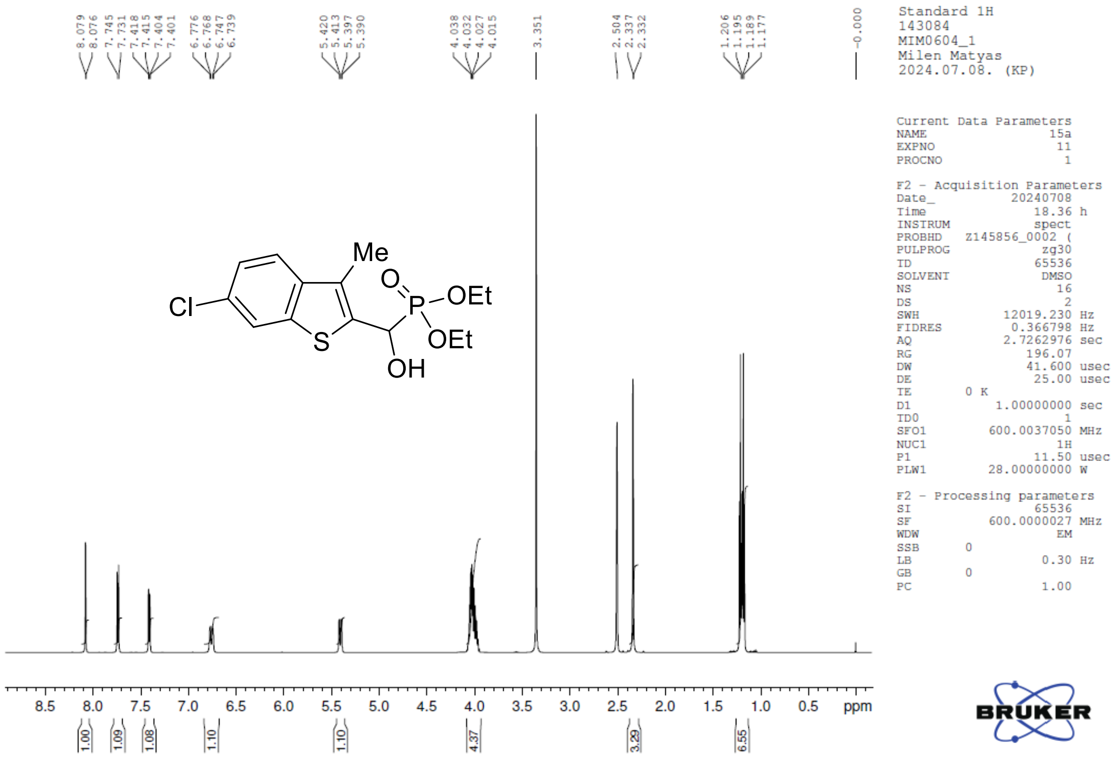

IR (KBr) spectra for compound 2r

143084	Milen Mátyás	PZ	BRUKER Alpha
MIM0604_1	KBr	7/8/2024	Resolution: 2 cm-1
			Number of Scans: 16

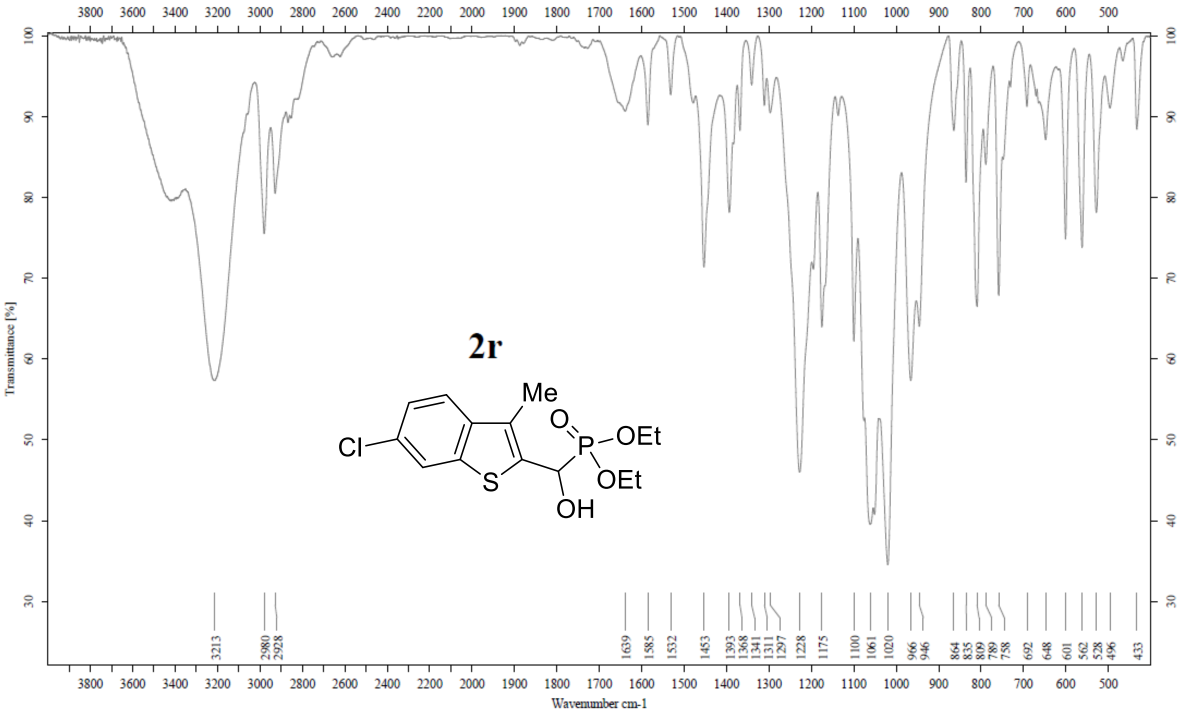

^{31}P NMR (600 MHz, $CDCl_3$) spectra for compound 2s

2s

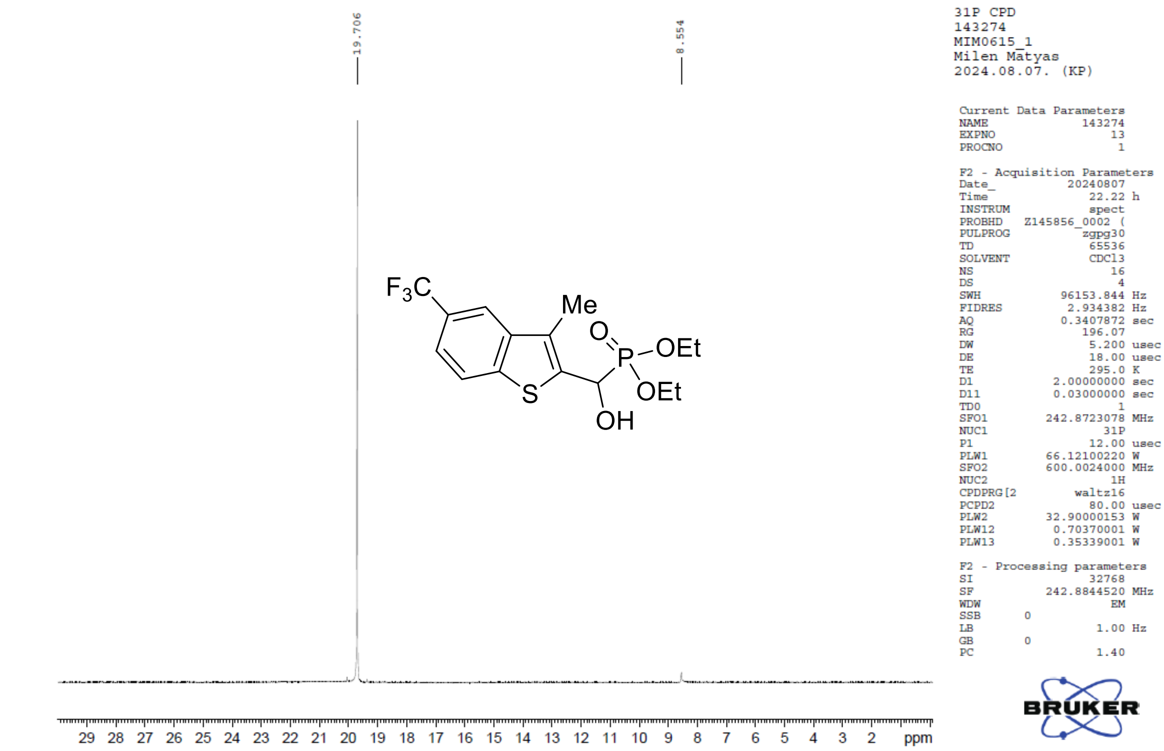

^{13}C NMR (150 MHz, $CDCl_3$) spectra for compound 2s

2s

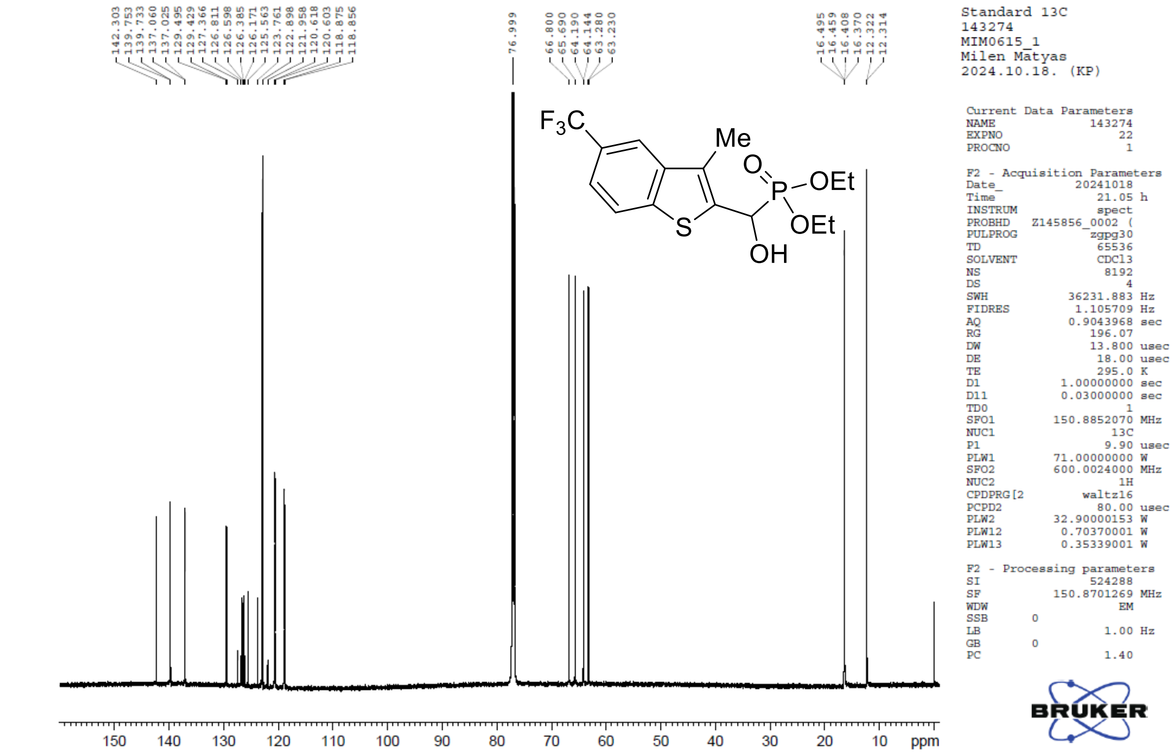

^{1}H NMR (600 MHz, $CDCl_3$) spectra for compound 2s

2s

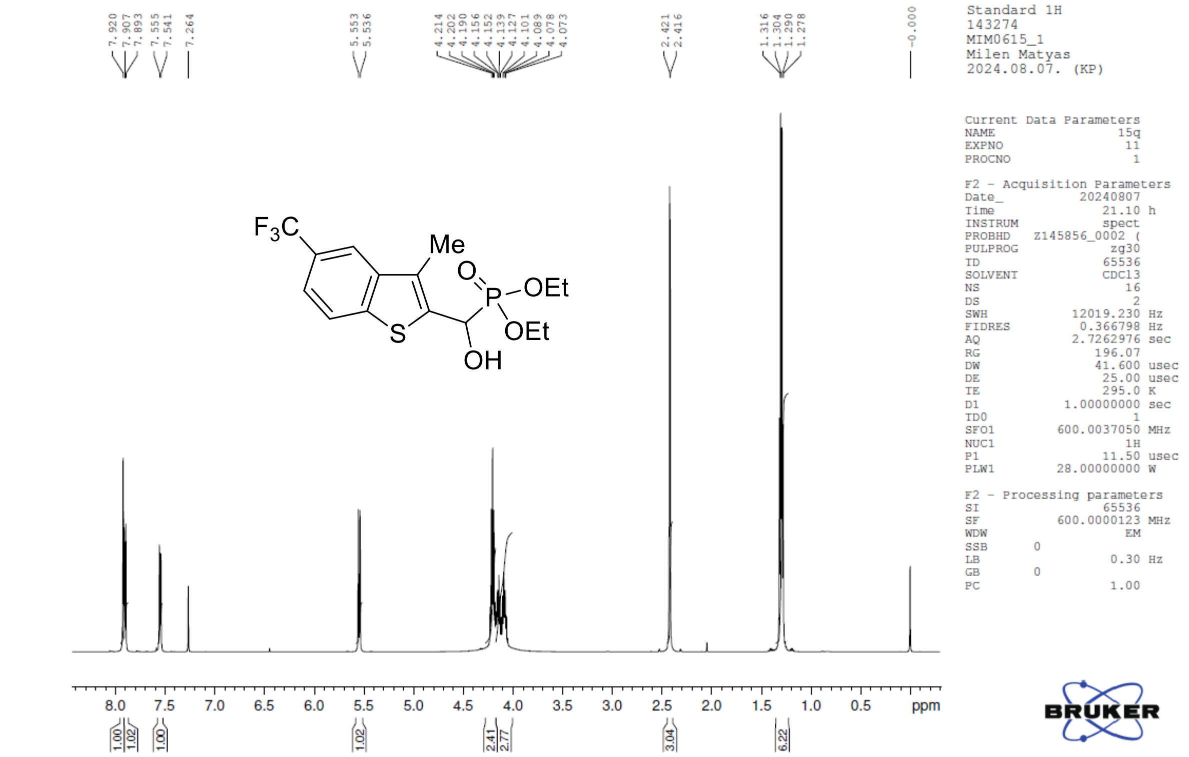

IR (KBr) spectra for compound 2s

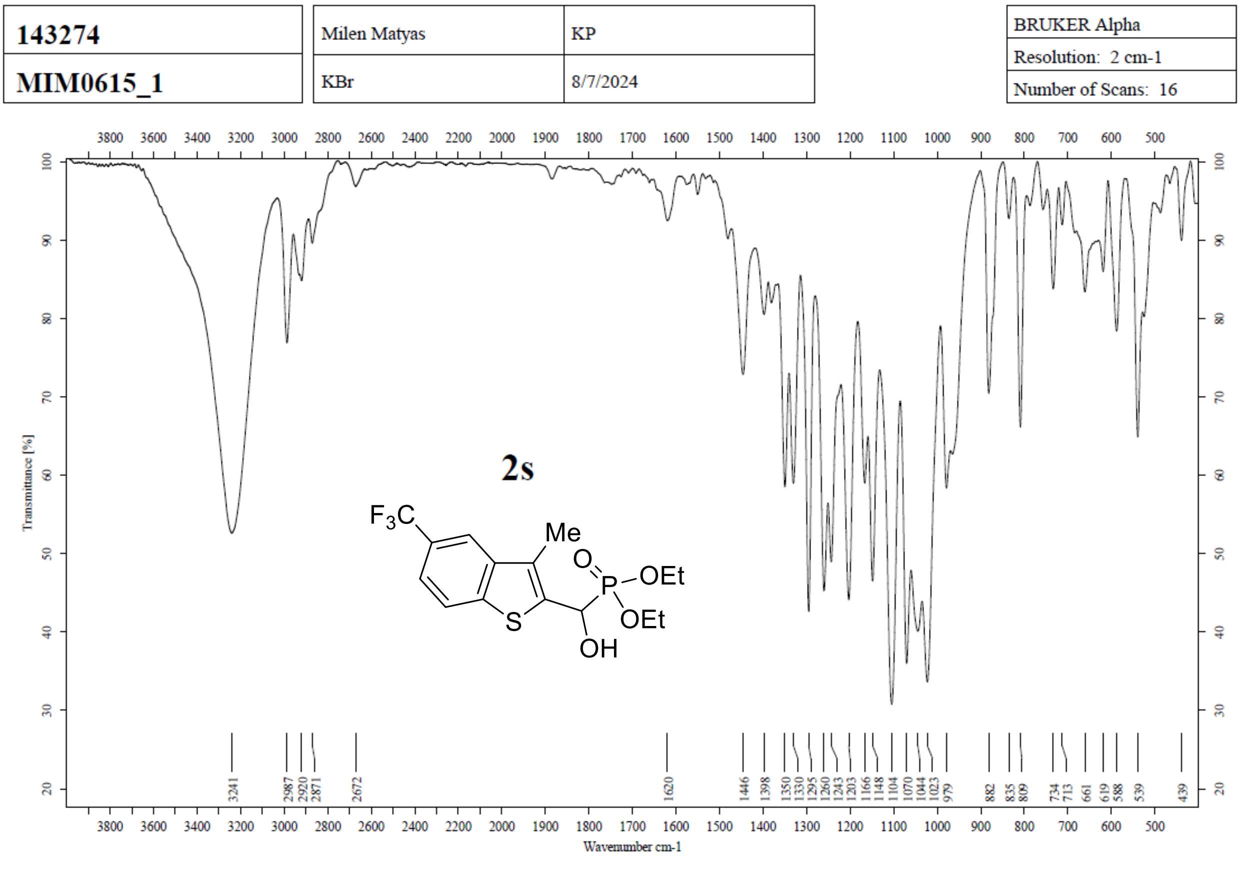

^{31}P NMR (242 MHz, $CDCl_3$) spectra for compound 2t

2t

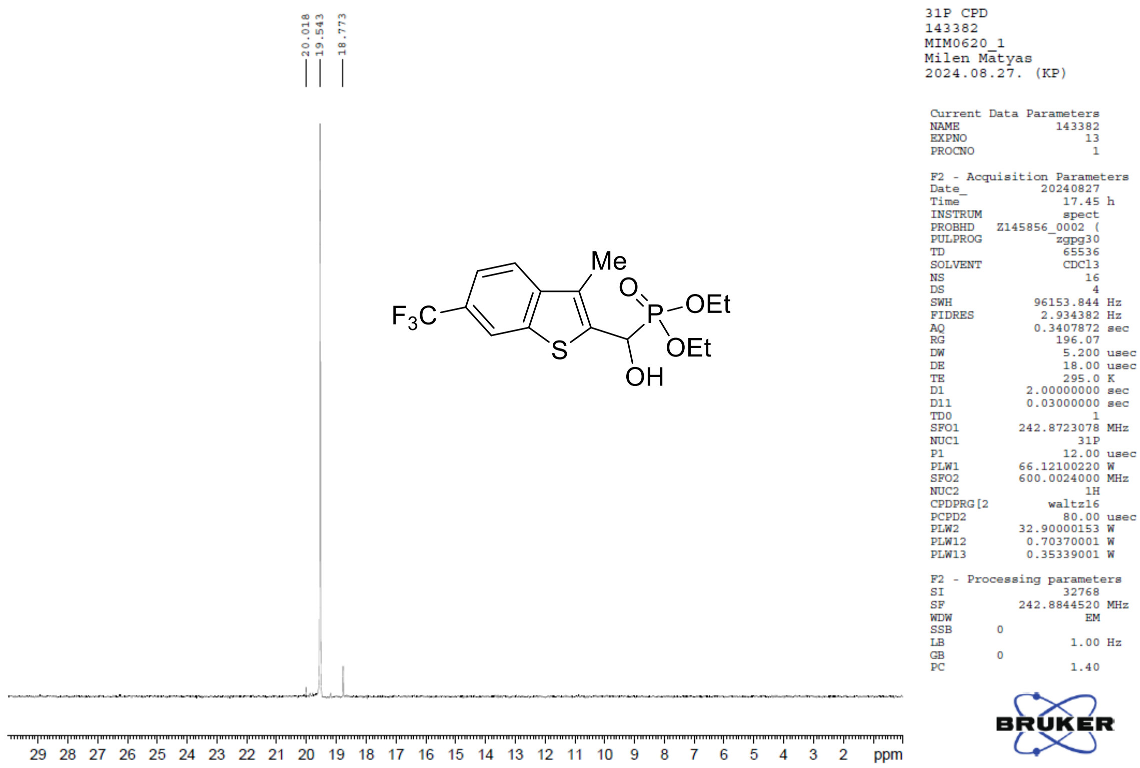

^{13}C NMR (150 MHz, $CDCl_3$) spectra for compound 2t

2t

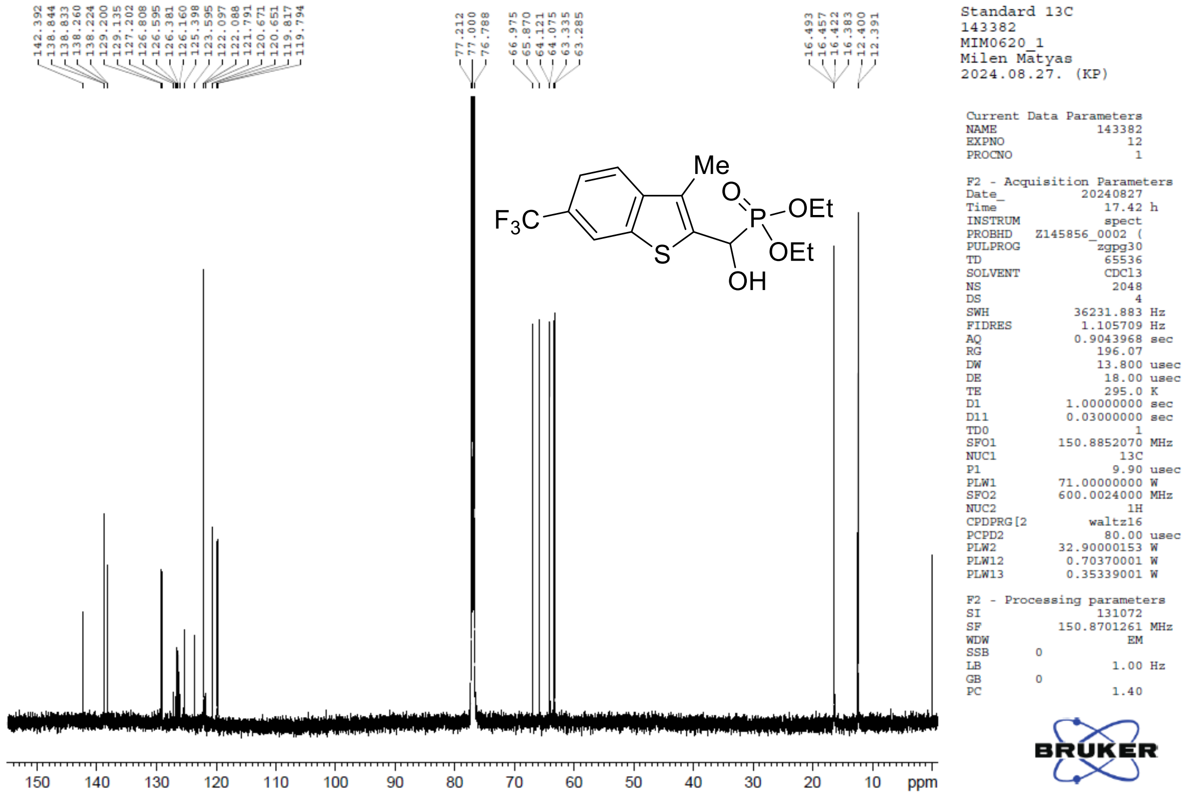

^{1}H NMR (600 MHz, $CDCl_3$) spectra for compound 2t

2t

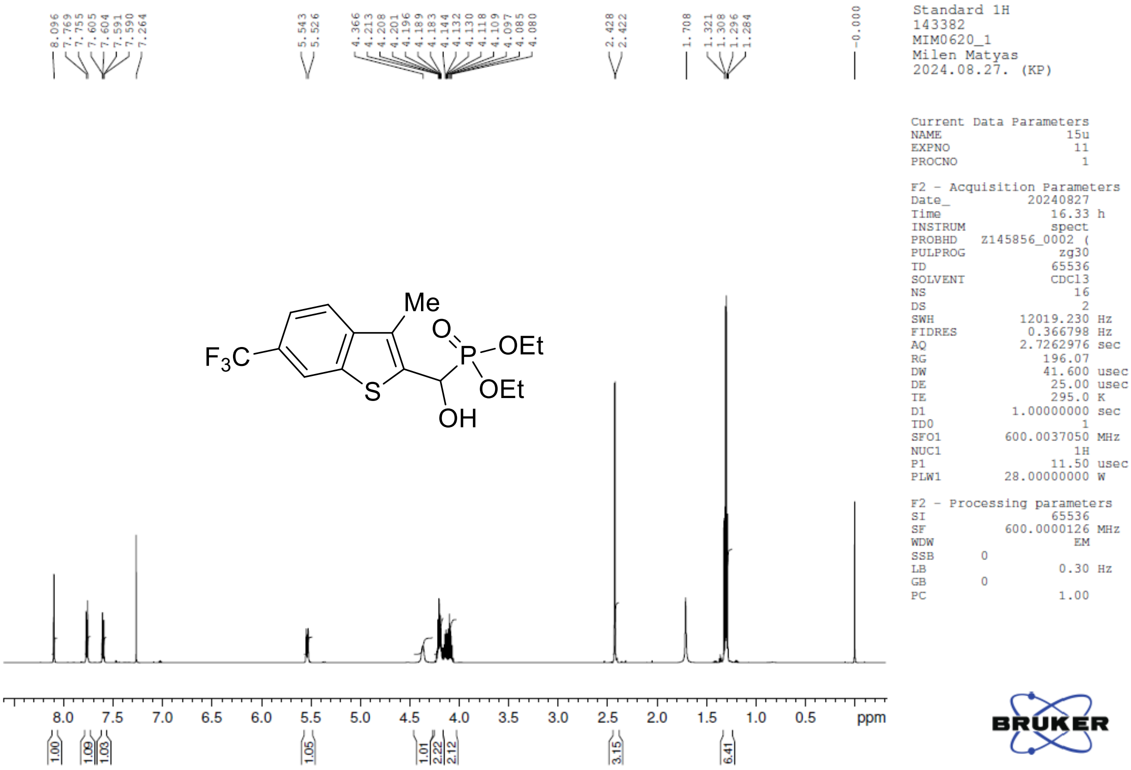

IR (KBr) spectra for compound 2t

143382	Milen Matyas	KP
MIM0620_1	KBr	9/3/2024

BRUKER Alpha
Resolution: 2 cm-1
Number of Scans: 16

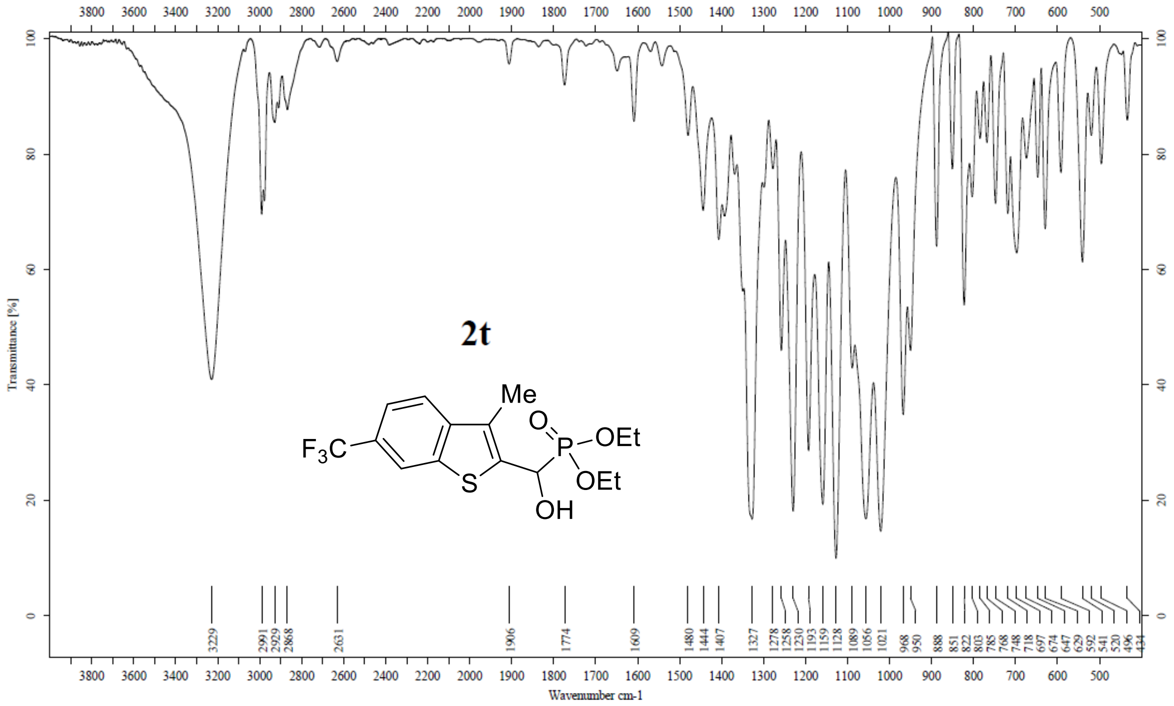

^{31}P NMR (242 MHz, $CDCl_3$) spectra for compound 2u

2u

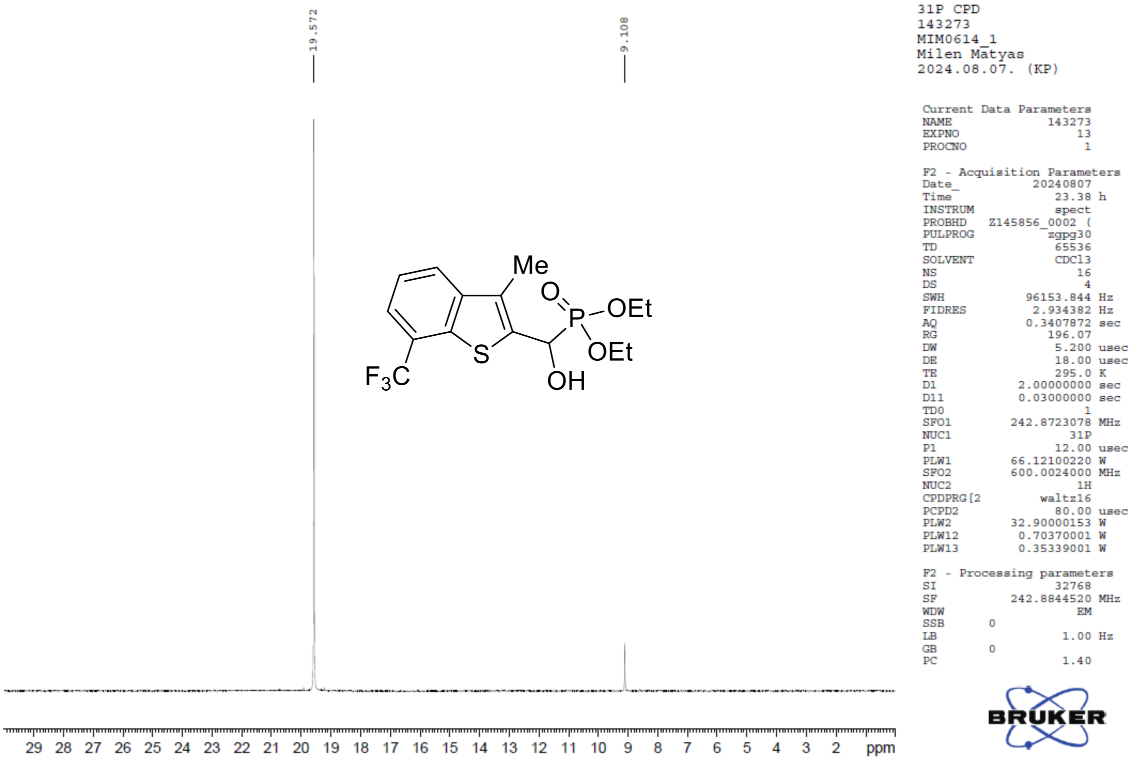

^{13}C NMR (150 MHz, $CDCl_3$) spectra for compound 2u

2u

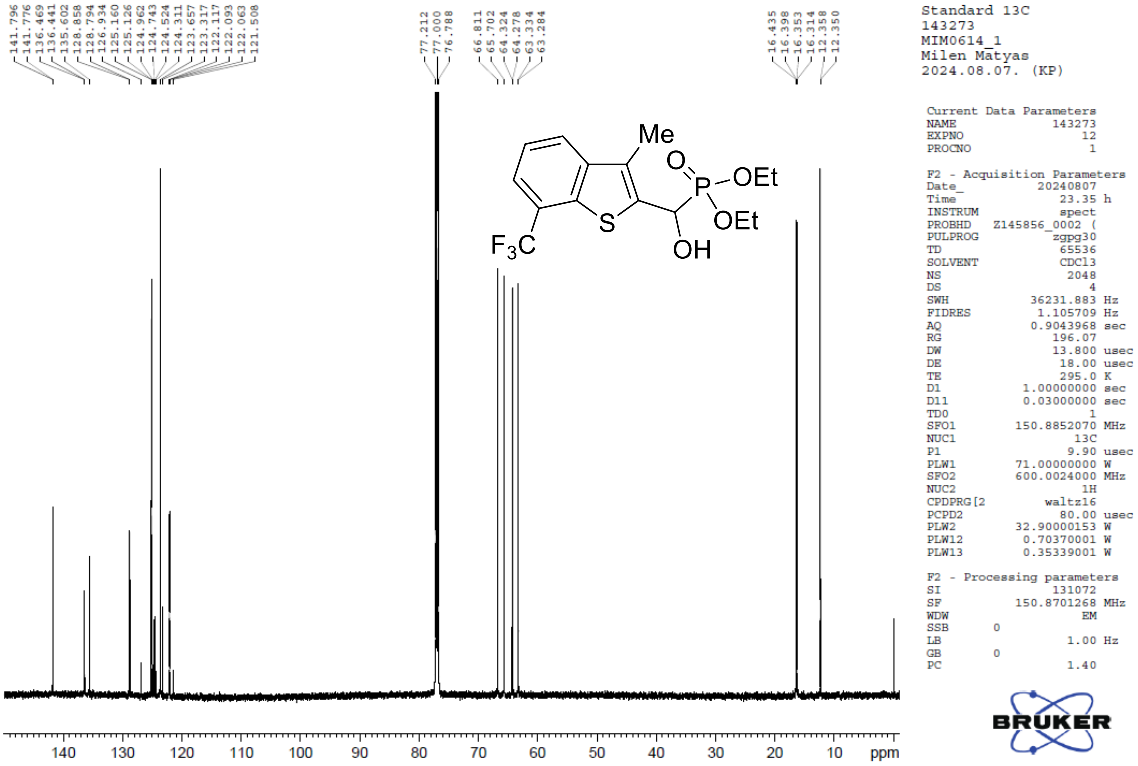

^{1}H NMR (600 MHz, $CDCl_3$) spectra for compound 2u

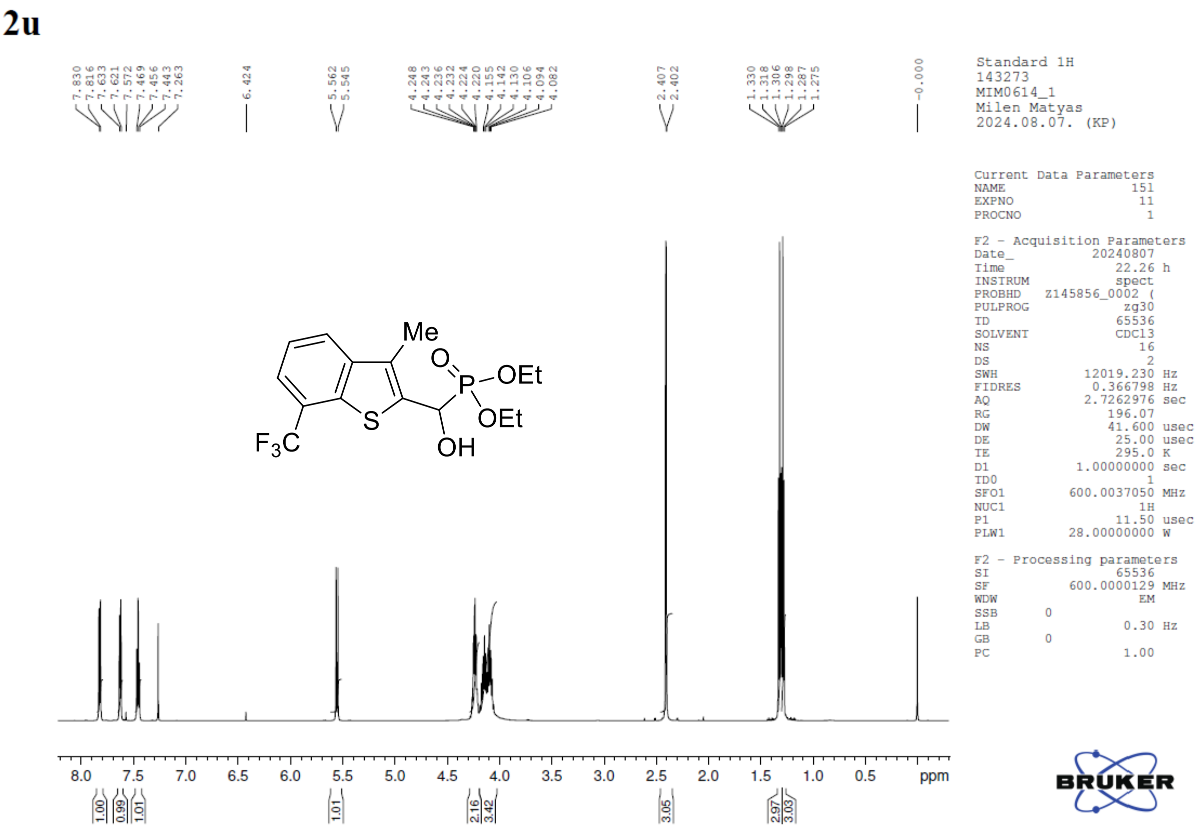

IR (KBr) spectra for compound 2u

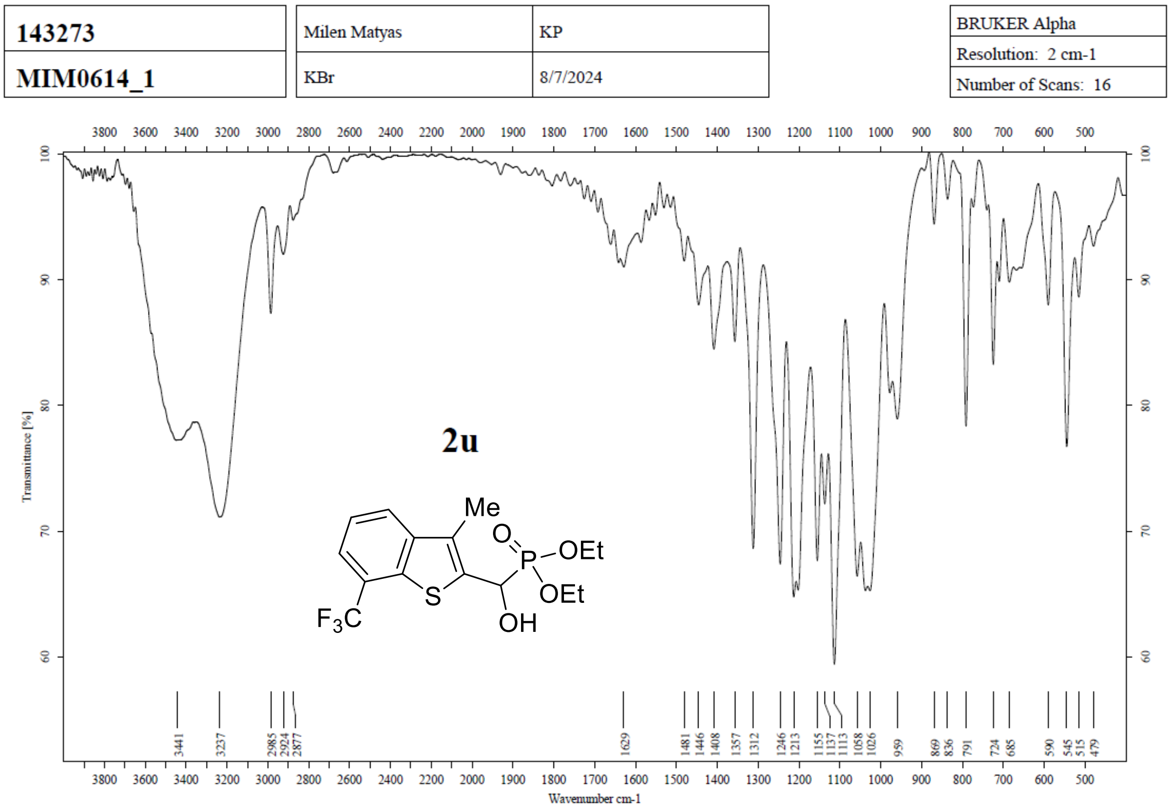

^{31}P NMR (242 MHz, $CDCl_3$) spectra for compound 2v

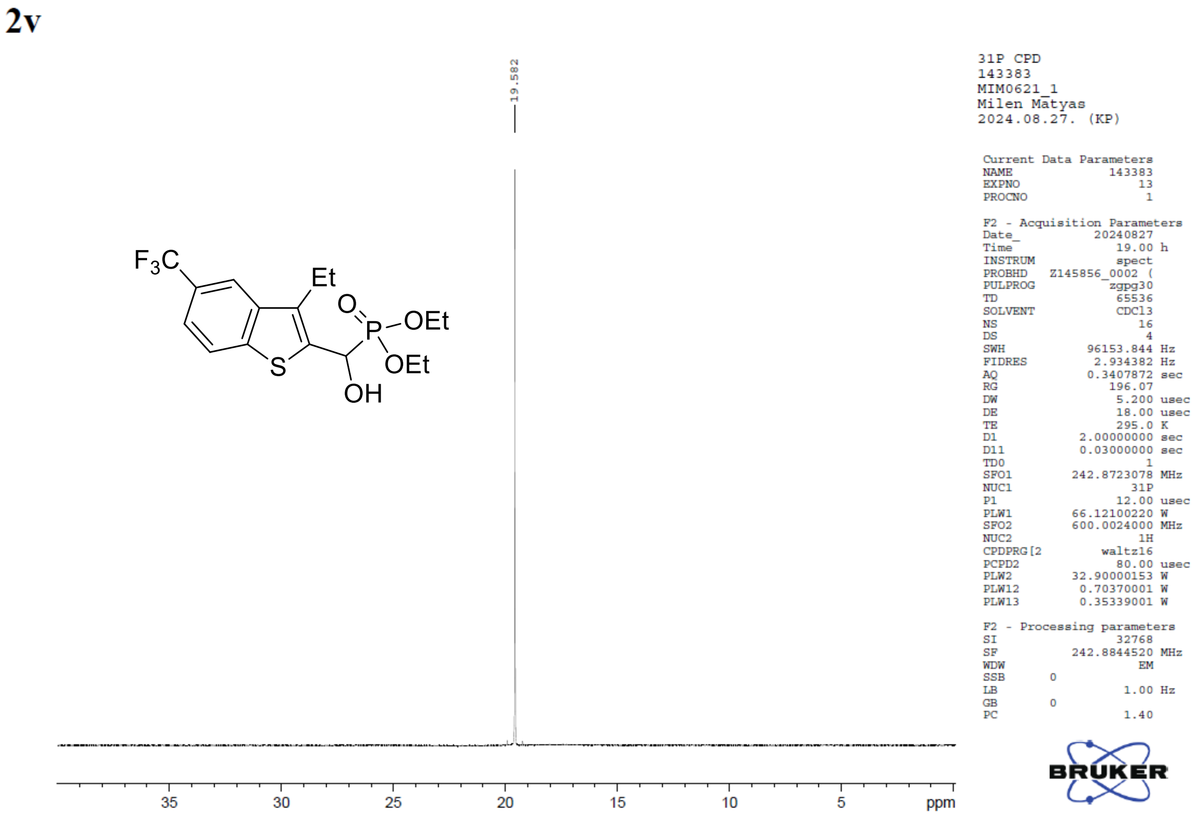

^{13}C NMR (150 MHz, $CDCl_3$) spectra for compound 2v

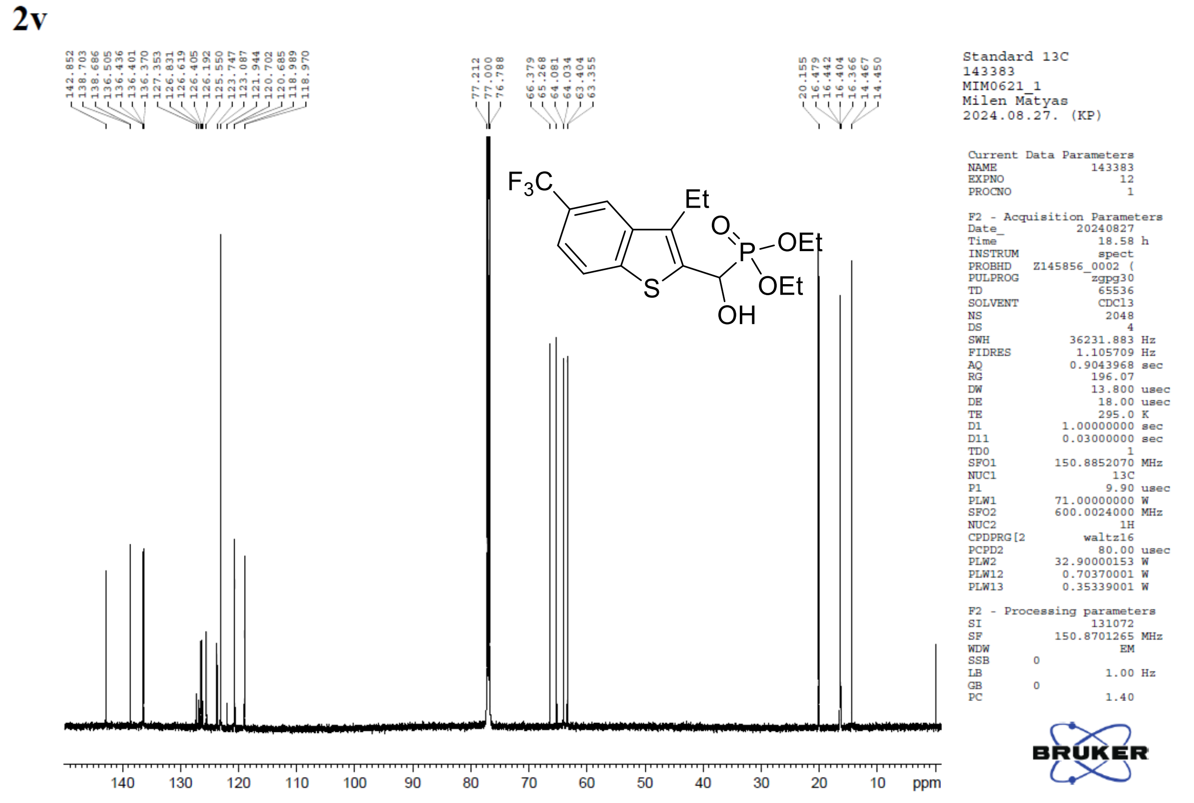

^{1}H NMR (600 MHz, $CDCl_3$) spectra for compound 2v

2v

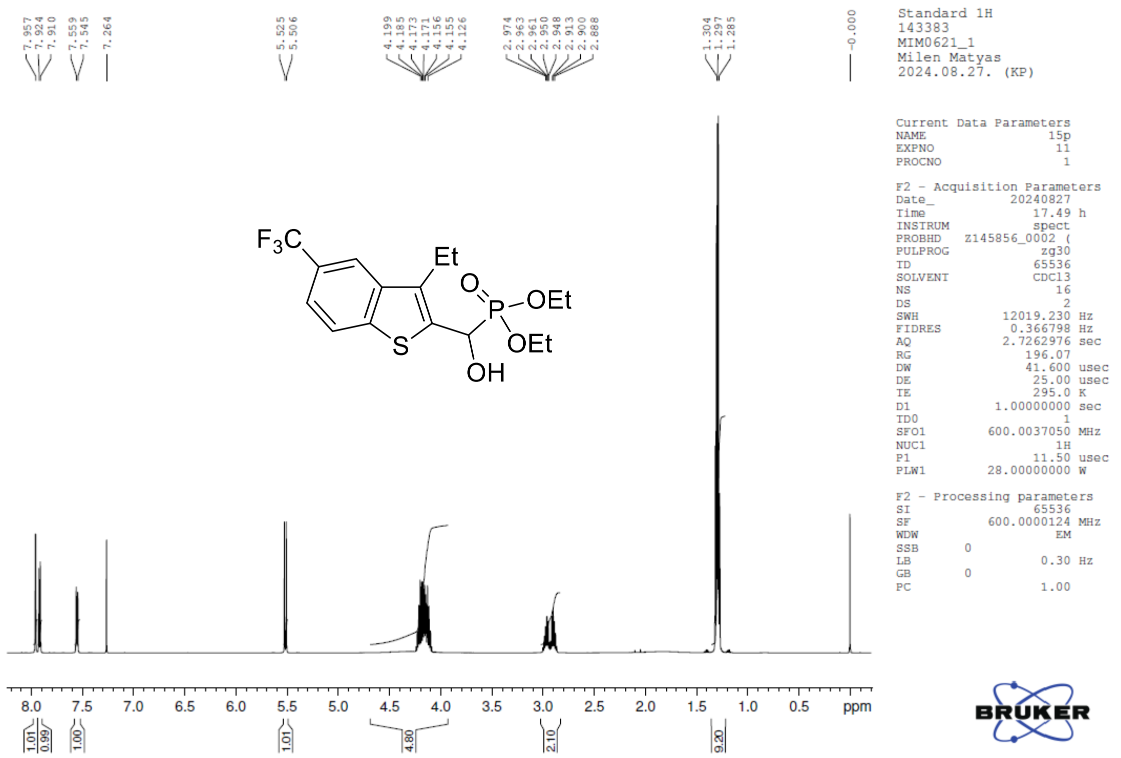

IR (KBr) spectra for compound 2v

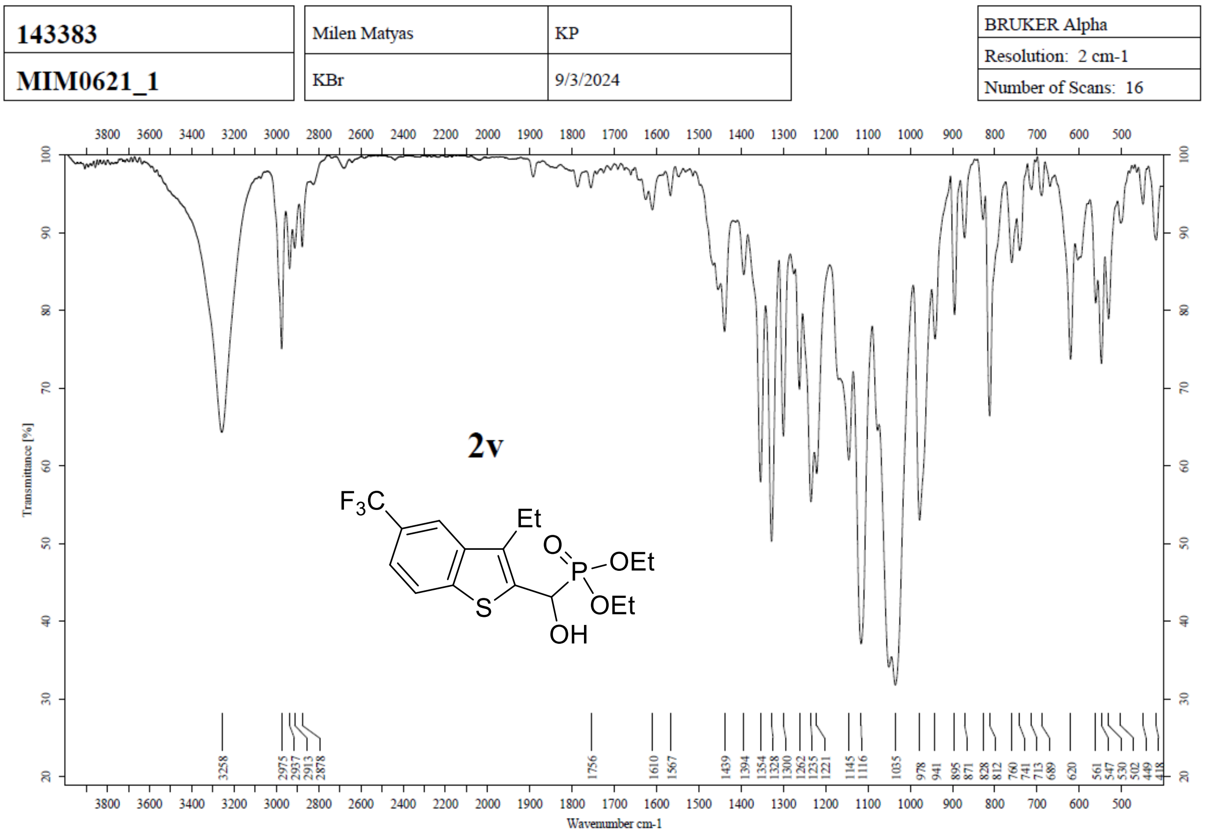

^{31}P NMR (242 MHz, $CDCl_3$) spectra for compound 2w

2w

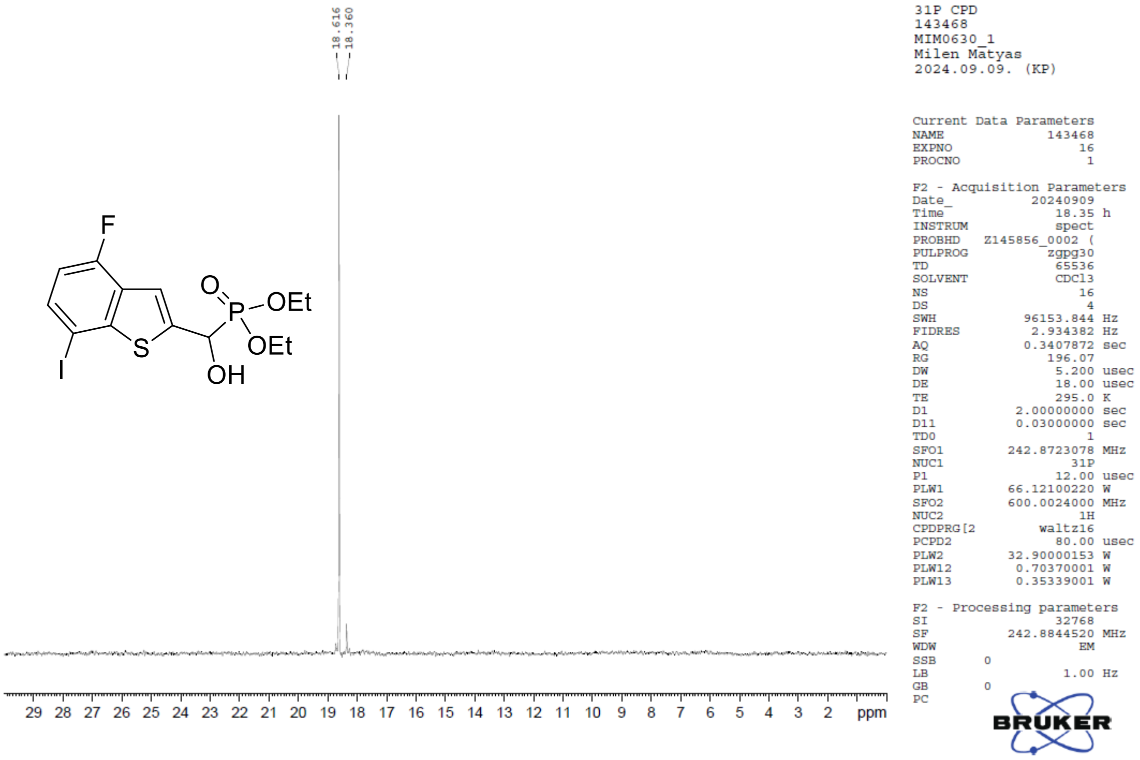

^{13}C NMR (150 MHz, $CDCl_3$) spectra for compound 2w

2w

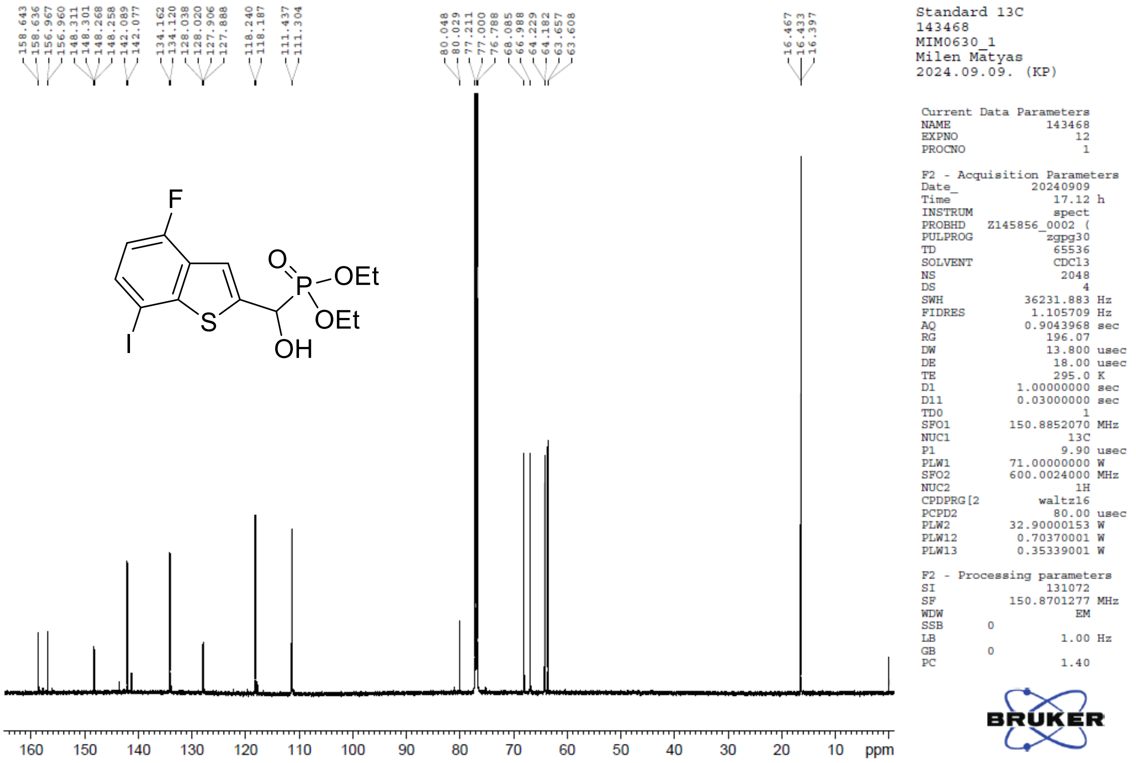

^{1}H NMR (600 MHz, CDCl$_3$) spectra for compound 2w

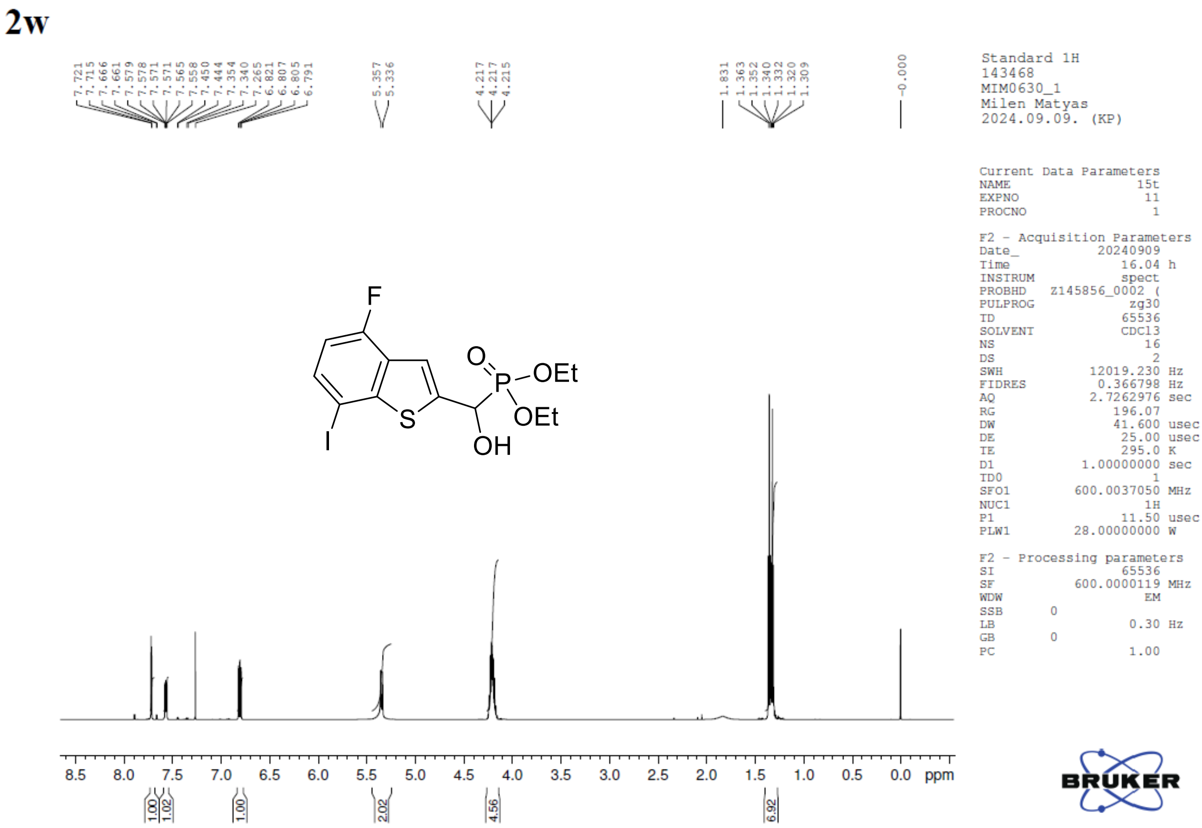

IR (KBr) spectra for compound 2w

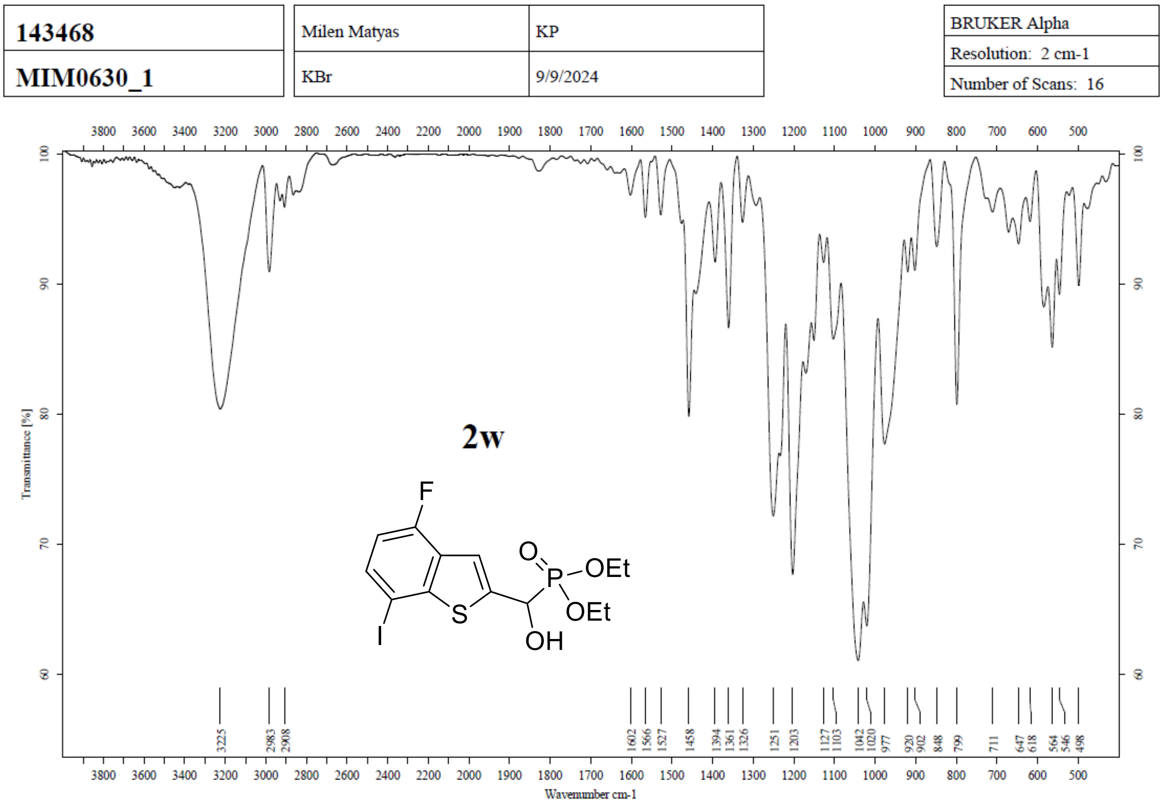

3. HSQC, HMBC and COSY NMR spectra for the compounds synthesized

HSQC NMR (140 Hz) spectra for compound 2a

2a

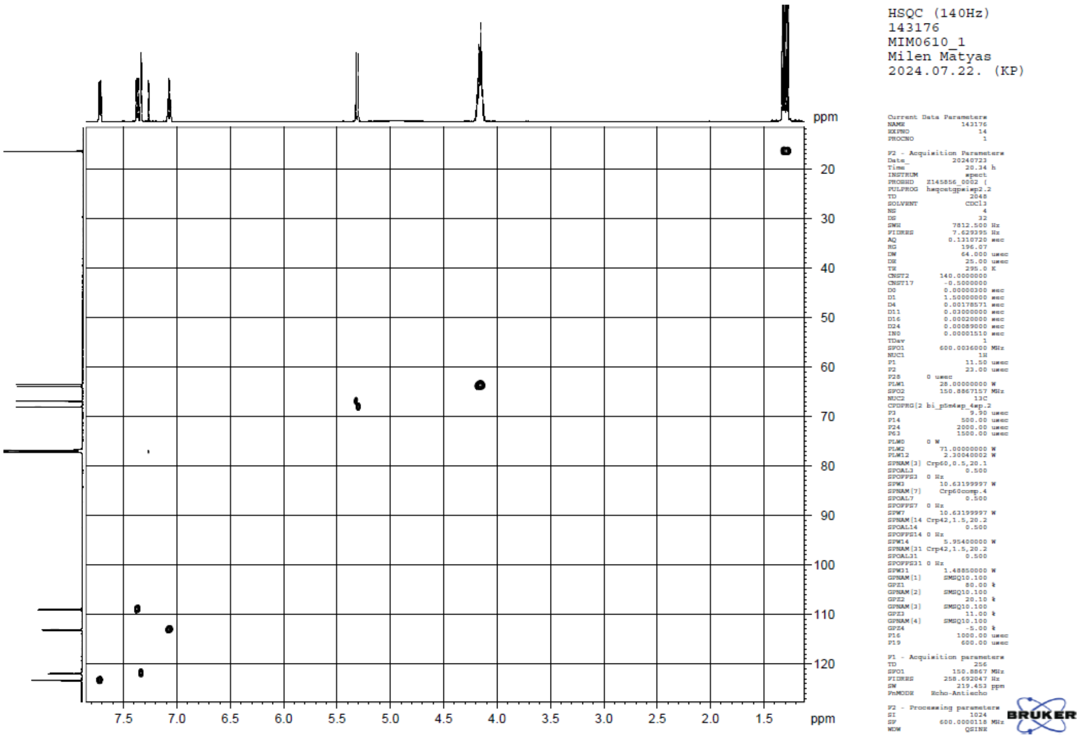

HMBC NMR (8 Hz, 140 Hz) spectra for compound 2a

2a

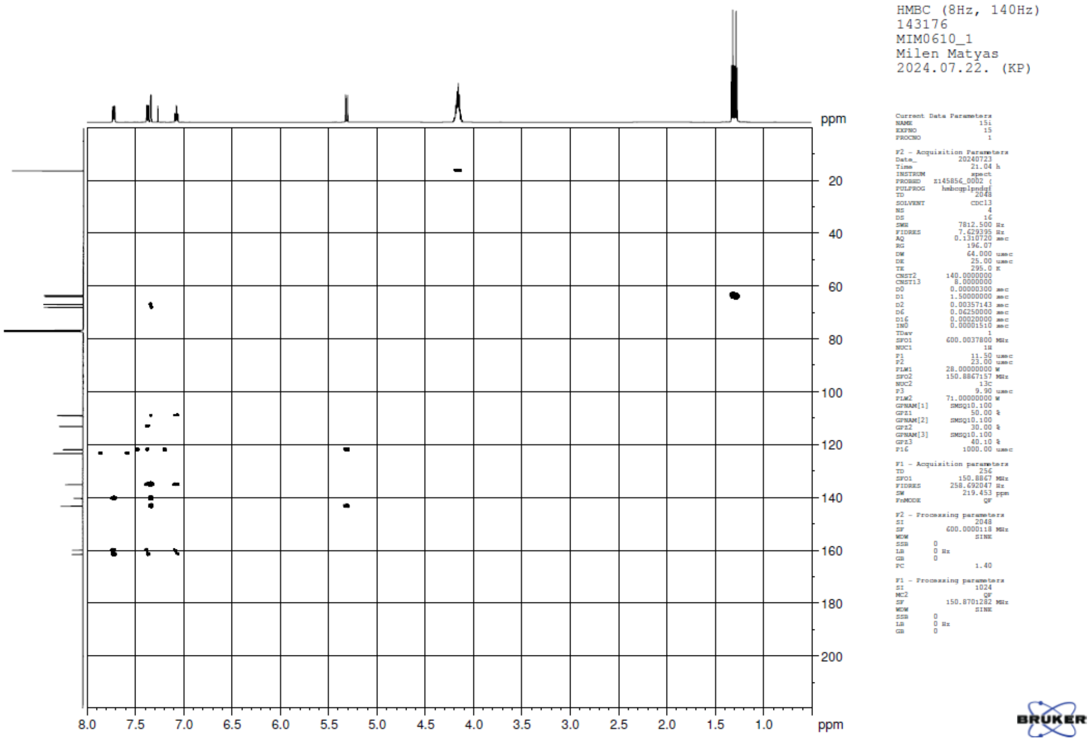

HSQC NMR (140 Hz) spectra for compound 2b

2b

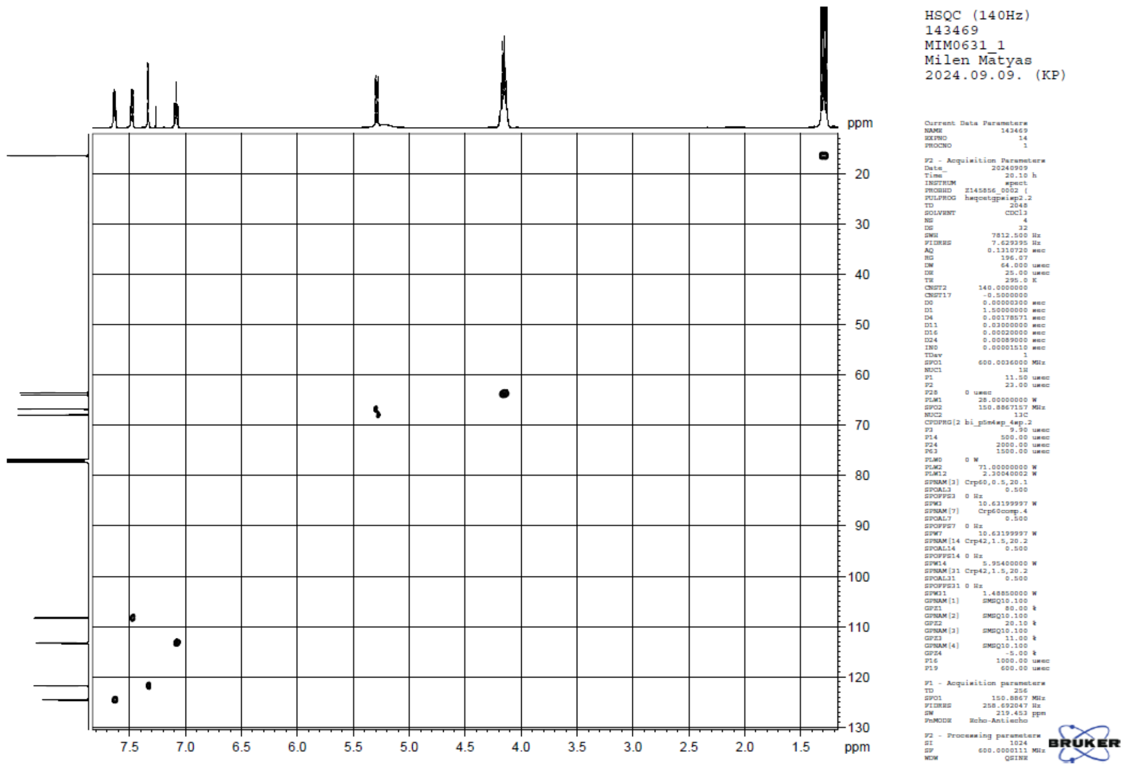

HMBC NMR (8 Hz, 140 Hz) spectra for compound 2b

2b

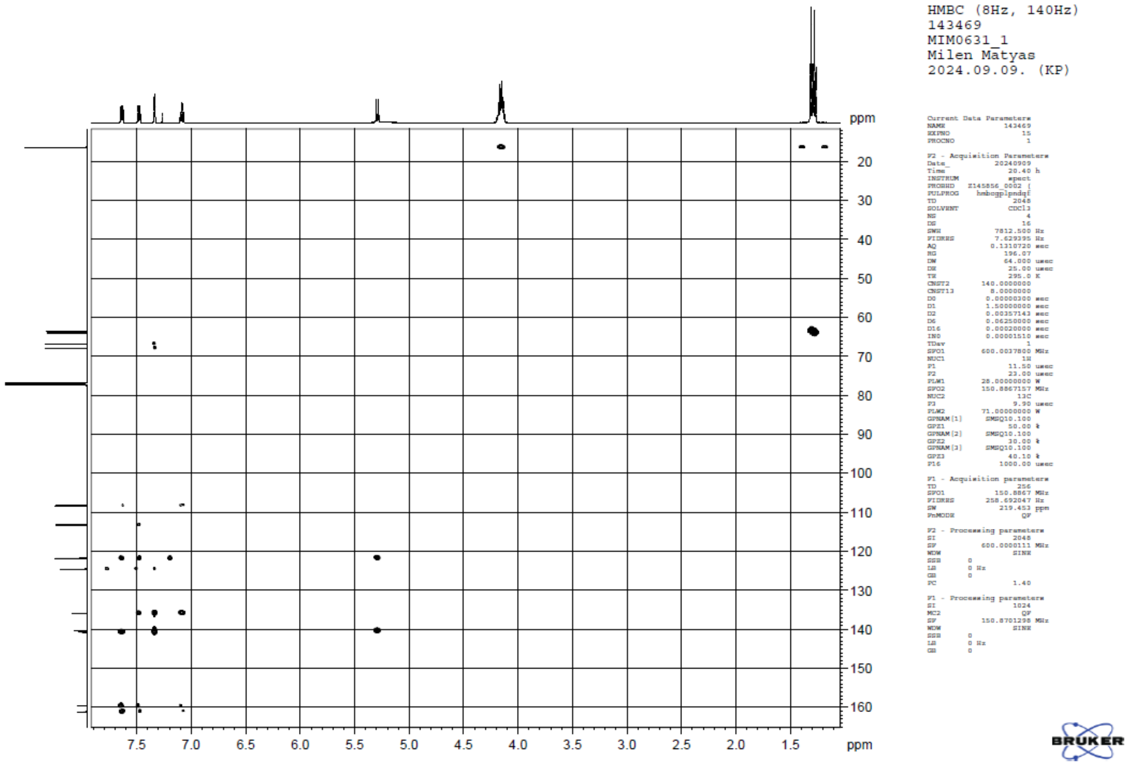

COSY NMR spectra for compound 2b

2b

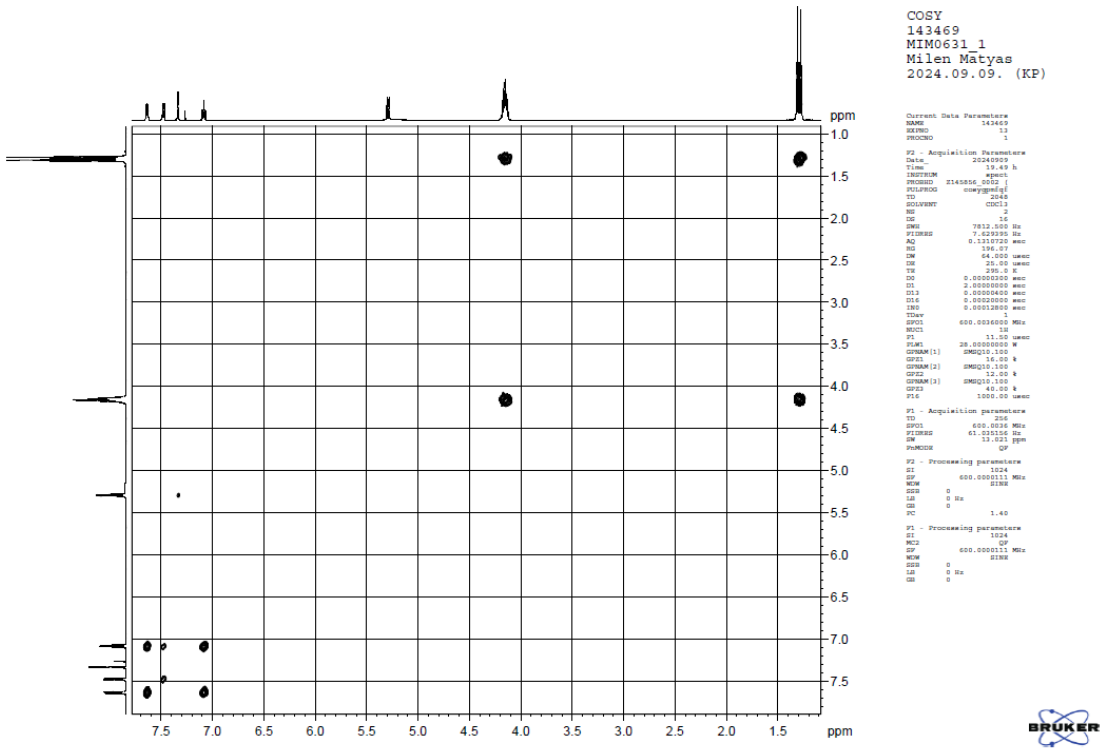

HSQC NMR (140 Hz) spectra for compound 2c

2c

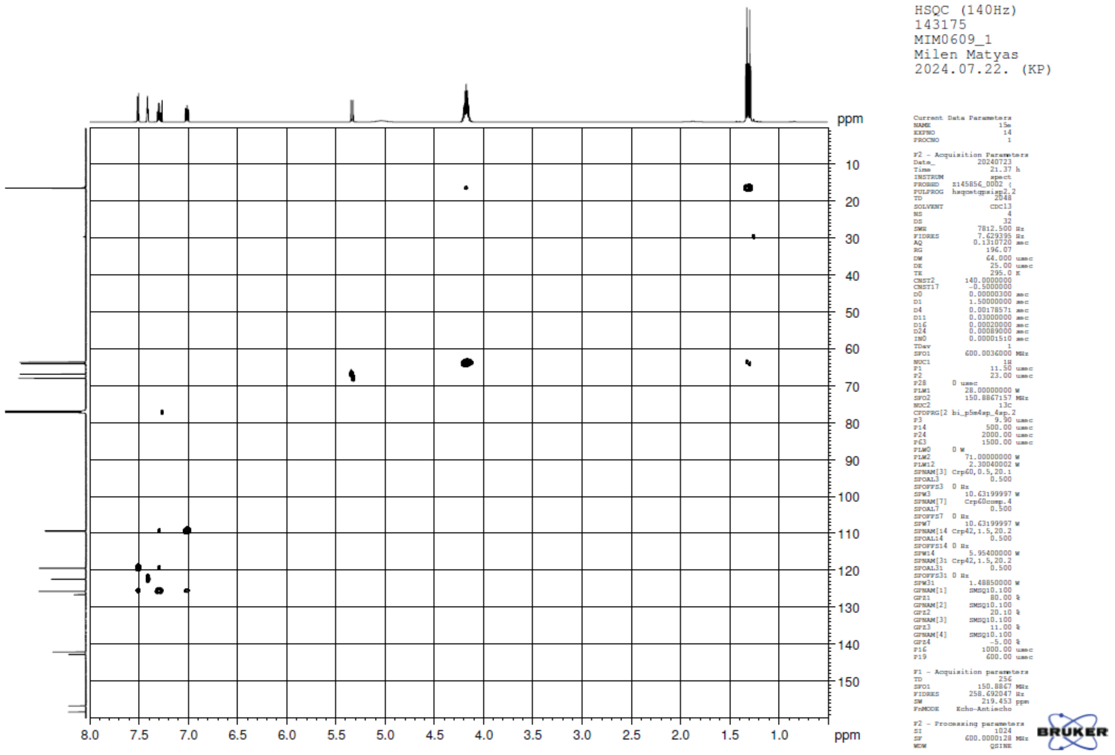

HMBC NMR (8 Hz, 140 Hz) spectra for compound 2c

2c

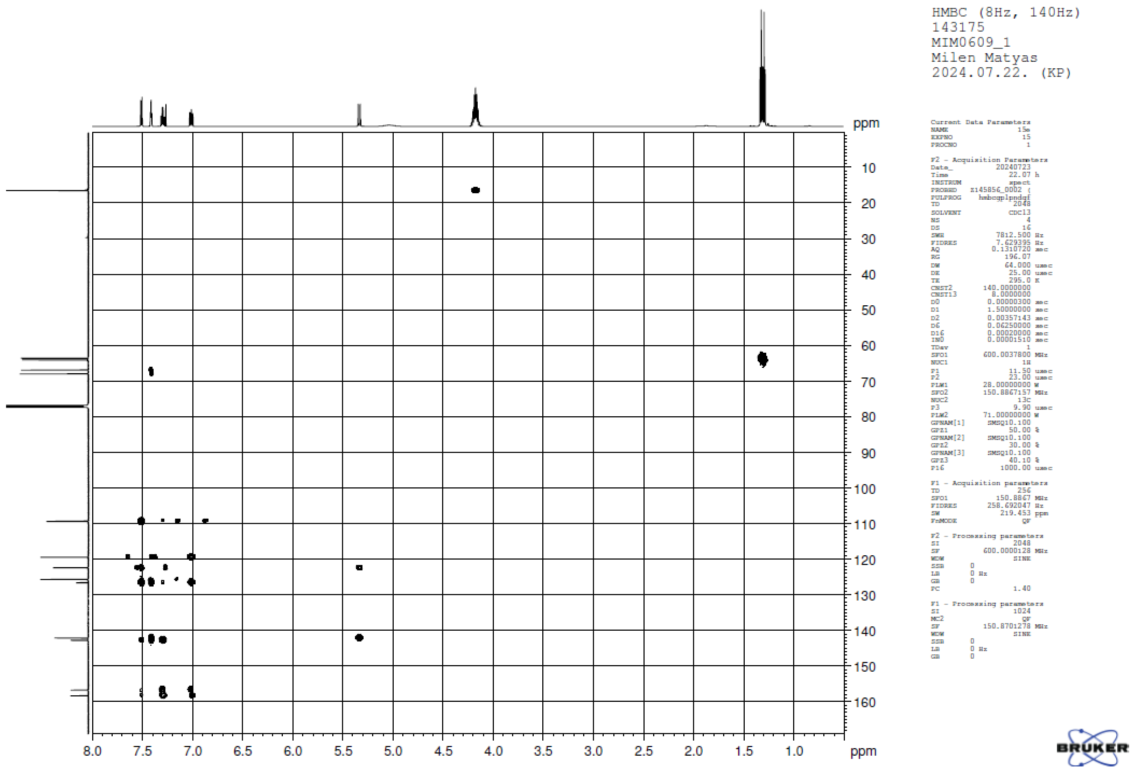

HSQC NMR (140 Hz) spectra for compound 2d

2d

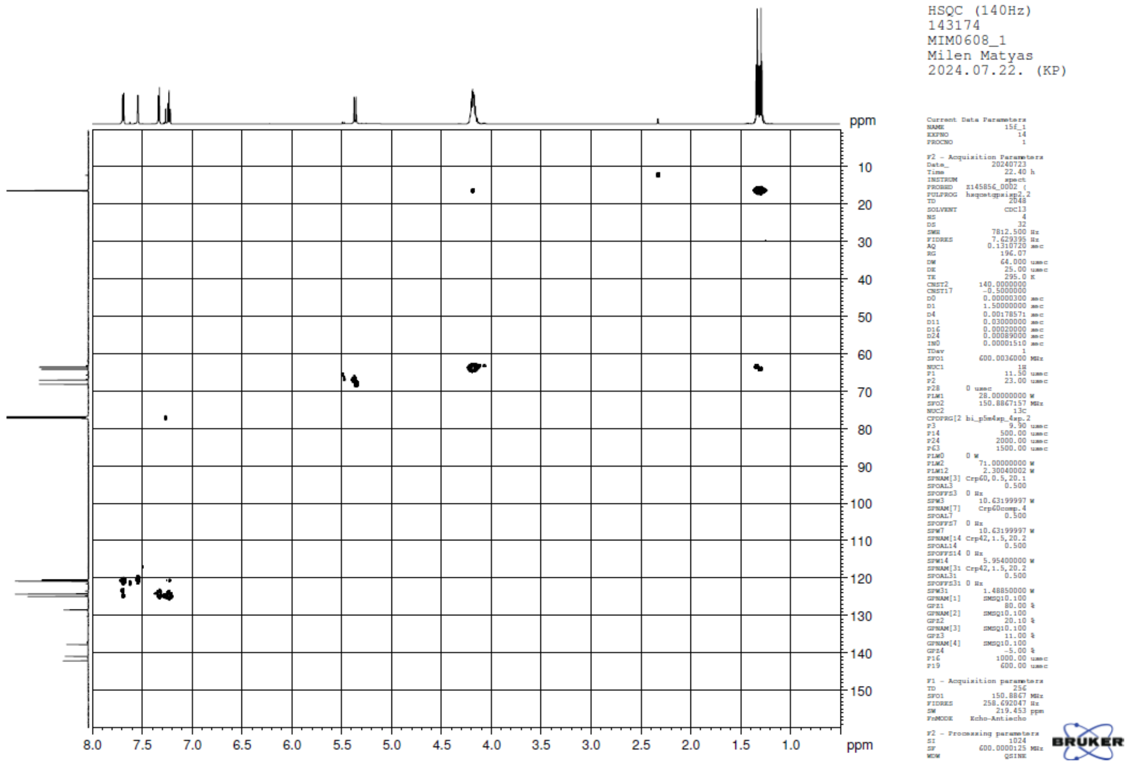

HMBC NMR (8Hz, 140 Hz) spectra for compound 2d

2d

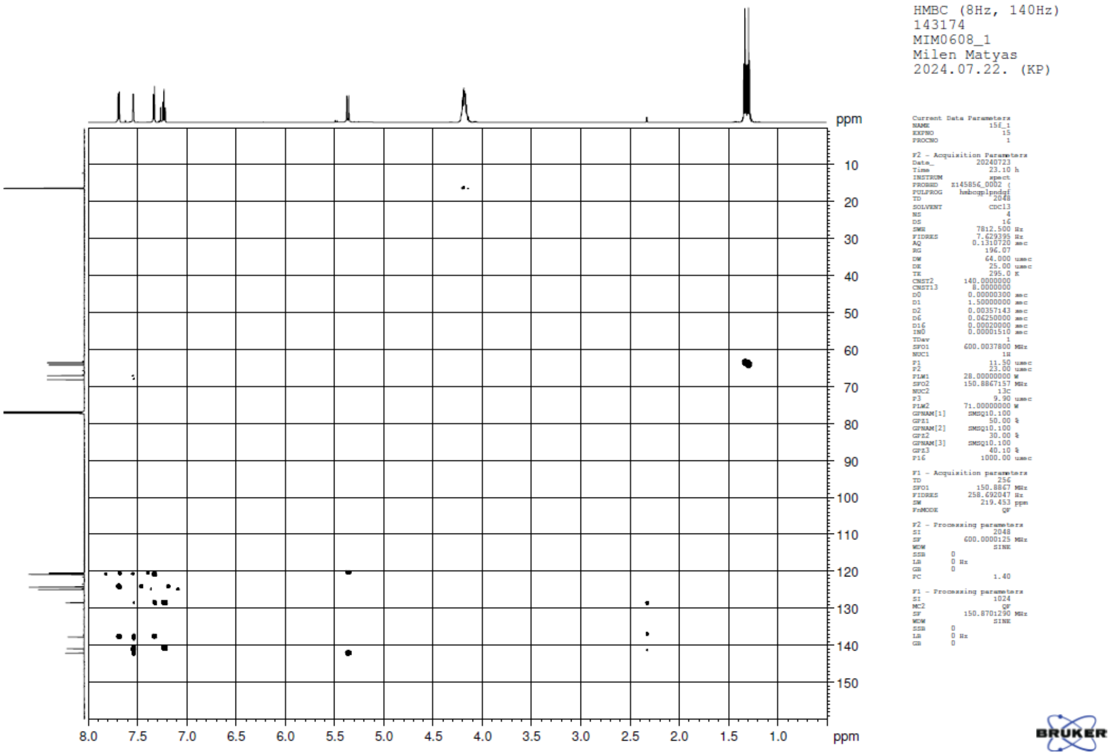

HSQC NMR (140 Hz) spectra for compound 2e

2e

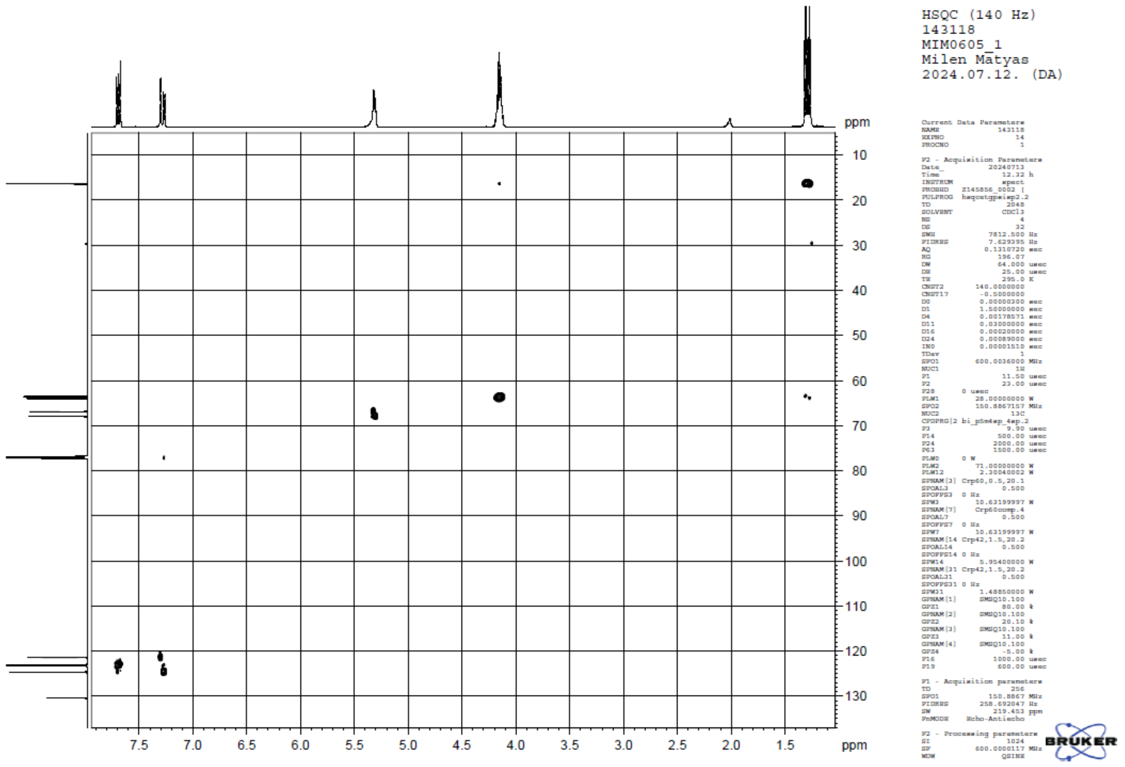

HMBC NMR (8 Hz, 140 Hz) spectra for compound 2e

2e

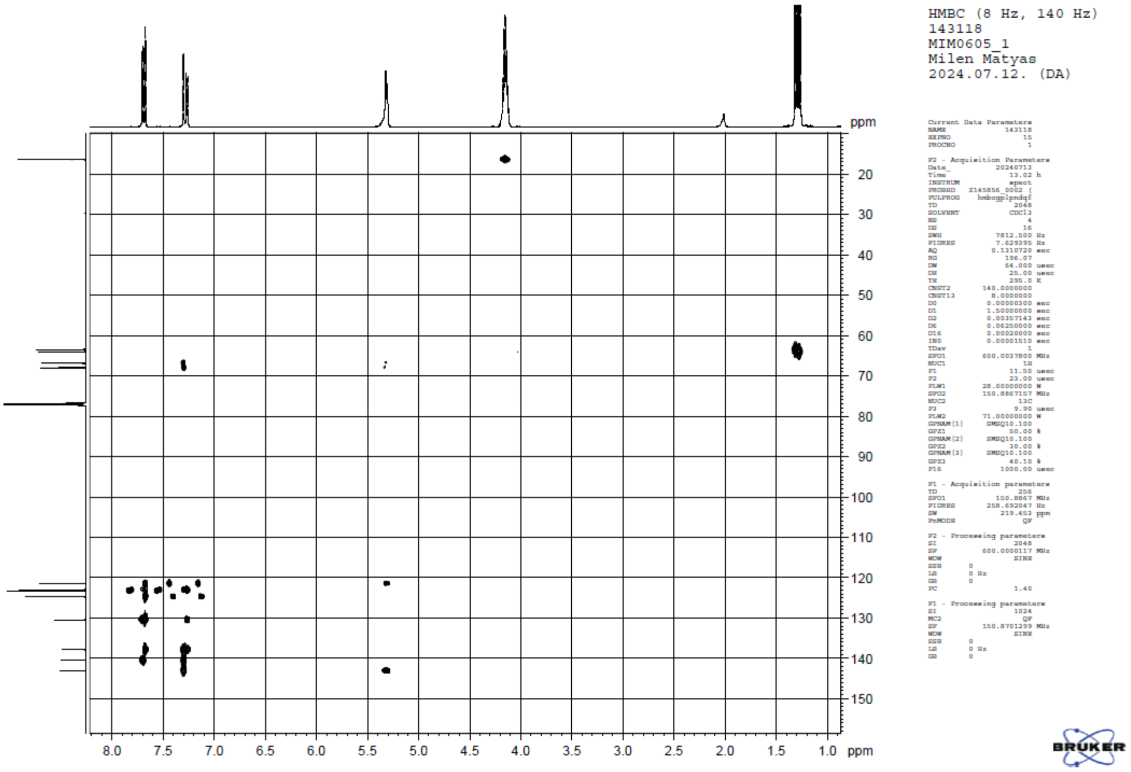

COSY NMR spectra for compound 2e

2e

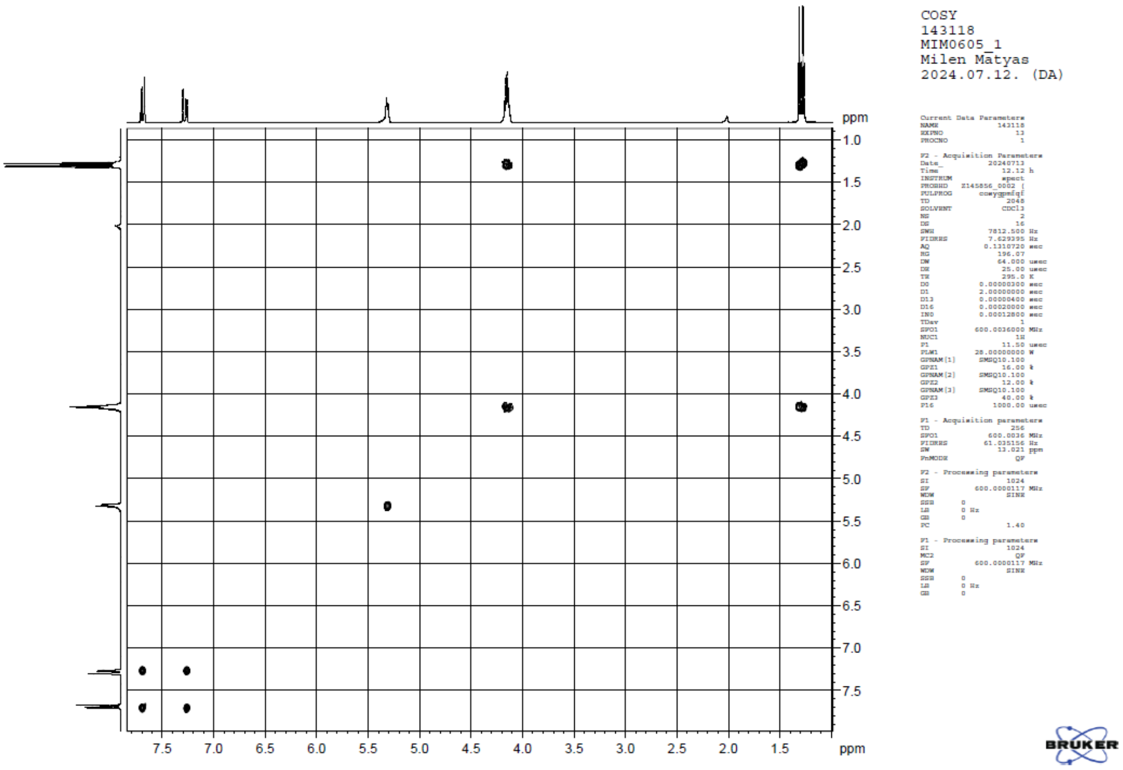

HSQC NMR (140 Hz) spectra for compound 2f

2f

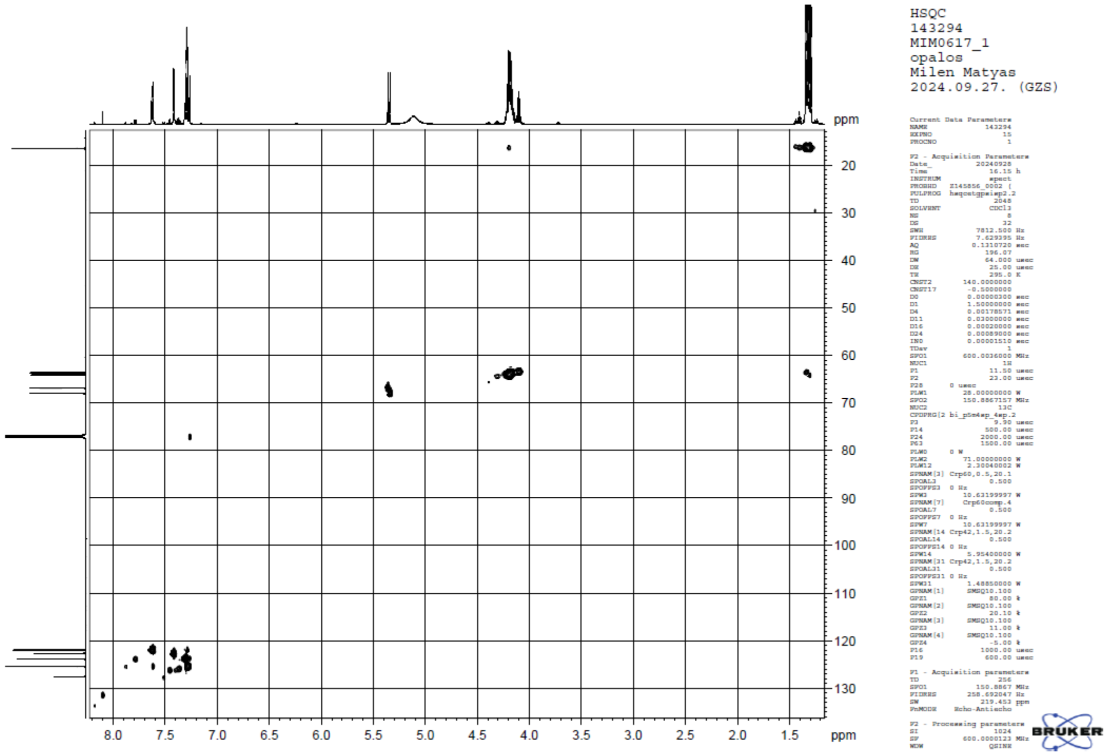

HMBC NMR (8Hz, 140 Hz) spectra for compound 2f

2f

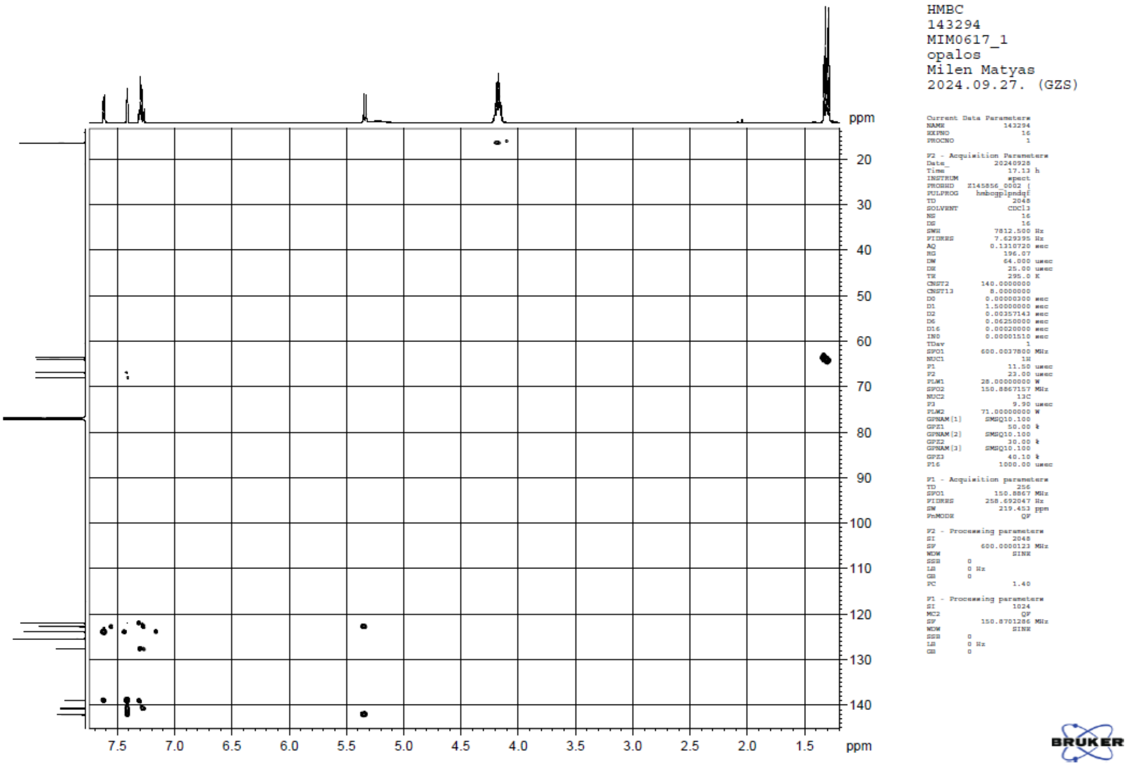

HSQC NMR (140 Hz) spectra for compound 2g

2g

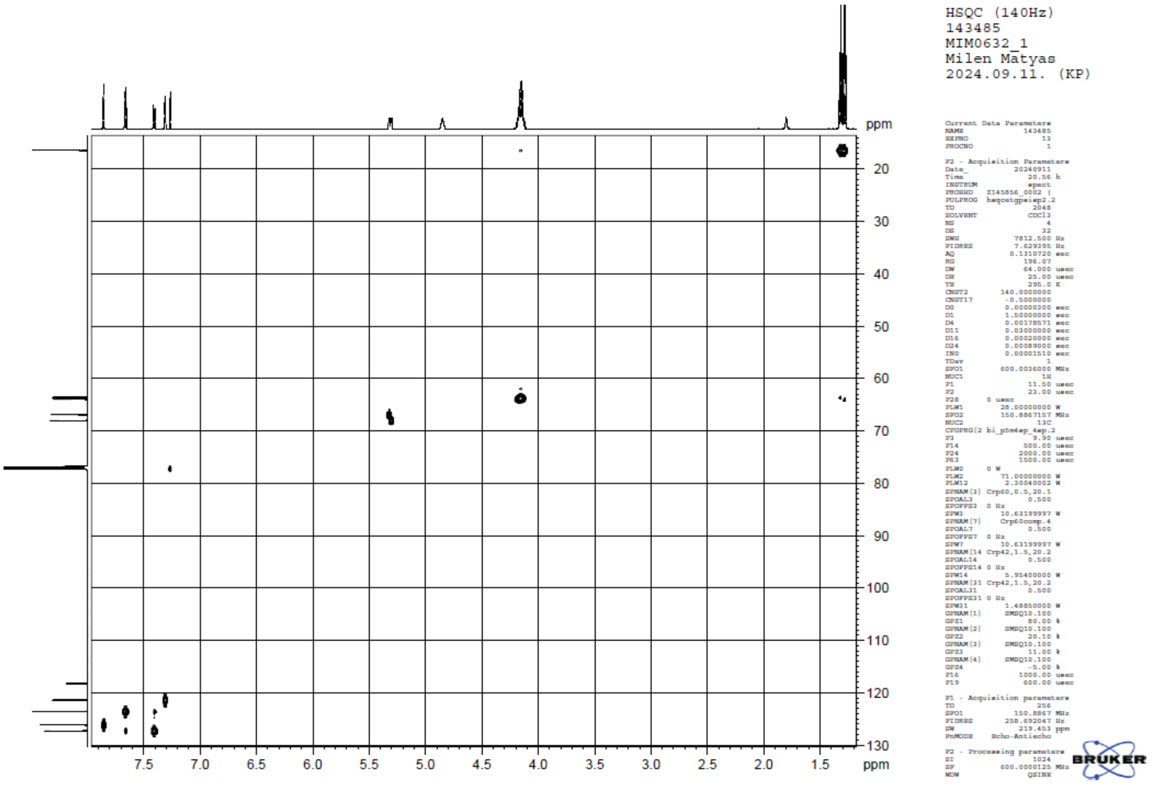

HMBC NMR (8 Hz, 140 Hz) spectra for compound 2g

2g

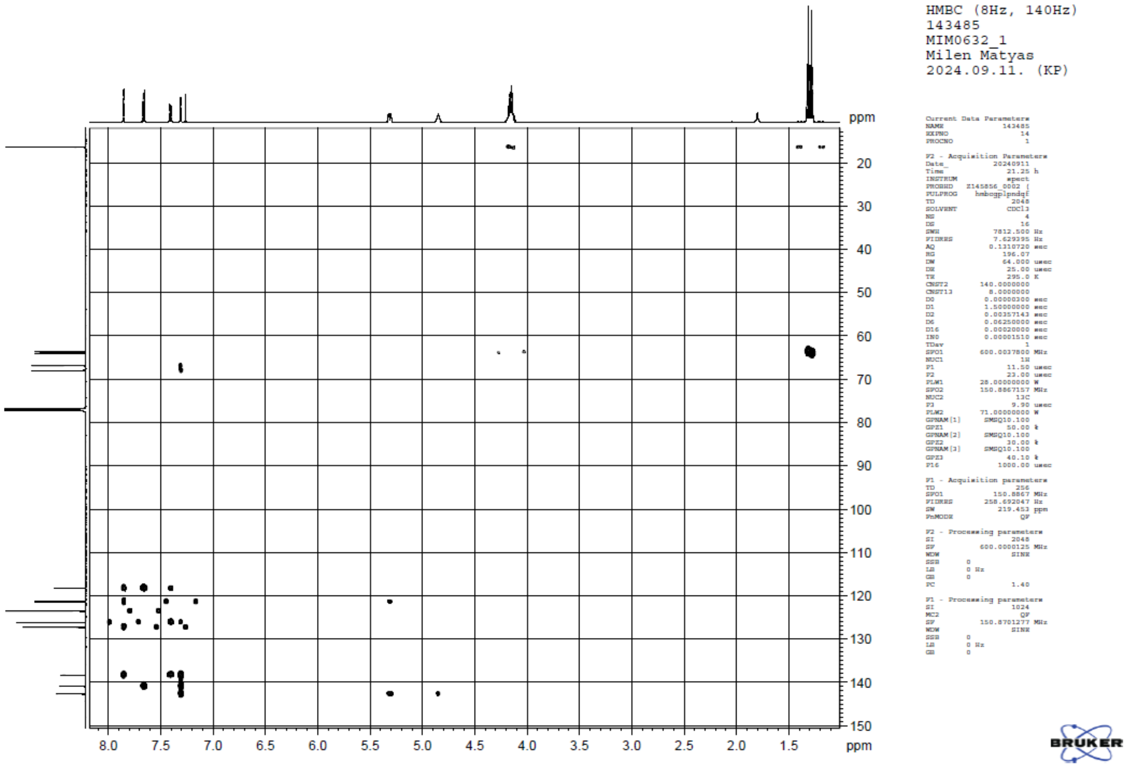

COSY spectra for compound 2g

2g

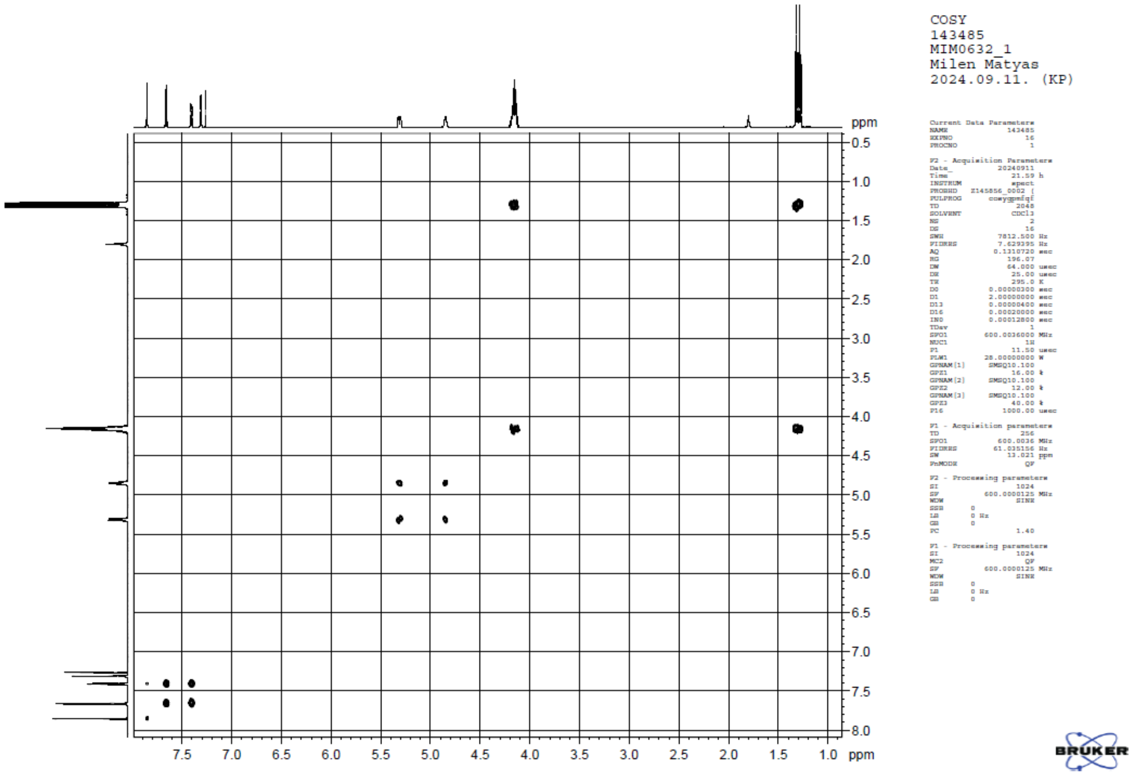

HSQC NMR (140 Hz) spectra for compound 2h

2h

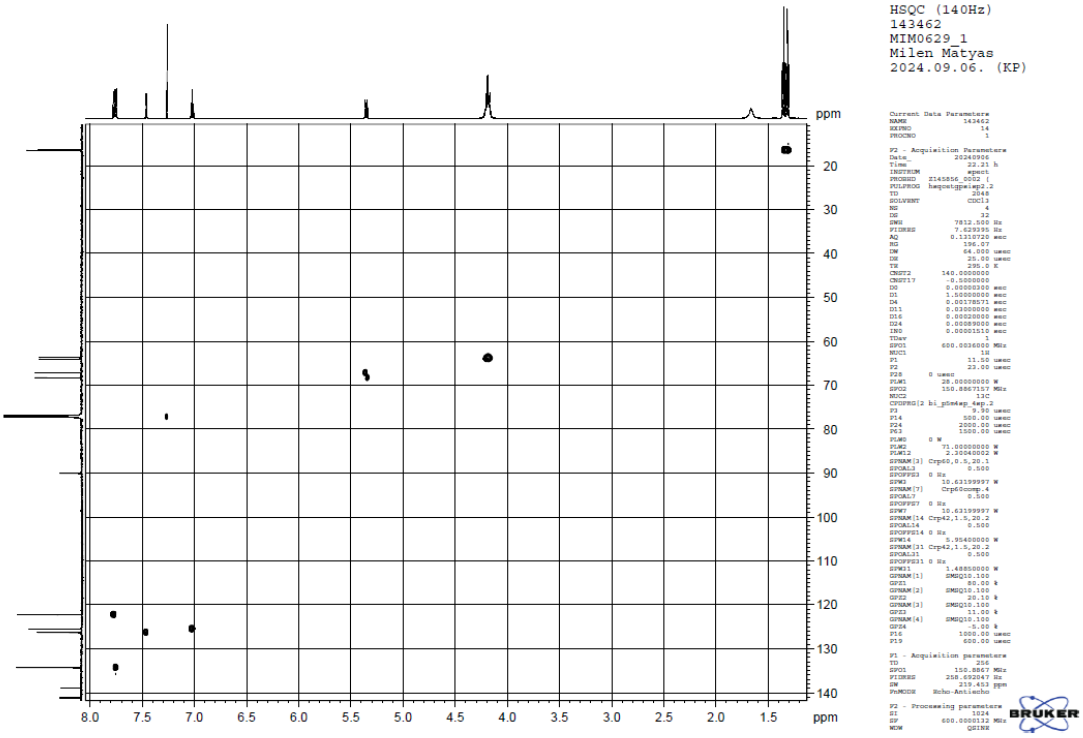

HMBC NMR (8 Hz, 140 Hz) spectra for compound 2h

2h

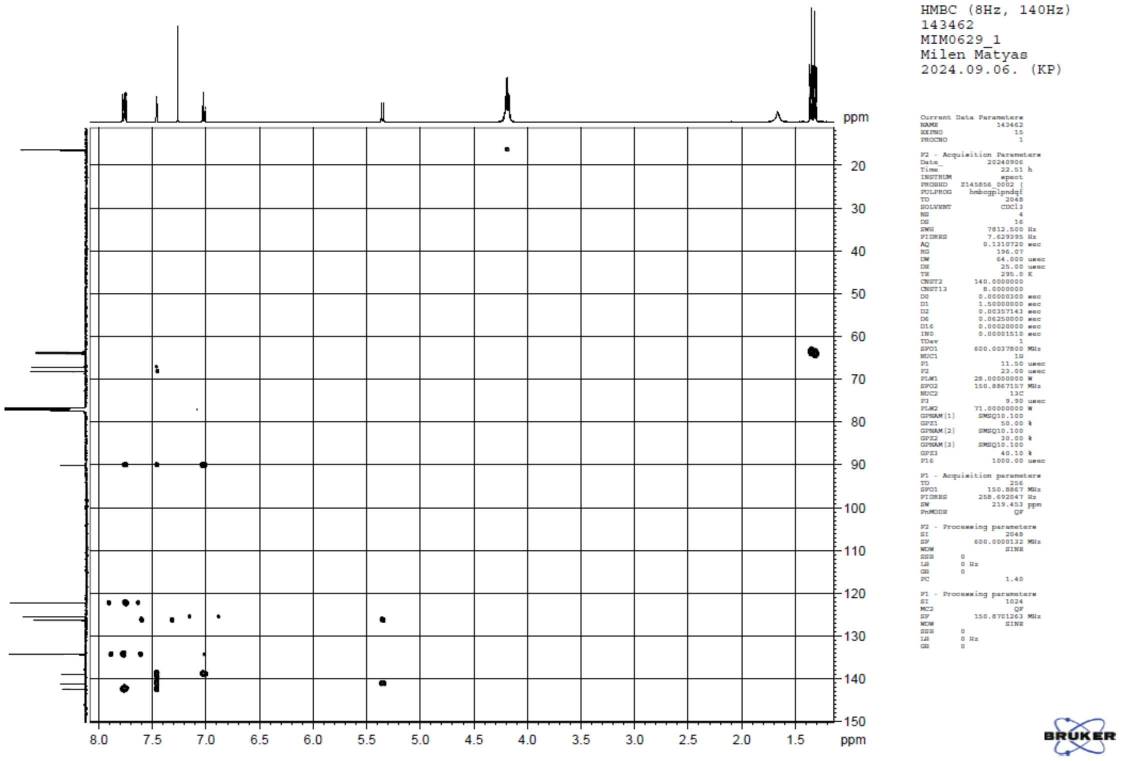

COSY NMR spectra for compound 2h

2h

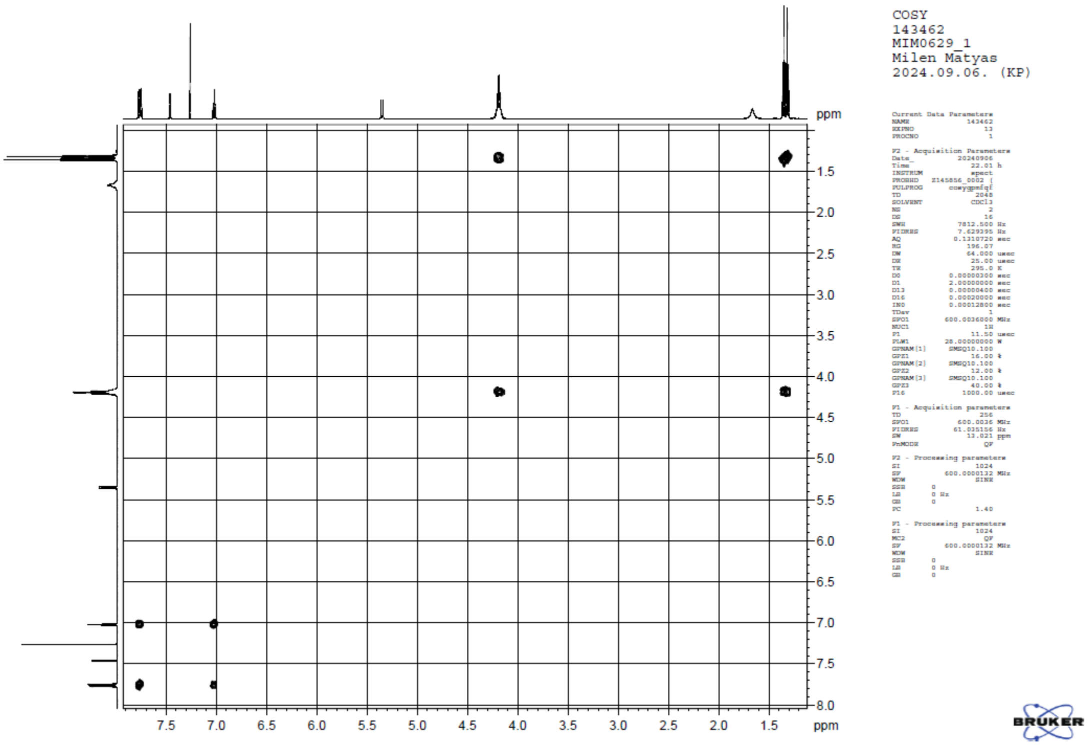

HSQC NMR (140 Hz) spectra for compound 2j

2j

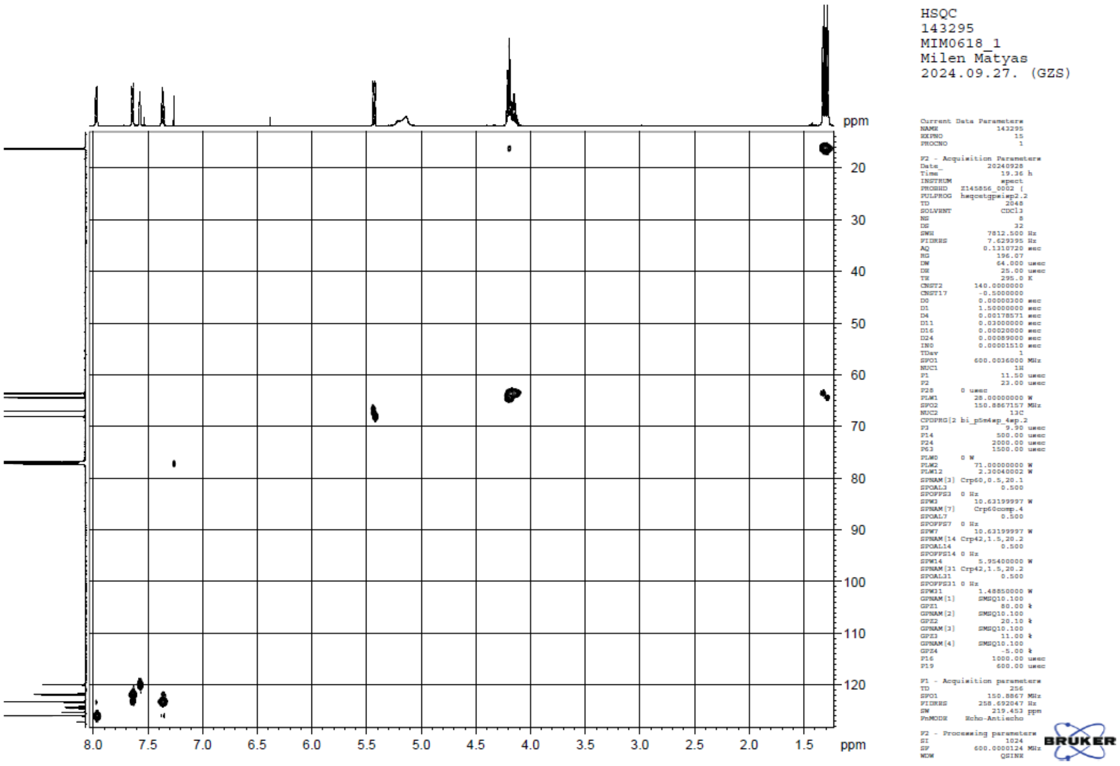

HMBC NMR (8 Hz, 140 Hz) spectra for compound 2j

2j

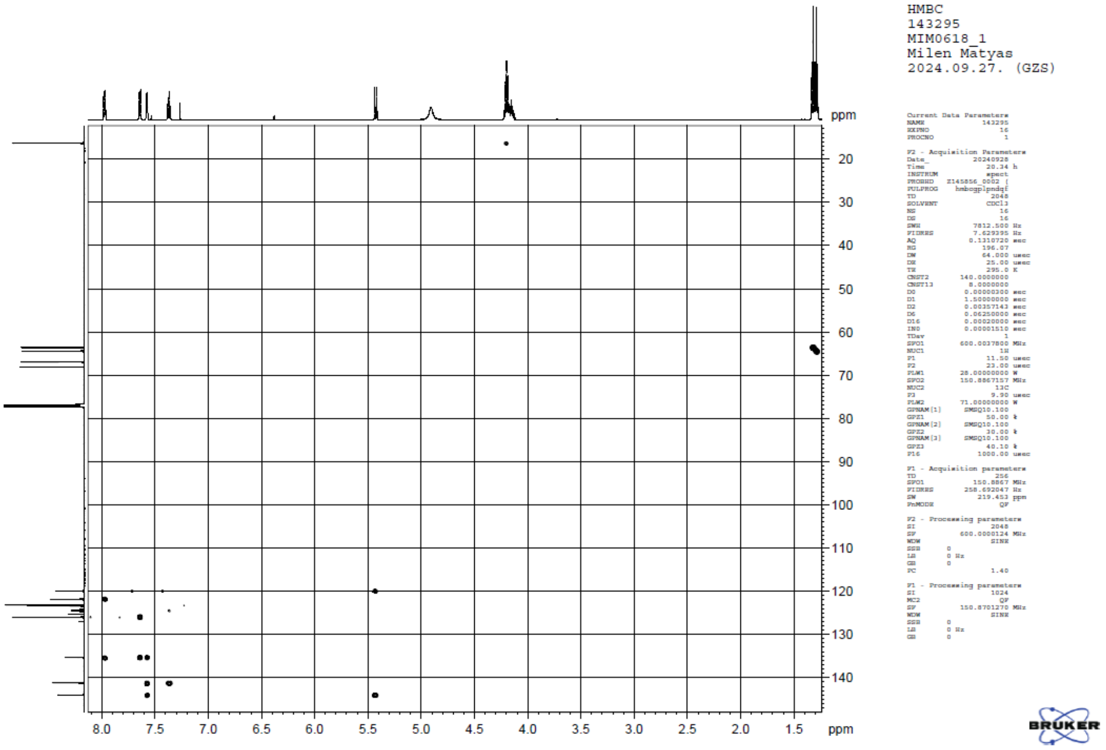

COSY NMR spectra for compound 2j

2j

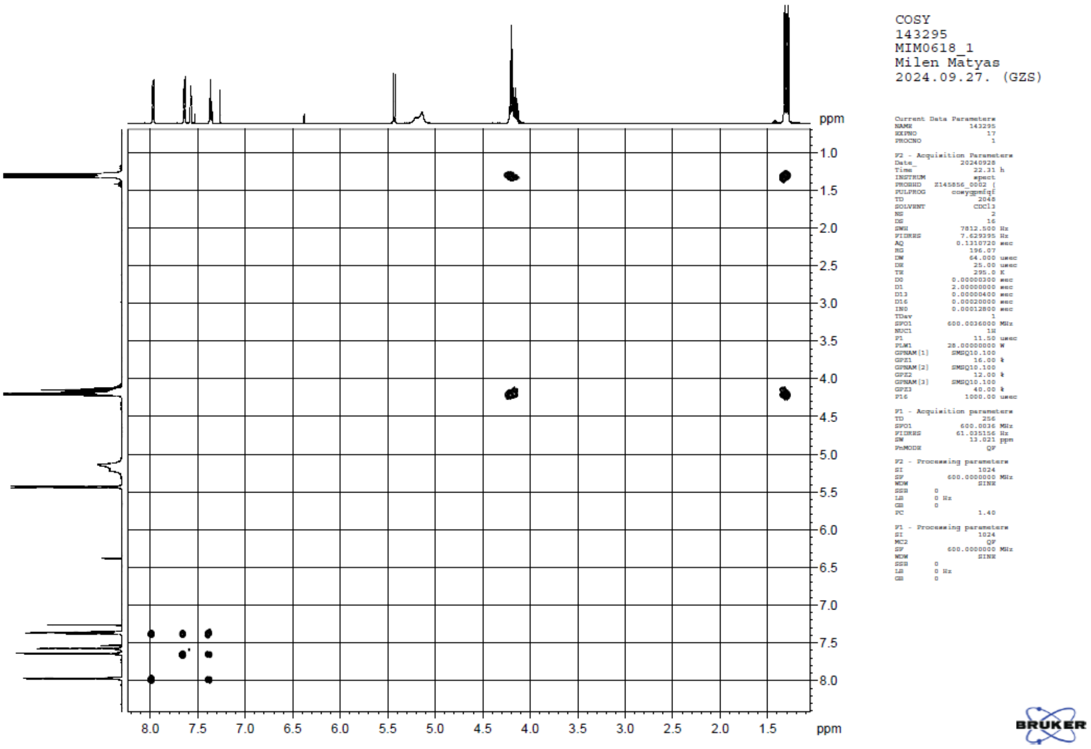

HSQC NMR (140 Hz) spectra for compound 2k

2k

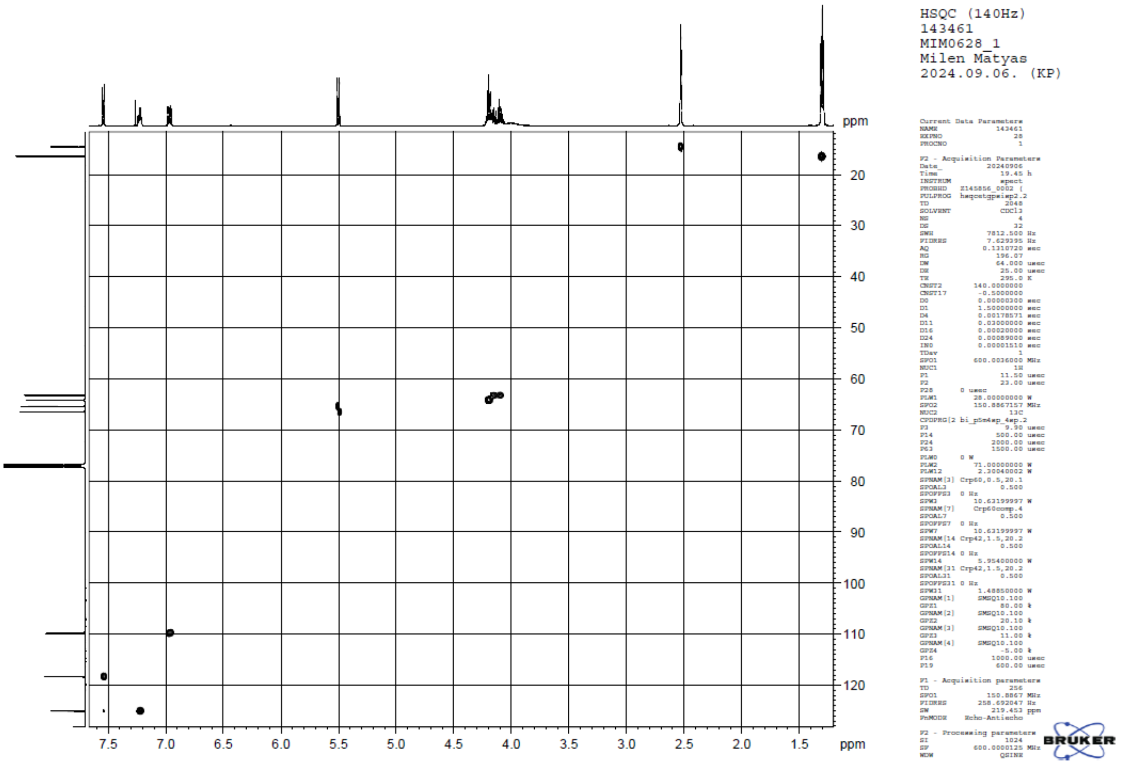

HMBC NMR (8Hz, 140 Hz) spectra for compound 2k

2k

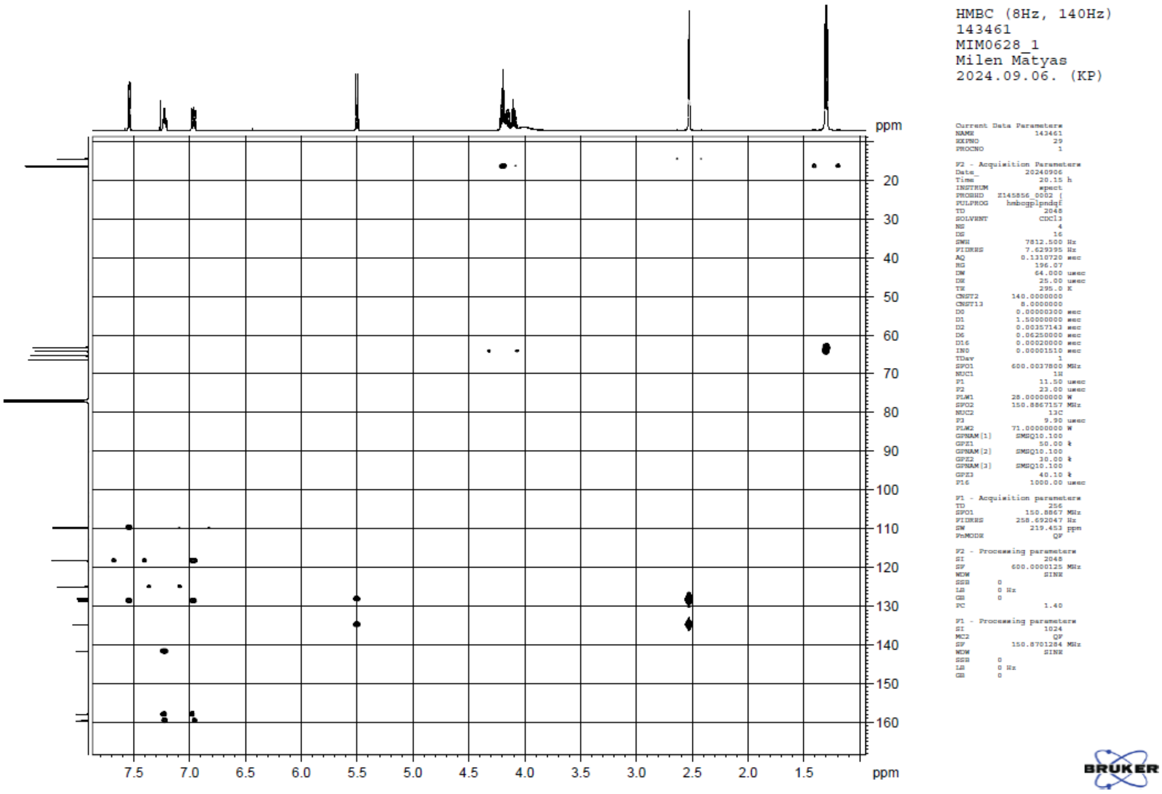

COSY NMR spectra for compound 2k

2k

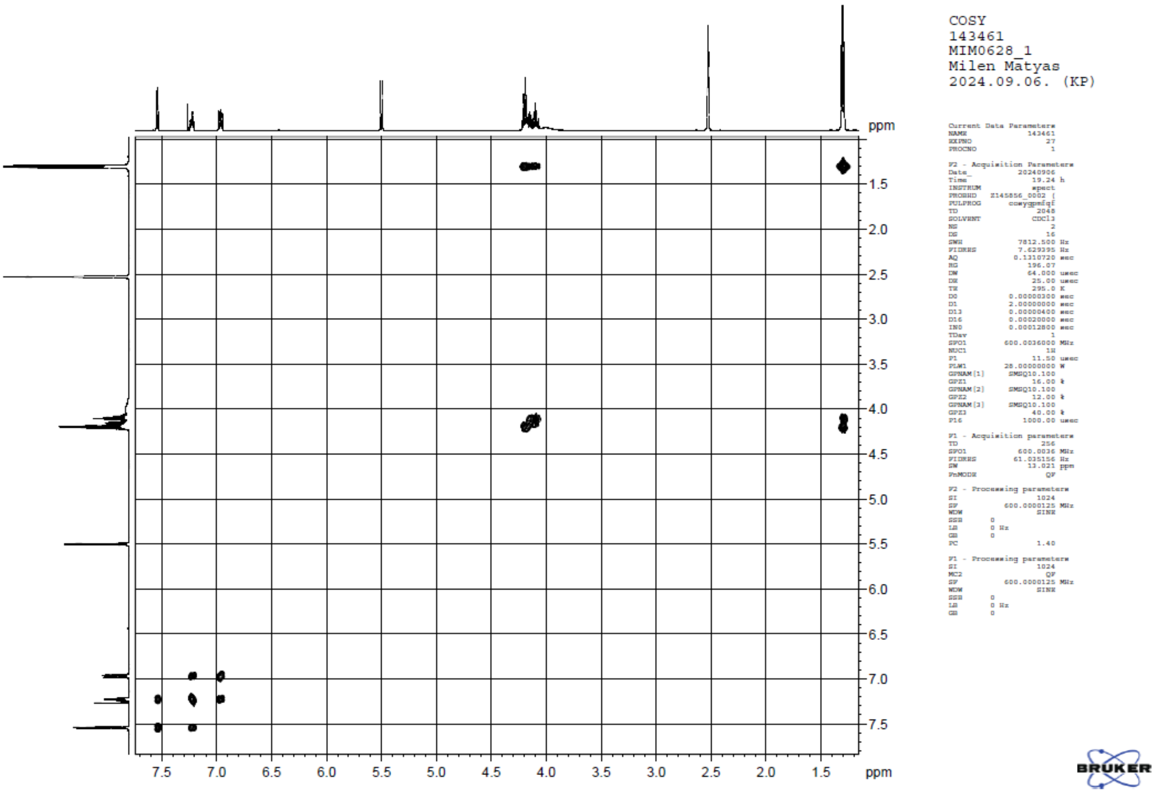

HSQC NMR (140 Hz) spectra for compound 2l

2l

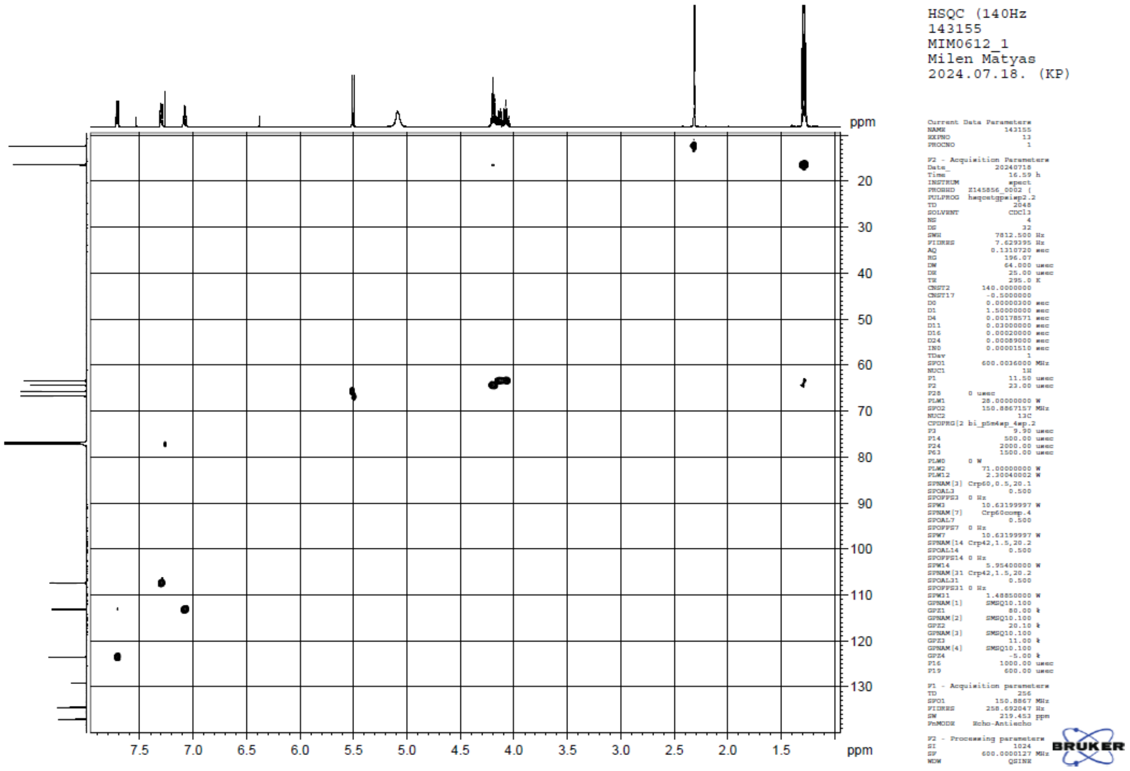

HMBC NMR (8 Hz, 140 Hz) spectra for compound 2l

2l

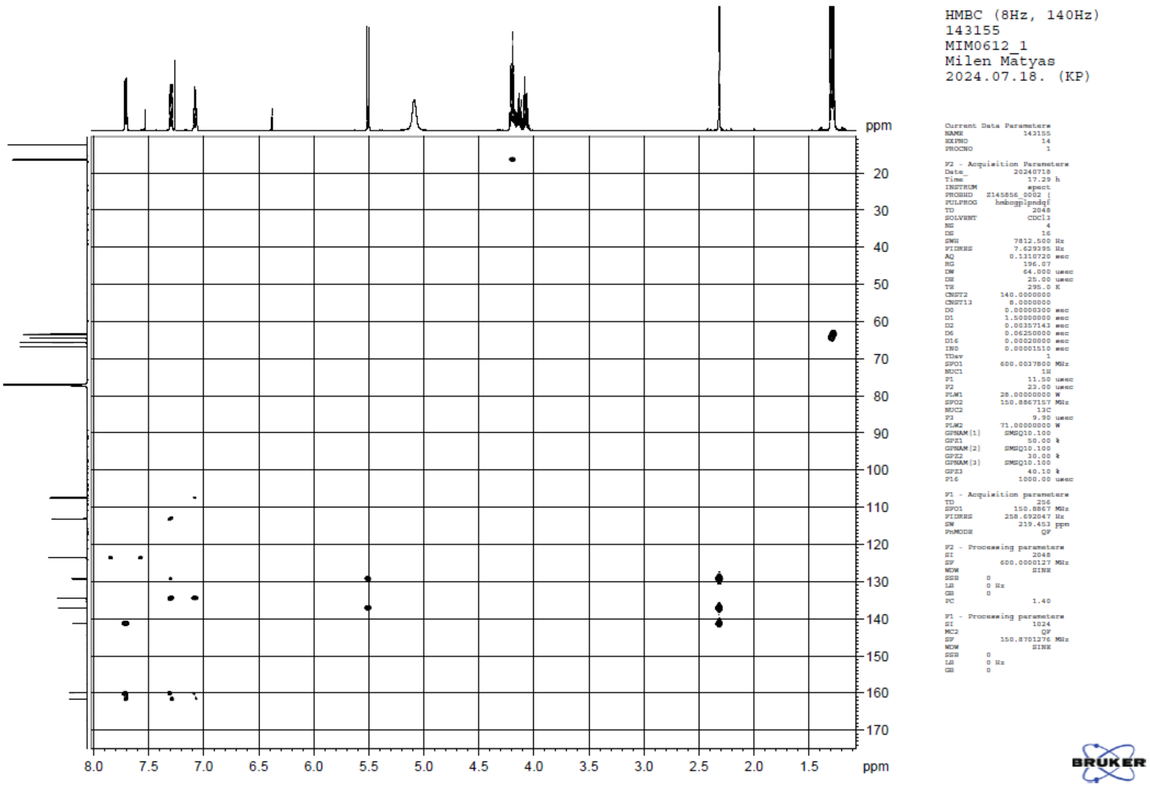

COSY NMR spectra for compound 2l

2l

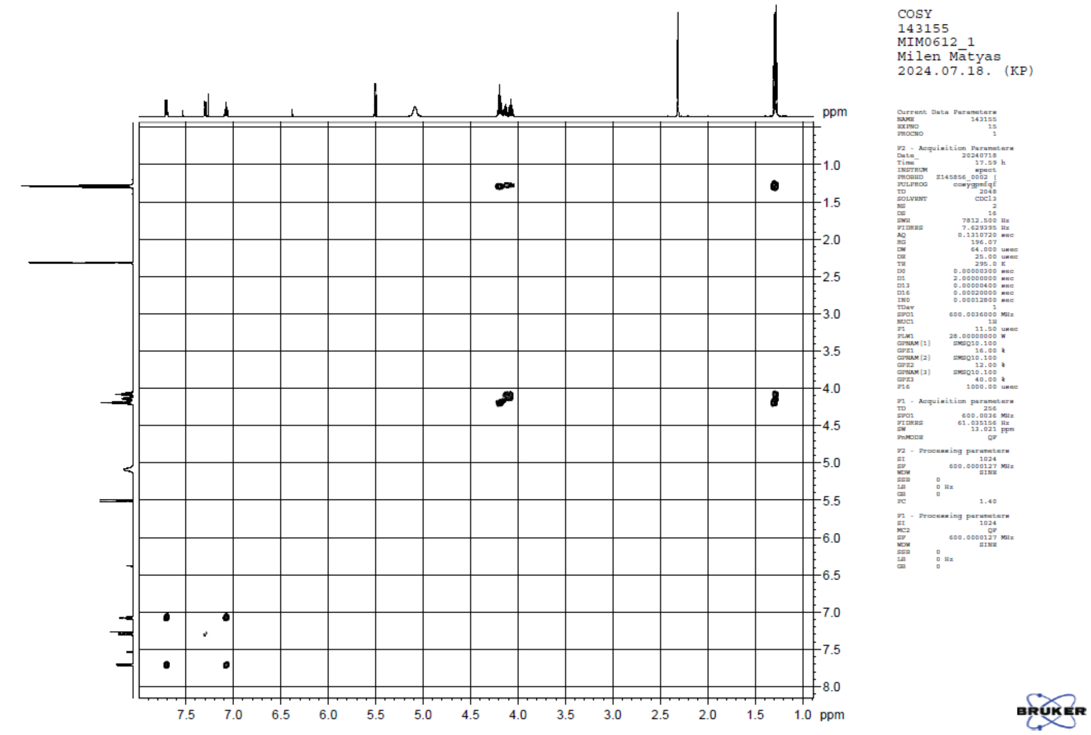

HSQC NMR (140 Hz) spectra for compound 2m

2m

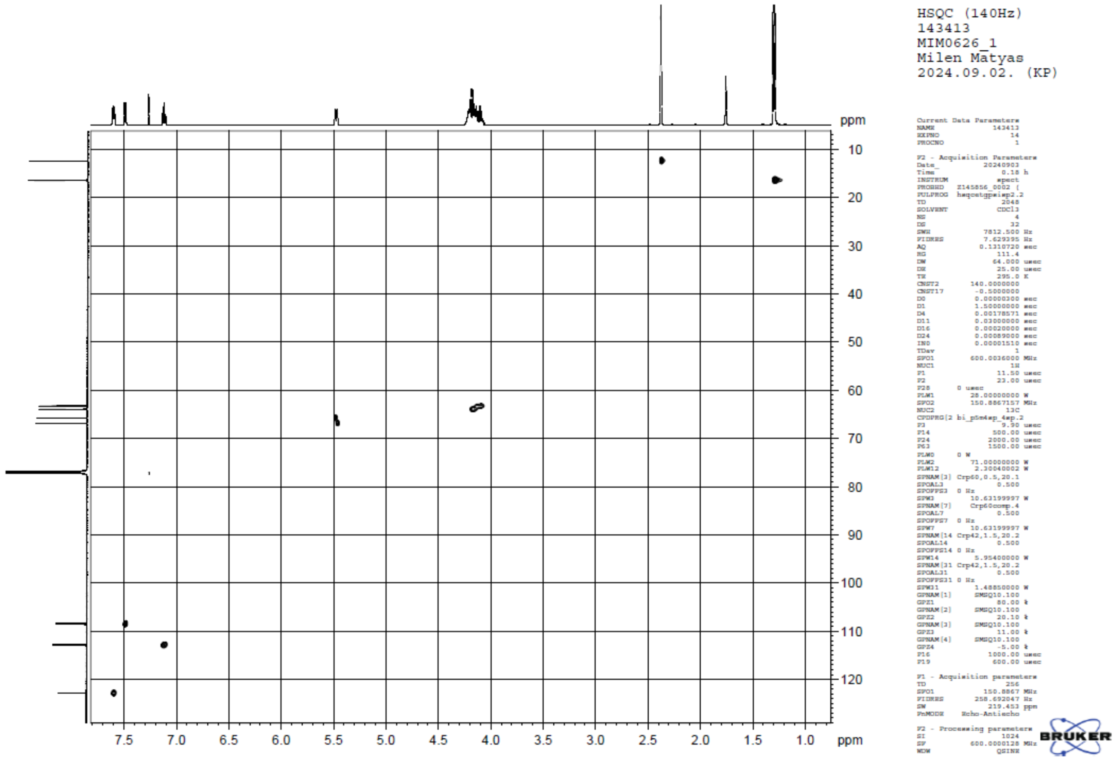

HMBC NMR (8Hz, 140 Hz) spectra for compound 2m

2m

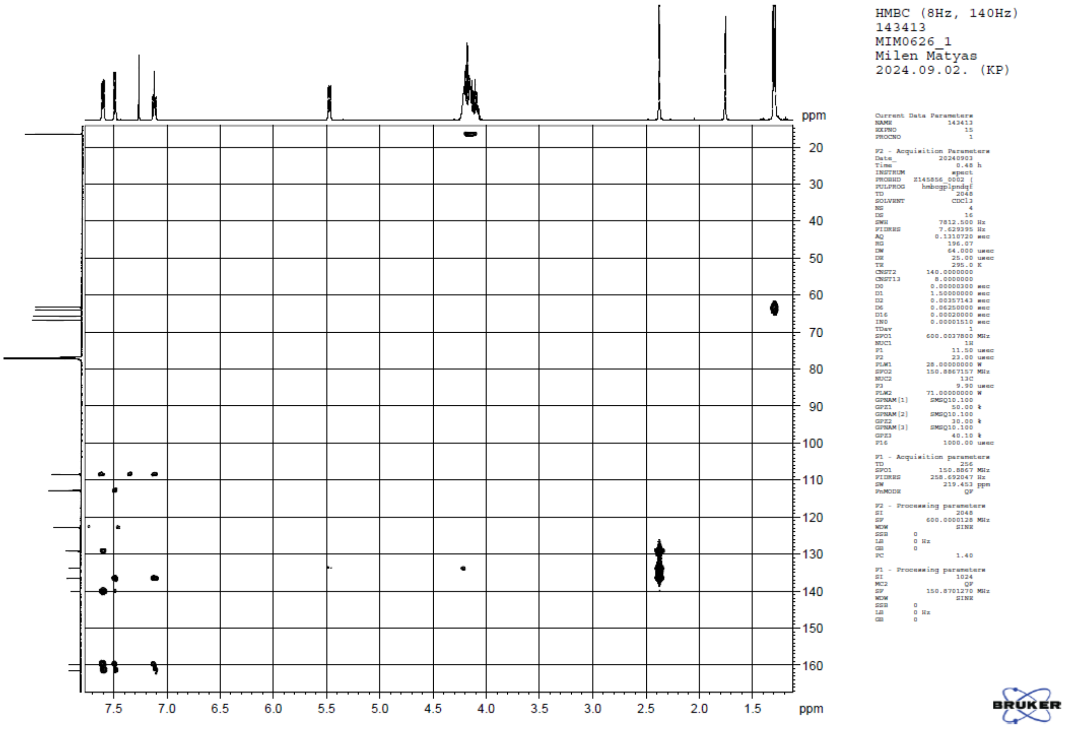

COSY NMR spectra for compound 2m

2m

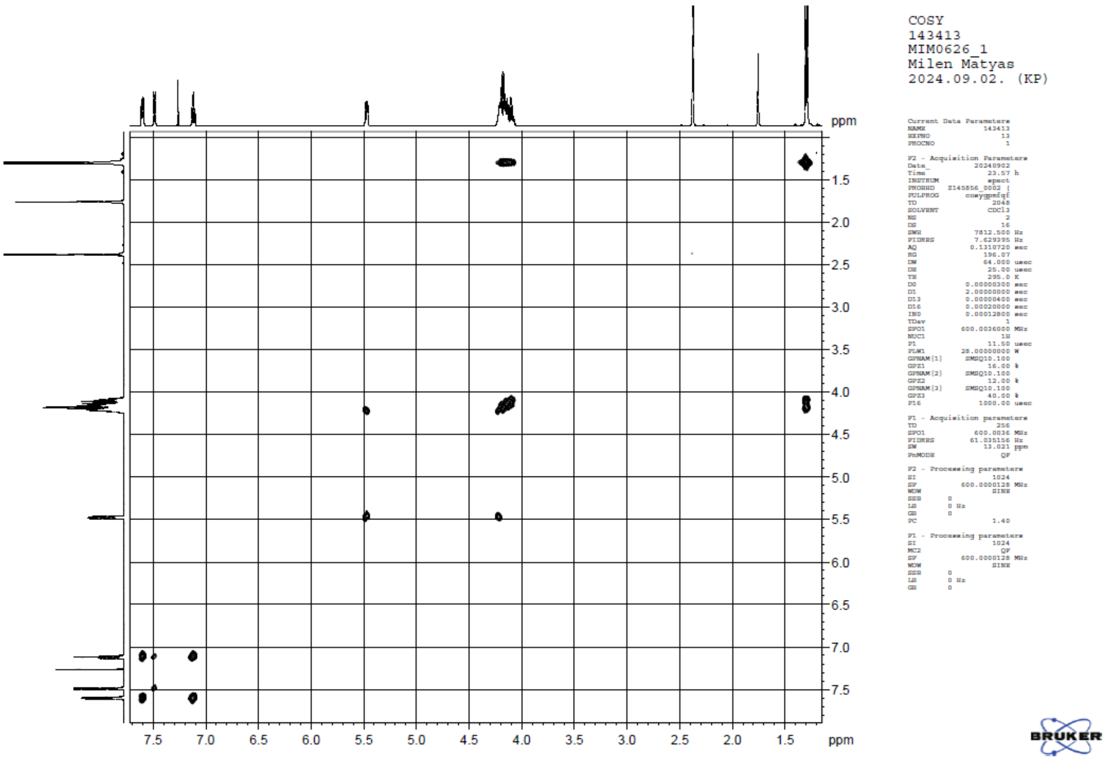

HSQC NMR (140 Hz) spectra for compound 2n

2n

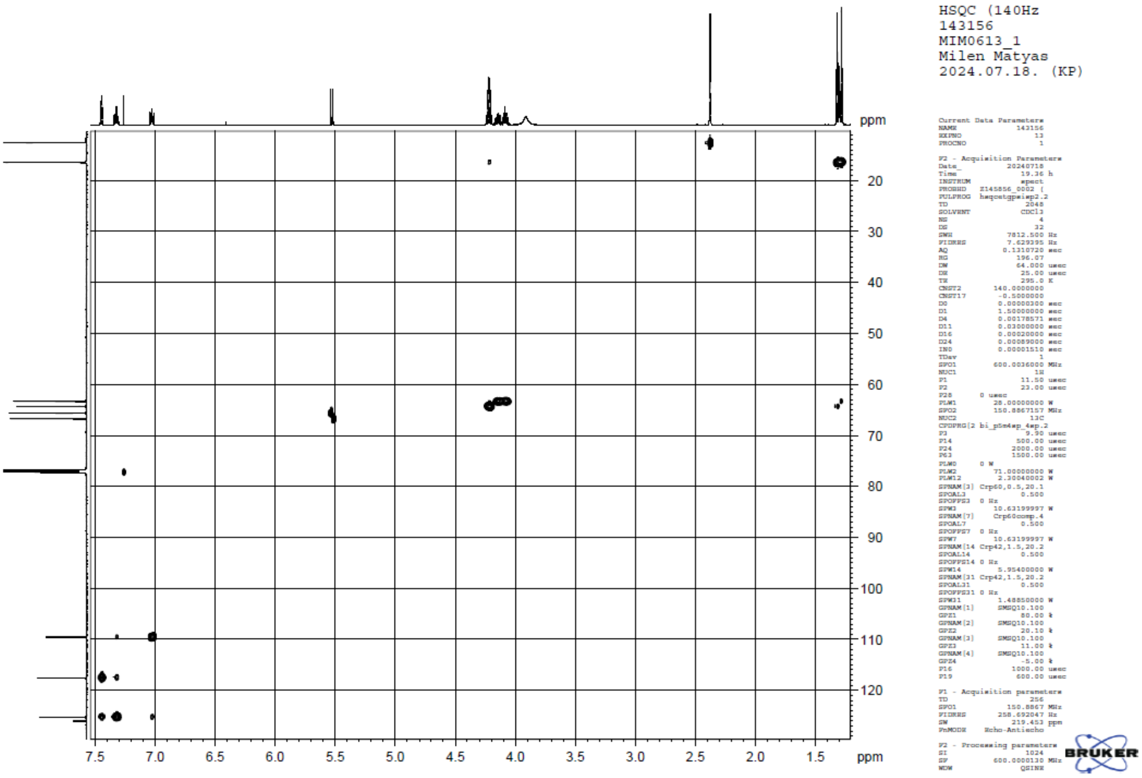

HMBC NMR (8 Hz, 140 Hz) spectra for compound 2n

2n

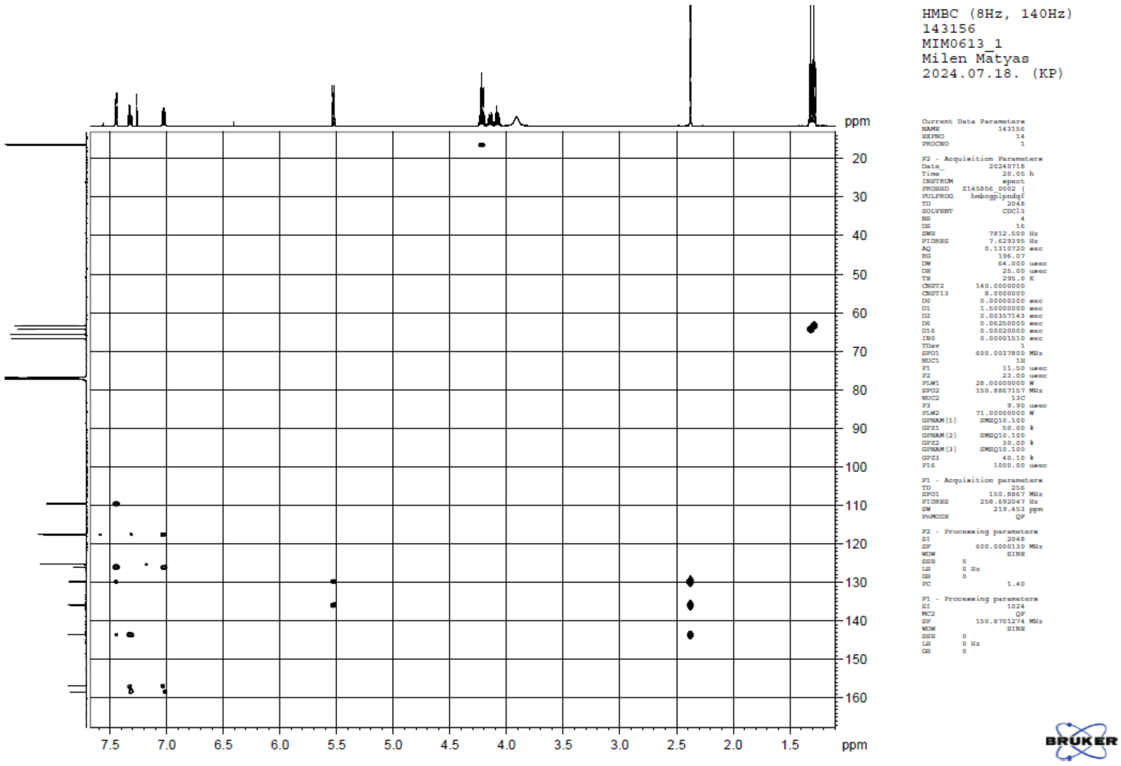

COSY NMR spectra for compound 2n

2n

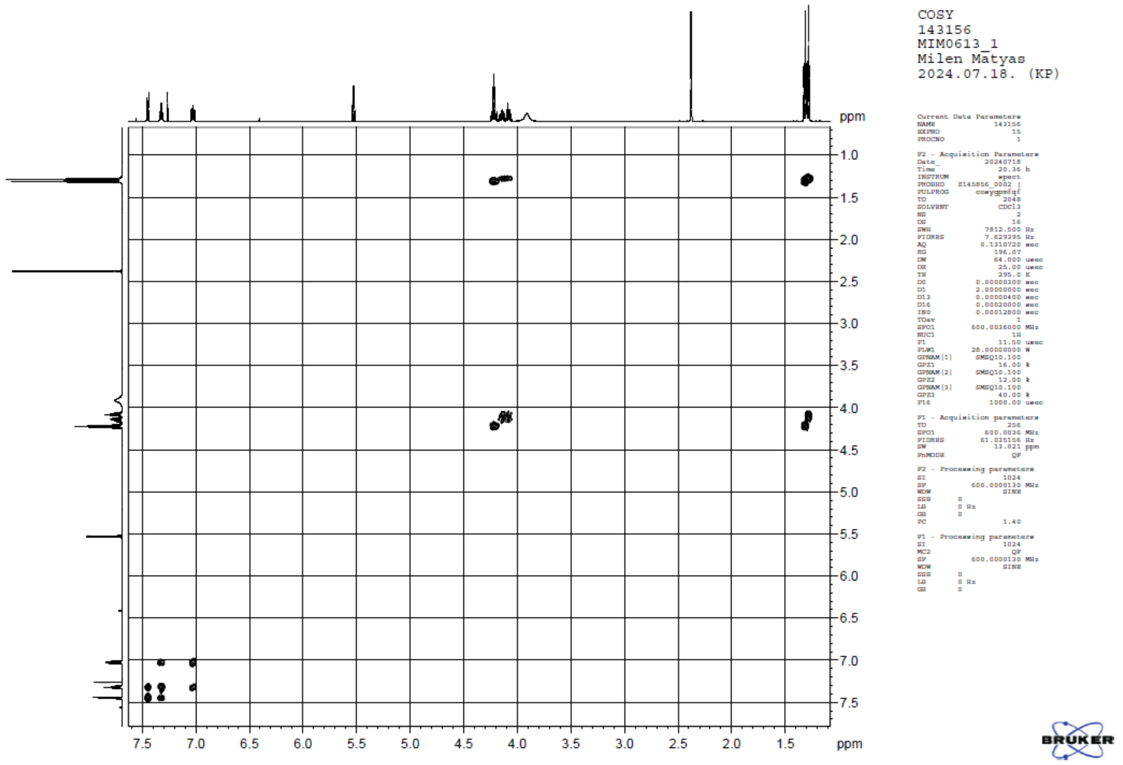

HSQC NMR (140 Hz) spectra for compound 2o

2o

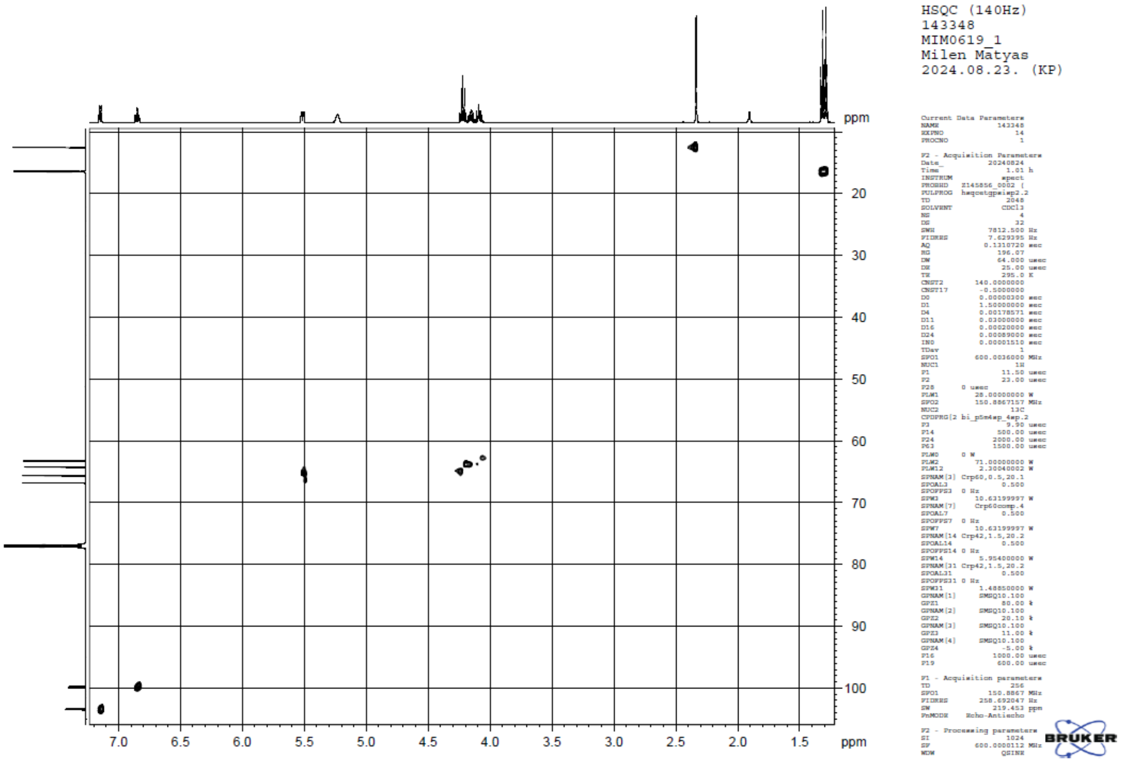

HMBC NMR (8 Hz, 140 Hz) spectra for compound 2o

2o

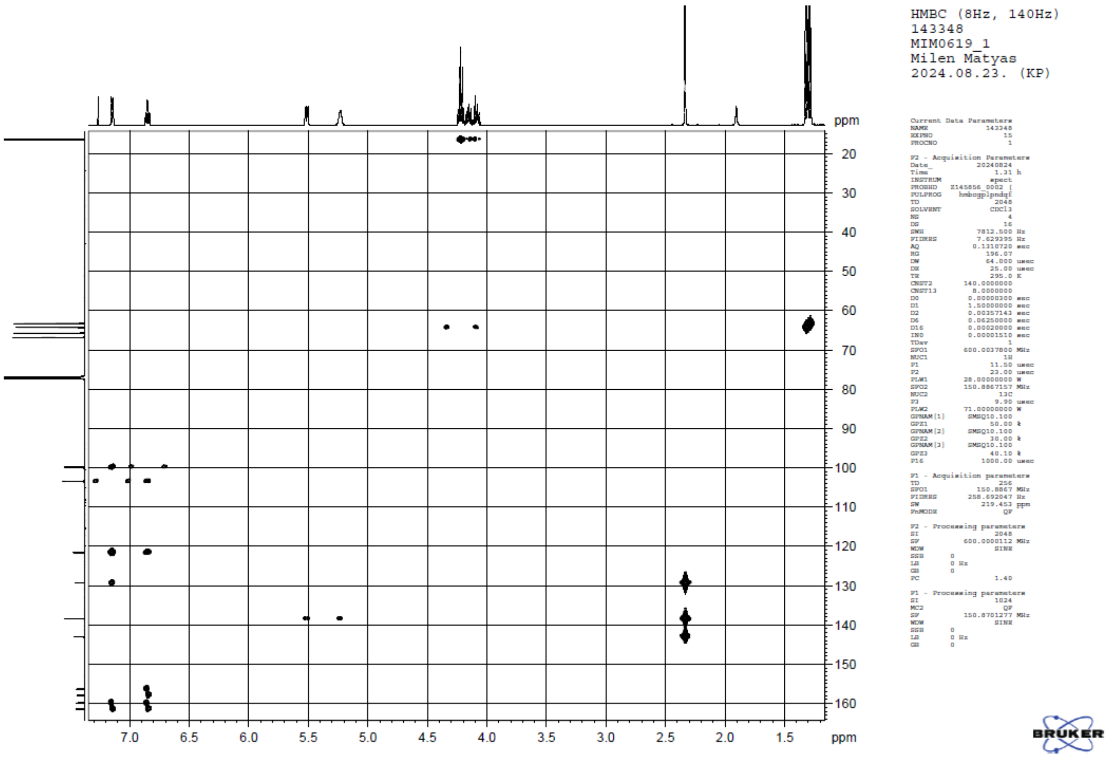

COSY NMR spectra for compound 2o

2o

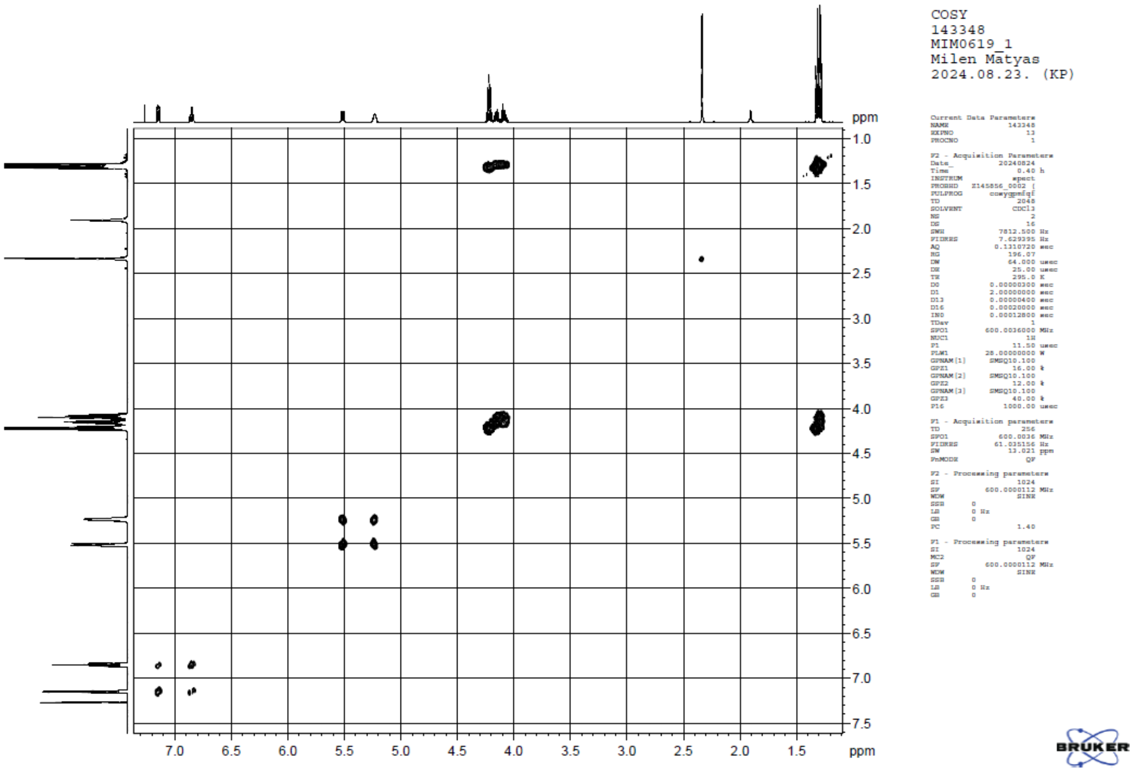

HSQC NMR (140 Hz) spectra for compound 2p

2p

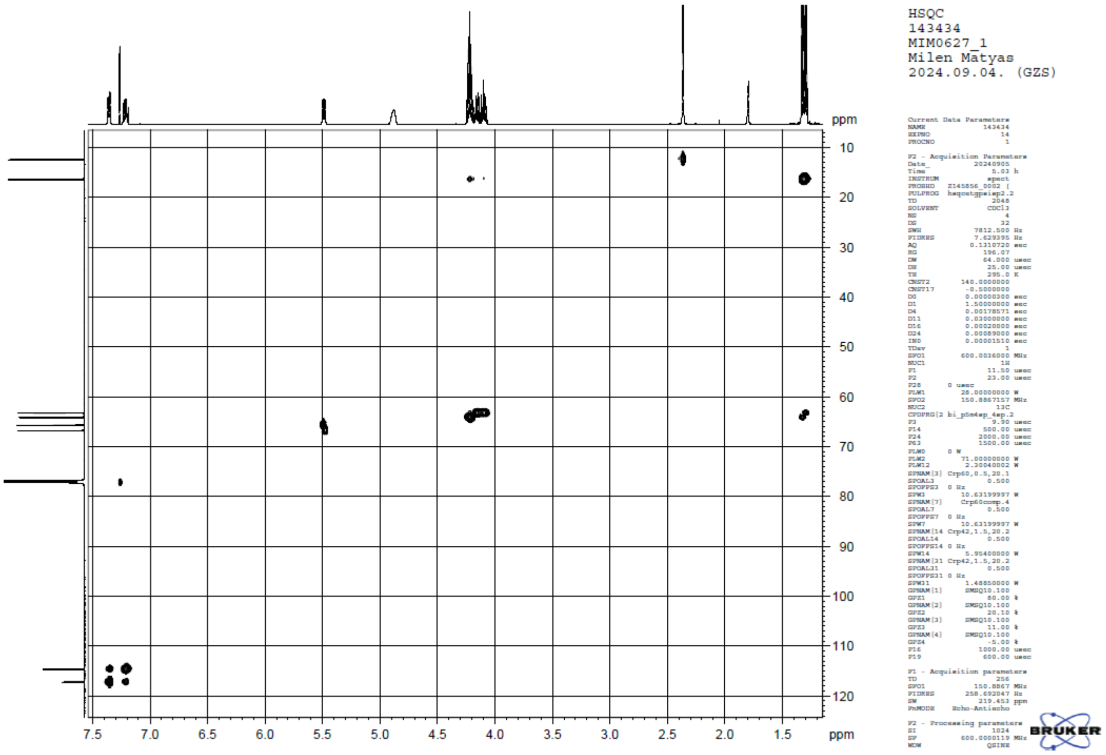

HMBC NMR (8 Hz, 140 Hz) spectra for compound 2p

2p

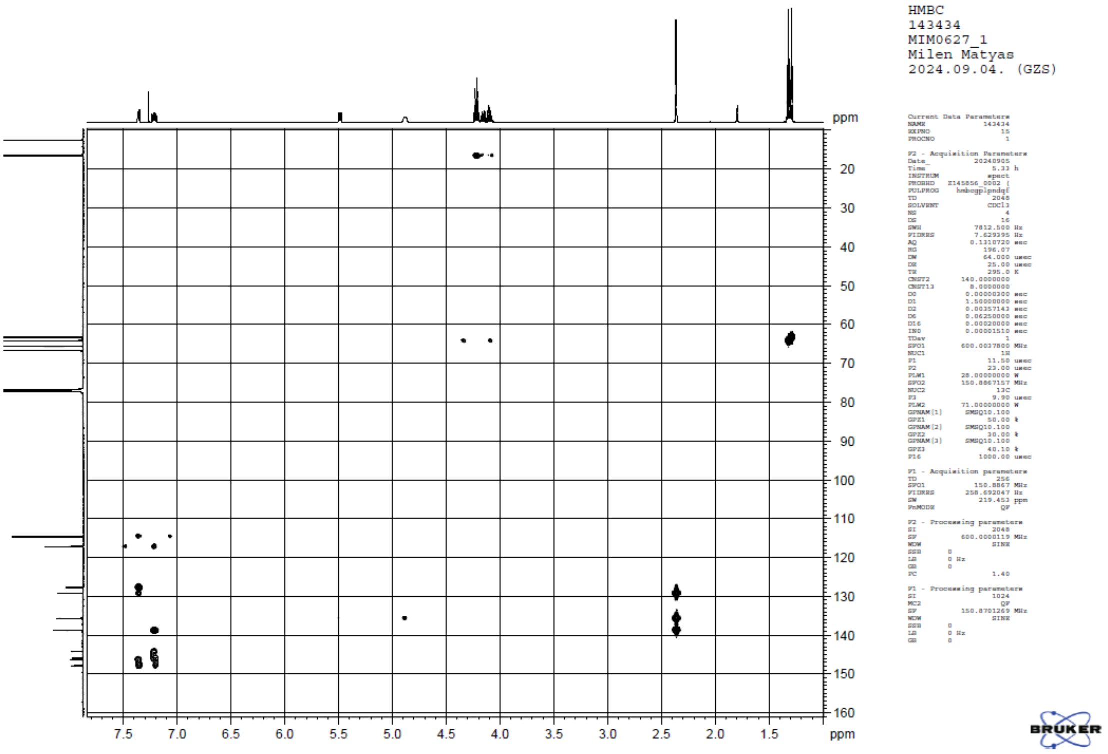

COSY NMR spectra for compound 2p

2p

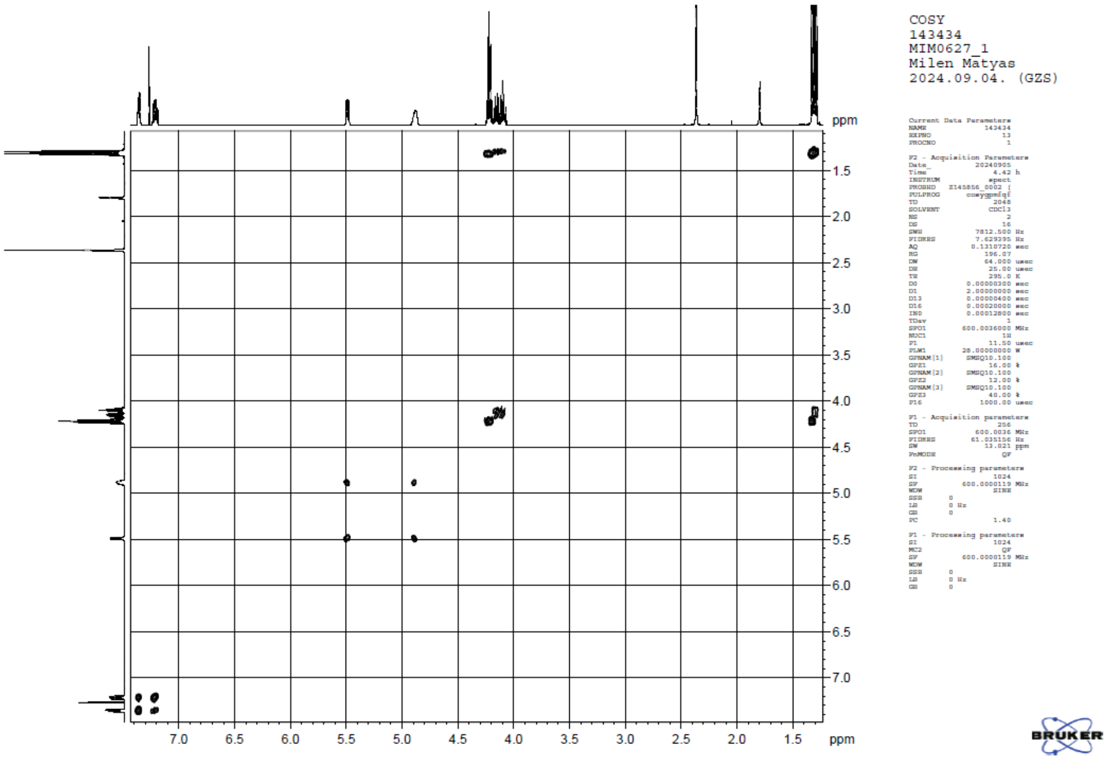

HSQC NMR (140 Hz) spectra for compound 2q

2q

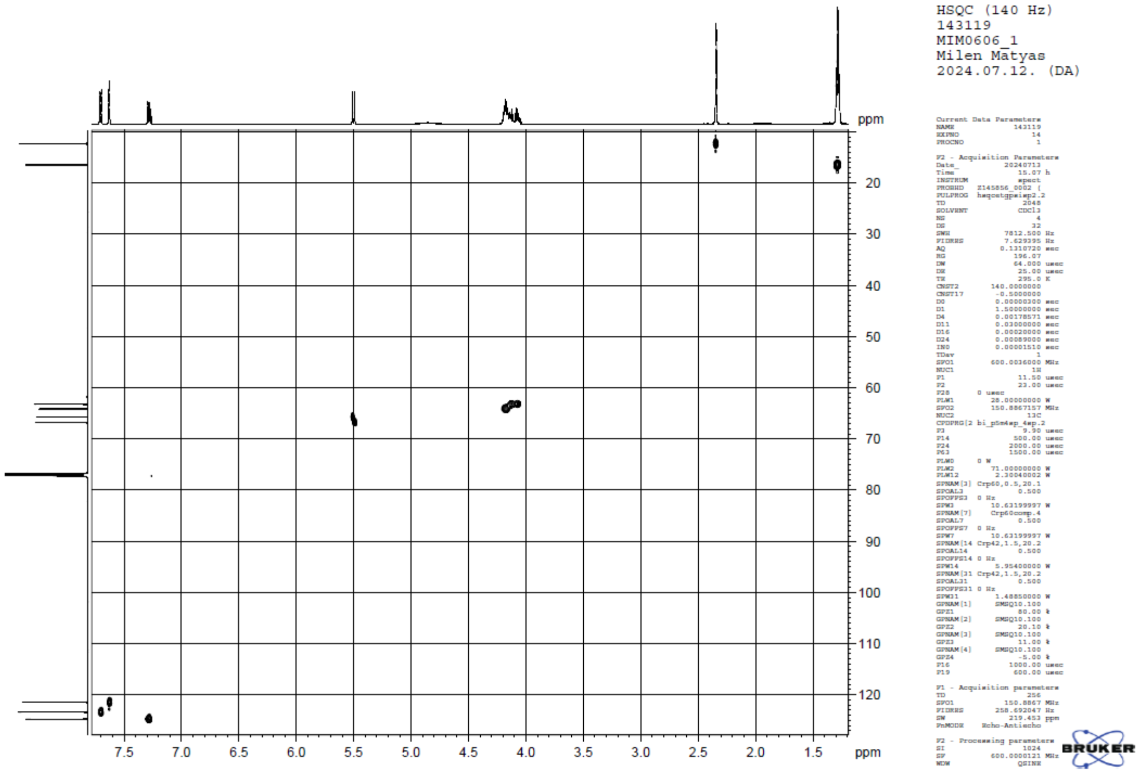

HMBC NMR (8 Hz, 140 Hz) spectra for compound 2q

2q

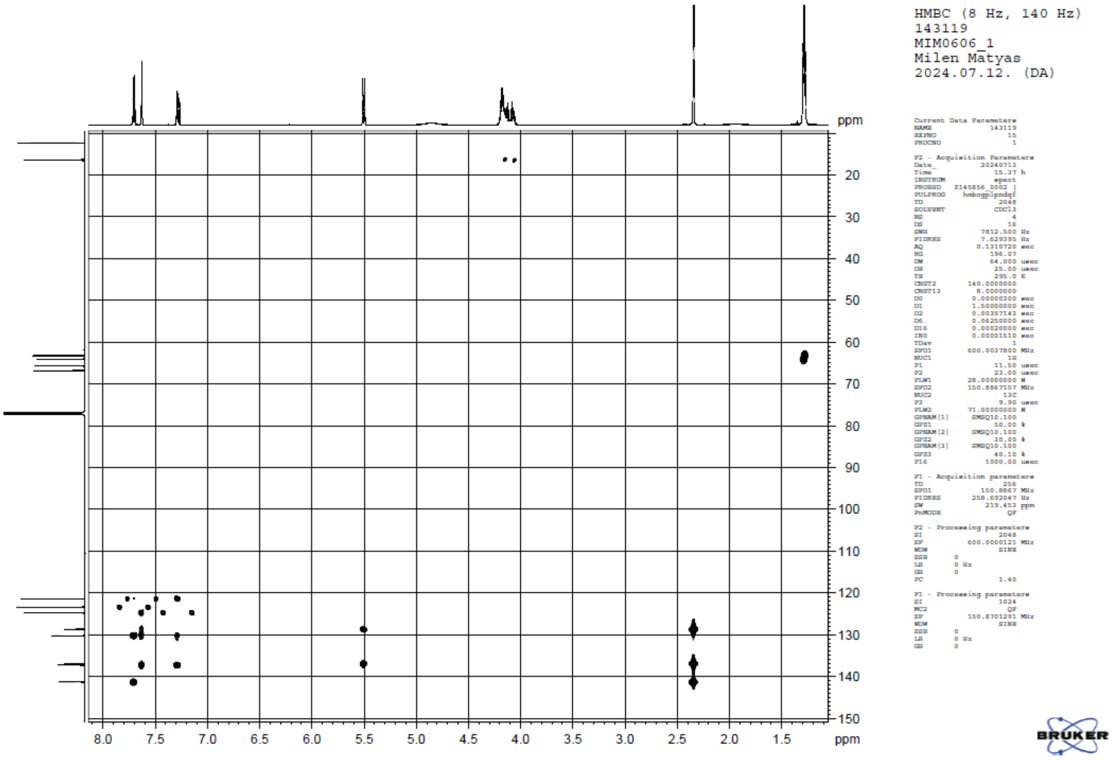

COSY NMR spectra for compound 2q

2q

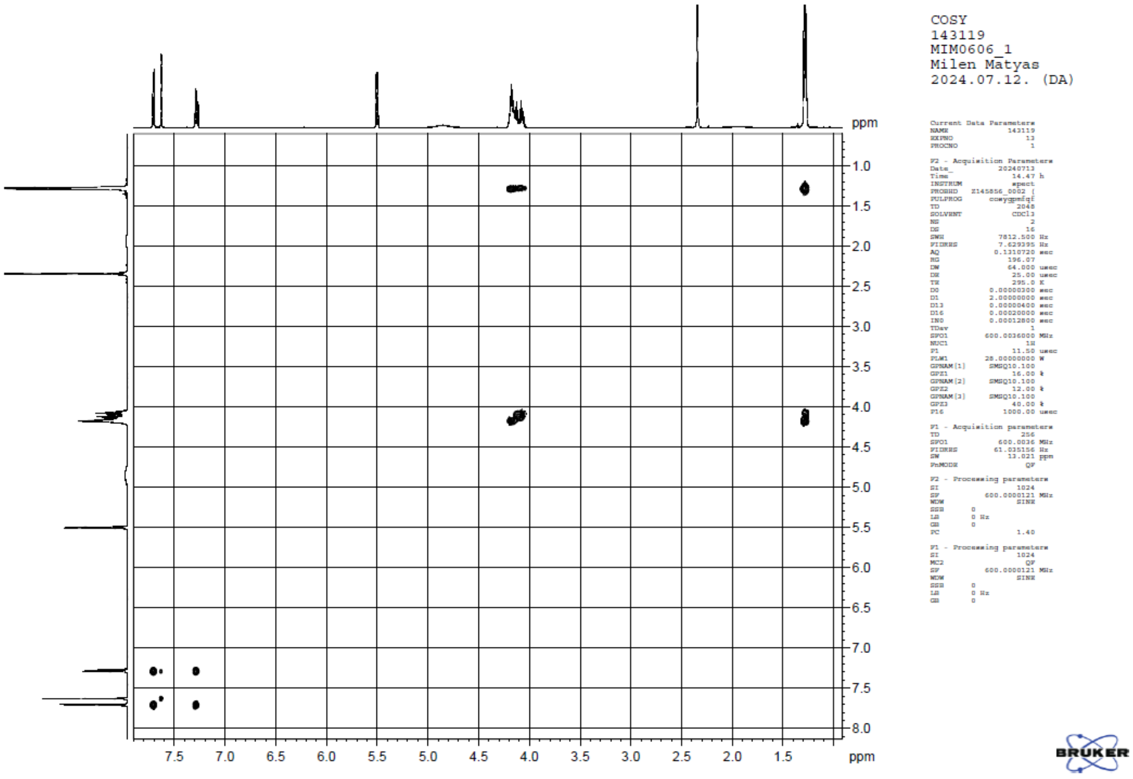

HSQC NMR (140 Hz) spectra for compound 2r

2r

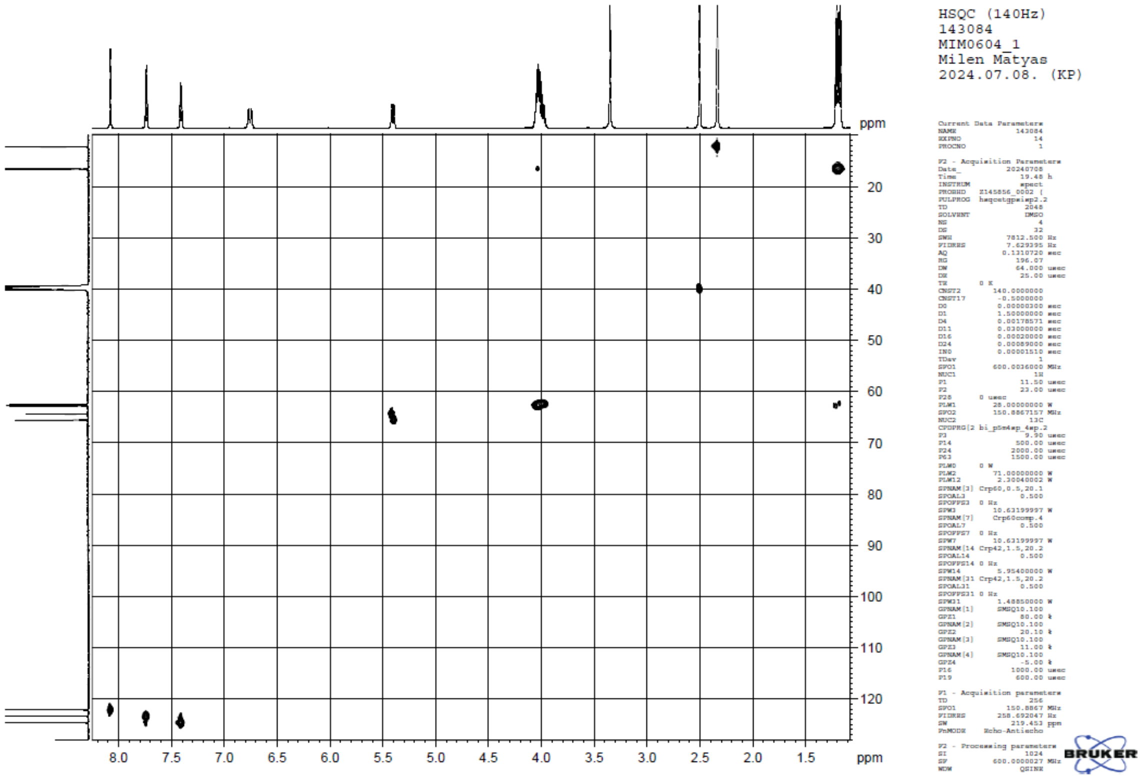

HMBC NMR (8 Hz, 140 Hz) spectra for compound 2r

2r

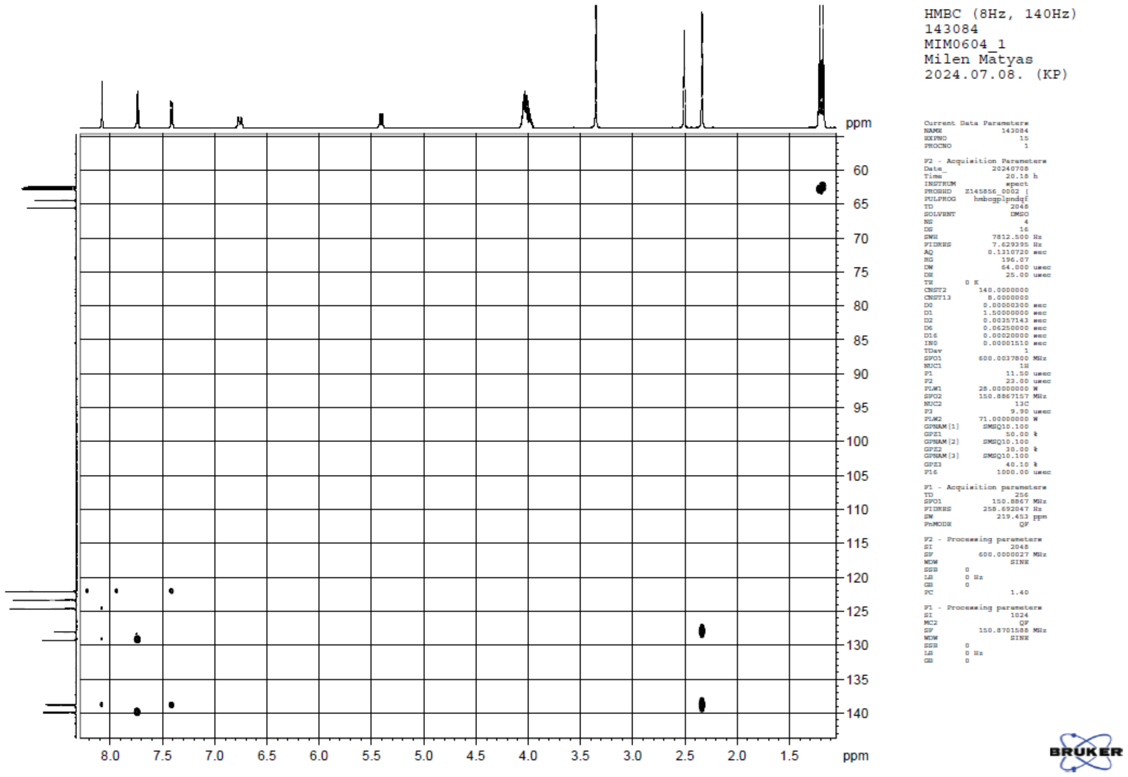

HSQC NMR (140 Hz) spectra for compound 2s

2s

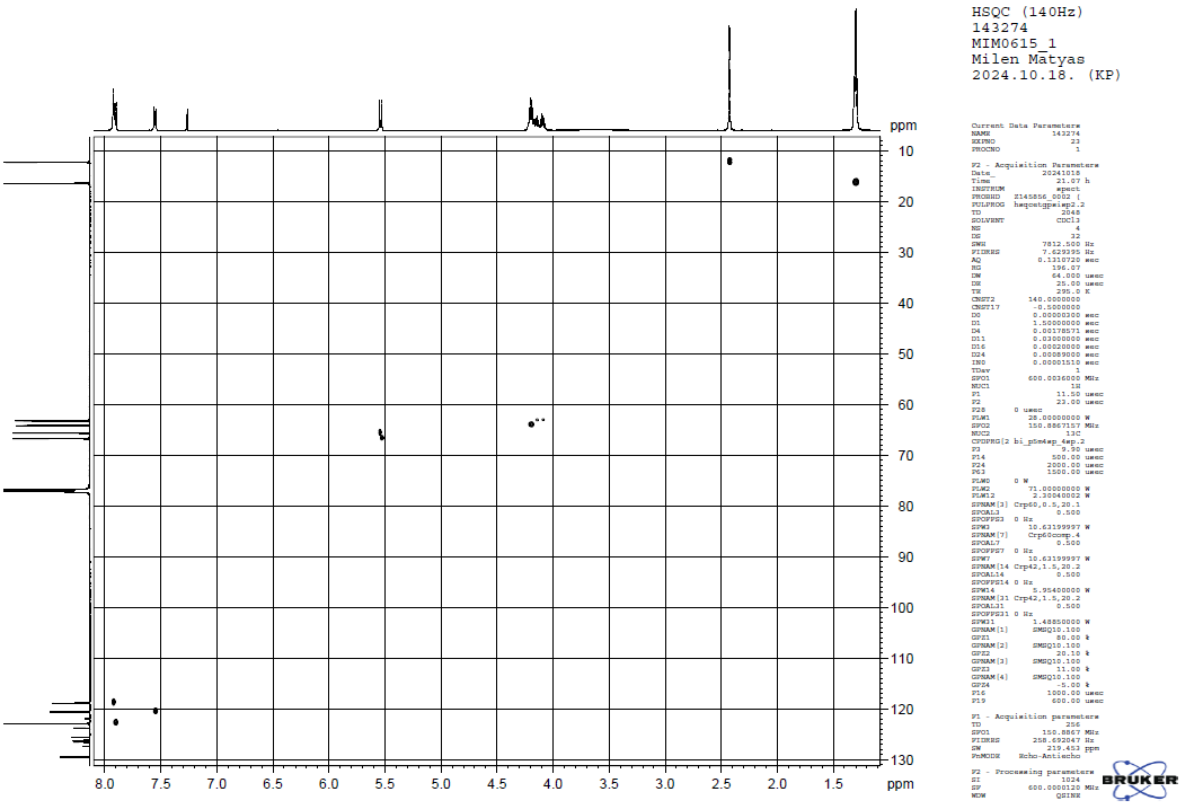

HMBC NMR (8 Hz, 140 Hz) spectra for compound 2s

2s

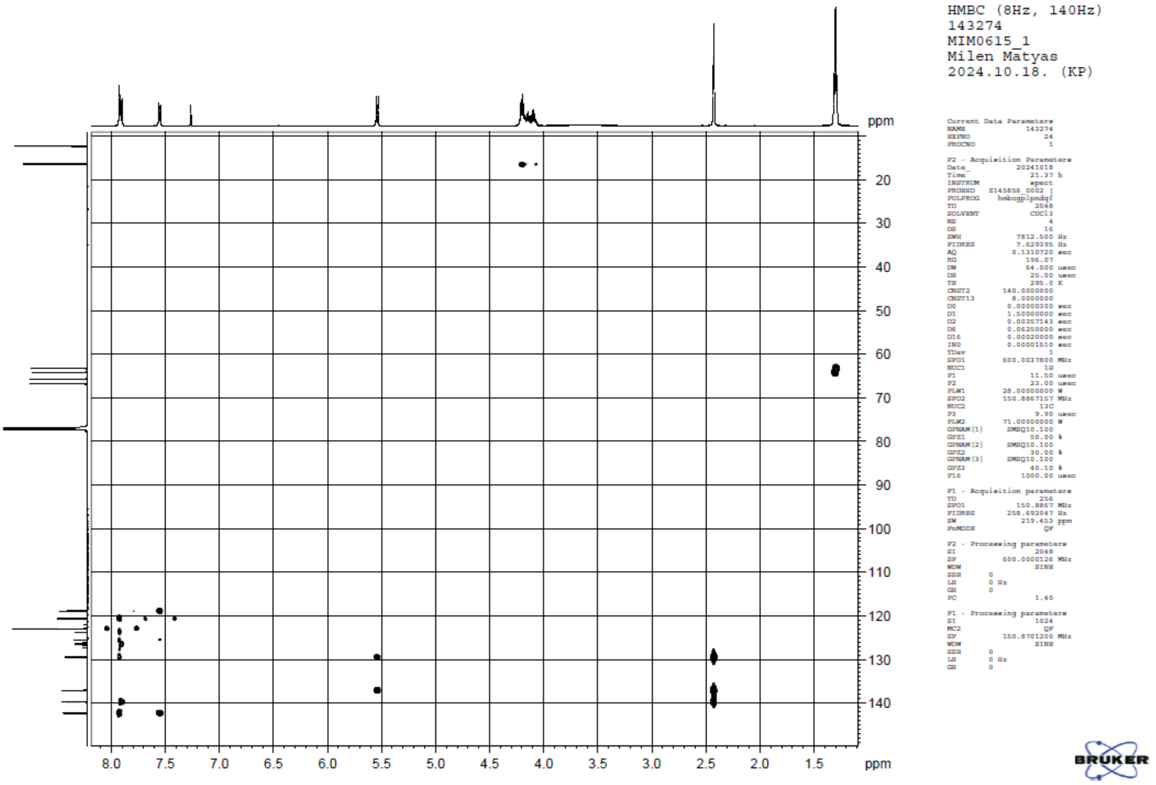

HSQC NMR (140 Hz) spectra for compound 2u

2u

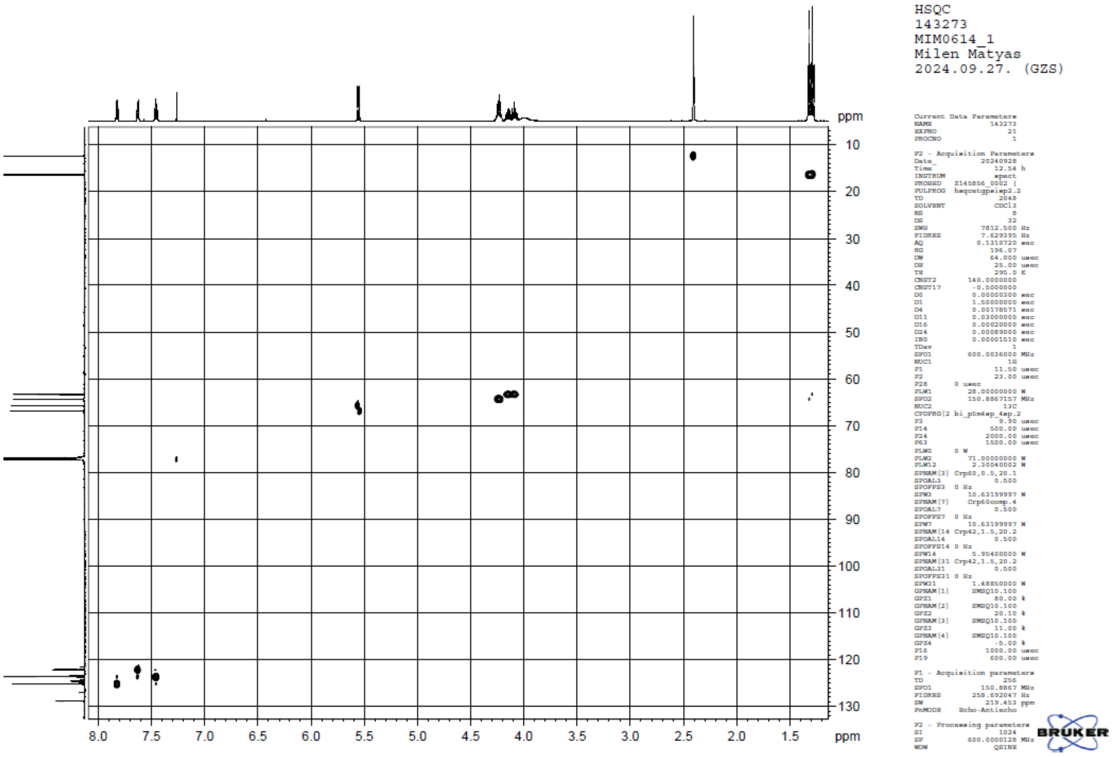

HMBC NMR (8 Hz, 140 Hz) spectra for compound 2u

2u

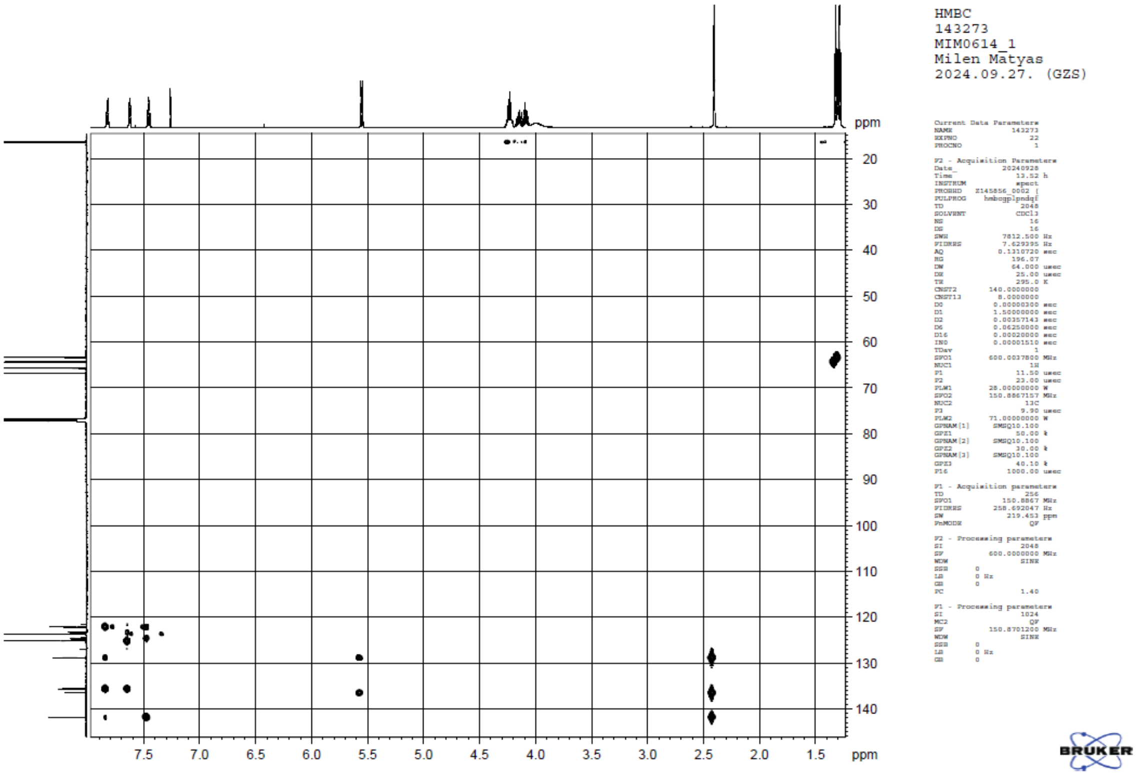

COSY NMR spectra for compound 2u

2u

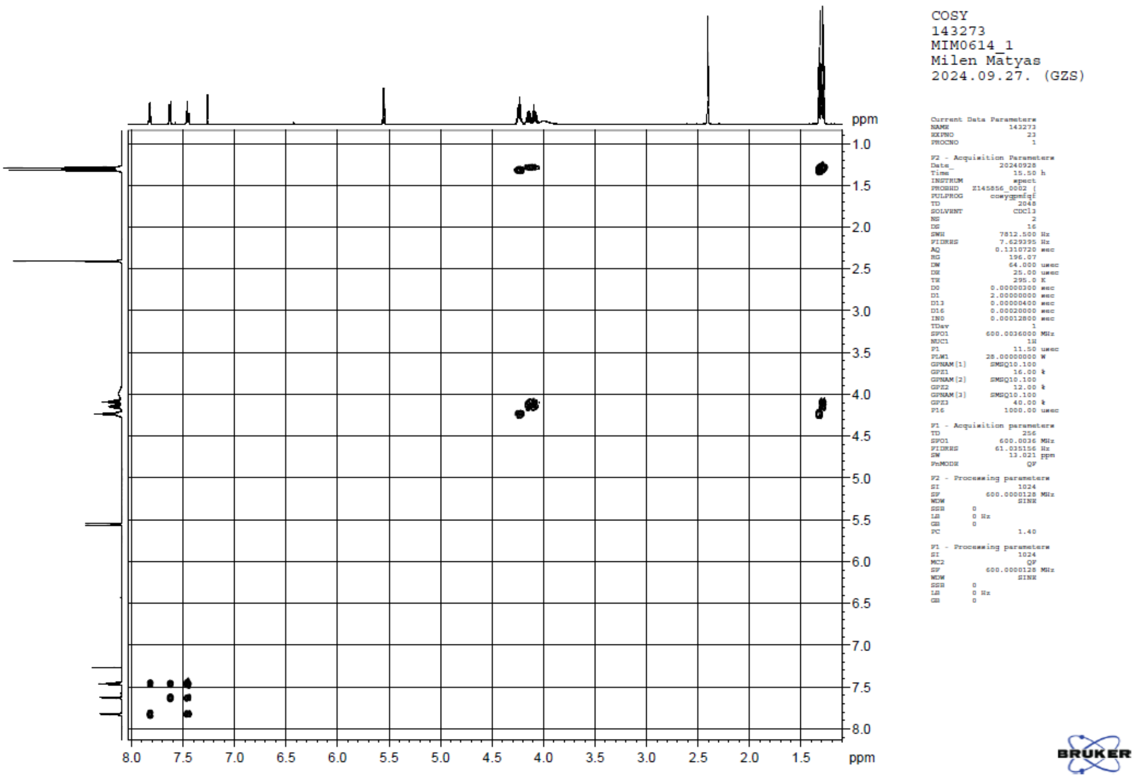

HSQC NMR (140 Hz) spectra for compound 2v

2v

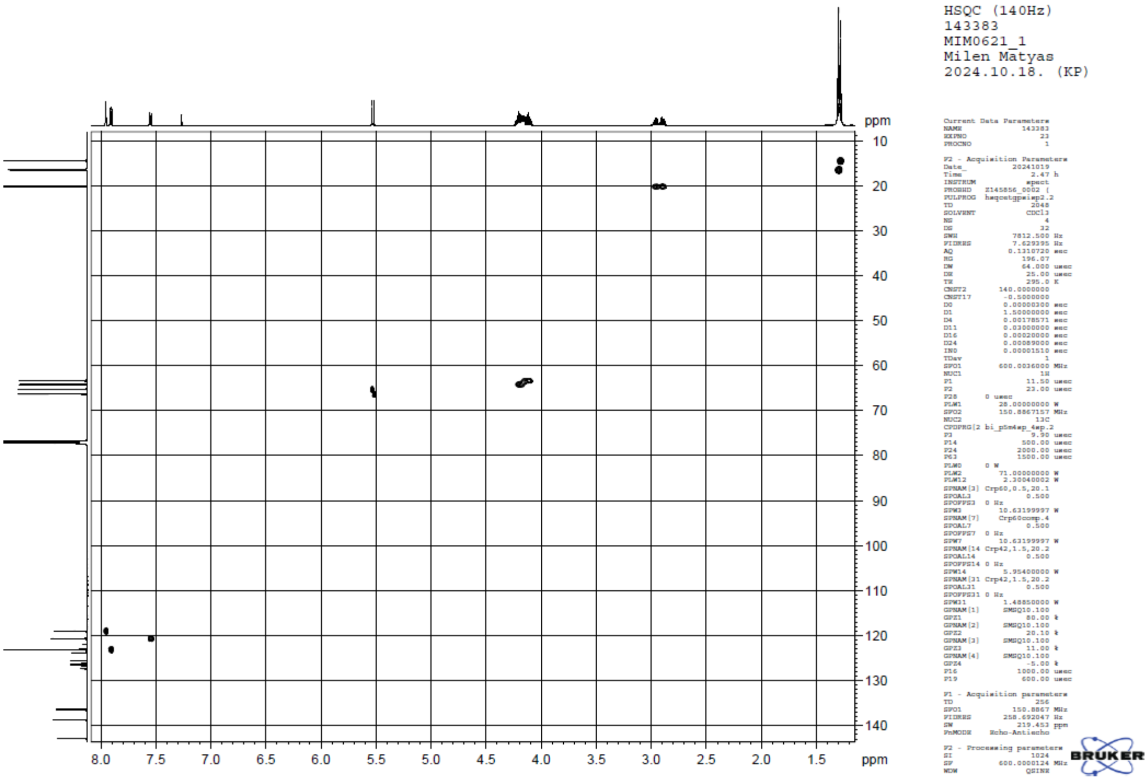

HMBC NMR (8 Hz, 140 Hz) spectra for compound 2v

2v

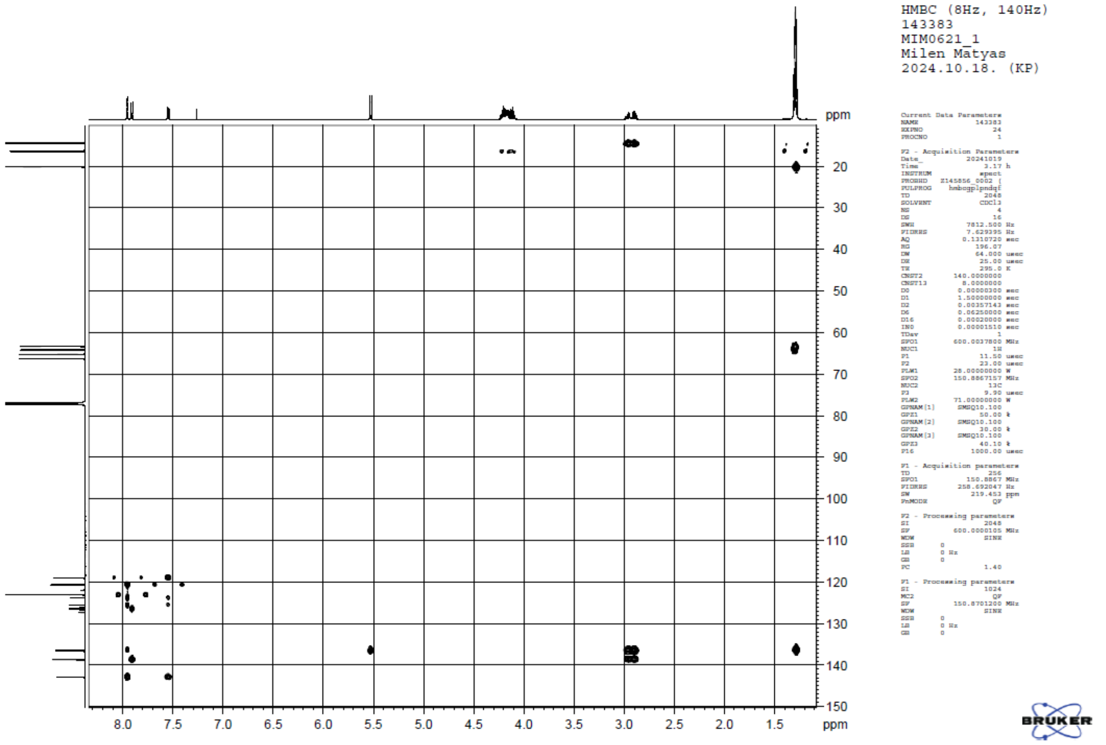

HSQC NMR (140 Hz) spectra for compound 2w

2w

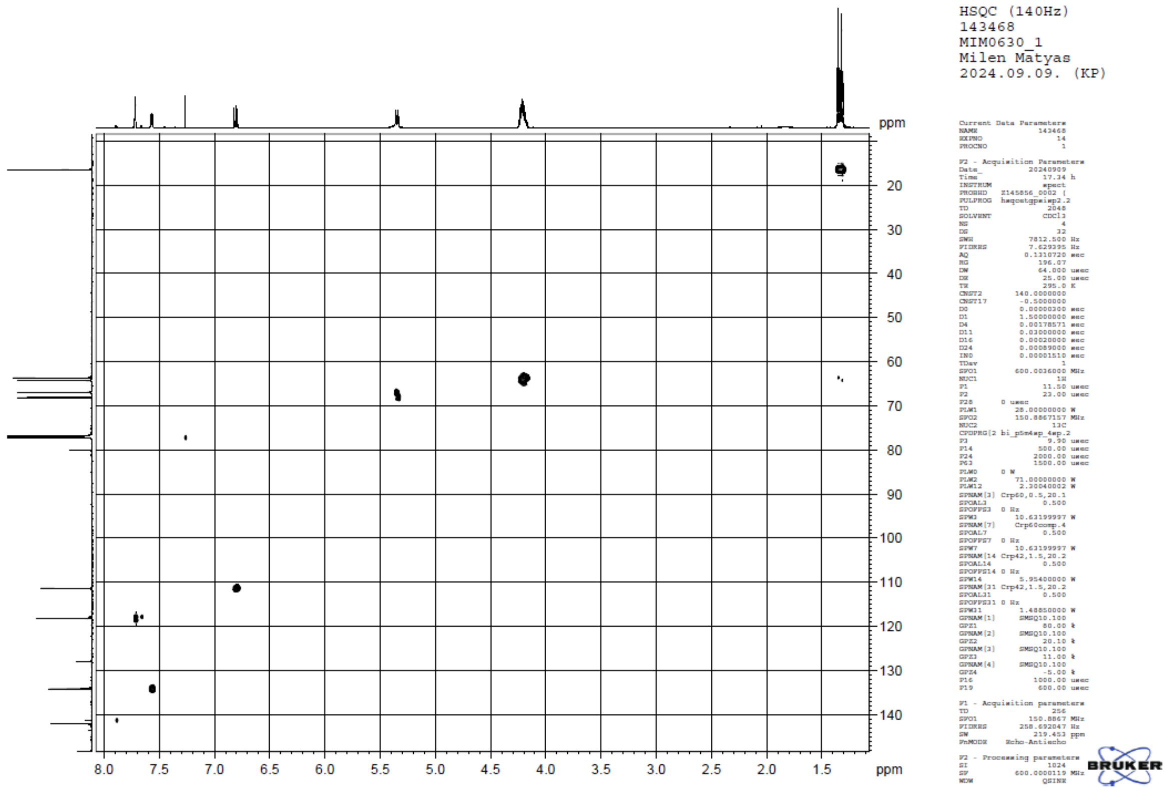

HMBC NMR (8 Hz, 140 Hz) spectra for compound 2w

2w

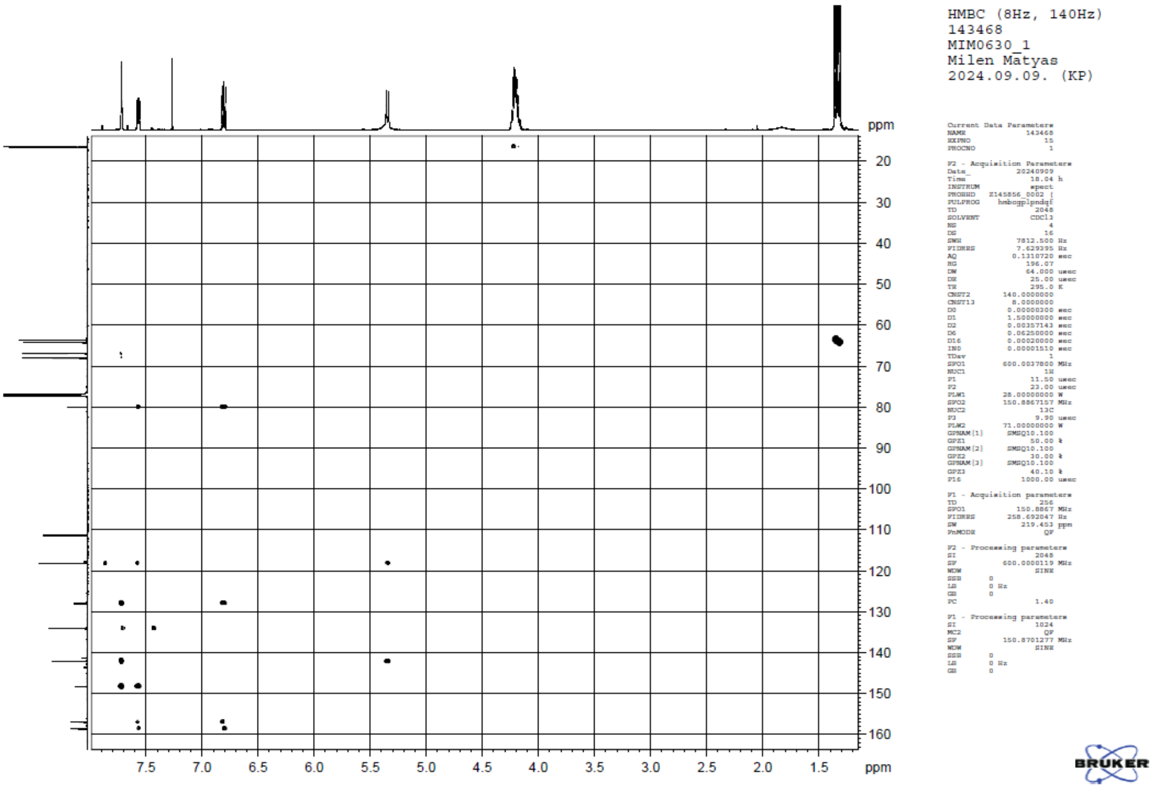

COSY NMR spectra for compound 2w

2w

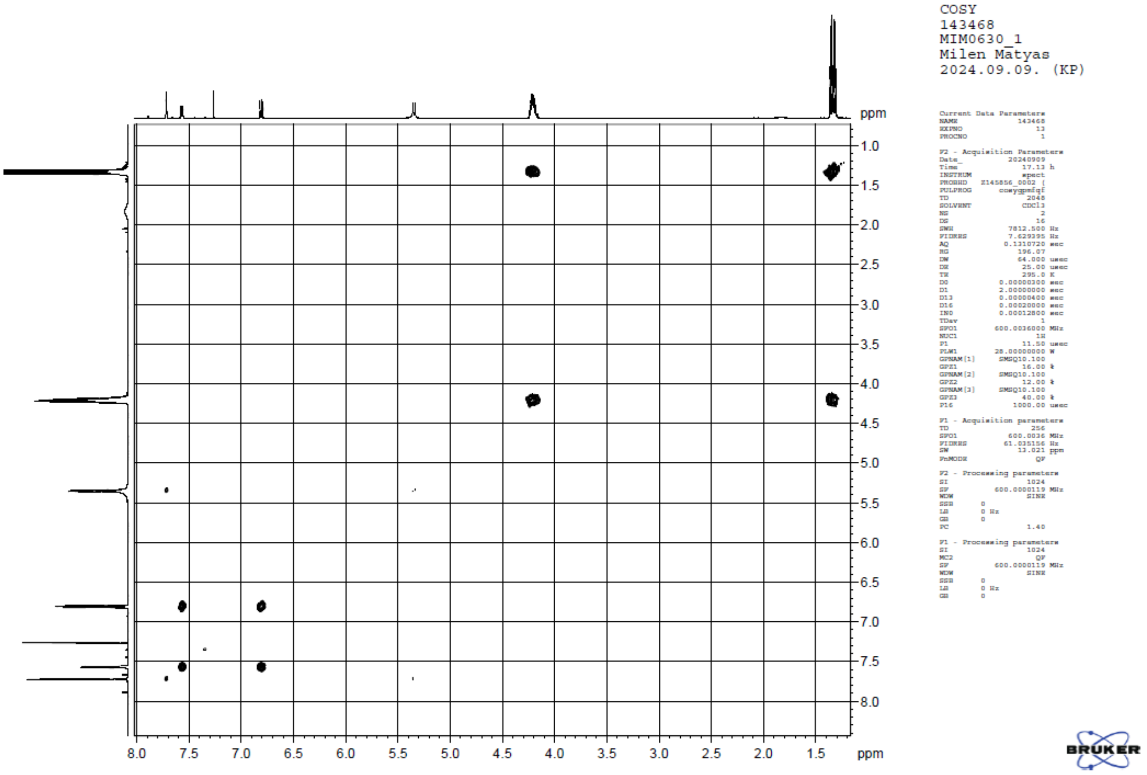

Supplement: Supplementary file 1 [file pharmaceuticals-18-00949-s001.zip › pharmaceuticals-3695123-supplementary.pdf]
